# Characterizing glycosyltransferases by a combination of sequencing platforms applied to the leaf tissues of *Stevia rebaudiana*

**DOI:** 10.1186/s12864-020-07195-5

**Published:** 2020-11-13

**Authors:** Shaoshan Zhang, Qiong Liu, Chengcheng Lyu, Jinsong Chen, Renfeng Xiao, Jingtian Chen, Yunshu Yang, Huihui Zhang, Kai Hou, Wei Wu

**Affiliations:** 1 Agronomy College, Sichuan Agricultural University, Chengdu, 611130 China; 2 Institute of Qinghai-Tibetan Plateau, Southwest Minzu University, Chengdu, 610041 China

**Keywords:** *Stevia rebaudiana* (Bertoni), Next-generation sequencing, Single-molecule real-time sequencing, Glycosyltransferase, Phylogenetic tree

## Abstract

**Background:**

*Stevia rebaudiana* (Bertoni) is considered one of the most valuable plants because of the steviol glycosides (SGs) that can be extracted from its leaves. Glycosyltransferases (GTs), which can transfer sugar moieties from activated sugar donors onto saccharide and nonsaccharide acceptors, are widely distributed in the genome of *S. rebaudiana* and play important roles in the synthesis of steviol glycosides.

**Results:**

Six stevia genotypes with significantly different concentrations of SGs were obtained by induction through various mutagenic methods, and the contents of seven glycosides (stevioboside, Reb B, ST, Reb A, Reb F, Reb D and Reb M) in their leaves were considerably different. Then, NGS and single-molecule real-time (SMRT) sequencing were combined to analyse leaf tissue from these six different genotypes to generate a full-length transcriptome of *S. rebaudiana*. Two phylogenetic trees of glycosyltransferases (SrUGTs) were constructed by the neighbour-joining method and successfully predicted the functions of SrUGTs involved in SG biosynthesis. With further insight into glycosyltransferases (SrUGTs) involved in SG biosynthesis, the weighted gene co-expression network analysis (WGCNA) method was used to characterize the relationships between SrUGTs and SGs, and forty-four potential SrUGTs were finally obtained, including *SrUGT85C2*, *SrUGT74G1*, *SrUGT76G1* and *SrUGT91D2,* which have already been reported to be involved in the glucosylation of steviol glycosides, illustrating the reliability of our results.

**Conclusion:**

Combined with the results obtained by previous studies and those of this work, we systematically characterized glycosyltransferases in *S. rebaudiana* and forty-four candidate *SrUGTs* involved in the glycosylation of steviol glucosides were obtained. Moreover, the full-length transcriptome obtained in this study will provide valuable support for further research investigating *S. rebaudiana*.

**Supplementary Information:**

The online version contains supplementary material available at 10.1186/s12864-020-07195-5.

## Background

*Stevia rebaudiana* (Bertoni) belongs to the Asteraceae family and is also one of the only two members (*S. rebaudiana* and *S. phlebophylla*) in this genus to produce steviol glycosides (SGs), compounds that appeal to people looking for more natural plant-based low-calorie sustainable sweeteners [1, 2]. Therefore, making stevia a leaf crop with significant economic value and the food industry has a very positive outlook regarding the opportunities for SGs. Consequently, the value of the SG market is expected to exceed $1 billion USD by 2021 [3]. To date, more than thirty-five SGs have been isolated and identified from *S. rebaudiana*, including steviolbioside, rebaudioside A-Q (Reb A-Q), 1,2-stevioside (ST), dulcoside A, dulcoside B and rubusoside [4, 5]. Notable progress has been made in elucidating the biosynthetic pathway of SGs [6, 7]. Taking steviol, the precursor of SGs, as an example, its biosynthesis in stevia was largely determined to include nine enzyme-catalysed reactions from isopentenyl diphosphate/dimethylallyl diphosphate [8]. With the discovery of new glycosides and the characteristics of genetic heterozygosity of stevia [9, 10], elucidating the biosynthetic pathways (especially for the enzymes in the UDPG-dependent glucosyltransferase (UGT) family, which play a critical role in the production of SGs) and regulatory mechanisms of active SGs has attracted the attention of scientists.

Owing to the interest in the properties of SGs, there has been extensive transcriptome research investigating stevia. An early report used expressed sequence tags (ESTs) to identify candidate UGTs involved in the glucosylation of SGs and successfully collected more than 5500 fully annotated ESTs from *S. rebaudiana* leaf; finally, twelve UGTs (SrUGT73E1, SrUGT89B2, SrUGT76G1, SrUGT76H1, SrUGT85C2, SrUGT85C1, SrUGT85A8, SrUGT74G1, SrUGT88B1, SrUGT71E1, SrUGT79A2, and SrUGT91D1) were functionally characterized and three of them (SrUGT85C2, SrUGT74G1, SrUGT76G1) that were responsible for the glucosylation reactions leading from steviol to Reb A were obtained [6, 11]. In 2014, Chen et al. used next-generation sequencing (NGS)-based RNA-Seq technology (Illumina RNA-Seq) to sequence three stevia genotypes with different Reb A and ST contents. A total of 191,590,282 high-quality reads were generated, and 80,160 assembled contigs were obtained and their average length was 969 bp [12]. In this study, a total of 143 assembled contigs were annotated as glycosyltransferase genes, but none of them had been functionally characterized. Subsequent RNA-Seq analysis (Illumina platform) of the stevia leaves in two different growing stages yielded twenty-three upregulated SrUGTs, but none of them had desired activity and the name & sequence of these genes were not provided in this paper; however, three SrUGT91D2 cDNA variants (designated as UGT91D2_#1, UGT91D2_#2, and UGT91D2_#3) that were not among the differentially expressed SrUGTs in this RNA-Seq were cloned from five individual plants and detected to catalyse the formation of the 1,6-β-D-glucosidic linkage of SGs [7, 13]. Moreover, there were still some other Illumina sequences of stevia [14], and these efforts provided abundant transcriptome data for stevia.

Because of technical limitations, the reported average lengths of the isotigs from the Illumina platform are < 500 bp and generally need assembly to obtain full-length transcripts, resulting in redundancy and distortion of the data [15]. Single-molecule real-time (SMRT) long-read sequencing technology (Pacific Biosciences of California, Inc., http://www.pacificbiosciences.com/), known as a third-generation sequencing platform, is the most reliable means of sequencing full-length cDNA molecules and is widely used in genome sequencing because of its long reads (average 4–8 kb), higher throughput, faster detection speed and fewer systematic errors caused by in vitro reverse transcription [16, 17]. Consequently, the use of SMRT sequencing could offer access to more complete transcriptome data [15, 18]. At the 2017 International Nutrition Conference, it was reported that the genome and the full-length sequence of three commercial stevia varieties were sequenced on the PACBIO platform and fully annotated, but the data were not published [3]. Therefore, providing the available full-length sequence for each RNA, especially for those corrected by NGS reads, plays a key role for researchers to understand and improve existing steviol glycoside biosynthesis pathways or discover new pathways or compounds and through traditional breeding for non-GMO improvement. In the current study, we combined NGS and SMRT sequencing approaches to sequence six stevia varieties with different accumulation levels of SGs and then generated a full-length transcriptome of *S. rebaudiana*. Accordingly, the transcriptome data obtained in this study provide a valuable resource for further research investigating of stevia, especially for SG biosynthesis. Moreover, a composite phylogenetic tree containing all SrUGTs was also constructed and the candidate *SrUGTs* involved in the glycosylation of steviol glucosides were analysized.

## Results

### Content of glucosides in samples

**Fig. 1:**
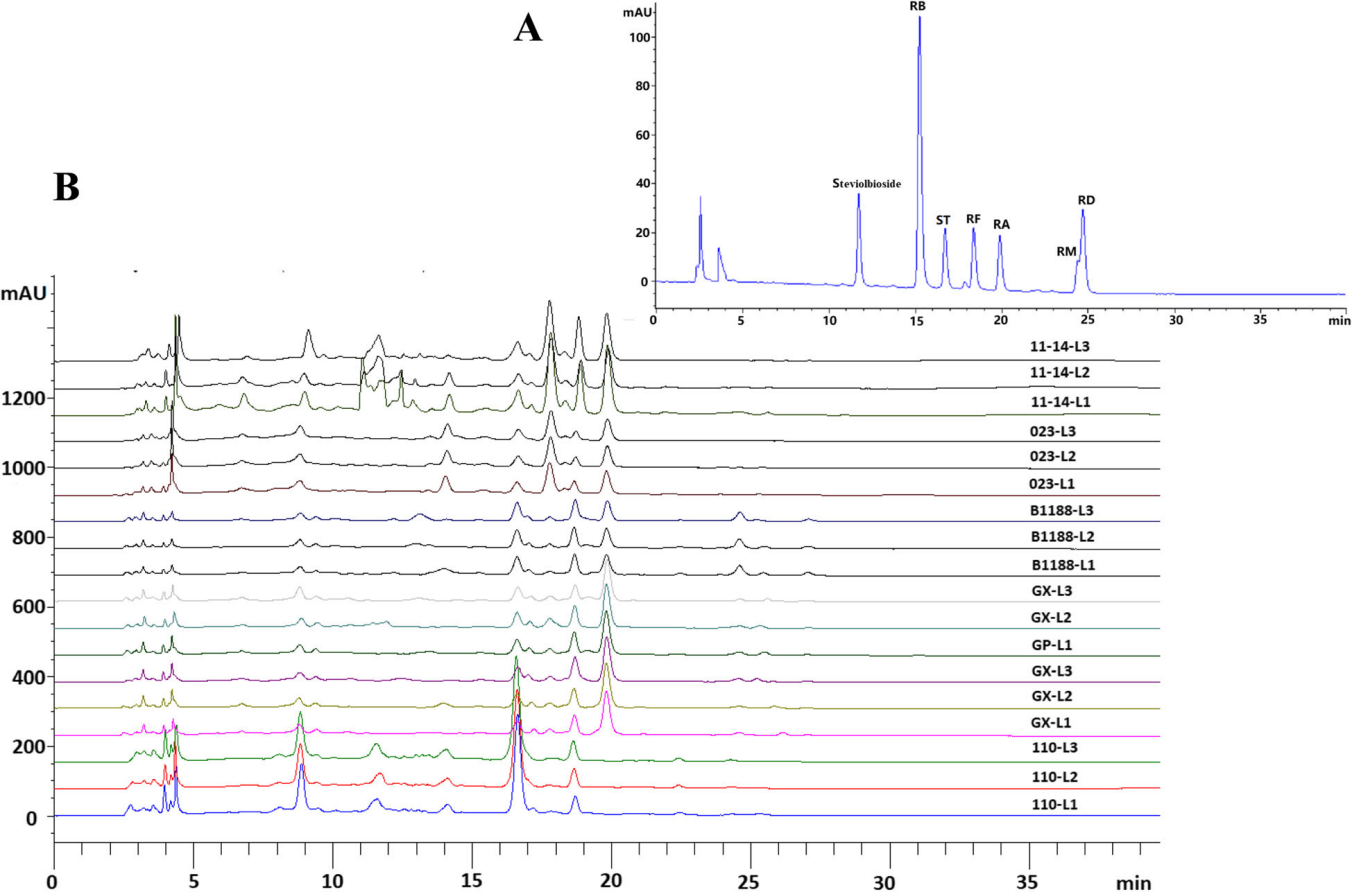
HPLC-UV profiles of the analytical samples and standards (steviolbioside, Reb B, ST, Reb F, Reb A, Reb D and Reb M). **a** HPLC-UV profiles of the seven standards. **b** HPLC-UV profiles of the analytical samples

All validation projects (Limit of detection (LOD), Limit of quantitation (LOQ), Calibration curve, Mean correlation coefficient Linear range, Accuracy, Injection precision, Stability, System suitability) used for detecting steviolbioside, rebaudioside B (Reb B), 1,2-stevioside (ST), rebaudioside F (Reb F), rebaudioside A (Reb A), rebaudioside D (Reb D) and rebaudioside M (Reb M) from HPLC-UV analysis satisfied the quantitative requirements (Table 1). The contents of steviol glucosides in the leaves of all detected samples are shown in Table 2, and the HPLC chromatograms are also shown in Fig. 1. The results demonstrated that all of the analytical glucosides in the experimental genotypes were clearly varied and provided a potential basis for WGCNA co-expression network analysis to uncover the glucosyltransferases involved in the biosynthesis pathway of the corresponding glycosides. Furthermore, data obtained separately from the leaves of the seedling, adult, and budding stages of the ‘023’ genotype also revealed that the accumulation of steviol glycosides in *S. rebaudiana* peaked in the budding period.

**Table 1:** Validation method parameters for quantification of seven steviol glycosides

Parameters	steviolbioside	Reb B	ST	Reb F	Reb A	Reb M	Reb D
LOD (μg/ml)	7.3	3.1	1.6	2.3	2.8	3.4	3.1
LOQ (μg/ml)	20.1	9.7	6.4	9.2	10.8	12.2	10.6
Calibration curve	y = 266.95x + 25.022	y = 219.28x + 1.9539	y = 222x + 2.7051	y = 170.87x-2.5236	y = 166.02x + 6.0046	y = 160.66x + 8.6147	y = 166.18x + 11.745
Mean correlation coefficient (R2)	0.9988	0.9989	0.9992	0.9991	0.9991	0.9995	0.9994
Linear range (μg/ml)	20.1–597.0	19.4–568.1	25.6–3610.4	33.7–586.0	21.7–4820.0	14.4–301.0	15.5–567.0
Accuracy (%, *n* = 3)	87.3	108.1	96.5	104.3	97.4	85.6	103.8
Injection precision (RSD%, *n* = 6)	1.97	1.94	1.53	2.03	1.72	1.58	2.34
Stability (RSD%)	1.51	1.73	1.21	1.93	1.05	1.46	2.01
System suitability (RSD%, *n* = 6)	1.92	1.85	1.74	2.13	1.59	1.95	1.87

**Table 2:** Contents of seven steviol glycosides in analytical samples

Samples	Content(%)						
	Steviolbioside	Reb B	ST	Reb F	Reb A	Reb M	Reb D
023-L1	0.53	0.36	1.98	1.76	8.80	-	0.36
023-L2	1.26	0.21	2.01	1.24	8.42	-	0.24
023-L3	0.97	0.12	1.55	0.98	7.74	0.05	0.06
110-L1	0.89	-	10.49	0.26	0.19	-	0.07
110-L2	0.99	-	9.44	0.29	-	-	0.09
110-L3	1.11	-	9.75	0.16	-	-	0.05
B1188-L1	-	0.43	4.13	0.21	5.77	0.14	3.17
B1188-L2	-	0.44	4.05	0.28	6.42	0.13	3.06
B1188-L3	-	0.29	3.93	0.33	5.76	0.12	3.06
GX-L1	-	0.44	3.27	0.54	14.46	-	0.95
GX-L2	-	0.37	3.13	0.45	14.08	-	0.71
GX-L3	-	0.47	3.19	0.46	13.09	0.05	1.03
11-14-L1	1.32	0.69	2.42	1.09	8.30	0.23	0.46
11-14-L2	1.61	0.60	1.94	0.87	6.37	0.16	0.35
11-14-L3	1.90	0.51	1.87	0.85	6.44	0.18	0.29
GP-L1	-	0.43	3.31	0.46	12.75	-	0.94
GP-L2	-	0.52	3.38	0.48	13.08	-	1.01
GP-L3	-	0.48	3.61	0.51	13.65	-	1.06

Note: “-” means no detection or lower than LOD

### Output from combining sequencing approach to the leaves of stevia

To obtain the complete full-length transcriptome of *S. rebaudiana* and identify the potential genes involved in the biosynthesis pathway of steviol glycosides, both NGS (ILLUMINA) and SMRT (PACBIO) sequencing platforms were combined to sequence six different stevia genotypes. First, eighteen high-quality cDNA libraries from six different genotypes (each in triplicate) were sequenced on the Illumina HiSeq X Ten platform, and 48,234,398 clean reads were generated after quality filtering (Table S1). Then, high-quality full-length cDNAs from the pooled RNA sample of the ‘023’ genotype were sequenced on the PacBio Sequel platform, and a total of 16,289,315 subreads (approximately 29.1 billion bases) were obtained. After we performed the IsoSeq protocols (https://github.com/PacificBiosciences/IsoSeq_SA3nUP/wiki#datapub), which included Circular Consensus Sequences (CCS), Classify and Cluster, a total of 39,879 consensus isoform sequences with considerably more accurate sequence information were obtained, and their average length was 1949 bp. To further correct the isoform sequences, all NGS clean reads were subsequently used to correct the 39,879 consensus isoform sequences using LoRDEC software (v0.7, parameter: -k 21 -T 20 -s 3 and others set to default) [19]. After removing the redundant sequences for all SMRT isoforms using CD-HIT (identity = 0.98) [20], 30,859 contigs (containing approximately 59.8 million bases) were produced with a mean length of 1938 bases. To validate the quality of 30,859 corrected contigs, firstly, we mapped the corrected contigs to the eukaryota_odb9 database and the result showed that there were 229 sequences on the perfect match (Figure S1). Secondly, mapping of the clean reads to the corrected contigs and resulting a high alignment rate of 85.42%.

**Fig. 2:**
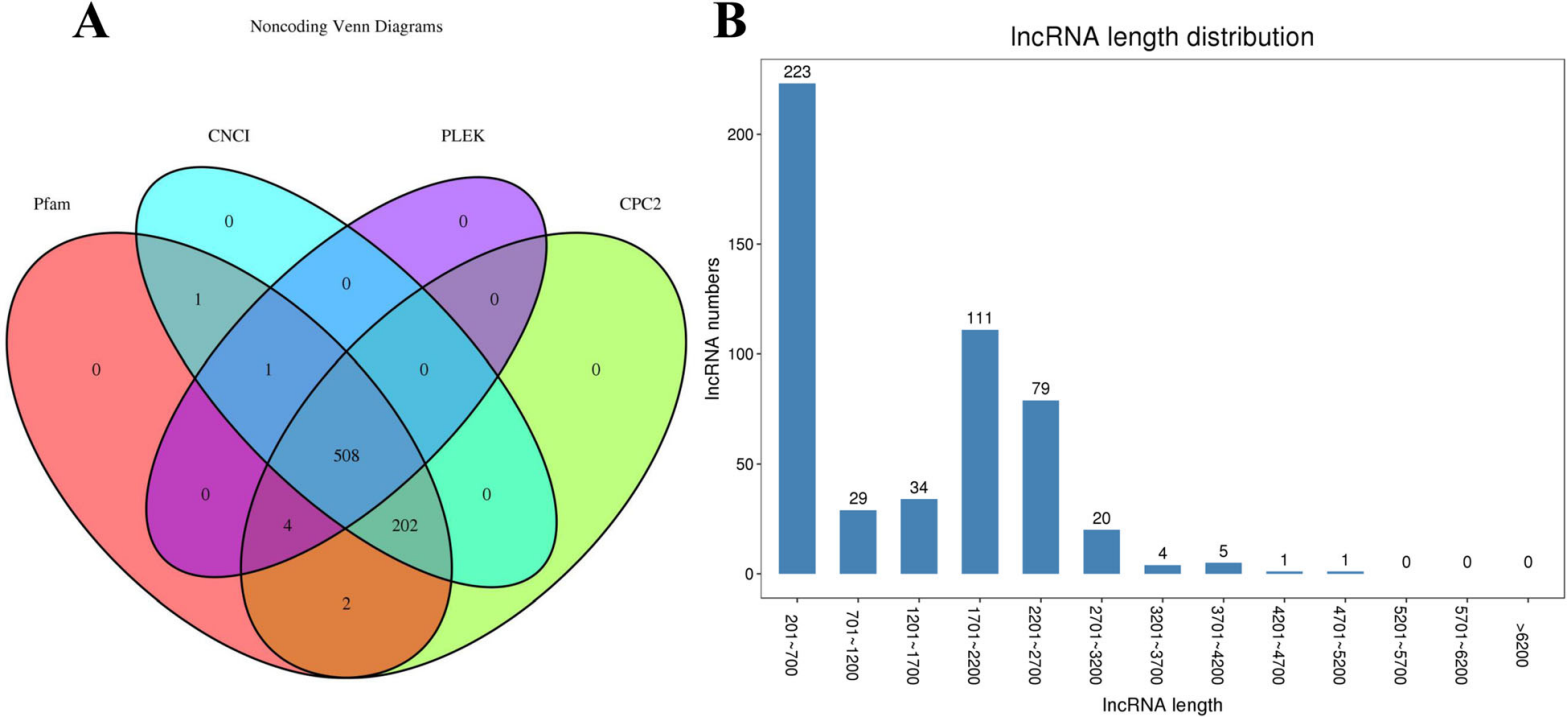
**a** Noncoding Venn diagrams; **b** LncRNA length distribution

In addition to contigs coding for proteins and the remaining contigs, homologous searches against the Coding Potential Calculator (CPC), Coding-Non-Coding Index (CNCI), Coding Potential Assessment Tool (CPAT) and Pfam reference protein databases predicted 508 of these contigs to be long non-coding RNAs (lncRNAs) with a mean length of 1321 bases (Fig. 2a, b). After SSR analysis, the number of sequences containing SSR was 1776 and without the compound SSRs; furthermore, the repeat numbers of the mononucleotides, dinucleotides, trinucleotides, tetranucleotides, pentanucleotides, and hexanucleotides were 4212, 2030, 3544, 94, 44, and 130, respectively.

**Fig. 3:**
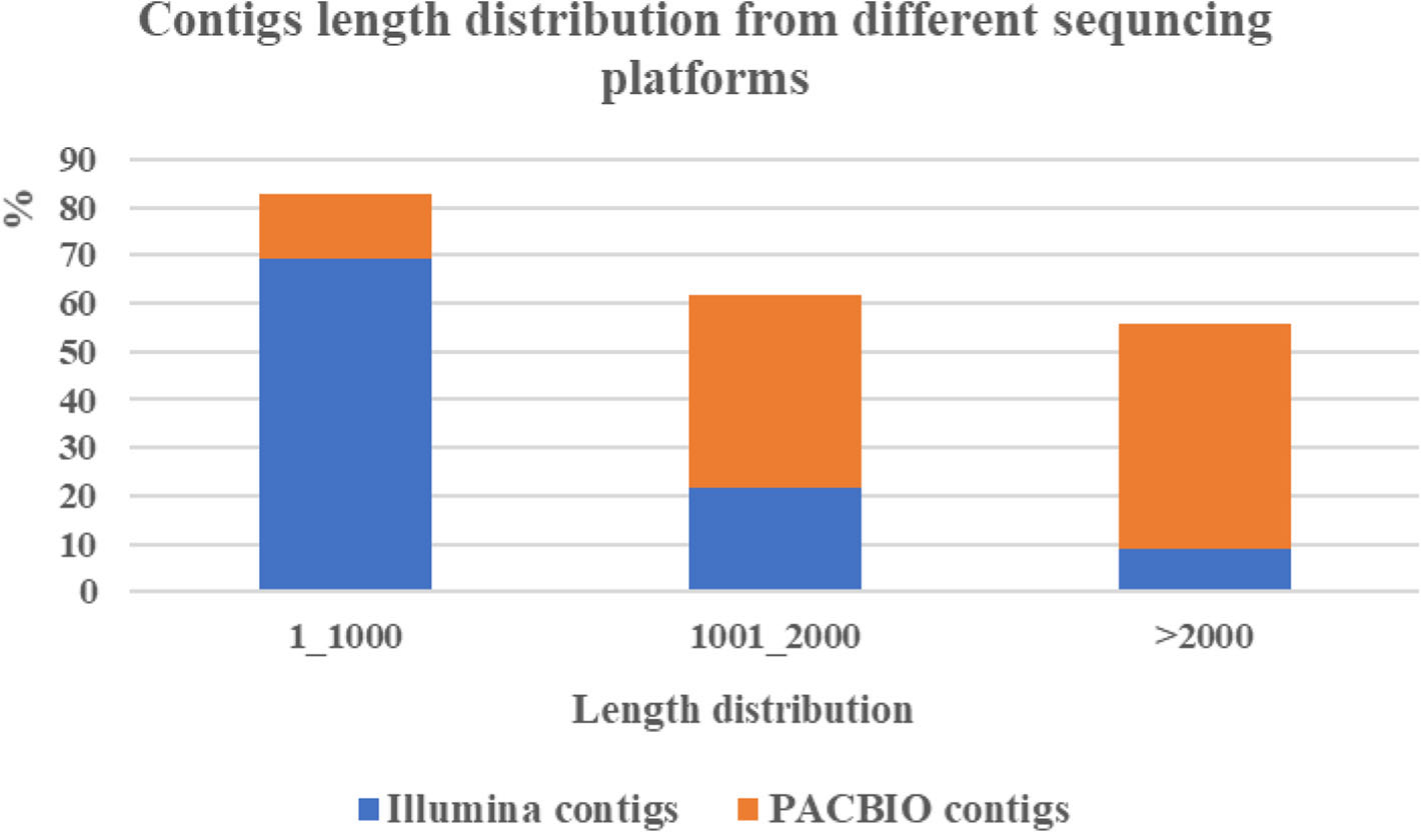
Comparison of contig length distribution sequencing on different platforms

According to the results from the comparison of transcript length distribution between Illumina and PacBio Sequel platforms, it was indicated that the transcripts assembled from the Illumina short reads by Trinity software [21] could not accurately represent full-length cDNAs in *S. rebaudiana*. The average length of assembled contigs from the Illumina platform (mean 905.8 bp) was notably shorter than those from the PacBio Sequel platform (mean 1949.2 bp). In addition, approximately 69.4% of the assembled contigs from NGS reads were < 1000 bp, whereas only 13.4% of the contigs from PACBIO reads were < 1000 bp (Fig. 3). Nevertheless, from this study, it seemed that the SMRT reads further corrected by the NGS data could be considerably better than simply relying on IsoSeq protocols.

### Phylogenetic analysis of the UDP-glycosyltransferase multigene family

**Fig. 4:**
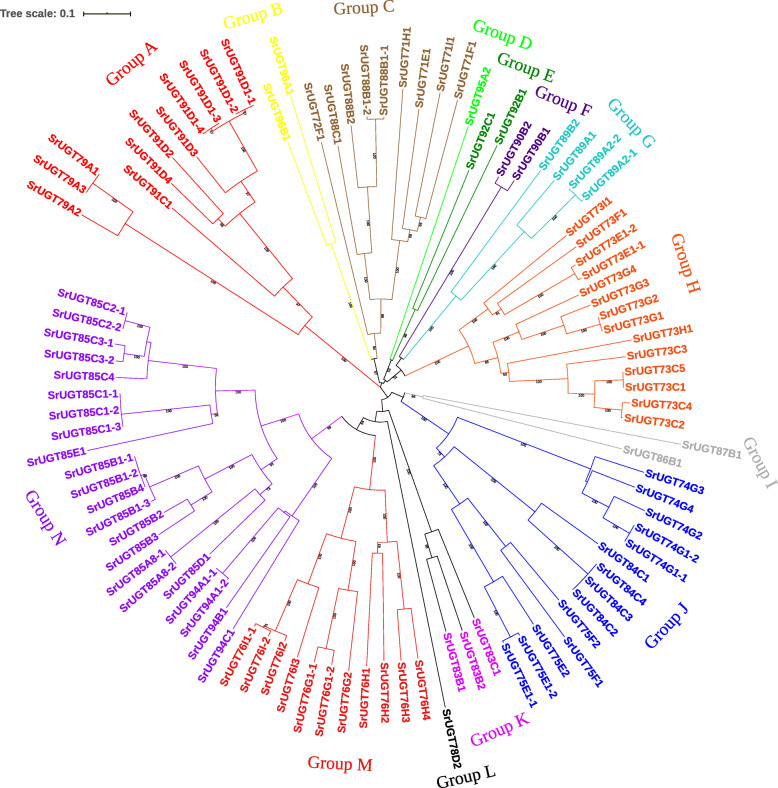
Phylogenetic analysis of the *S. rebaudiana* UGT superfamily shows 14 distinct groups, each with a bootstrap support greater than 90% in distance analysis excluding group I (66% bootstrap). The tree shown was derived by neighbour-joining distance analysis of the full-length amino acid sequence described in Additional file 6. Distance bootstrap analyses consisted of 1000 replicates. Bootstrap values are listed as percentages of the replications, where values over 50% are indicated above the nodes

UDP-glycosyltransferases (UGTs) are defined by the presence of a C-terminal consensus sequence containing 44 amino acids and are responsible for transferring a glycosyl moiety from an activated donor to an acceptor molecule in all living organisms [22, 23]. In *Arabidopsis thaliana*, a molecular phylogenetic tree was constructed consisting of ninety-nine UGT sequences and a composite phylogenetic tree that also includes all of the additional plant UGTs with known catalytic activities [22]. This work has significantly promoted the prediction of the evolutionary history, substrate specificities and structure-function relationships of UGTs in *Arabidopsis*. Nevertheless, although many studies have been performed on the glycosyltransferases of *S. rebaudiana*, there are still no reports on its phylogenetic tree. Therefore, a comprehensive neighbour-joining tree with ninety-eight complete SrUGTs was constructed (Fig. 4). After bootstrap analysis with 1000 replicates, the SrUGTs were strongly divided into fourteen major groups, with each having a support greater than 95% in distance analysis excluding group I (66% bootstrap). The fourteen well-defined major groups of SrUGTs suggest that at one time, there were fourteen ancestral genes. One sequence (SrUGT78D2) with a long unique terminal branch, suggesting accelerated evolutionary rates, tends to distort phylogenetic analyses by reducing apparent bootstrap support for nearby clades. Therefore, the data were reanalysed without this sequence. This analysis provided stronger statistical confidence (bootstrap from 64 to 88%) to two of the ancestral genes, corresponding to groups M and N, which are likely to share a more recent common origin. Interestingly, a similar *AtUGT78D1* gene has been found in *Arabidopsis* [22], indicating that the *UGT78D* genes in the glycosyltransferase family may have evolved more rapidly.

**Fig. 5:**
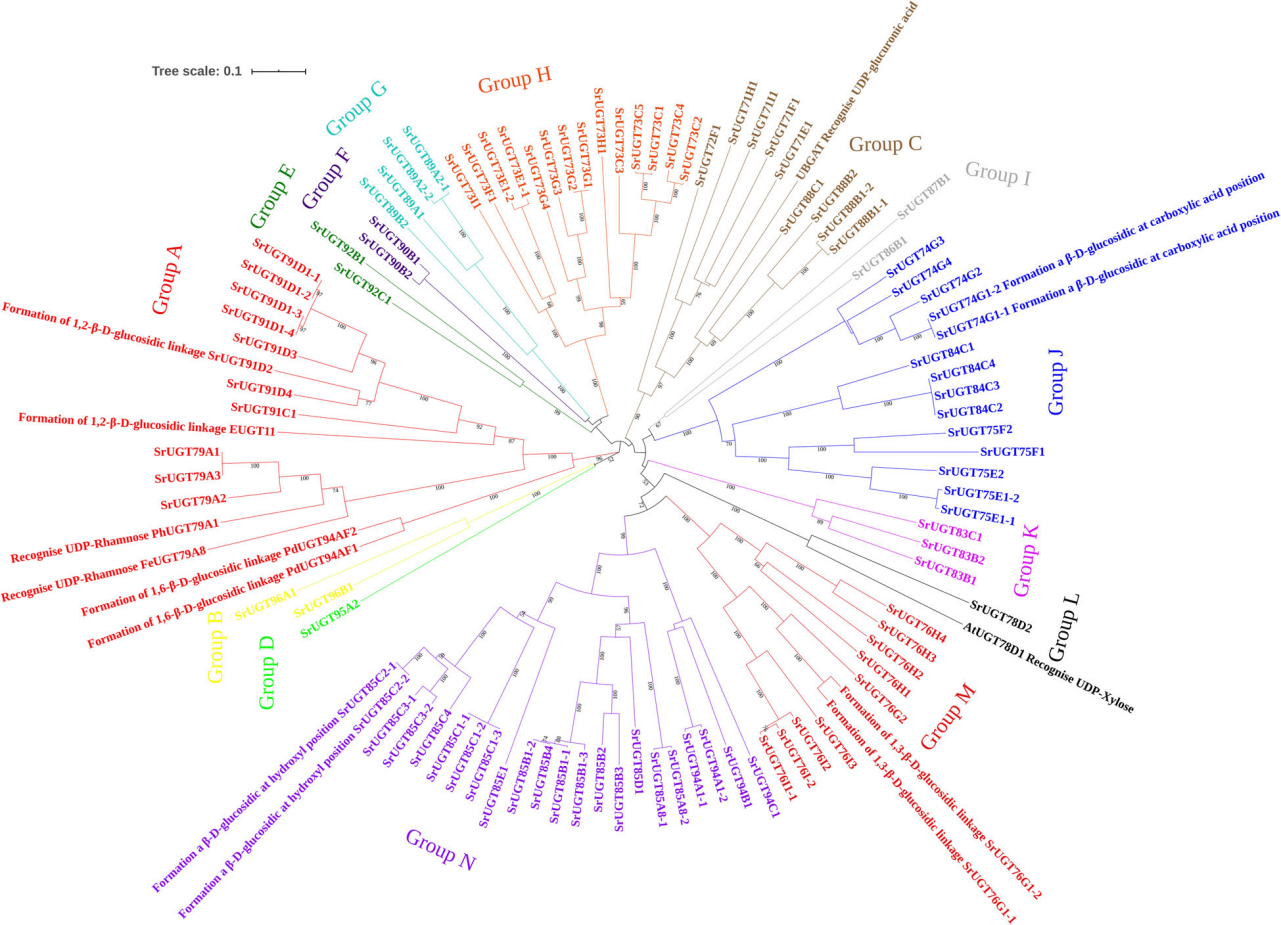
Composite phylogenetic tree. Seven UGTs from other plant species and four SrUGTs identified previously were also annotated in this tree. Distance bootstrap analyses consisted of 1000 replicates. Bootstrap values are listed as percentages of the replications, where values over 50% are indicated above the nodes

In an attempt to predict the structure-function relatedness of the SrUGT family, numerous UGTs identified from a wide range of plant species and having different biochemical functions were aligned with the ninety-eight SrUGTs and constructed a composite phylogenetic tree. Among these additional plant UGTs, seven of the corresponding UGTs were successfully clustered within the fourteen groups identified by this study (Fig. 5). Interestingly, PdUGT94AF1 and PdUGT94AF2 derived from *Prunus dulcis* involved in the formation 1,6-β-D-glucosidic linkage of Prunasin [24] were clustered in group A but had a long genetic distance between them and SrUGTs of this group, implying that the SrUGTs in this group may have no related specificity. Due to the lack of other glycosyltransferases capable of forming 1,6-glucosidic bonds, this tree cannot predict the glycosyltransferases involved in the synthesis of steviol glycosides containing 1,6-glucosidic bonds (such as Reb L). FeUGT79A8 and PhUGT79A1 identified from *Fagopyrum esculentum* and *Petunia x hybrida* could utilize UDP-rhamnose as sugar donors [25]; both UGTs belong to group A and are closely related to the SrUGT79 subfamily (100% bootstrap), demonstrating that the SrUGT79 subfamily may be the glycosyltransferases of UDP-rhamnose specificity in *S. rebaudiana*. In addition, EUGT11 from *Oryza sativa* and SrUGT91D2 are known to catalyse the 1,2-β-D-glucosidic linkage of steviol glycosides [7, 26]; therefore, it is reasonable to believe that the SrUGT91 subfamily is responsible for the formation of the 1,2-β-D-glucosidic linkage in stevia. UBGAT from *Scutellaria baicalensis* is one of the few glycosyltransferases identified in plants that could use UDP-GlcUA as the sugar donor to catalyse a glucuronosylation reaction [27]. In the composite tree, UBGAT was clustered in group C and had a closer relationship with the SrUGT88 subfamily; moreover, the similarity of the PSPG box between the UBGAT and SrUGT88 subfamily was more than 65%; therefore, we speculate that the SrUGT88 subfamily should be the UDP-glucuronic acid-recognizing glycosyltransferases in stevia. AtUGT78D1 identified from *Arabidopsis thaliana* could utilize UDP-xylose as a sugar donor [28]. In this tree, AtUGT78D1 and SrUGT78D2 are clustered in group L and have high bootstrap support (100%). Therefore, it is speculated that SrUGT78D2 should be a glycosyltransferase in stevia, which may recognize UDP-xylose and participate in the xylosylation of glycosides, such as RF. In 2005, Richman et al. (2005) identified and characterized three UGTs (SrUGT85C2, SrUGT74G1 and SrUGT76G1) involved in the synthesis of steviol glycosides: SrUGT85C2 and SrUGT74G1 glucosylate the *C*13-hydroxyl and *C*19-carboxylic acid functional groups of the steviol backbone, forming a β-D-glucoside, respectively, while SrUGT76G1 is capable of catalysing 1,3-β-D-glucosylation at both the *C*13 and *C*19 positions of steviol. Most SrUGTs in group N belong to the SrUGT85 subfamily and have a high support value; therefore, we hypothesized that the SrUT85C subfamily may be responsible for glucosylating the *C*13-hydroxyl position of the steviol backbone. Similar to the SrUGT85 subfamily, in group J, the SrUGT74G subfamily could theoretically be the glucosylated *C*19-carboxylic acid functional group of the steviol backbone. Furthermore, the SrUGT76G and SrUGT76I subfamilies not only have a high degree of support (100% bootstrap) but also have a close genetic distance, indicating that these two subfamilies may be responsible for the formation of the 1,3-β-D-glucosidic linkage.

### WGCNA co-expression network analysis for the investigation of steviol glycoside biosynthesis

**Fig. 6:**
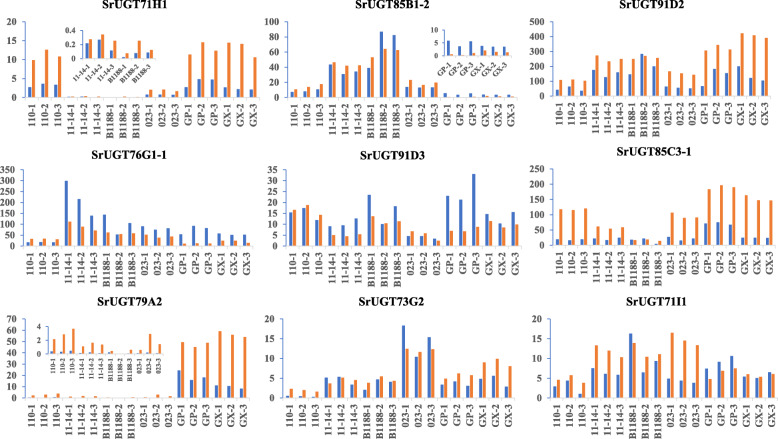
Comparison of RNA-seq and qRT-PCR data. RNA-seq data and qRT-PCR quantification of changes in nine selected differentially expressed *SrUGT* genes in all sequenced materials. Transcript expression of 26S rRNA was used as an internal control. Blue represents the qRT-PCR data, and red represents the RNA-Seq data. The results showed that the expression levels of these analysed genes were similar between the qRT-PCR and transcriptome data

**Fig. 7:**
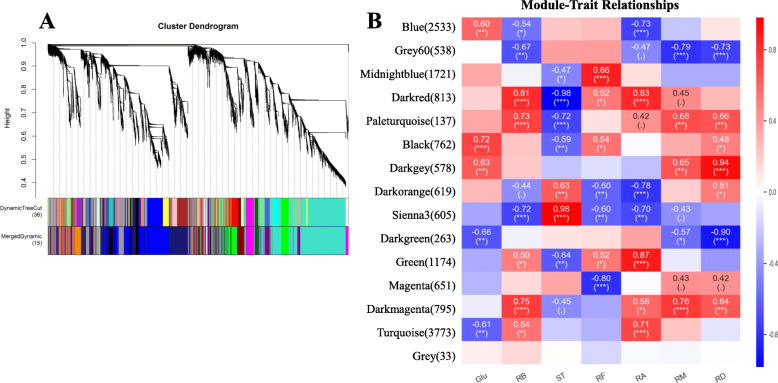
Network visualization plots. **a** Clustering dendrograms of genes with dissimilarity based on topological overlap together with assigned module colours. The same colour represents the same module, and the modules with certain correlations are merged into the same module. **b** Module-trait relationships. Each row corresponds to a module gene and column to a trait. Each cell contains the corresponding correlation and the significance. The table is colour-coded by correlation according to the colour legend

To date, many steps involved in the biosynthesis pathway of steviol glycosides have been successfully uncovered, especially for elucidating four UDP-dependent glucosyltransferases (UGTs) [6, 29], but several glucosylation steps of some glycosides that have not been resolved to date. In addition, for the large family of glucosyltransferases, we speculate that multiple enzymes with similar functions may participate in the same catalytic step in the glucosylation of steviol glycosides. Typically, the traditional method for differential expression analysis is constrained to paired sample analysis and thus unable to perform systematic analysis with large datasets from heterogeneous sources simultaneously [30, 31]. Therefore, in this study, one co-expression network approach named WGCNA, which was proved to be a powerful tool in systematically describing the correlation relationship between clusters of highly correlated genes or modules and external conditions or sample traits [31, 32], was used to analyse the potential *UGTs* involved in the glucosylation of steviol glycoside. First, we performed qRT-PCR analysis of the expression levels of nine *UGTs* (*SrUGT71H1*, *SrUGT85B1–2*, *SrUGT91D2*, *SrUGT76G1–1*, *SrUGT91D3*, *SrUGT85C3–1*, *SrUGT79A2*, *SrUGT73G2* and *SrUGT71I1*) in the leaves of six genotypes to confirm the reliability of the transcriptome data, and the primers used for qRT-PCR are shown in Table S2. The results showed that the tendency of these genes to be expressed was similar between the qRT-PCR and the transcriptomic data, confirming that the transcriptomic results were reliable (Fig. 6). And then all 71,718 transcripts assembled from NGS as input raw data for WGCNA co-expression network analysis. First, genes with low fluctuation expression (standard deviation < 1) were filtered, and 14,995 genes remained. When the power value of adjacency functions for weighted networks was 9, both the correlation coefficient and degree of gene connectivity could satisfy the requirement of scale-free network distribution to the greatest extent possible. Based on the selected power value, a weighted co-expression network model was established, and 14,995 genes were eventually divided into fifteen modules, of which the grey module had no reference significance because of the failure to assign to any module. The hierarchical clustering dendrogram of gene networks is visualized in Fig. 7a.

**Fig. 8:**
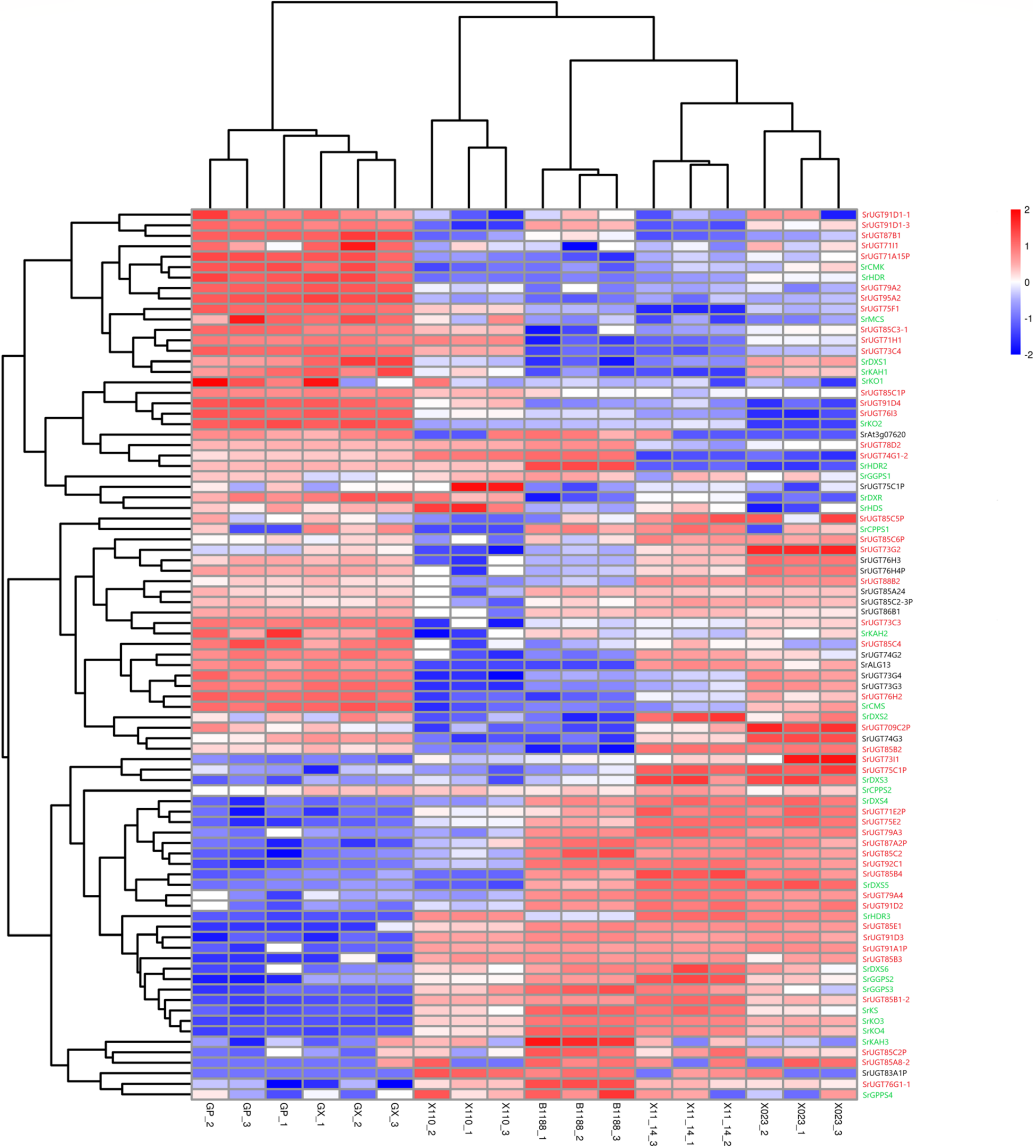
Heat map of the fifty-seven potential glucosyltransferase genes (in red and black) obtained from the weighted correlation network analysis and the genes (in green) known to be involved in steviol glycoside biosynthesis. Genes were clustered by expression patterns. Among the fifty-five glucosyltransferase genes, forty-four SrUGTs (in red) are co-expressed with at least one upstream gene, including SrDXS, SrDXR, SrCMS, SrCMK, SrMCS, SrHDS, SrHDR, SrGGPS, SrCPPS, SrKS, SrKO and SrKAH. Of these genes, those having the P letter at the end were found to contain a single nucleotide substitution, insertion, or deletion, thereby causing interruption of ORFs

To identify modules that are significantly associated with the traits of steviol glycoside content, fifteen generated modules were correlated with the traits. The modules related to each trait were screened according to the absolute value of correlation coefficient > 0.3 and *p*-value < 0.05. The colour-coded table in Fig. 7b shows the full module-trait relationships. For each trait-related module, the correlation between the gene expression profile and the corresponding traits (Gene Significance, GS) and the correlation between the gene expression profile and the module eigengenes were calculated. The results showed that the genes in the module are both highly correlated with the traits and the eigengenes. For example, the module eigengenes of turquoise (r = 0.71, correlation p-value = 0.00092) and dark-orange (r = − 0.78, correlation p-value = 0.00014) were significantly positively correlated or negatively correlated with RA, respectively. As a result, fourteen gene modules that are highly associated with steviol glycosides were identified. Among these genes in the fourteen modules, two genes belong to the acetylglucosaminyltransferase, fifty-five genes were annotated to be members of the plant *UGT* superfamily, including *SrUGT85C2*, *SrUGT74G1*, *SrUGT76G1*, *SrUGT85A8* and *SrUGT91D2,* which have already been reported to be involved in the glucosylation of steviol glycosides except for *SrUGT85A8* [6, 7], illustrating the reliability of our results. Furthermore, the expression levels of these genes in the leaves of the six genotypes are shown in Fig. 8.

In *S. rebaudiana*, steviol glycosides have been derived from the tetracyclic diterpene steviol backbone [6], and the precursors of steviol are actually synthesized via a series of enzymes consisting of DXS, DXR, CMS, CMK, MCS, HDS, HDR, GGDPS, CPPS, KS, KO and KAH [8, 33, 34]. Accordingly, the genes encoding enzymes involved in steviol biosynthesis might be expected to exhibit a similar co-expression pattern with the *SrUGTs* involved in the synthesis of steviol glycosides. Therefore, we further performed a similar co-expression analysis between the fifty-five *SrUGT*s and the genes involved in steviol biosynthesis. Notably, forty-four *SrUGT*s, including *SrUGT85C2*, *SrUGT74G1*, *SrUGT76G1* and *SrUGT91D2*, were then identified as being co-expressed with at least one of the upstream genes (Fig. 8), and it is reasonable to believe that these *SrUGTs* may be directly involved in the synthesis of the corresponding steviol glycosides, which warrants further research.

To confirm the sequence accuracy of the candidate *SrUGTs*, twenty of these forty-four candidate *SrUGTs* (randomly selection) were cloned by reverse transcriptase polymerase chain reaction (RT-PCR) using the designed primers (Table S3), and then sequenced with the pClone007 Blunt vector (TsingKe, China). After analysis the nucleotide similarity between the cloned and the transcriptome, it was found that there were three base substitutions in the *SrUGT91D1–1* sequence, twelve base substitutions in the *SrUGT85B4* sequence, one base substitutions in the *SrUGT73G2* sequence, eight base substitutions in the *SrUGT85C3–1* sequence, eleven base substitutions in the *SrUGT95A2* sequence and no base difference in the remaining fifteen *SrUGTs* sequence (Additional file 7). Because of the self-incompatible in stevia, the sequence difference should be due to genetic heterozygosity [9, 10]. Moreover, the forty-four candidate glycosyltransferase genes were aligned with the *S. rebaudiana* genome (GenBank: WOUH00000000.1), and found that these genes were located in different positions in the *S. rebaudiana* genome, except *SrUGT91D1–3* & *SrUGT91D1–1* (Table S4). These results demonstrated the reliability of our transcriptome sequences and the analysis were performed on distinct genes.

## Discussion

To reduce the intake of sucrose and other high-energy sweeteners, the demand for natural non-caloric sweeteners is increasing. Steviol glycosides from the leaves of stevia constitute such a natural alternative. To this end, several previous transcriptome studies have focused on steviol glycosides [6, 7, 11–13]; however, it was still generally requiring further cloning efforts to obtain the full-length cDNA sequence for the investigation of target genes in SG biosynthesis. The taste of steviol glycosides relies on glycosylation at the C13-hydroxyl and/or C19-carboxylic acid positions of the diterpenoid steviol backbone; generally, perceived sweetness is positively correlated with the total number of glucose residues present [35]. Therefore, current research primarily focuses on the discovery and regulation of glycosyltransferases, the investigation of which largely depends on synthetic biology approaches using genes codon-optimized for recombinant expression [6, 29], which clearly demands accurate and full-length cDNAs. In this study, we combined short-read NGS and long-read SMRT sequencing of six different genotypes of stevia, which are the largest number of materials used for sequencing at all times, and then successfully generated a full-length transcriptome of the stevia leaf (total of contigs = 39,879, average length = 1949 bp). After correcting the SMRT reads using ILLUMINA reads, we finally obtained more high-quality full-length transcripts (total of contigs = 30,859, average length = 1938 bp, contigs (length ≥ 1000 bp) more than 85%), reducing misassembly of genes and gene families with high sequence identity. According to the transcriptome of *S. rebaudiana* sequenced on Illumina platform reported by Chen et al. in 2014, 80,160 contigs were obtained by de novo assembly, with an average length of 969 bp and length of more than 65% contigs ≤1000 bp [12]. Similarly, in the transcriptome of *S. rebaudiana* reported by Singh et al. in 2017, 41,262 contigs were obtained by de novo splicing, with an average length of 922 bp and length of more than 67% contigs ≤1000 bp [14]. These reflect that the contigs in our corrected full-length transcriptome were better than those of the assembled transcripts. To the best of our knowledge, this report presents the first public data to characterize the structure of transcripts in *S. rebaudiana*. Moreover, it should be noted that the *S. rebaudiana* genome (GenBank: WOUH00000000.1) was published on January 28, 2020 and available in the public databases, but the genome with no annotation file and the sequencing work of this study was finished on January 7, 2019. Therefore, we used a de novo transcriptome assembly approach to reconstruct the transcript in NGS sequencing. With the publication of genomic data of *S. rebaudiana* (GenBank: WOUH00000000.1), our transcriptome data will provide strong support for the study of alternative splicing (AS) forms and alternative polyadenylation (APA) events of genes in stevia.

In previous studies, the number of *S. rebaudiana* samples used for sequencing was usually nine (three genotypes with three repetitions), which led to the restriction of analysis methods to paired sample analysis using differential expression analysis [7, 12, 13]. For the large family of glycosyltransferases, it is difficult to systematically reveal their role in SG synthesis. After more than a decade of study, we screened six genotypes of *S. rebaudiana* with significantly different concentrations or types of steviol glycosides from hundreds of wild-type and mutant plants. Based on this research, we can systematically analyse the transcriptome data of eighteen *S. rebaudiana* materials using the WGCNA method and then systematically reveal the correlation relationship between *SrUGTs* and stevioside biosynthesis in stevia for the first time. Among the forty-four candidate SrUGTs, including the four identified SrUGTs (SrUGT85C2, SrUGT74G1, SrUGT76G1 and SrUGT91D2)*,* therefore, it is reasonable to believe that these forty-four enzymes are mainly involved in the glycosylation of steviol glucosides. Due to the lack of UDP-Rha and UDP-Xyl, the SrUGTs using UDP-Rha or UDP-Xyl as sugar donors in the stevia were not successfully identified, in this work, we provided a high-quality analysis in the composite phylogenetic tree and obtained potential candidate *SrUGTs* with full-length sequences. Our study provides a template for investigating secondary metabolism in other species, paving the way for synthetic biology approaches to such natural products. Moreover, some of these six genotype stevias could be ideal materials for industrial production and could be directly used to extract high-purity steviol glycosides.

## Conclusion

In summary, the full-length transcriptome data of *S. rebaudiana* were generated by a combination of Illumina and SMRT sequencing platforms. We systematically revealed the glycosyltransferase in *S. rebaudiana* and finally forty-four candidate *SrUGTs* involved in the glycosylation of steviol glucosides were obtained using WGCNA, phylogenetic trees, and qRT-PCR methods. The present study may serve as a valuable resource for future *S. rebaudiana* studies and may also benefit investigations involving other closely related species. The full-length transcriptome dataset may also provide useful candidate genes for the elucidation of the mechanism of steviol glucosides biosynthesis.

## Methods

### Plant materials and RNA sample preparation

Six *S. rebaudiana* genotypes (named 110, 11–14, 023, GX, GP, B1188) with different concentrations of the steviol glycosides were harvested from an experimental field of Sichuan Agricultural University (Chengdu, Sichuan Province in China). The genotypes of 11–14 and B1188 were obtained from Anhui Province and Shandong Province, respectively. The remaining genotypes were obtained by the induction through mutagenic methods as follows: 023 and 110 were obtained from induction by ^60^Co γ-ray irradiation of the callus of 11–14 leaf tissue; GX was induced from the tissue culture seedling of 11–14 in medium with high concentration of zinc; GP was induced from the tissue culture seedling of 11–14 in medium with high concentration of boron. Eighteen independent samples (six different genotypes of leaf tissues with three repetitions) of the 3rd leaf tissues in the budding period (the accumulation of steviol glycosides peaked) were collected. Genotype ‘023’, which had a wide variety of steviol glycosides, was used for the full-length transcriptome sequence. To obtain sufficient full-length transcriptome sequences, samples of the 3rd leaf tissues of the ‘023’ genotype were separately collected from the seedling and adult stages. All samples were identified by Professor Wei Wu, who studied *stevia* for more than 10 years, and all genotypes used in this study were planted at Sichuan Agricultural University. Material collection was conducted in accordance with local legislation, and there was no need for permission from other organizations. We complied with the Convention on the Trade in Endangered Species of Wild Fauna and Flora.

A total of twenty RNA samples were isolated using the mirVana miRNA Isolation Kit (Ambion) following the protocol; these samples were from the ‘110’ (budding period), ‘11–14’ (budding period), ‘023’ (budding period), ‘GX’ (budding period), ‘GP’ (budding period), ‘B1188’ (budding period), ‘023’ (seedling period), and ‘023’ (adult period). The enrichment of Poly(A) RNA from the total RNA was carried out by the oligo d(T) magnetic bead binding method. The total RNA was quantified, and the quality was assessed using the Agilent 2100 Bioanalyzer (Agilent Technologies, Santa Clara, CA, USA) and NanoDrop (Thermo Fisher Scientific, USA). The samples with RNA integrity number (RIN) ≥ 7 were subjected to subsequent analysis. Finally, equal amounts of ‘023’ (seedling period), ‘023’ (adult period) and ‘023’ (budding period) were combined to provide the total *S. rebaudiana* RNA and then subjected to Pacific Biosciences (PacBio) single-molecule long-read sequencing (Pacific Bioscience, Menlo Park, USA). Eighteen samples of the six different genotypes were submitted for second-generation transcriptome sequencing using the Illumina HiSeq X Ten platform (Illumina, USA).

In *S. rebaudiana*, more than thirty-five steviol glycosides have been successfully identified [4, 5]. Of these glycosides, the standards of steviolbioside, Reb B, ST, Reb F, Reb A, Reb D and Reb M were easy to purchase; furthermore, their concentrations were relatively higher than those of other glycosides. Therefore, analysis of the eight steviol glycosides in all samples was conducted by reference to the HPLC-UV method in [36]. In addition, a calibration curve, limit of detection (LOD), limit of quantification (LOQ), system suitability, precision and accuracy parameters were used to validate the method. The LOD and LOQ were calculated using both the values of the calibration curve and signal-to-noise ratios of 10 and 3, respectively [37].

### Library preparation and Illumina short-read sequencing

The libraries were constructed using the TruSeq Stranded mRNA LTSample Prep Kit (Illumina, San Diego, CA, USA) according to the manufacturer’s instructions. First, fragmented mRNAs were generated by adding the interruption reagent. Second, first-strand cDNA was synthesized by SuperScript II Reverse Transcriptase using random primer 6. Next, second-strand cDNA synthesis was performed using Phusion High-Fidelity DNA Polymerase. Purified cDNA was normalized by end repair, adenylation of the 3ʹ ends and ligation of the adapters. Finally, the normalized cDNA was amplified using the PCR method to enrich cDNA fragments, and the amplified cDNAs were purified by the AMPure XP system (Beckman Coulter, Beverly, USA). After purification, the PCR products were validated on an Agilent 2100 Bioanalyzer (Agilent Technologies), and the sizes were also checked by agarose gel electrophoresis. Then, these libraries were sequenced on the Illumina sequencing Illumina HiSeq X Ten platform (Illumina), and 150-bp paired-end reads were generated. De novo transcriptome assembly was accomplished using Trinity software [21] with min_kmer_cov set to 2 and all other parameters set to default. The assembled sequences were clustered using CD-HIT (with default parameters) [20] to generate contigs.

### Library preparation and PACBIO sequencing

The library construction and PacBio sequencing were performed according to the official protocol as described by Pacific Biosciences (Pacific Biosciences, USA). Briefly, 1 μg of total RNA was used as input for first-strand cDNA synthesis using a SMARTer PCR cDNA Synthesis kit (Clontech, USA). The first-strand products were diluted to an appropriate volume and subsequently used for large-scale PCR. Next, a total of 12 PCR cycles of amplification were performed for second-strand cDNA synthesis using PrimeSTAR GXL DNA Polymerase (Clontech, USA). After amplification, the PCR products were purified with AMPure PB Beads (Pacific Biosciences) and then normalized by repairing DNA damage, repairing ends and blunt ligation reactions. The normalized cDNA products were then subjected to the construction of SMRTbell template libraries using the SMRTbell Template Prep Kit 1.0 (Pacific Biosciences). Finally, two SMRT cells were sequenced on a PacBio Sequel instrument using sequencing kit 2.1 (Pacific Biosciences) with 10 h movie recordings.

### Isoform analysis

Subreads were subjected to circular consensus sequences (CCS) using SMRT analysis software (https://www.pacb.com/products-and-services/analytical-software/devnet/), and then full-length (FL) transcripts with a correction accuracy greater than 99% (high-quality isoforms) were obtained. Pac PacBio reads were classified into full-length (FL) and non-full-length reads, and then reads were corrected with the data generated with Illumina HiSeq X Ten using LoRDEC. The corrected isoforms were clustered using CD-HIT (identity = 98%) to generate contigs [38]. To validate the quality of the contigs, we used two different approaches. Firstly, the clean reads obtained from each genotype by NGS were mapped on the contigs using Bowtie2 tool, and secondly, mapping of contigs to the eukaryota_odb9 database (including a total of 100 species with 303 sequences) using BUSCO tool (V3.0.1, parameter: -m tran and others set to default) [39] to assess the completeness of contigs.

### Functional annotation of contigs

The contig sequences were selected to map with the eight databases using diamond software (Crystal Impact GbR, Germany) and HMMER software (www.hmmer.org), and obtained the annotation information of the contigs with e-values <1e^− 5^ against the eight databases, including non-redundant protein sequence database (NR; https://www.ncbi.nlm.nih.gov/), Clusters of Orthologous Groups of proteins/euKaryotic Ortholog Groups (COG/KOG; ftp://ftp.ncbi.nih.gov/pub/COG/KOG/kyva), Gene Ontology (GO; http://www.geneontology.org/), non-redundant protein sequence database (Swiss-Prot; http://www.uniprot.org/), evolutionary genealogy of genes: Nonsupervised Orthologous Groups (eggNOG; http://eggnog.embl.de/), Kyoto Encyclopedia of Genes and Genomes (KEGG; http://www.genome.jp/kegg/pathway.html), and the database of Homologous protein family (Pfam; http://pfam.xfam.org/).

### LncRNA prediction

LncRNAs are a kind of RNA molecule measuring over 200 bp and having no coding ability. In this study, contig sequences were used to predict the potential lncRNAs and followed these steps: first, transcripts with length upper than 200 bp were screened out and then removing the transcripts already annotated on the coding libraries; finally, the coding ability of screened transcripts was predicted using CPC, CNCI, CPAT and Pfam (v1.5) protein structure domain analysis.

### Simple sequence repeat (SSR) analysis

Simple sequence repeat (SSR), from DNA sliding and mismatch during DNA replication and repair or the unequal exchange of sister chromatids in mitosis and meiosis, is a tract of DNA (ranging in length from 2 to 13 base pairs) with certain motifs repeated 5–50 times [40]. Contig sequences were selected for SSR analysis using MIcroSAtellite software (MISA, v1.0). After analysis, a total of six SSR types can be detected, including mononucleotide, dinucleotide, trinucleotide, tetranucleotide, pentanucleotide, and hexanucleotide SSRs. In addition, the repeat times were divided into 5, 6, 7, 8, 9, 10, 11, and > 11 times.

### Differential expression analysis

In this study, reads from eighteen samples of six different genotypes (110, 11–14, 023, GX, GP and B1188) were produced. The expression analysis from ILLUMINA reads of different samples was carried out with bowtie2 software (v3.3.3.1, parameter: --reorder -k30 -t and others set to default) (obtaining the reads on contigs for each sample) and eXpress software (v1.5.1, parameter: --rf-stranded and others set to default) (calculating the FPKM value of expression). The bam files, which used for eXpress analysis, were generated by comparing the reads to transcripts using bowtie2 software.

### qRT-PCR analysis

To verify the reliability of the transcriptional data, qRT-PCR experiments were carried out with ten *UGT* genes. Eighteen RNA samples were extracted separately from six different genotypes (110, 11–14, 023, GX, GP and B1188, one genotype with three repetitions), and the leaves used for RNA isolation were the same as those for sequencing. Reverse transcription was performed with a HiScript® II Q RT SuperMix for qPCR (+gDNA wiper) Kit (Vazyme, China). qRT-PCR primers were designed from the specific domain of target genes using PRIMER PREMIER 6 (PREMIER Biosoft, Canada), and their specificity was checked by PCR. qRT-PCR amplification was carried out in triplicate using 2 × T5 Fast qPCR Mix (SYBR Green I) (TSINGKE), with 26S rRNA as the reference gene, by a 7500 real-time PCR system (ABI).

### Phylogenetic analysis of *UGTs*

To maximize the transcript information of *UGT* genes, we combined all the *UGT* genes obtained from Pac PacBio and the ILLUMINA sequencing. Then, those containing a single nucleotide substitution, insertion, or deletion were excluded, causing interruption of ORFs. Moreover, numerous UGTs identified from a wide range of plant species with different biochemical functions were also selected from the National Center for Biotechnology Information (NCBI) and then pooled with the abovementioned UGTs before performing an alignment using MEGA-X (MEGA, http://www.megasoftware.net/) to construct another composite tree. Furthermore, the alignment was then reconciled and further optimized to minimize insertion/deletion events. An unrooted phylogenetic tree was then constructed by the neighbour-joining clustering method with the amino acid sequences of the ORFs using the bootstrap method with 1000 replicates. Guided by the system established by the GT Nomenclature Committee [41] and further combining the classification of *UGTs* in *Arabidopsis* and the named *UGTs* in *S. rebaudiana*, we systematically classified and named *UGTs* from *S. rebaudiana*.

### WGCNA co-expression network analysis

Due to the highly multivariate and complex RNA-Seq data, our first application of WGCNA to stevia transcriptome datasets was performed to reveal possible transcript modules associated with the biosynthesis of steviol glycosides, especially for *UGT* genes that directly catalyse their formation. The R package WGCNA was used to complete statistical analysis. Unsigned, weighted correlation networks were constructed by R package WGCNA with the default power of nine. In addition, network visualization was performed using R language and Python.

## Supplementary Information

**Additional file 1: Table S1.** Summary of transcriptome data sequenced by the Illumina platform and their pre-treatment.

**Additional file 2: Table S2.** Primers and annealing length in qRT-PCR.

**Additional file 3: Figure S1.** BUSCO assessment of the 30,859 corrected contigs.

**Additional file 4: Table S3.** Primers and annealing temperature of the twenty *SrUGTs*.

**Additional file 5: Table S4.** Position in *S. rebaudiana* genome of the forty-four candidate *SrUGTs*.

**Additional file 6.** Supplementary information, nucleotide and protein sequences used for phylogenetic tree in this study.

**Additional file 7.**The nucleotide sequences of the five *SrUGTs*, which cloned and differed with transcriptome in this study.

## Data Availability

All data sets supporting the conclusions of this study are included within the article (and additional files). The raw data from the Illumina HiSeq X Ten platform have been submitted to the Sequence Read Archive (SRA) of the NCBI under accession numbers SRR10799213, SRR10799212, SRR10799203, SRR10799202, SRR10799201, SRR10799200, SRR10799199, SRR10799198, SRR10799197, SRR10799196, SRR10799211, SRR10799210, SRR10799209, SRR10799208, SRR10799207, SRR10799206, SRR10799205 and SRR10799204. The accession number of the raw data from SMRT sequencing was SRR10567116. The assembled and the corrected transcripts were submitted to the Transcriptome Shotgun Assembly (TSA) of the NCBI under the accession numbers GISI00000000 and GISQ00000000, respectively.

## Abbreviations

SG: Steviol glycoside
GTs: Glycosyltransferases
UGT: UDP-glycosyltransferase
SMRT: Single-molecule real-time
NGS: Next-generation sequencing;
SrUGTs: Glycosyltransferases of *Stevia rebaudiana*
HPLC: High performance liquid chromatography
WGCNA: Weighted gene co-expression network analysis
ESTs: Expressed sequence tags
Reb A: Rebaudioside A
Reb B: Rebaudioside B
Reb D: Rebaudioside D
Reb F: Rebaudioside F
Reb M: Rebaudioside M
ST: 1,2-stevioside
CCS: Circular Consensus Sequences
CPC: Coding Potential Calculator
CNCI: Coding-Non-Coding Index
CPAT: Coding Potential Assessment Tool;
lncRNAs: Long non-coding RNAs
qRT-PCR: Quantitative Real-time PCR
DXS: Deoxyxyulose-5-phosphate synthase
DXR: Deoxyxyulose-5-phosphate reductoisomerase
CMS: 4-diphosphocytidyl-2-C-methyl-D-erythritol synthase; CMK4-diphosphocytidyl-2-C-methyl-D-erythritol kinase
MCS: 4-diphosphocytidyl-2-C-methyl-D-erythritol 2,4-cyclodiphosphate synthase
HDS: 1-hydroxy-2-methyl-2(E)-butenyl 4-diphosphate synthase
HDR: 1-hydroxy-2-methyl-2(E)-butenyl 4-diphosphate reductase
GGDPS: Geranylgeranyl diphosphate synthase
CPPS: Copalyl pyrophosphate synthase
KS: ent-Kaurene synthase
KO: Kaurene oxidase
KAH: Kaurenoic acid 13-hydroxylase
AS: Alternative splicing
APA: Alternative polyadenylation
UDPG: Uridine diphosphate glucose
PCR: Polymerase chain reaction
