# Supplementary material for: Characterizing glycosyltransferases by a combination of sequencing platforms applied to the leaf tissues of Stevia rebaudiana
Source: BMC Genomics. 2020 Nov 13;21:794. doi: 10.1186/s12864-020-07195-5 (PMC7664074; doi:10.1186/s12864-020-07195-5)
Supplement: Supplementary file 1 — Additional file 1: Table S1. Summary of transcriptome data sequenced by the Illumina platform and their pre-treatment. [file 12864_2020_7195_MOESM1_ESM.docx]

Additional file 1

Table S1. Summary of transcriptome data sequenced by Illumina platform and their pretreatment.

| **Sample** | **raw_reads** | **raw_bases** | **clean_reads** | **clean_bases** | **valid_bases** | **Q30** | **GC** |  |
| --- | --- | --- | --- | --- | --- | --- | --- | --- |
| 023_1 | 49843922 | 7476588300 | 47876648 | 6806329140 | 91.04% | 93.94% | 44.13% |  |
| 023_2 | 48996426 | 7349463900 | 46919448 | 6675057794 | 90.82% | 93.54% | 44.33% |  |
| 023_3 | 49401742 | 7410261300 | 47552518 | 6751209684 | 91.11% | 93.98% | 44.19% |  |
| 110_1 | 49722014 | 7458302100 | 45926926 | 6458642249 | 86.60% | 92.16% | 44.84% |  |
| 110_2 | 49404910 | 7410736500 | 46582200 | 6502944162 | 87.75% | 93.25% | 44.73% |  |
| 110_3 | 49362384 | 7404357600 | 44945204 | 6356051092 | 85.84% | 92.12% | 44.61% |  |
| 11_14_1 | 49653564 | 7448034600 | 47307950 | 6692486455 | 89.86% | 94.15% | 44.49% |  |
| 11_14_2 | 49231842 | 7384776300 | 47763764 | 6812825495 | 92.26% | 95.13% | 44.12% |  |
| 11_14_3 | 49166446 | 7374966900 | 47782594 | 6806876800 | 92.30% | 95.34% | 44.40% |  |
| B1188_1 | 49377538 | 7406630700 | 48234398 | 6924315420 | 93.49% | 95.53% | 44.34% |  |
| B1188_2 | 49777320 | 7466598000 | 47851898 | 6854222317 | 91.80% | 94.71% | 44.16% |  |
| B1188_3 | 49228444 | 7384266600 | 47198328 | 6662790571 | 90.23% | 94.63% | 44.62% |  |
| GP_1 | 49241838 | 7386275700 | 47895052 | 6864522293 | 92.94% | 95.16% | 44.29% |  |
| GP_2 | 49628110 | 7444216500 | 47971050 | 6854301787 | 92.08% | 94.87% | 43.80% |  |
| GP_3 | 49265404 | 7389810600 | 46875058 | 6605777227 | 89.39% | 93.95% | 43.79% |  |
| GX_1 | 49252564 | 7387884600 | 46469388 | 6528722426 | 88.37% | 93.80% | 44.07% |  |
| GX_2 | 49786730 | 7468009500 | 46838488 | 6667098353 | 89.28% | 93.56% | 44.36% |  |

Note: (1) raw_reads: the number of original reads; (2) raw_bases: the amount of original sequencing, i.e. the base number; (3) clean_reads: the number of clean reads after filtration; (4) clean_bases: the amount of sequencing after filtration, i.e. the base number; (5) valid_base: the percentage of effective bases; (6) Q30: the percentage of the base with the Phred value greater than 30; (7) GC: the percentage of the total number of G and C bases in clean bases.
