# Supplementary material for: Characterizing glycosyltransferases by a combination of sequencing platforms applied to the leaf tissues of Stevia rebaudiana
Source: BMC Genomics. 2020 Nov 13;21:794. doi: 10.1186/s12864-020-07195-5 (PMC7664074; doi:10.1186/s12864-020-07195-5)
Supplement: Supplementary file 2 — Additional file 2: Table S2. Primers and annealing length in qRT-PCR. [file 12864_2020_7195_MOESM2_ESM.docx]

Additional file 2

Table S2. Primers and annealing length in qRT-PCR.

| ***SrUGT* name** | **F-primer(5′-3′)** | **R-primer(5′-3′)** | **length(bp)** |
| --- | --- | --- | --- |
| *SrUGT71H1* | ACCACCATCTCCGTCGTATCT | GATGCGTGCGTTGTATTGATT | 165 |
| *SrUGT85B1-2* | CGCAAAGAGCAACTCGCAAAGAGCAACTATCCGTT | ACACCATCCAACCACCATCC | 125 |
| *SrUGT91D2* | TCCATAGTTGACGACCGTAAGC | ATGAGTGGCGAGATATGAGAAGAG | 176 |
| *SrUGT76G1* | TCTTAACCTCCGACGGCTTG | CCCACTCGCTTGTTCTTCCA | 137 |
| *SrUGT91D4* | CCACTATCGACGTTCACACTCAA | GACGGACGGCAACCAATAC | 144 |
| *SrUGT85C3-1* | AGCATCCACAACACCAGAGAACT | CAGGCAGCAAGTGACCAATACA | 191 |
| *SrUGT79A2* | GCAACAATCCTACGGCATACC | GCGATCCTTCCATCTCCCTAC | 146 |
| *SrUGT73G2* | TGAGGAGTTGGAGCAAGAGTA | CTACCGAAGCAGGCATACACA | 183 |
| *SrUGT71I1* | CGCCAGGTGTCGGTCATC | CGGTTAGAGCAGGAACAGAGA | 122 |
