# Supplementary figures and images for: Characterizing glycosyltransferases by a combination of sequencing platforms applied to the leaf tissues of Stevia rebaudiana
Source: BMC Genomics. 2020 Nov 13;21:794. doi: 10.1186/s12864-020-07195-5 (PMC7664074; doi:10.1186/s12864-020-07195-5)

Additional file 3


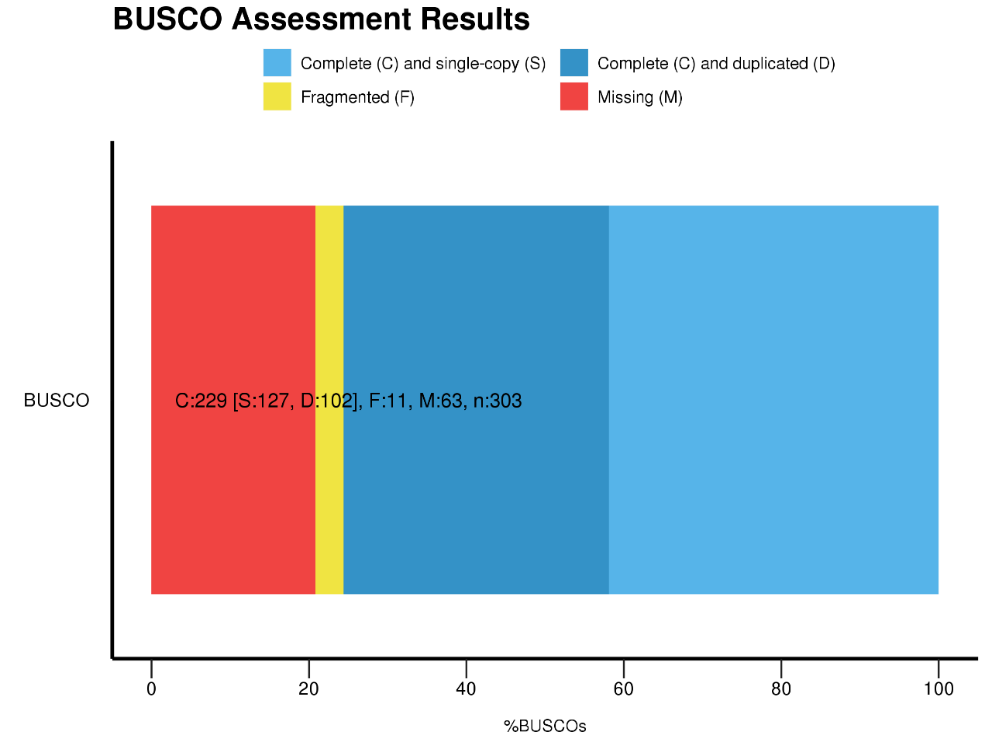


Figure S1. BUSCO assessment of the 30,859 corrected contigs

Supplement: Supplementary file 3 — Additional file 3: Figure S1. BUSCO assessment of the 30,859 corrected contigs. [file 12864_2020_7195_MOESM3_ESM.docx]
