# Supplementary material for: Characterizing glycosyltransferases by a combination of sequencing platforms applied to the leaf tissues of Stevia rebaudiana
Source: BMC Genomics. 2020 Nov 13;21:794. doi: 10.1186/s12864-020-07195-5 (PMC7664074; doi:10.1186/s12864-020-07195-5)
Supplement: Supplementary file 4 — Additional file 4: Table S3. Primers and annealing temperature of the twenty SrUGTs. [file 12864_2020_7195_MOESM4_ESM.docx]

Additional file 4

Table S3. Primers and annealing temperature of the twenty *SrUGT*s.

| ***SrUGTs*** | **F-primer (5′-3′)** | **R-primer (5′-3′)** | **Annealing temperature (℃)** |
| --- | --- | --- | --- |
| *SrUGT95A2* | ATGGATACCGAAAATCAGACC | TCAAGTTGCTTTTTTGCTAATAAAC | 47.5 |
| *SrUGT87B1* | ATGACGGAATCCGTCAACCA | CTACAGGTCTTTGTCACAACTCTTT | 52.0 |
| *SrUGT85A8-2* | ATGGCTTCAATAGCAGAAATGCAAA | TCACTTTCTTAAAAGAACATCGGTA | 49.0 |
| *SrUGT76I3* | ATGGATACCGATGGAAGAAATAC | TTATAACGACAAAATATAATCAAC | 45.0 |
| *SrUGT91D1-3* | ATGGACGACCATAAGCAGCTTC | TCAAAGCTCATGCTCAATAGCAACC | 54.0 |
| *SrUGT91D3* | ATGGCTAACACCGAGTCCTTG | TCATACGGTTAACGTACGTCGTG | 49.0 |
| *SrUGT75E2* | ATGACCAAAGTTATGGCTCAAACCC | TCATAGAACAGACTCCAACTTGGAT | 51.0 |
| *SrUGT85B2* | ATGGGTTCGAAACACGAAAAC | TCATATTGATGGAGTTTGCATGAG | 50.0 |
| *SrUGT91D1-1* | ATGTTCCCATGGCTTGCTTTC | TCAAAGCTCATGCTCAATAGCAACC | 51.0 |
| *SrUGT85B4* | ATGGGTTCGGTTCAAGAGAAA | TTATTTTGAAAATGTACGAATCAG | 46.0 |
| *SrUGT79A3* | ATGACTTTAACCTGCAAACC | TTATGAAAGAGCCTTCAAGTTC | 47.5 |
| *SrUGT85B3* | ATGGATTCGAAACAAGAAAAAAAGC | TCACTTTGAAAATGTTTTCATGAGA | 47.0 |
| *SrUGT71I1* | ATGGCGACCGAAGTTGCAGA | TCATACGATGTTACTCATGATATCC | 50.5 |
| *SrUGT78D2* | ATGGATACCACCAAAAGCTCCA | TCAAGTGGCAGTACCCGTGACC | 51.5 |
| *SrUGT91D4* | ATGGCTACCACCGAAAACCG | TTAACTCTCATGGTTGTTGGCAACC | 53.0 |
| *SrUGT85C3-1* | ATGGATGCAGTGGTCGAAACAGAAA | CTAGTCTCTTGATAGCACCGT | 51.0 |
| *SrUGT75F1* | ATGTCAAGCAATCGGAAAATC | TTAGTTTCCAAGGTCATTCAAGA | 47.5 |
| *SrUGT76H2* | ATGGGAGATGAGAGAAACCAAA | CTAAGTTGTAACACTTTGTAAAAC | 47.0 |
| *SrUGT88B2* | ATGGGCCACCTGGTTTCCAT | TTACATGTTTGTCCAGGAGT | 47.0 |
| *SrUGT73G2* | ATGGCTTTGGAATCAGTCAAC | TTAGCTACAATAAGTAGTTTCTGGC | 49.5 |
