# Supplementary material for: Characterizing glycosyltransferases by a combination of sequencing platforms applied to the leaf tissues of Stevia rebaudiana
Source: BMC Genomics. 2020 Nov 13;21:794. doi: 10.1186/s12864-020-07195-5 (PMC7664074; doi:10.1186/s12864-020-07195-5)
Supplement: Supplementary file 5 — Additional file 5: Table S4. Position in S. rebaudiana genome of the forty-four candidate SrUGTs. [file 12864_2020_7195_MOESM5_ESM.docx]

Additional file 5

Table S4. Position in *S. rebaudiana* genome of the forty-four candidate *SrUGT*s.

| Candidate genes | Position in genome | Candidate genes | Position in genome |
| --- | --- | --- | --- |
| SrUGT71I1 | >WOUH01000778.1(P5626437) | SrUGT85B1-2 | >WOUH01007778.1(P31165388) |
| SrUGT74G1-2 | >WOUH01000778.1(P23817375) | SrUGT85C4 | >WOUH01007778.1 (P12642818) |
| SrUGT91D1-3 | >WOUH01000778.1(P19394746) | SrUGT76I3 | >WOUH01007778.1(P37659447) |
| SrUGT91D1-1 | >WOUH01000778.1(P19394746) | SrUGT71H1 | >WOUH01003985.1(P2017) |
| SrUGT73C1 | >WOUH01004839.1(P5037) | SrUGT76H2 | >WOUH01000308.1(P439) |
| SrUGT73C4 | >WOUH01004839.1(P2644) | SrUGT79A1 | >WOUH01007778.1(P20274205) |
| SrUGT73C3 | >WOUH01004839.1(P7770) | SrUGT79A2 | >WOUH01004751.1(P8798) |
| SrUGT73C1 | >WOUH01004839.1(P5037) | SrUGT85B2 | >WOUH01000057.1 (P6105483) |
| SrUGT73C4 | >WOUH01004839.1(P2644) | SrUGT91D4 | >WOUH01000008.1(P207926) |
| SrUGT73C3 | >WOUH01004839.1(P7770) | SrUGT75F1 | >WOUH01015533.1(P1401) |
| SrUGT75E2 | >WOUH01005557.1(P17844656) | SrUGT85B3 | >WOUH01022408.1(P5) |
| SrUGT85B4 | >WOUH01005557.1(P3161467) | SrUGT88B2 | >WOUH01002233.1(P7186) |
| SrUGT85C3 | >WOUH01005557.1(P34459789) | SrUGT71A15P | >WOUH01002667.1(P8254) |
| SrUGT87B1 | >WOUH01005557.1(P92690836) | SrUGT85C5P | >WOUH01002458.1(P10078) |
| SrUGT91D3 | >WOUH01005557.1(P72832223) | SrUGT85C6P | >WOUH01003326.1(P3106) |
| SrUGT92C1 | >WOUH01005557.1(P90385569) | SrUGT75C1P | >WOUH01000061.1(P25373) |
| SrUGT76G1-1 | >WOUH01005557.1(P88275449) | SrUGT71E2P | >WOUH01017008.1(P654) |
| SrUGT85C2-1 | >WOUH01005557.1(P34457130) | SrUGT87A2P | >WOUH01005557.1（P4076813） |
| SrUGT78D2 | >WOUH01005557.1(P3755841) | SrUGT79A4 | >WOUH01005557.1(P72711656) |
| SrUGT95A2 | >WOUH01005557.1(P73836288) | SrUGT91A1P | >WOUH01000078.1(P1311390) |
| SrUGT85C1P | >WOUH01005557.1(P83528949) | SrUGT85E1 | >WOUH01000557.1(P23839145) |
