# Supplementary material for: Characterizing glycosyltransferases by a combination of sequencing platforms applied to the leaf tissues of Stevia rebaudiana
Source: BMC Genomics. 2020 Nov 13;21:794. doi: 10.1186/s12864-020-07195-5 (PMC7664074; doi:10.1186/s12864-020-07195-5)
Supplement: Supplementary file 6 — Additional file 6. Supplementary information, nucleotide and protein sequences used for phylogenetic tree in this study. [file 12864_2020_7195_MOESM6_ESM.docx]

Additional file 6

Nucleotide and protein sequences used for phylogenetic tree in this study

SrUGT71E1

ATGAACCAAATTCAGACCGAAAACACAGATATCAAATCATCAACCTCACAAAAACAAATGTCCACCTCAGAGCTTGTTTTCATCCCATCTCCCGGAGCTGGCCACCTGCCACCAACGGTCGAGCTCGCAAAGCTTCTGTTACATCGCGATCAACGACTTTCGGTTACAATCATCATCATGAATCTCTGGTTAGGTCCAAAACACAACACTGAAGCACGACCTTGTGTTCCCGGTTTACGGTTCGTTGACATCCCTTGCGATGAGTCCACCATGGCTCTCATCTCACCCAAAACTTTTCTATCTGCGTTCGTTGAACACCACAAACCTCGTGTTAGAGACATAGTCCGAGGTATAATTGAGTCTGACTCGGTTCGACTCGCTGGGTTCGTTCTTGATATGTTTTGTATGGCGATGAGTGATGTTGCAAACGAGTTTGAAGTTCCGAGTTACAATTATTTCACATCCGGTGCAGCCACGTTAGGGTTGATGTTTCACCTTCAATGGAAACGTGATCATGAAAGTTATGATGCAGCCGAGTTGAAAAACTCGGATACTGAGTTGTCTGTTCCGAGTTATGTTAACCCGGTTCCTGCTAAGGTTTTACCGGAAGTGGTGTTGGATAAAGAAGGTGGGTCCAAAATGTTTCTTGACCTTGCGGAAAGGTTTCGCGAGTCGAAGGGTATAATAGTAAATTCATGTCGGGCGATTGAAAGACACGCGCTCGAGTACCTTTCAAGCAACAATAACGGTATCCCACCTGTTTTCCCGGTTGGTCCGATTTTGAACCTTGAAAACAAAAAAGACGATGCTAAAACCGACGAGATTATGAGGTGGTTAAATGAGCAACCGGAAAGCTCGGTTGTGTTTTTATGTTTCGGAAGCATGGGTAGCTTTAACGAGAAACAAGTGAAGGAGATTGCGGTTGCGATTGAAAGAAGTGGACATAGATTTTTATGGTCGCTTCGTCGTCCGACACCGAAAGAAAAGATAGAGTTTCCGAAAGAATATGAAAACTTGGAAGAAGTTCTTCCAGAGGGATTCCTTAAACGTACATCAAGCATCGGGAAGGTGATCGGGTGGGCCCCGCAAATGGCGGTGTTGTCTCACCCGTCAGTTGGTGGGTTTGTGTCGCATTGTGGTTGGAACTCGACATTGGAGAGTATGTGGTGTGGGGTTCCGATGGCAGCTTGGCCATTATATGCTGAGCAACAGTTGAATGCTTTTCTACTTGTGGTGGAACTGGGATTGGCGGCGGAGATTAGGATGGATTATCGGACGGATACGAAAGCGGGGTATGACGGTGGGATGGTGGTGACGGCGGAGGAGATTGAAGATGGAATTAGGAAGTTGATGAGTGATGGTGAGATTAGAAATAAGGTGAAAGATGTGAAAGAGAAGAGTAGAGCTGCGGTTGTTGAAGGTGGATCTTCTTACGCATCCATTGGAAAATTCATCGAGCATGTATCGAATGTTACGATTTAA

MNQIQTENTDIKSSTSQKQMSTSELVFIPSPGAGHLPPTVELAKLLLHRDQRLSVTIIIMNLWLGPKHNTEARPCVPGLRFVDIPCDESTMALISPKTFLSAFVEHHKPRVRDIVRGIIESDSVRLAGFVLDMFCMAMSDVANEFEVPSYNYFTSGAATLGLMFHLQWKRDHESYDAAELKNSDTELSVPSYVNPVPAKVLPEVVLDKEGGSKMFLDLAERFRESKGIIVNSCRAIERHALEYLSSNNNGIPPVFPVGPILNLENKKDDAKTDEIMRWLNEQPESSVVFLCFGSMGSFNEKQVKEIAVAIERSGHRFLWSLRRPTPKEKIEFPKEYENLEEVLPEGFLKRTSSIGKVIGWAPQMAVLSHPSVGGFVSHCGWNSTLESMWCGVPMAAWPLYAEQQLNAFLLVVELGLAAEIRMDYRTDTKAGYDGGMVVTAEEIEDGIRKLMSDGEIRNKVKDVKEKSRAAVVEGGSSYASIGKFIEHVSNVTI

SrUGT71F1

ATGGGTCATGCTGGTCAAGCCATTGAGCTAGCCAGACTCTTCGTCGAACGATTTCACCATCTAACCATCACGGTGCTCGTGATGAAGCTACCCGGTGATCCAATCGGCAATGAATACACCGATTCTCTCACCGACAAAAGTTGCATCAAATTCATCCATTTCCCTCCAATGGATCTTGATTTGTTTCAAGATTGTTCAAACTTTGGGTTCTTGGCCGAAGAAGTTATTTCTCGCCATAAGCCCATCATAAGGGATCTTGTGGCTAGTCGGTTCAACAGGTCTGGGTCTGATCATCGACTGGGTGCGTTGGTTGTTGACATGTTTTGTACACCAATGATCGATGTTGCCAAGGAGTTTAACATCCCAGCTTACGTGTTCTTCCCCTCTAATGCCGCTTTTCTTGGAATCATCTTTCATTTCCAGACCCTTGACGATGAACATGGTCAAAACATATTAGAACTCGTACACTCAGACACTGATCTAACGGTCCCAAGCTATGCCAACCCGGTTCCTCCGAGTGTCATGCCTACGGTGTTTTGGGCTAAGGATCAATGGCCCAAAAGGTTCATTATTCACACCCGAAAGTACAGAGAAGCCAAGGGCATAATCGTAAATACGTTTCAAGAGCTTGAACATCACGCCCTTCGATCCTACGATGATAAAACACCTCCGGTGTACCCGGTGGGTCCTTTGTTGAAACCCATAGAACCGACACCGAGCAACGAAGTGTTGCAATGGTTGAAAGATCAACCGAAGTCATCGGTCCTACTTTTATGCTTTGGGTCTAGGGGTTGCTTCGATGCGGACCAAGTGAGAGAGATAGCGGTTGCAATAGAAAGAAGCGGATGCCGGTTCATATGGTCCCTACGCCGGCCTTCTAACGACGAGCAAAAAGGATTGCCGGATGAATACACGGATTACAACGAAGTTTTGCCATATGGATTCCTCGAGCGTACAGCTGGAAAGGGGAAAGTGATTGGGTGGGTCCCACAGTCGGAAGTGCTGGCTAACGTGGCGACCGGGGGGTTCGTGTCCCACTGCGGGTGGAACTCCATACTCGAGAGCCTTTGGTACGGGGTTCCGGTCGCAACATGGCCAATATACGCAGAGCAACAACTAAATGCATTTCAAATGGTTAAAGACTTGGGTTTGGCGGTTGAAATTTCTTTGGATTATAACCAAATAAACAAGAATCAAAAGCCAGTTTCAGCGGAAGACATTGAGAAGGGAATAAGAGAAGTGATGGATAGCAATAGCGCGGTTAGAGCCAACGTGAAGAAGATGAAAGATACGGGCCGAATGGCGATTGAAGAAGGCGGTTCATCGTTCGAAAGTTTGAGTCGTCTTGTTAATGATATTATGTAG

MGHAGQAIELARLFVERFHHLTITVLVMKLPGDPIGNEYTDSLTDKSCIKFIHFPPMDLDLFQDCSNFGFLAEEVISRHKPIIRDLVASRFNRSGSDHRLGALVVDMFCTPMIDVAKEFNIPAYVFFPSNAAFLGIIFHFQTLDDEHGQNILELVHSDTDLTVPSYANPVPPSVMPTVFWAKDQWPKRFIIHTRKYREAKGIIVNTFQELEHHALRSYDDKTPPVYPVGPLLKPIEPTPSNEVLQWLKDQPKSSVLLLCFGSRGCFDADQVREIAVAIERSGCRFIWSLRRPSNDEQKGLPDEYTDYNEVLPYGFLERTAGKGKVIGWVPQSEVLANVATGGFVSHCGWNSILESLWYGVPVATWPIYAEQQLNAFQMVKDLGLAVEISLDYNQINKNQKPVSAEDIEKGIREVMDSNSAVRANVKKMKDTGRMAIEEGGSSFESLSRLVNDIM

SrUGT71H1

ATGGGCAACCTTGTTCCCGCCGTAGAATTTGCAACCCACTTAATCAACCACCATCTCCGTCGTATCTCCGTCACAATACTCACCATCTCCATGCCTCAGTGGCCAATCATGGATCACTATCTCCGATCTCAAACATCCACCACTCACATCCGGTTCATCCAACTCCCTCCGGCCGACACACCATCACCAAATCAATACAACGCACGCATCGAATTCATTTCACTCTATATACAAAACCACAAACCCATAGTCCAACAAACACTCAAAAACCTTGCCACCAACAATTCCAACGAGCCACTCGTCGGCTTTTTCTTCGACATGTTTTGCACCTCGATGATTGACGTCGCAAATGATCTAAACATTCCTTGTTATCTCTACTTCGCGTCTCCGGCCGCTTATCTTGGTTTTGTAATCCACCTCACATCACTGCCAGACTCGGTCACTGAGTCTAACGACTCAGACTCAGTGACCGAGTTGACGGTACCGAGTTTCATCAACCCGGTTCCGTCAGCCGCCTTTCCGTTATTTTGTATTAACAAAAACGAACTTGGTTACTCATGTTTTGTACGTCATGCGGCTAGGTATAAAGACACCAAGGGCATAGTTGTAAATACGTTCCAAGAGCTTGAACCATACGCGCTTGACTCTCTGTCTTCTGATTACACCGAGTTGCCACCGGTTTACCCGATTGGACCGGTGGTTGATCATGTGGGACCGGTTAAGTGGCATCCTAACCGATCCGGGCATGAAAAGGTGATCAAGTGGCTCGACCAACAACCTGATTGTTCGGTTGTTTTCTTGTGTTTTGGTAGCATGGGGAGTTTGAACCGGGCCCAAGTGAGGGAAATAGCAACGGGTCTGGATCGGGCCGGGTACCGGTTTTTGTGGTCCTTTCGTGAACCCGCGAAGACGAAGCTGGAGCTTCCAGACGACTATGAAGAACTCGAAGATGATTTGTTTCCAGATGGGTTTGTTAATCGAACGGCCGATATTGGGTTGGTGTGTGGTTGGGTTTCTCAAGTGAGTGTGTTGGCTCACAAAGCGATTGGTGGGTTTGTGTCACATTGTGGTTGGAACTCAATTTTGGAGAGCTTATCGTACGGTCTACCGATTGCCACGTGGCCATTGTACGCGGAACAACATTTGAATGCGTTTGAGATGGTCAAAGAGGTTGGATTGAGTTTGGAGATAAGATTCGATATGGGGAATGATCTGGTGTTGGCGGATGAAGTGGAGAGAGGTGTGAGGGAGTTGATGGACGGTGGTGATGGTGAGTTGAAGAAAAAAGTCAAAGAAATGAGTGAAAAGAGTAAAATAGCTTTGATGAAAAACGGGTCTTCATTTCAAGCTCTAGAGAAATTGACGAATGTTCTTCTATCCGAAGTTCAAAATTAA

MGNLVPAVEFATHLINHHLRRISVTILTISMPQWPIMDHYLRSQTSTTHIRFIQLPPADTPSPNQYNARIEFISLYIQNHKPIVQQTLKNLATNNSNEPLVGFFFDMFCTSMIDVANDLNIPCYLYFASPAAYLGFVIHLTSLPDSVTESNDSDSVTELTVPSFINPVPSAAFPLFCINKNELGYSCFVRHAARYKDTKGIVVNTFQELEPYALDSLSSDYTELPPVYPIGPVVDHVGPVKWHPNRSGHEKVIKWLDQQPDCSVVFLCFGSMGSLNRAQVREIATGLDRAGYRFLWSFREPAKTKLELPDDYEELEDDLFPDGFVNRTADIGLVCGWVSQVSVLAHKAIGGFVSHCGWNSILESLSYGLPIATWPLYAEQHLNAFEMVKEVGLSLEIRFDMGNDLVLADEVERGVRELMDGGDGELKKKVKEMSEKSKIALMKNGSSFQALEKLTNVLLSEVQN

SrUGT71I1

ATGGCGACCGAAGTTGCAGAGCTCGTGTTCATCCCAACGCCAGGTGTCGGTCATCTAATGGTCACAATCGAGATGGCAAAACTACTCTTGAACCGCGATCAAAGACTTTCAATAACCGTTCTTCTCATCAACCCTCCTTTCTCTGTTCCTGCTCTAACCGCATACATTGAATCGTTGTCTAAAAACACAATCGAACGCGTACGATTCATCAACCTCCCGCATAATCCAACATCACCAAAATTCGACCCCAAAGCTCCCGTTACTTCTTTCTTTGAGTTCATCAACAGTCATTGCGAATACGTCAGAGATATCGTTACTGACATTATAAATCAAACCGGTTCGAGTCGGATCGTTGGGTTTGTTGTTGACATGTTATGCACGGGCTTGATCGATGTGGTTAATGAATTTAATGTCGCGTCATACGTTTTCTACCCGTCTAATTCTGCTTTTATCGGTTTAAACATGTATATCGAGACACAACATGTGGATCAGAAACAAGATCTTGTTAAAATGAGTTATGAATCCGAAGGCGAGATTTCTTTTCCGTGTTTTGTTAATCCGATACCGACGAGGGTTGTTCGGGCGGTGTATCAGAAAGACGAGCGAACGGAGTTTCTAGTACAATGTTTTCGGAATTTGAGAAAGGCTAAAGGGATTATGGTTAATACGTTTTTGGAGTTGGAAACACACGCGATCAATTCGTTTTCGAAAACTAACTTCCCGCGCGTGTATCCTGTGGGACCCGTACTTAATCTAGACGGTGTTGATGGAAAAGCTGAAGACAAGGATGTACTAAGTTGGTTGGACGATCAACCGCCTTCTTCAGTGGTGTTGTTATGTTTTGGTAGTATGGGAAGCTTTATTAAGCCCCAAGTGGTGGAAATAGCGCGCGGTTTAGAACAAAGTGGTCATCGTTTTGTTTGGTCTCTGCGCGTACCTCCACCGTCAAAACAATCGTTTGATGTGGTTCCTGATAATTACGACGACCCAAGAAGGCTATTGCCGGATGGGTTCCTTGAGCGAACCATGGGAATAGGGAAAGTGATTGGATGGGCCCCACAAGTGGCGTTGTTGGTCCATGAAGCGGTTGGAGGGTTCGTGACCCACTGTGGATGGAACTCGATGCTAGAGAGCTTGTGGTTCGGTGTACCAACAGCTACATGGCCAATGTATTCTGAGCAGCAGATGAATGCATTTGAAATGGTGGTGGAGCTTGGACTCGCGGTTGACTTGAAGATGGATTTTAATATGAACGTTTTTAATCGTGAGAGTGAGATCGTTGTTGTGACGGCAAAGGAGATAGAGACGGGAATAAGGCGACTAATGGAGGACGAAAAGGTCAGAACGAAAGTGATAGAGATGAGTAAATTGAGTAGAGCAACAGTGGTTGATGGTGGTTCATCGTATGCTTCGGTTGGCTACCTTGTTCAGGATATCATGAGTAACATCGTATGA

MATEVAELVFIPTPGVGHLMVTIEMAKLLLNRDQRLSITVLLINPPFSVPALTAYIESLSKNTIERVRFINLPHNPTSPKFDPKAPVTSFFEFINSHCEYVRDIVTDIINQTGSSRIVGFVVDMLCTGLIDVVNEFNVASYVFYPSNSAFIGLNMYIETQHVDQKQDLVKMSYESEGEISFPCFVNPIPTRVVRAVYQKDERTEFLVQCFRNLRKAKGIMVNTFLELETHAINSFSKTNFPRVYPVGPVLNLDGVDGKAEDKDVLSWLDDQPPSSVVLLCFGSMGSFIKPQVVEIARGLEQSGHRFVWSLRVPPPSKQSFDVVPDNYDDPRRLLPDGFLERTMGIGKVIGWAPQVALLVHEAVGGFVTHCGWNSMLESLWFGVPTATWPMYSEQQMNAFEMVVELGLAVDLKMDFNMNVFNRESEIVVVTAKEIETGIRRLMEDEKVRTKVIEMSKLSRATVVDGGSSYASVGYLVQDIMSNIV

SrUGT95A2

ATGGATACCGAAAATCAGACCAAGAAAAGAAAGCTTGAATCCACAACAATGGAAGCTGCCGGTGAGATCTTTGTTCTACCATTCTTCGGTCAAGGTCATCTCAACCCATCGATGGAACTCTGCCGGAACATCTCCTCTCATAACTTCAATGTCACACTCATCATCCCCTCTCACCTTTCTTCATCGATCCCTACAACTTTTCCCGGCGATTCGCCCTTCATTCATGTTGCAGAGATTCCGTTCGCCGCTTCTCCGCCGGAAACAGAGGCTCCGAATTGGGGGAACCGTTTTGAACAGCAGAATAAACAGATGGGTGAGGGAATCAAGTCGTTTTTGTCGAATAGATCCGGAATCCGACCCACGTGCGTTGTGATTGATGTTATGATGAGCTCGATCAAGGAGATTTTTGCAGATCACCGGATTCCGGTGGTGTCGTTTTTCACTTCCGGCGTGACGAATTACGCCATAGAGCACGGAAAGTGGAAGGCGAAGATCGGAGACTTGAAACCCGGTGAGACCCGAGAGTTACCCGGGTTACCCAAAGAAATGGCTGCTACTTATTCGGATCTTTTCAAAGGTCCGAGAGGAAGACCTCAGAGACCCAACCTGCCGACTGGAGATCACGCAAACCGGGTCGGACCGCCACATGGATCAAGGGGCCACCGCGGTCCACCCGGTCCGGGTGATAAGCCACGTTGGGTTGACGAAGTTGATGGAGCGGTCGCGTTGCTTATCAACACATGCGACAATCTCGAGCGTTTGTTCCTCGATTACCTCGCGGAACAAACCAAGGTTCCGGTGTGGGGCGTCGGACCGCTCCTACCGGAAAAATTCTGGCAATCCGCCGGTTCAATCCTCCACGATCGGGATATGAGATCGAATCGGAAAGCTAATTACTCAGAAGACGAAGTTTTCCAATGGCTAGAGTCCAAACCAATAGGTTCGGTGATCTACATCGCATTCGGAAGTGAAGTTGGACCATCGATTGATGAATACAAAGAGCTAGCGAAAGCGTTGGAAGAATCGAACCACGCTTTCATATGGGTGATCCAACCCGGTTCGGGTAAAAACCCGATCCCAAAATCTTTTTTAGGACCGGTTCAAACCGATAGCGAAGAAGAACAAGAAGGTTACTATCCAGATGGTTTAGACAAAATAGTTGGGAATAGGGGTATGATCATCACCGGATGGGCCCCACAATTGTTGATTCTGAGCCACCCATCAACCGGTGGGTTCTTGTCACACTGCGGTTGGAACTCGACTGTTGAGGCGATTGGTCGAGGGGTCCCGATCTTGGGTTGGCCAATTAGGGGTGATCAGTTTGAAAATGCGAAGCTGGTGGCTAACCATCTCAAGATTGGGTTCGTGATTGCAAGTGGGGTCGGTGAAGACGGTCGGCCGAAAAGGTTCAACAAGGATGATATAGCAACAGGGGTTGAGAAACTGATGAGTGATGAAGAGGTTCATGAAAATGCCAAGAAATTAAGTAAGGAGTTTGAAAGTGGGTTTCCAGTGAGTTCGGTTAATGCTTTGGGTGCATTTGTGGAGTTTATTAGCAAAAAAGCAACTTGA

MDTENQTKKRKLESTTMEAAGEIFVLPFFGQGHLNPSMELCRNISSHNFNVTLIIPSHLSSSIPTTFPGDSPFIHVAEIPFAASPPETEAPNWGNRFEQQNKQMGEGIKSFLSNRSGIRPTCVVIDVMMSSIKEIFADHRIPVVSFFTSGVTNYAIEHGKWKAKIGDLKPGETRELPGLPKEMAATYSDLFKGPRGRPQRPNLPTGDHANRVGPPHGSRGHRGPPGPGDKPRWVDEVDGAVALLINTCDNLERLFLDYLAEQTKVPVWGVGPLLPEKFWQSAGSILHDRDMRSNRKANYSEDEVFQWLESKPIGSVIYIAFGSEVGPSIDEYKELAKALEESNHAFIWVIQPGSGKNPIPKSFLGPVQTDSEEEQEGYYPDGLDKIVGNRGMIITGWAPQLLILSHPSTGGFLSHCGWNSTVEAIGRGVPILGWPIRGDQFENAKLVANHLKIGFVIASGVGEDGRPKRFNKDDIATGVEKLMSDEEVHENAKKLSKEFESGFPVSSVNALGAFVEFISKKAT

SrUGT85B4

ATGGGTTCGGTTCAAGAGAAAAAGGCGCCACATGTTGTGTGCATACCGGCACCACTTCAAGGTCACATTAACCCGATGCTAAAACTAGCCAAAATCCTGCACTCCAAAGGCTTTCTTATCACCTTTGTCAACACCGAGTTTAACCACCAACGGCTCGTTAGGTCACAGGGGGTTGAAGCCCTACACGGGCTCCCAACCTTCCGGTTTGAGACCATCCCAGATGGTCTACCGCCACCTGAAAACAAAGATGCCACCCAAGATATCCCGACTCTAGCCAAGTCGGTTGATGAAAACTTTTTGGGTCCGTTTAAAAGTCTTGTAACCAAAGTGGGTGCTTTGTATGCACCCGTGACTTGTATCGTGTCTGACATGCTTATGTGCTTCACTCTTGATGCCGGTGCTGAATTGGATATCCCGGTAATACTCCATTGGACCAGTGGTACTGGTTCTTTGATATGTTACAATGAATATCCTAATCTATTGGAAAGCAAATTGATGCCCCTCAAAGATGCAAGTTATTTAGTGAATGGTTACTTAGATACGATTGTAGATTCTATCCCCATTTTGCATGGCATACGTTTAAGAGATTTCCCTCCCTTCATTAGAAAGATCTTTCCTGGTGATGAGTTCATGGTTCAATTTTTGACTTCACAAGTAAACAAAGCAAAAAACGGATCTTCTGCTATCATTTTCAACACTTTTGATGAACTAGATCGTGATGTTTTAGACACACTCGCTTCAATGTATCCTCCATGTTATGGAATTGGTCCGTTACATCTACTAGAGAAACATGTTACCGATAAATCTCTTGATTTCGTGAAATCAAACCTTTGGAAAGAAGAACCCGAATGTTTAAAATGGTTAGATACACAAGCTCCATCATCAGTCATTTATGTGAATTTTGGTAGCATTACAGTAATGACACCTCAACAACTAGTCGAGTTTTGTTGGGGACTCGCAAAGAGCAACTATCCGTTCTTATGGATAATACGACCTGACCTTGTGATTGGTGATTCCGCGATGCTTCCACCCGAGTTTGTAAAGGAAACAAGTGATAGAGGGATGCTGGTTGGATGGTGTCCTCAAGAAGAAGTTTTGAATCACCCGTCAATTGGAGGGTTTTTAACGCACAGTGGATGGAATTCAACGCTTGAAAGTATTTCGAGTGGTGTGCCGATGATTTGTTGGCCGTTTTTTGCGGATCAACAAACGAATTGCTGGTGGAGTTGCAACAAATGGGGTGTTTCCATGGAGATTGATAATAATGTGAAGAGTGATGAAGTTTCAAAGCTTGTGATTGAATTAATGGATGGAGAAAAAGGAAAGGAAATTAAGAAGAATGCCATTGACTTGAAGAATAAAGCTAAGGATGCATGTACCTCTCCTTTGGGTTCATCCGTGGCTAATTTGGAGAAAGTGGTTCAACTGATTCGTACATTTTCAAAATAA

MGSVQEKKAPHVVCIPAPLQGHINPMLKLAKILHSKGFLITFVNTEFNHQRLVRSQGVEALHGLPTFRFETIPDGLPPPENKDATQDIPTLAKSVDENFLGPFKSLVTKVGALYAPVTCIVSDMLMCFTLDAGAELDIPVILHWTSGTGSLICYNEYPNLLESKLMPLKDASYLVNGYLDTIVDSIPILHGIRLRDFPPFIRKIFPGDEFMVQFLTSQVNKAKNGSSAIIFNTFDELDRDVLDTLASMYPPCYGIGPLHLLEKHVTDKSLDFVKSNLWKEEPECLKWLDTQAPSSVIYVNFGSITVMTPQQLVEFCWGLAKSNYPFLWIIRPDLVIGDSAMLPPEFVKETSDRGMLVGWCPQEEVLNHPSIGGFLTHSGWNSTLESISSGVPMICWPFFADQQTNCWWSCNKWGVSMEIDNNVKSDEVSKLVIELMDGEKGKEIKKNAIDLKNKAKDACTSPLGSSVANLEKVVQLIRTFSK

SrUGT87B1

ATGACGGAATCCGTCAACCACCATGTGGTGGCGATACCATATCCCGGCAGAGGCCACATCAACCCGATGATGAACCTCTGCAACCTCATCTCTCTACGCCGACCATCCGACTTCCTCATCACCGTCGTCGTCACCGAAGAGTGGTTGGGCTTCATCGGAAGTGAACCTAAACCGACCAACATCCGGTTCGCCACCATCCCTAACGTCATCCCGTCGGAGGTCAACCGAGCCGCTGATTTCACCGGATTTACAAAAGCCACACAAACCAATATGGAAGATCCGGTCGAGCAGTTGCTCCGGCGGATGGAAATACCGGCTAGTGTTATCATATACGATACTTACCTTCCATGGGTGTTGAACATCGGAAACCGGATGAATACTCCGGTGGCGAGTTTGTTTACGATGTCGGCAACGGTGTTCTCCATGTGTTACAATTACCATCTTCTCGTTGAAAATCACCACGTTGGTGATAACTTCTCTGGAAACACCGAGGAAATAGTAGATTACATACCCGGAGTTCCTCCGATGAAGGTGGCTGATCTTTTGACAGGCTTCAATGGCAATGGCAAAGAAGTTACACCGATAGCTTTGAATGGCATTATGATTTCTCCAAAAGCTCAGTTTCTCATTTTCGCATCAGTTTATGAGCTCGAAGCTCATGTCATTGATGCTTTAAAGTCGGATCTTTCTATACCAGTTTATGCGATCGGACCTGCAATTCCTTACTTTAATTTCAATGACGTGGAAAAAGACCAAAATACCCCTGACTACTTCAAATGGTTGGACCGTCAACCAGAGGGTTCGGTGTTGTACATTTCACAAGGGAGTTTTCTCTCGGTCTCGAGCGCCCAACTAGATGAAATCGTGGCGGGTGTGCTGGATAGTGGAGTGCGATACATGTGGATTATGCGTGACGAAACGTCTCGCTTTAAACGTGAAGATGATGATAAAGGGATAGTTGTACCTTGGTGTGACCAATTACGAGTGTTGTGCCATCGTTCTGTAGGGGCATTTTGGTCACATTGCGGGTGGAATTCGACTAAAGAAGGTGCTTATTCGGGAAAGCCCATGATTTGTTTTCCCATATTTTGGGATCAAGTCCCGAATAGTAAAATGATAGTTGAAGACTGGAAAATCGGTCGGAGAGTGAAGAACAATGAGGGTAATATGGTCACTCGAGATGAAATTTCGATACTCATTAAAAGTTTTATGGATCCAAACGGGAAAGATATAAGGAACCGGAGTAAAGAAGTTGAAAAGATTTGTCGGCAAGCAACATCAGAAGGTGGATCTGCCCAAATAGATATTGATTCATTCATTCGCGACATTTCAAAGAGTTGTGACAAAGACCTGTAG

MTESVNHHVVAIPYPGRGHINPMMNLCNLISLRRPSDFLITVVVTEEWLGFIGSEPKPTNIRFATIPNVIPSEVNRAADFTGFTKATQTNMEDPVEQLLRRMEIPASVIIYDTYLPWVLNIGNRMNTPVASLFTMSATVFSMCYNYHLLVENHHVGDNFSGNTEEIVDYIPGVPPMKVADLLTGFNGNGKEVTPIALNGIMISPKAQFLIFASVYELEAHVIDALKSDLSIPVYAIGPAIPYFNFNDVEKDQNTPDYFKWLDRQPEGSVLYISQGSFLSVSSAQLDEIVAGVLDSGVRYMWIMRDETSRFKREDDDKGIVVPWCDQLRVLCHRSVGAFWSHCGWNSTKEGAYSGKPMICFPIFWDQVPNSKMIVEDWKIGRRVKNNEGNMVTRDEISILIKSFMDPNGKDIRNRSKEVEKICRQATSEGGSAQIDIDSFIRDISKSCDKDL

SrUGT85A8-2

ATGGCTTCAATAGCAGAAATGCAAAAGCCACATGCCATATGCATCCCCTACCCAGCCCAAGGCCACATCAACCCCATGATGCAATTTGCTAAGCTCCTTCACTTCAAAGGCTTTCACATCTCTTTTGTCAATAACCACTACAACCATAAGCGGTTGCAGCGGTCCCGCGGTCTGTCCGCCCTCGAAGGTCTACCTGATTTTCATTTCTACTCGATTCCCGATGGCCTTCCGCCTTCAAATGCTGAGGCCACCCAGTCGATCCCCGGGCTATGTGAGTCGATTCCTAAGCACAGTTTGGAACCATTTTGTGAATTGATCGCTACGCTAAATGGTTCGGACGTGCCACCTGTAAGCTGTATAATCTCTGACGGGGTCATGAGCTTTACGCTTCAAGCTGCCGAGAGGTTCGGGTTGCCGGAAGTTTTGTTCTGGACCCCAAGTGCTTGTGGGTTTTTGGCTTACACTCACTATCGAGATCTTGTGGATAAGGAGTATATTCCCCTCAAAGACACGAAGGACTTGACAAATGGGTATTTAGAAACAAGCTTGGATTGGATTCCTGGGATGAAAAACATCCGATTAAAAGATTTCCCATCCTTTATTCGAACCACAGACATAAATGATATTATGCTCAATTATTTCTTGATTGAAACCGAAGCGATCCCAAAAGGCGTAGCGATCATTCTTAACACATTTGACGCGTTAGAAAAAGATAGTATTACGCCTGTACTTGCTCTAAATCCACAAATATACACCATTGGTCCATTACACATGATGCAACAATATGTCGATCATGATGAGAGACTCAAACACATTGGGTCCAACCTTTGGAAGGAAGATGTGAGCTGCATCAATTGGCTTGACACCAAAAAGCCTAATTCGGTTGTTTATGTGAACTTTGGAAGTATTACGGTTATGACGAAAGAACAACTGATCGAGTTTGGGTGGGGACTGGCTAATAGCAAGAAGGATTTCTTGTGGATAACGAGGCCTGATATTGTTGGAGGCAATGAAGCCATGATACCACCAGAGTTCATAGAGGAGACCAAAGAAAGGGGCATGGTTACTAGCTGGTGCTCTCAGGAAGAGGTTTTAAAACATCCATCAATCGGGGTATTCTTGACTCATAGTGGATGGAACTCGACCATTGAGAGTATTAGCAACGGTGTTCCCATGATTTGTTGGCCTTTTTTTGCAGAGCAACAAACAAATTGTCGGTATTGTTGTGTTGAATGGGAAATTGGATTGGAAATTGATACAGATGTGAAGAGAGAGGAGGTAGAGGCTCAAGTGAGGGAGATGATGGATGGGTCGAAAGGGAAGATGATGAAAAACAACGCTTTGGAATGGAAGAAGAAGGCTGAAGAAGCGGTATCCATTGGTGGATCTTCTTATCTCAACTTTGAAAAATTAGTTACCGATGTTCTTTTAAGAAAGTGA

MASIAEMQKPHAICIPYPAQGHINPMMQFAKLLHFKGFHISFVNNHYNHKRLQRSRGLSALEGLPDFHFYSIPDGLPPSNAEATQSIPGLCESIPKHSLEPFCELIATLNGSDVPPVSCIISDGVMSFTLQAAERFGLPEVLFWTPSACGFLAYTHYRDLVDKEYIPLKDTKDLTNGYLETSLDWIPGMKNIRLKDFPSFIRTTDINDIMLNYFLIETEAIPKGVAIILNTFDALEKDSITPVLALNPQIYTIGPLHMMQQYVDHDERLKHIGSNLWKEDVSCINWLDTKKPNSVVYVNFGSITVMTKEQLIEFGWGLANSKKDFLWITRPDIVGGNEAMIPPEFIEETKERGMVTSWCSQEEVLKHPSIGVFLTHSGWNSTIESISNGVPMICWPFFAEQQTNCRYCCVEWEIGLEIDTDVKREEVEAQVREMMDGSKGKMMKNNALEWKKKAEEAVSIGGSSYLNFEKLVTDVLLRK

SrUGT76I3

ATGGATACCGATGGAAGAAATACCAGTCTCCAGTCAACACACCCGCCGGAGACTCGCCGGAGAATATTATTCTTCCCCTTACCTTTCCAAGGTCACATCAACCCCATGTTTCAGCTCGCAAACATTCTTCACACCCAAGGTTTCAAAATAACCATCATACACACCCAATTCAACTCACCGGACCATTCAACCTATCCTTACTTCCAATTCCGGTCCATAATCGACCGTTTATCCGAGGTCGAACACCTGTTTCCGACGATCCGAGATCCAAGCTATTTCATCAAGTACCTTAATAAAAACTGTGAGGTTTCGTTTAGGGATTGTTTGATTGGGTTGTTGGATGAGGAACCGGTGGCTTGTTTGATCGCCGATGCGATGTTCTACTTCACGCAGGCGGTTGCCGATGATCTGAAACTACCTCGGTTGGTGCTCCGGACTAGTAGTCTTGGTTGCGCCATTGGTTATGGAGCTTTACCGTTTTCCTCGATCGGAGATGGTGTTAACCTTCCCAAACAAGATCCAGGTTATGAGACACCGGTGCGAGAATATCCACTAATTAAAGTTAAAGACATGTTGAAGGTAGCGGTTAACCCGGAAGGCTACGGTGACCTAGTGACCAACATGCTTAAACAAATGAAAGCGTCATCTGGAATCATATGGAATACGTTCAAAGAACTCGAAGAACCCGAACTAGAAACAATCAATCATGATTTTCGAGTTCCAAACTTCACTTTAGGCCCGTTTCATAACTACTTTCCAGCAACTTCAACAAGCTTGATTGAACAAGACAGAACCATTCTCTCATGGCTCGACACAAAACCTCCCAAATCCGTGATCTATATAAGTTTCGGAAGTGCCGCGCGTATTAAAAGTTTAGAGTTTCAAGAAGTGGCCCACGGGTTGGCAAACGCGAGCTCACCGTTCCTGTGGGTGGTTCGACCAGGGATGGTCCTTGGTTCAGAATGGCTCGAGTCATTGCCAGAAAAGTTTCTAGAAATAGTGGGTGATAGGGGTCGTGTAGTGAAATGGTGTCCTCAACAAGAAGTATTAGCTCATCCAGCAACGGGGTGTTTTTGGACTCATAGTGGATGGAACTCAACATTGGAAAGCATTTGTGAAGGAGTTCCCATGATTTGTTCGCCTTGTTTTGCCGACCAACCAATCATTGCGCGATACGTGAGTGATGTTTGGAAGATCGGTGTTTTGTTGGAAGACGGGTTTGAGCGAGAGGGAATCGCGATCGCGATCAAACGAGTAATGATGGATGAGGAAGGAGAAGAAATATGCGAACGAATAGATCGTCTTAAGGAGAAAGTAAACATCTCACTTGATGAAGGTGGCTCTTCTAATAAGTCGTTAAAGAGTTTAGTTGATTATATTTTGTCGTTATAA

MDTDGRNTSLQSTHPPETRRRILFFPLPFQGHINPMFQLANILHTQGFKITIIHTQFNSPDHSTYPYFQFRSIIDRLSEVEHLFPTIRDPSYFIKYLNKNCEVSFRDCLIGLLDEEPVACLIADAMFYFTQAVADDLKLPRLVLRTSSLGCAIGYGALPFSSIGDGVNLPKQDPGYETPVREYPLIKVKDMLKVAVNPEGYGDLVTNMLKQMKASSGIIWNTFKELEEPELETINHDFRVPNFTLGPFHNYFPATSTSLIEQDRTILSWLDTKPPKSVIYISFGSAARIKSLEFQEVAHGLANASSPFLWVVRPGMVLGSEWLESLPEKFLEIVGDRGRVVKWCPQQEVLAHPATGCFWTHSGWNSTLESICEGVPMICSPCFADQPIIARYVSDVWKIGVLLEDGFEREGIAIAIKRVMMDEEGEEICERIDRLKEKVNISLDEGGSSNKSLKSLVDYILSL

SrUGT91D1-3

ATGGACGACCATAAGCAGCTTCATGTTGCGATGTTCCCATGGCTTGCTTTCGGTCACATTCTCCCATTCTTCGAACTCTCCAAATTCATAACCAAAAACGGTCACAAAGTTTCTTTTCTTTCCCCCACCGGAAACATACAACGTCTCCCTTCTTCTAATCTTTCGCCACTCATGAATCTTGTTAAACTCACACTTCCACGTGTTCAAGAGCTGCCCCAAAATGCATCCGCCACCACGGACCTCCATGCTGATGATGTCCAATATCTCAAACAAGCTTTTGATGGTCTTCAACCGGAGGTCACTCGATTTCTTGAACAAGAGTCGCCGGACTGGATTATTTATGATTTTACTCCCTATTGGTTGCCGGCGGTAGCCACTAGCCTTGGAATTTCACGAGGTTTTTTCTCAATTTTTAACGCATGGACCGTCTCTTTTTTCGGATCTTCTCCCGATGACATCATAAACGGTACAGACGATCGGAAAACAGCCGACGATTTCCTGACACCGCCGAAGTGGTTTCCGTTCCCAAGTAAAGTATGCTACCGGAAGCATGAGGCCAATTTGATATTTGCTGATAATATTTCTGTTAATTCTTCTGGTGTATCAGATTTATATCGTTTAGGAATGGTTATAAAGGGATCAGATTGTATGTTTATAAGACATTGCCATGAGTTCGAACCCCAATGGCTAACCCTTTTAGAAAAGCTGCATCAGCTACCGGTGGTTCCCGTGGGATTATTGCCACCCGAACCACCCACCAGCACCGGAGACCCATGGGTGACAATCAAGAAGTGGCTCGATGGTCAACCAATAGGGCATGTGGTGTACGTGGCATTTGGAAGTGAAGGTACGATGAGCCAAAGCGAGCTGGCTGAGTTAGCTCTGGGTCTCGAGCTCTCTGGGTTGCCATTCTTTTGGGTTCTTAGAAAACCGGTTGGCTCTGGTAACTCGGTGGAGTTGCCAGAGGGGTTCTTGGAACGAACTCGTGACCGTGGGTTGGTGTGGACGAGTTGGGTACCTCAGTTACAAATACTGAGCCATGAGTCGGTGTGTGGTTTCTTGACTCATTCTGGTTGGAGTTCATTTGTGGAAGCGATGATGTTCGGTCACCCTTTGATAATGCTACCGCTTTCGGTGGATCAAGGTCTAAATGCTCGAGTAATGGCGGATAATCAGGTGGGAATTGAGATACCAAGAAATGACGAAGATGGTTCATTCACCAAGGAGTCGGTGGCCAGATCATTGAGGTTGGTTTTAGTCGATGATGAAGGGAAGATCTACAAGGCGAAGGCGATGGAGTTGAGTCAACGATTCGGGGATAGTAAGCCGGAAAATAAGTATATAAACCCTTTTATAGACTATTTGGAACAAAAAGGTCGTGTGGTTGCTATTGAGCATGAGCTTTGA

MDDHKQLHVAMFPWLAFGHILPFFELSKFITKNGHKVSFLSPTGNIQRLPSSNLSPLMNLVKLTLPRVQELPQNASATTDLHADDVQYLKQAFDGLQPEVTRFLEQESPDWIIYDFTPYWLPAVATSLGISRGFFSIFNAWTVSFFGSSPDDIINGTDDRKTADDFLTPPKWFPFPSKVCYRKHEANLIFADNISVNSSGVSDLYRLGMVIKGSDCMFIRHCHEFEPQWLTLLEKLHQLPVVPVGLLPPEPPTSTGDPWVTIKKWLDGQPIGHVVYVAFGSEGTMSQSELAELALGLELSGLPFFWVLRKPVGSGNSVELPEGFLERTRDRGLVWTSWVPQLQILSHESVCGFLTHSGWSSFVEAMMFGHPLIMLPLSVDQGLNARVMADNQVGIEIPRNDEDGSFTKESVARSLRLVLVDDEGKIYKAKAMELSQRFGDSKPENKYINPFIDYLEQKGRVVAIEHEL

SrUGT91D3

ATGGCTAACACCGAGTCCTTGAACGACCATAAGCAGCTTCATGTCGCGATGTTCCCATGGCTTGCTTTTGGTCACATCATCCCTTTCCTTGAACTCTCCAAATTCATAGCTGAAAAGGGTCACAAAGTCTCGTTTCTTTCAACCACTAGAAACATTCAACGTCTCCCTACAATCCCTTCTAACCTCTCGCCACTAATAAATCTTGTTAAACTCACACTTCCACGTGTTCAAGAGCTGCCCGAAGATGCAGAGGCCACTATCGACGTTCACACTCAAGATGTTCATCATCTTAAAAAGGCTTTCGATGGTCTTCAGCCGGAGGTCACTCGGTTTCTTGAAAAAGAGTCTCCAGACTGGATTATTTATGATTTTGCTCCGTATTGGTTGCCGTCCGTCGCAGCTGGACTACGGACCTCGCGAGCCTTTTACTCGAATTTTAACGCATGGTTCATTGCTTTTCTCGGAGCGTCGGCTGACGACTTGATAAGTGGTTCCGGTTATGATCATCGAACGAGGGTTGAGAATTTTATGACACCGCCGAAGTGGGTTCCGTTTCCGACAAACGTGTGCTACCGGAAGTATGAGGCGGTTCGGATGGTAGGGAATACTTCGGCTAATGCTTCTGGAATATCAAGTGTGTATCGTGTGGGAATGATTTTGAAAGGATCTGACTGTATGTTTATAAGACATAGTTATGAGTTCGAACCTCAATGGTTAACCCTTTTAGAGAAGCTCCATCACCTACCGGTGGTTCCTGTCGGATTATTGCCACCAGAAAAACCCACCAACATCGAAGACGGGAATGATAAGACGTGTGACACAGTCAAGAAGTGGCTCGATGGTCAACAAAAAGGTCATGTGGTGTACGTGGCATTTGGAAGTGAGGTTACACTGAGCAGAAGCGAGCTGGCGGAGTTAGCTTTGGGCCTCGAGCTCTCCGGGTTGCCATTCTTTTGGGCTCTTAGAAAACCGGTAGCTTCCACCGAGTCAAAGTTGGTGGAGTTGCCAGACGGGTTCTTGGATCGAACTAGTGACCGTGGGTTGGTGTGGACGAGTTGGGCACCTCAGCTACAAATACTTAGCCATGAGTCAGTGGGTGGTTTCTTGACTCACTGTGGTTGGAGTTCAATTGTGGAAGCGATGATGTTCGGTCACCCTCTAATAATGCTACCGTGTTTGGCGGACCAAGGTCTGAATGCTCGAGTAATGGTGGACAAAAAGGTGGGAATTGAGATACCAAGAAATGGCGAAGATGGCTCCTTCAACAAGGAGTCAGTCGCAAGATCAGTGCGGGCAGTTGTAGCTGATGATGAAGGGAAGATTTACAAGGAAAATGCGATGGAGTTAAGTAGATTGTTCGGAGACACTAAGATGGGAAAGAAGTATATAAACCATTTCATAGATTATTTGGAGAAGACACGACGTACGTTAACCGTATGA

MANTESLNDHKQLHVAMFPWLAFGHIIPFLELSKFIAEKGHKVSFLSTTRNIQRLPTIPSNLSPLINLVKLTLPRVQELPEDAEATIDVHTQDVHHLKKAFDGLQPEVTRFLEKESPDWIIYDFAPYWLPSVAAGLRTSRAFYSNFNAWFIAFLGASADDLISGSGYDHRTRVENFMTPPKWVPFPTNVCYRKYEAVRMVGNTSANASGISSVYRVGMILKGSDCMFIRHSYEFEPQWLTLLEKLHHLPVVPVGLLPPEKPTNIEDGNDKTCDTVKKWLDGQQKGHVVYVAFGSEVTLSRSELAELALGLELSGLPFFWALRKPVASTESKLVELPDGFLDRTSDRGLVWTSWAPQLQILSHESVGGFLTHCGWSSIVEAMMFGHPLIMLPCLADQGLNARVMVDKKVGIEIPRNGEDGSFNKESVARSVRAVVADDEGKIYKENAMELSRLFGDTKMGKKYINHFIDYLEKTRRTLTV

SrUGT85B1-2

ATGGGTTCGGTTCAAGAGAAAAAGGCGCCACATGTTGTGTGCATACCGGCACCACTTCAAGGTCACATTAACCCGATGCTAAAACTAGCCAAAATCCTGCACTCCAAAGGCTTTCTTATCACCTTTGTCAACACCGAGTTTAACCACCAACGGCTCGTTAGGTCACAGGGGGTTGAAGCCCTACACGGGCTCCCAACCTTCCGGTTTGAGACCATCCCAGATGGTCTACCGCCACCTGAAAACAAAGATGCCACCCAAGATATCCCGACTCTAGCCAAGTCGGTTGATGAAAACTTTTTGGGTCCGTTTAAAAGTCTTGTAACCAAAGTGGGTGCTTTGTATGCACCCGTGACTTGTATCGTGTCTGACATGCTTATGTGCTTCACTCTTGATGCCGGTGCTGAATTGGATATCCCGGTGATACTCCATTGGACCAGTGGTACTGGTTCTTTGATATGTTACAATGAATATCCTAATCTATTGGAAAGCAAATTGATGCCCCTCAAAGATGCAAGTTATTTAGTGAATGGTTACTTAGATACGATTGTAGATTCTATCCCCATTTTGCATGGCATACGTTTAAGAGATTTCCCTCCCTTCATTAGAAAGATCTTTCCTGGTGATGAGTTCATGGTTCAATTTTTGACTTCACAAGTAAACAAAGCAAAAAACGGATCTTCTGCTATCATTTTCAACACTTTTGATGAACTAGATCGTGATGTTTTAGACACACTCGCTTCAATGTATCCTCCATGTTATGGAATTGGTCCGTTACATCTACTAGAGAAACATGTTACCGATAAATCTCTTGATTTCGTGAAATCAAACCTTTGGAAAGAAGAACCCGAATGTTTAAAATGGTTAGATACACAAGCTCCATCATCAGTCATTTATGTGAATTTTGGTAGCATTACAGTAATGACACCTCAACAACTAGTCGAGTTTTGTTGGGGACTCGCAAAGAGCAACTATCCGTTCTTATGGATAATACGACCTGACCTTGTGATTGGTGATTCCGCGATGCTTCCACCCGAGTTTGTGAAGGAAACAAGTGATAGAGGGATGCTGGTTGGATGGTGTCCTCAAGAAGAAGTTTTGAATCACCCGTCAATTGGAGGGTTTTTAACGCACAGTGGATGGAATTCAACGCTTGAAAGTATTTCGAGTGGTGTGCCGATGATTTGTTGGCCGTTTTTTGCGGATCAACAAACGAATTGCTGGTGGAGTTGCAACAAATGGGGTGTTTCCATGGAGATTGATAATAATGTAAAGAGTGATGAAGTTTCAAAGCTTGTGATTGAATTAATGGATGGAGAAAAAGGAAAGGAAATTAAAAAGAATGCCATTGACTTGAAGAATAAAGCTGAGGATGCATGTACCTCTCCTCTTGGTTCATCAGTGGTTAATTTGGAGAAAGTGGTTCAACTGATTCATACATTTTCAAAATAA

MGSVQEKKAPHVVCIPAPLQGHINPMLKLAKILHSKGFLITFVNTEFNHQRLVRSQGVEALHGLPTFRFETIPDGLPPPENKDATQDIPTLAKSVDENFLGPFKSLVTKVGALYAPVTCIVSDMLMCFTLDAGAELDIPVILHWTSGTGSLICYNEYPNLLESKLMPLKDASYLVNGYLDTIVDSIPILHGIRLRDFPPFIRKIFPGDEFMVQFLTSQVNKAKNGSSAIIFNTFDELDRDVLDTLASMYPPCYGIGPLHLLEKHVTDKSLDFVKSNLWKEEPECLKWLDTQAPSSVIYVNFGSITVMTPQQLVEFCWGLAKSNYPFLWIIRPDLVIGDSAMLPPEFVKETSDRGMLVGWCPQEEVLNHPSIGGFLTHSGWNSTLESISSGVPMICWPFFADQQTNCWWSCNKWGVSMEIDNNVKSDEVSKLVIELMDGEKGKEIKKNAIDLKNKAEDACTSPLGSSVVNLEKVVQLIHTFSK

SrUGT75E2

ATGACCAAAGTTATGGCTCAAACCCAACCTCACTTCCTCGTATTAACCTTCCCAGCCCAAGGTCACATCAACCCAGCTTTCCAGTTCGCTAAACGACTCATTCGGTTGGGTGTCAAAGTCACCTTCACCACCACCATCTCCGCCTACAATAAAATCACCAAAGTTGGCCGAATTCCAGAGTCTTTCAATCTTGTAGCTTTTTCCGACGGATTTGATGACGGTTACAAGGCCGGAATCGATGACACCACAAAATACATGACCCAGTTAAGGACCGGAGGAATCCAAAGCTTGAAAGAAACCATTCTTTCTAACGCCGAAAAACACACTCCGATCACTTGTTTGGTTTACACCCTCCTCCTTCCATGGGCTGCCAACGTCGCGCATGGGCTCAACGTACCATCGGCTCTACTTTGGATCCAACCTGCGTCCGTTATGCGCGTATACTACTATTACTTCAACGGGTACGATAAACTTATTGGCGACGATTGTAGTGATCCATCATGGTCCATCGAGTTACCAGGGTTACCGTTGCTCAAAAGCCGTGATCTACCGTCGTTTTGTCAACCGACAAACACTTACAACTTCGCATTGCCTTTGTTCAAAGAGCAATTTGATATGTTGGCATCCGAAGACAAGCCTACGATACTCGTTAACAGCTTTGATGCGTTGGAACAAGAGGCGTTGAAGGAGATTGATGGCAAGCTTAGAATGGTCGCGGTTGGACCATTGATTCCTTCGGCTTTCTTGGATGGTAAGGATCCGTCGGACAAAAGTTTTGGTGGAGATTTGTTTGAGACCACGAAAGATTATGTCGAATGGATGAACACAAAGCCTCAAGGGTCCGTTGTGTATATATCTTTTGGTAGCATCATAATGTTGTCAAAGAAACAAAAGGAGGAAATGGCTCATGGTTTGTTAGAAATTGGGAGGCCGTTTTTATGGGTTATTAGAGAGAAAGATGGAAACATGATAGGAGACAAAACCGACGACGAAGAAGACGAATTGATGTGTATGGAGAAGCTTGAACAATTAGGTTTGATAGTCCCATGGTGCAGCCAACTAGAGGTGTTGTCACACCCGTCTTTGGGTTGTTTCGTGAGTCATTGTGGATGGAATTCGACGCTAGAGAGTATCGCATGTGGGGTCCCAGTTGTAGCGTTTCCTCATTGGACGGATCAGTCGACGAACGCGAAGCTTCTAGAAGATGTATGGGGAACAGGGACACGGGTGACCGCGAACGAAGACGGAGTTGTTTTAGGTAATGAGATAAGAAGGTGCATAGAAATGGTGATGGGTGGACATGAAAGAGGGGAAACAATGAGAAAGAATGCTAATAAGTTGAAGGATTTGGCTAGAGAGGCTATGAAAGAGAGTGGATCTTCGTATATGAATCTCAAGGATTTTGTTGAACGAGTTGATGGAAGTTATTCGACTCGTGACAAAGAGGTCATGATCTCGAGTGAAGAAGTGAAAGATCTAGAATCCAAGTTGGAGTCTGTTCTATGA

MTKVMAQTQPHFLVLTFPAQGHINPAFQFAKRLIRLGVKVTFTTTISAYNKITKVGRIPESFNLVAFSDGFDDGYKAGIDDTTKYMTQLRTGGIQSLKETILSNAEKHTPITCLVYTLLLPWAANVAHGLNVPSALLWIQPASVMRVYYYYFNGYDKLIGDDCSDPSWSIELPGLPLLKSRDLPSFCQPTNTYNFALPLFKEQFDMLASEDKPTILVNSFDALEQEALKEIDGKLRMVAVGPLIPSAFLDGKDPSDKSFGGDLFETTKDYVEWMNTKPQGSVVYISFGSIIMLSKKQKEEMAHGLLEIGRPFLWVIREKDGNMIGDKTDDEEDELMCMEKLEQLGLIVPWCSQLEVLSHPSLGCFVSHCGWNSTLESIACGVPVVAFPHWTDQSTNAKLLEDVWGTGTRVTANEDGVVLGNEIRRCIEMVMGGHERGETMRKNANKLKDLAREAMKESGSSYMNLKDFVERVDGSYSTRDKEVMISSEEVKDLESKLESVL

SrUGT85B2

ATGGGTTCGAAACACGAAAACAAACCACATGCATTATGCATACCAGCTCCAGTGCAAGGTCACATTAACCCAATGTTAAAACTAGCCAAAATCCTACATTCCAAAGGCTTCATTATCACCTTCGTCAACACCGAATTCAACCACCAACGTCTCATGAGAGCTCAAGGTTCTGAAGCGGTTCGTGGCCTCCCATCCTTTCAGTTCGAGACCATTCCTGACGGTCTTCCACCGCCAAAAAACCCTGATGCGACACAAGAGGTCCCTTCTCTGGCACGAGCCATTGAAGATAATTTTCTGGGTCCGTTTAAGACCATGGTCACCAAAGTTAGCGCGTCTTATTCACCAGTAACTTGTATAGTAGCCGATGTGCTTATGGGTTTCACCCATGTTGTTGCCTCTGAGTTGGGTATCCCGGTGGTTGTCTTATGGACGAGTGGAGCCGGTTCGTTAATATGTGTACATCAGTATCCTAATCTTTTGGAGAAGGGATTGATGCCCCTTAAAGATTCAAGTTATTTAGTTAATGGTTATTTGGATATGGTTCTTGATTTTATCCCCGCAATGAGTGGAATACGTCTAAAAGACATTCCTCCTTTCATTCGACATATCAACCCTGGCGATGAATACGTCGCTCAATTTCTGTGTTCACAAATAGAGAGAGCAAAAAAAGCTTCCGCTATCATTTTCAACACTTTTGATGAACTCGATCGTGACATTTTAGACACCATCTCTTTAAAGTTCCCTCCCTGTTATGGAATTGGACCTTTAAATCTACTTGAAAACAAGATTGAAGATACAACTCTAGCATGCATCAGATCAAACCTTTGGAAAGAAGAAGACGAATGTTTAAAATGGCTAGACTCAAAAGAAAAATCATCAGTTATCTATGTGAACTTTGGTAGTATCACAGTGATGACATCTCAACAACTAGTCGAGTTTGGATGGGGGCTCGCGAAGAGCCAATATTCATTCTTGTGGATTATACGTCCAGACCTCGTGACTGGTGATGATCAGTCGTCTGCTGGTCTTCCACCTGAGTTGTTGACGGAGATAAGCGATAGAGGGTTGTTGGTAGGTTGGTGTCCCCAAGAACAAGTTCTAAACCACTCGTCAATAGGAGGGTTTTTAACGCATAGTGGATGGAATTCGACAATTGAAAGCATTTCGTGTGGGGTGCCAATGGTTTGTTGGCCGTATTTTGGCGACCAACAAACGAATTGTTGGTTGGCTTGCAACAAGTGGGGTGTTGCGATGGAGATTGACAATAATGTAAAGAGTGATGAGGTTCAGAAGTTGGTGATTGAGTTGATGAAAGGAGAAAAAGGAAATTCTATAAGGAAGAAAGTTGCTGAATTGAAGAACAAAGCTGAGCAAGCATGTGTCTTTCCTTCTGGTTCATCAATAGCTAATTTGGAGAAGATCATTCAACTCATGCAAACTCCATCAATATGA

MGSKHENKPHALCIPAPVQGHINPMLKLAKILHSKGFIITFVNTEFNHQRLMRAQGSEAVRGLPSFQFETIPDGLPPPKNPDATQEVPSLARAIEDNFLGPFKTMVTKVSASYSPVTCIVADVLMGFTHVVASELGIPVVVLWTSGAGSLICVHQYPNLLEKGLMPLKDSSYLVNGYLDMVLDFIPAMSGIRLKDIPPFIRHINPGDEYVAQFLCSQIERAKKASAIIFNTFDELDRDILDTISLKFPPCYGIGPLNLLENKIEDTTLACIRSNLWKEEDECLKWLDSKEKSSVIYVNFGSITVMTSQQLVEFGWGLAKSQYSFLWIIRPDLVTGDDQSSAGLPPELLTEISDRGLLVGWCPQEQVLNHSSIGGFLTHSGWNSTIESISCGVPMVCWPYFGDQQTNCWLACNKWGVAMEIDNNVKSDEVQKLVIELMKGEKGNSIRKKVAELKNKAEQACVFPSGSSIANLEKIIQLMQTPSI

SrUGT91D1-1

ATGTTCCCATGGCTTGCTTTCGGTCACATTCTCCCATTCTTCGAACTCTCCAAATTCATAACCAAAAACGGTCACAAAGTTTCTTTTCTTTCCCCCACCGGAAACATACAACGTCTCCCTTCTTCTAATCTTTCGCCACTCATGAATCTTGTTAAACTCACACTTCCACGTGTTCAAGAGCTGCCCCAAAATGCATCCGCCACCACGGACCTCCATGCTGATGATGTCCAATATCTCAAACAAGCTTTTGATGGTCTTCAACCGGAGGTCACTCGATTTCTTGAACAAGAGTCGCCGGACTGGATTATTTATGATTTTGCTCCCTATTGGTTGCCGGCGGTAGCCACTAGCCTTGGAATTTCACGAGGTTTTTTCTCAATCTATAACGCATGGACCGTCTCTTTTATCGGATCGTCTTCCGATAACATGATAAACGGTACAGACAATCGGAAAACACCCGACGATTTCTTGACACCGCCGAAGTGGGTTCCGTTTCCAAGCAAAGTATGCTACCGGAAGCATGAGGCCAATTCGATATTTGCTGATAATTTTTCTATTAATTCTTCTGGGGTTTCAGATATATATCGTGCTGGAATGGTTATAAAGGGATCAGATTGTATGTTTATAAGACATTGCCATGAGTTCGAACCCCAATGGCTAACCCTTTTAGAAAAGCTGCATCACCTACCAGTGGTTCCCGTGGGATTATTGCCACCCGAACCACCCACCAGCACCGGAGACCCATGGGTGACAATCAAGAAGTGGCTCGATGGTCAACCAATAGGGCATGTGGTGTACGTGGCATTTGGAAGTGAAGGTACGATGAGCCAAAGCGAGCTGGCTGAGTTAGCTCTGGGCCTCGAGCTCTCCGGGTTGCCATTCTTTTGGGTTCTTAGAAAACCGGTTGGCTCTGGTAACTCGGTGGAGTTGCCAGAGGGGTTCTTGGAACGAACTCGTGACCGTGGGTTGGTGTGGACGAGTTGGGTACCTCAGTTACAAATACTGAGCCATGAGTCGGTGTGTGGTTTCTTGACTCATTCTGGTTGGAGTTCATTTGTGGAAGCGATGATGTTCGGTCACCCTTTGATAATGCTACCGCTTTCGGTGGATCAAGGTCTAAATGCTCGAGTAATGGCGGATAATCAGGTGGGAATTGAGATACCAAGAAATGACGAAGATGGTTCATTCACCAAGGAGTCGGTGGCCAGATCATTGAGGTTGGTTTTAGTCGATGATGAAGGGAAGATCTACAAGGCGAAGGCGATGGAGTTGAGTCAACGATTCGGGGATAGTAAGCCGGAAAATAAGTATATAAACCCTTTTATAGACTATTTGGAACAAAAAGGTCGTGTGGTTGCTATTGAGCATGAGCTTTGA

MFPWLAFGHILPFFELSKFITKNGHKVSFLSPTGNIQRLPSSNLSPLMNLVKLTLPRVQELPQNASATTDLHADDVQYLKQAFDGLQPEVTRFLEQESPDWIIYDFAPYWLPAVATSLGISRGFFSIYNAWTVSFIGSSSDNMINGTDNRKTPDDFLTPPKWVPFPSKVCYRKHEANSIFADNFSINSSGVSDIYRAGMVIKGSDCMFIRHCHEFEPQWLTLLEKLHHLPVVPVGLLPPEPPTSTGDPWVTIKKWLDGQPIGHVVYVAFGSEGTMSQSELAELALGLELSGLPFFWVLRKPVGSGNSVELPEGFLERTRDRGLVWTSWVPQLQILSHESVCGFLTHSGWSSFVEAMMFGHPLIMLPLSVDQGLNARVMADNQVGIEIPRNDEDGSFTKESVARSLRLVLVDDEGKIYKAKAMELSQRFGDSKPENKYINPFIDYLEQKGRVVAIEHEL

SrUGT73C5

ATGGCTTTAGATGAGTCACATGAACCCAACCAACTTCACTTTCTTGTTATACCCTTAGGATCTCCAGGCCACTATATCCCAACCATTGATTTAGCCAAATTACTAGCTCAACATGGTGTTAGAGTCACAATAATCACCACACCGGTCAACGCAATCAGATTCGGGTCAATCCTTGATCAAGCAATCAAATCGGGTCTTCCCATTAATTTTCTTGAATTTCAGTTACCATATCTGAAGTTTGGTATTCCAGAAGGTTGTGAATGCATGGATGATCTTCCTAATATTGGATTAGTCAATGATTTTTTTCTTGCCCATAGTTCACTTCAACAAGAAGTTGAACAATATATTGAAAAGCTTGATTCTAAACCAAGTTGCATACTTTCAAGCACATATATTCTTTGGACAGATGAAACATCAAAAAAATTTAAGATTCCAAAGATTGTGTTTGATGGAATGAATTGCTTCACTCAAATGTGTAACCACGTTTTATACCTCTCAAAGGTATATGAAAGTGTCAACGAGTCGGATTATTTTGTTTTGCCCGGGTTGCCTGATCATATTGAACTAACAAGATCGCAACTAGCCTTCGTATTCAATTCAGGGTCCAAAGACGTAAAGGATTATAGGGAAAAGCTTCGGGTATCGGAGTCCGAAGCGTTTGGGATAGTTATAAACAGTTTTCAGGAGTTGGAACAAGAATATGTTGATGCATATCAAAAAGTCAAAGAAGATAAAGCTTGGTGCATAGGGCCATTATCTCTATGCCACAAGGATGCATCCGAGAAGGTCCAAAGAGGTAACAAGTCCTCAATTGACAAAAATGAATGCATCAAGTGGCTAGATTCTCAAGAAAACGAATCGGTGATATACACATGTTTAGGTAGCATTAGTCGCCTTGAGCCTACACAACTCATAGAGCTCGCTTTAGGTCTAGAGTCATCAAAAAAACCATTCATTTGGGTGGTTCGAGCTGGTCATAAGACTGAGAAGATAGAAAAGTGGATAGATGAAGAGGGGTTTGAACAAAGAACCAAAGATAGAGGTCTACTGATCCGCGGGTGGGCTCCACAACTGCTAGTGTTGTCACACCCTGCAATTGGCGCGTTTTTGACTCATTGCGGTTGGAACTCGGCTCTAGAAGGGATATCTGCTGGTGTCCCTATGGTAACATGGCCTCAGTTTCAAGAACAATTTTACAATGAGAAGTTACTTGTACAAGTATTAAAAATTGGCGTCAGTGTTGGCGCGCAAAAAGTTGTGCATTGGGGTGAAGAAGAAAAGTCGGGAGTGGTAGTAAAGAGTGAGGAATTTATAAAGGCTATAGAGCTGGTGATGGAAGATGGGAAAGAAAGTGAAGATAGAAGAAAGAGAGCTAAAGAACTTGGTAAGATGGCTAATGAAGCAATAGAAGATGGAGGATCTTCTCACCGGAATATGACACGATTAATCCAAGATATTAGGAACCTATCATGTGCAAACAATTCAAGCTAA

MALDESHEPNQLHFLVIPLGSPGHYIPTIDLAKLLAQHGVRVTIITTPVNAIRFGSILDQAIKSGLPINFLEFQLPYLKFGIPEGCECMDDLPNIGLVNDFFLAHSSLQQEVEQYIEKLDSKPSCILSSTYILWTDETSKKFKIPKIVFDGMNCFTQMCNHVLYLSKVYESVNESDYFVLPGLPDHIELTRSQLAFVFNSGSKDVKDYREKLRVSESEAFGIVINSFQELEQEYVDAYQKVKEDKAWCIGPLSLCHKDASEKVQRGNKSSIDKNECIKWLDSQENESVIYTCLGSISRLEPTQLIELALGLESSKKPFIWVVRAGHKTEKIEKWIDEEGFEQRTKDRGLLIRGWAPQLLVLSHPAIGAFLTHCGWNSALEGISAGVPMVTWPQFQEQFYNEKLLVQVLKIGVSVGAQKVVHWGEEEKSGVVVKSEEFIKAIELVMEDGKESEDRRKRAKELGKMANEAIEDGGSSHRNMTRLIQDIRNLSCANNSS

SrUGT79A2

ATGTCTTTGAAGGGAAATGATAAAGAACTACACTTAGTAATGTTCCCTTTCTTTGCATTTGGTCACATAACTCCTTTTGTGCAATTATCCAACAAGATATCATCATTATATCCCGGTGTCAAGATTACTTTCTTAGCGGCTTCAGCCAGTGTCAGTCGTATTGAAACCATGCTCAACCCCACCACCAACACCAAAGTCATCCCTCTAACTCTACCACGTGTCGATGGTCTCCCTGAAGGGGTGGAAAACACCGCCGACGCATCACCAGCAACCATTGGACTTCTCATAGTTGCATTAGACCTGATGCAACCCCAGATCAAAACTTTACTAGCTAATCTCAAACCTGATTTCGTAATCTTTGACTTTGTGCATTGGTGGCTACCGGAAATAGCGTCTGAGCTCGGGATAAAAACCATCTATTTCTCTGTCTATATGGCAAATGTCGTCATGCCTTCGACCTCCAAGTTAACCGGCAATAAGCCATCAACTGTAGAAGACATCAAAGCTCTGCAACAATCCGACGGCATACCTGTAAAGACATTTGAAGCTATACGTTTGATGAACGTCTTCAAGAGCTTCCATGGACCACTAAGCTTATCTGATCGGATGGATAAATGCATCAATGGATGCAATCTTATGCTCATCAAGTCATGTAGGGAGATGGAAGGATCGCGCATCGATGACGTCAGGAAACAATCTACGAGACCAGTTTTTCTAATCGGTCCGGTGGTTCCCGAGCCACACTCCGGTGAGTTAGATGAAACATGGGCCAATTGGTTGAACCGGTTCCCGGCTAAATCGGTGATATATTGTTCTTTCGGGAGTGAAACTTTTCTGACCGACGATCAGATTAGGGAATTAGCTTTAGGGTTGGAACTCACCGGACTTCCATTCTTCCTGGTGTTGAATTTTCCGGCGAATGTTGATAAATCTGCAGAACTGAAAAGAACACTACCAGATGGGTTTCTTGAAAGAGTGAAGGATAAAGGGATTGTACACTCAGGGTGGGTGCAACAACGACACATATTGGCGCACGATAGTGTGGGGTGTTATGTGTTTCATGCTGGTTATGGTTCGGTGATTGAAGGTCTGGTTAACGACTGTCAGCTGGTGATGCTGCCAATGAAGGTTGACCAGTTTACGAACTCGAAAGTGATTGCTTTGGAGTTGAAGGCTGGAGTTGAAGTAAACAGGAGAGATGAAGATGGGTATTTTGGGAAAGATGATGTGTTTGAAGCTGTGGAGAGTGTAATGATGGATACTGAAAACGAACCGGCGAAATCCATTAGAGAAAATCATAGAAAACTGAAGGAATTTTTGCAAAATGATGAGATTCAGAAGAAGTATATTGCAGATTTTGTTGAGAACTTGAAGGCTCTTTGA

MSLKGNDKELHLVMFPFFAFGHITPFVQLSNKISSLYPGVKITFLAASASVSRIETMLNPTTNTKVIPLTLPRVDGLPEGVENTADASPATIGLLIVALDLMQPQIKTLLANLKPDFVIFDFVHWWLPEIASELGIKTIYFSVYMANVVMPSTSKLTGNKPSTVEDIKALQQSDGIPVKTFEAIRLMNVFKSFHGPLSLSDRMDKCINGCNLMLIKSCREMEGSRIDDVRKQSTRPVFLIGPVVPEPHSGELDETWANWLNRFPAKSVIYCSFGSETFLTDDQIRELALGLELTGLPFFLVLNFPANVDKSAELKRTLPDGFLERVKDKGIVHSGWVQQRHILAHDSVGCYVFHAGYGSVIEGLVNDCQLVMLPMKVDQFTNSKVIALELKAGVEVNRRDEDGYFGKDDVFEAVESVMMDTENEPAKSIRENHRKLKEFLQNDEIQKKYIADFVENLKAL

SrUGT79A3

ATGACTTTAACCTGCAAACCTAAGGAACTACACTTAGTGATGCTCCCTTTTTTTGCATTTGGTCACATTAGTCCTTTTGTTCAGTTAACCAACAAGTTATCATCGTATCTCGGTGTCAAGATTTCTTTCTTGGCTGCTTCCGCTAGTGTCAGCCGTATTGAAACCATGCTCAACTCTACCACCAACACCAAAGTCATCCCTCTAACTCTACCGCGTGTCGATGGTCTCCCTGAAGGCGTTGAAAACACCTCCGACACCTCACCAGCCACCATCGAGCTTCTCAAAGTCGCATTAGACCTCATGCGACCCCAAATTAAAACCGTACTAGCTAATCTTAAACCTAATTTTGTCTTCTTTGACTTTGCGCAATGGTGGTTGCCCGAAATGGCGTCTGAACTCGGCATAAAAACAGTATTTTTTTCTGTTTTTATGCCGAGCAACTTGGCATTTGTTGCCACGTGGTTCACCCACGATAAGGTCCCAACTATAGAAGTAATGAAGAAGCCTTCATCCGTGTTCCCAGATCTACAACTACCCGAAGCCATAACTTTAAAGACTTTTGAAGCGCTAGATTTCATGTATATCTTCAAGAGCTTTCATGGCACACTAAGTGTATTCGATCGGTTGATGAAATGCTTCAATGGATGCAATGCTGTACTCATCAAGTCATGTAGAGAAATGGAAGCACCGTACATCGACTACCTTAGCAACCAAGTTAAAAAGCCAGTTCTTCTAATCGGTCCGGTGGTTCCTGAGCCACACTCCGGCGAGTTAGATGAAACATGGGCCAACTGGTTGAGCCAATTTCCGGCTAAATCGGTTATATATTGTTCTTTCGGGAGTGAAACGTTTCTAACCGACGATCAAATTAGGGAATTAGCTTTAGGGTTGGAACTCACCGGACTTCCATTCTTCCTGGTGTTGAATTTTCCGGCGAATGTTGATAAATCTGCAGAACTGAAAAGAACACTACCTGATGGGTTTCTTAAAAGAGTGAAGGATAAAGGGATTGTACACTCAGGGTGGGTGCAACAACGACACATTTTGGCGCACGTTAGTGTGGGGTGTTATGTGTTTCATGCTGGTTTTAGCTCAGTGGTTGAAGGTCTGATTAATGATTGTCAGCTGGTGATGCTGCCATTAAAAGGAGATCAACTTACGAATTCTAAAGTGATTGCTTCAGATTGGAAAGCTGGAGTTGAAGTAAACAGGAGAGATGAAGATGGGTATTTTGGGAAAGATGATGTTTTTGAAGCTGTGAAGAGTGTTATGATGGAGACTGAAAAAGAACCAGCAAAATCTATAAGAGAAAATCAAAAGAAATGGAAAGAGTTTTTGCAGAACGATGAGATACATAGCAAGTATATTGCAGATTTTGTTGAGAACTTGAAGGCTCTTTCATAA

MTLTCKPKELHLVMLPFFAFGHISPFVQLTNKLSSYLGVKISFLAASASVSRIETMLNSTTNTKVIPLTLPRVDGLPEGVENTSDTSPATIELLKVALDLMRPQIKTVLANLKPNFVFFDFAQWWLPEMASELGIKTVFFSVFMPSNLAFVATWFTHDKVPTIEVMKKPSSVFPDLQLPEAITLKTFEALDFMYIFKSFHGTLSVFDRLMKCFNGCNAVLIKSCREMEAPYIDYLSNQVKKPVLLIGPVVPEPHSGELDETWANWLSQFPAKSVIYCSFGSETFLTDDQIRELALGLELTGLPFFLVLNFPANVDKSAELKRTLPDGFLKRVKDKGIVHSGWVQQRHILAHVSVGCYVFHAGFSSVVEGLINDCQLVMLPLKGDQLTNSKVIASDWKAGVEVNRRDEDGYFGKDDVFEAVKSVMMETEKEPAKSIRENQKKWKEFLQNDEIHSKYIADFVENLKALS

SrUGT85B3

ATGGATTCGAAACAAGAAAAAAAGCCACATGCATTGTGCATACCAACCCCCGTACAAGGTCACATCAGCCCGATGTTAAAGCTAGCCAAAATCCTTCATTCCAAAGGCTTTCTTATAACCTTCGTCAACACCGAATTTAACCACCAACGTCTAGTGAGAACTCACGGCTCAACCGCCGTCACTGGCCTCCCATCGTTCCGGTTCGAGACCATTCCCGACGGTCTCCCACCGCCACAAAACCTTGATGCGACTCAAGACGTCCCTTCTCTGGCTCGAGCCATTGTTGAAAATTGTGCGGGTCCGTTTAAAACTTTAATCGACAAAGTTAGCGCGTCTTATTCACCGGTAACTTGTATAGTAGCTGATGTGTTTATGGGTTTCACTCACGCTGTTGGCTCTGAGGTTGGTATTCCGGTGTTTGTGTTTTGGACAAGTGGAGCTGGTTCTTTATTATGTTTCGATCGGTATCCATATCTTTTGGAGAAGGGATTGATGCCTCTTAAAGATTCAAGTTATTTAGAAAACGGTTATTTGGATACCGTTCTAGATTGTATGCCTGTCATGCATGGTATACGTCTAAAAGACATTCCTCCTTGGAATAGATATATCAACCCGGGTGATGAATACATGGTTCAGTTTGTCTGTTCACAAGTAGAGAGAGCAAAATTGGCTTATGCAGCTATTTTCAATACTTTTGATGAACTCGATCGTGACATTTTAGACACCATCTCTTCAAAGTTTCCTCCTTGTTATGGGATTGGCCCCTTACATCTACTCGAGAACAAGATTGTAGACAAAACTGTTGCACCCTTCAAATCAAACCTCTGGAAAGAAGATTATGAATGCGTAAGATGGTTAGACTCAAAAGAACCATTGTCGGTTATTTATGTGAACTTTGGTAGTATTACAGTGATGACATCTCAACAACTAGTCGAGTTTGGATGGGGAGTCGCGAAAAGTAATTATTCATTCTTGTGGATAATACGTCCTGACCTTGTAACTGGTGAGTCTGCTGTTCTTCCGCCTGAGTTGTTGGCGGAGATAAGCAACAGAGGATTCTTGGCTGGTTGGTGTCCCCAAGAACAAGTTTTAAACCATCCGTCAATAGGAGGGTTTTTAACGCACAGTGGATGGAATTCGACGATTGAAAGTATTTCTGGTGGAGTCCCAATGATTTCTTGGCCGTTTTTTGGCGACCAACAGCCAAATTGTTGGTTGGGTTGCAACAAATGGGGTATCTCCATGGAGATTGACAATAATGTAAAGAGTGATGAAGTTGAAAAGCTTGTGATTGAGTTGATGAAAGGAGAGAAAGGGAATTCGATGAGGAAGAAGTCTATTGAATTAAAGAAGAAGGCAGAGGAAGCATGCGACTTTCCTTCCGGTTCATCAGTGGCTAATTTGGAGAAGATCATTCATCTCATGAAAACATTTTCAAAGTGA

MDSKQEKKPHALCIPTPVQGHISPMLKLAKILHSKGFLITFVNTEFNHQRLVRTHGSTAVTGLPSFRFETIPDGLPPPQNLDATQDVPSLARAIVENCAGPFKTLIDKVSASYSPVTCIVADVFMGFTHAVGSEVGIPVFVFWTSGAGSLLCFDRYPYLLEKGLMPLKDSSYLENGYLDTVLDCMPVMHGIRLKDIPPWNRYINPGDEYMVQFVCSQVERAKLAYAAIFNTFDELDRDILDTISSKFPPCYGIGPLHLLENKIVDKTVAPFKSNLWKEDYECVRWLDSKEPLSVIYVNFGSITVMTSQQLVEFGWGVAKSNYSFLWIIRPDLVTGESAVLPPELLAEISNRGFLAGWCPQEQVLNHPSIGGFLTHSGWNSTIESISGGVPMISWPFFGDQQPNCWLGCNKWGISMEIDNNVKSDEVEKLVIELMKGEKGNSMRKKSIELKKKAEEACDFPSGSSVANLEKIIHLMKTFSK

SrUGT92C1

ATGCAACCACCACATATAATCATGCTTCCATTCATGGCTCAAGGTCATCTCATACCATTCCTAGAACTAGCTCACAAAATCCTCAACAACAACCCAAATTTCACCATCACCATCGTTAACACCCCCCTCAACATCAATTACCTCCGTTCGACCACCGCCAACCACCCATCTCCGCCGCTTCAACTCCACCTTCAACCTCTCCCATTCACCAGCTCCGATCACCAACTCCCACCCAACTCCGAAAACACCGACGGTTTATCGTTATCCCAAATCATCAAACTCTTGCACGCCTCCGCCGCCCTGGAACCCCCATTCCGCCGGTACATCTCCGACGTTGTTTCCGGCGAAGGGAGCTCGTCGGTGTGTATAGTTTCTGATATACTCATGGGGTGGGCTAATGAAGTGGCGAAATCTTTTAACATTGTTAATTACTCGTTCACCACCGGCGGTGCGTATGGTACGGCGGCGCATTGTTCGATTTGGATGAATCTGCCTCACCGGAATGTAGCTGACGGTGGAACCCACGACGAGTTTCCGGTGCCGTCGTTCCCGGAATCGTGCCGGTTTACGATCACACAACTTCATCAGTATCTTCGAGCTGCTGATGGTGAAGATGAGTGGTCGAGATTCTTTCAACCGCAAATATCGTTCTCTCTTCAATCTAACGGTTGGTTATGTAACACCGTTGAAGAGATTGAAACATTGGGGGGGGAGGTGTTGCTAAATTACATAAAACTCCCTGTTTGGTGTATTGGGCCACTTATCCCATTAAAGATGTTGAAAAAGGATTTGGGTTCGGGTATTATTAGTCCAAGATCCGGAAAGCGACCCGGTATCCAACCCGAAAAATGTATAGAATGGTTAGACTCGCACCCTACCGGGTCGGTGCTTTATATTTCATTTGGGTCTCATAATACGATCAGGGAGACCCAAATGATGGAATTAGCGAAAGGGTTGGAAGAGATCAAGAAACCGTTTATTTGGGTAATTAGACCACCAATAGGGTTCGACCTAAAGGGTGAGTTTCAACGTGAATGGTTGCCTGATGGTTTTGAGGATAGAATTGGAAAACAAGGATTGGTGGTGCATAATTGGGCACCTCAGCTAGAGATATTATGTCATCGGTCAACTGGTGCTTTTTTGAGTCACTGTGGATGGAACTCGGTGATGGAGGGTTTGAGCCAAGGCGTGCCATTGATTGGTTGGCCGATAGCGGCAGAACAAGGGTATAATGCGAAGATGTTGGTTGAGGATGCGGGTGTTTGTGTGGTGTTAGCGCGAGGGGTGCATTGTAGGATTACGAAAGAAGAGGTCAGTAGTGTGATCAATGAGGTGTTGGATAAAAGTGAAGGTGGAAAAGGCGAAGATATAAGGAAAAAAGCAGGCGAATTAGGGAAATTGATAAGAGCAAGTGTTGAGAATGACAAGGGTTCTTCATTCATCGCGATGAATGATTTCTTGACCACCGTTCTTTCTGGATTTAAATGA

MQPPHIIMLPFMAQGHLIPFLELAHKILNNNPNFTITIVNTPLNINYLRSTTANHPSPPLQLHLQPLPFTSSDHQLPPNSENTDGLSLSQIIKLLHASAALEPPFRRYISDVVSGEGSSSVCIVSDILMGWANEVAKSFNIVNYSFTTGGAYGTAAHCSIWMNLPHRNVADGGTHDEFPVPSFPESCRFTITQLHQYLRAADGEDEWSRFFQPQISFSLQSNGWLCNTVEEIETLGGEVLLNYIKLPVWCIGPLIPLKMLKKDLGSGIISPRSGKRPGIQPEKCIEWLDSHPTGSVLYISFGSHNTIRETQMMELAKGLEEIKKPFIWVIRPPIGFDLKGEFQREWLPDGFEDRIGKQGLVVHNWAPQLEILCHRSTGAFLSHCGWNSVMEGLSQGVPLIGWPIAAEQGYNAKMLVEDAGVCVVLARGVHCRITKEEVSSVINEVLDKSEGGKGEDIRKKAGELGKLIRASVENDKGSSFIAMNDFLTTVLSGFK

SrUGT78D2

ATGGATACCACCAAAAGCTCCAAAAACGGCAACAAAAAAGAAAAACATGTAGCCGTTTTTGCATTACCATTTGCATCACACCCTGCTCTCCTTCTAGCCGTCACCCGGCGGCTAGCTTCCGCCGCACCGAATGTGGTGTTCTCCTTTTTCAACACCGAACCATCTAACCGACTTTTGTTCTCTGAACTCAATTGCGACAATATATTGCCGAATAATATATCGAATGGTACACCGGAGGGTTTTGTTTTCTCCGGAAACCATGATGAGGGTATGAACTTGTTTCTCGCGGTGGCGAATGAAGAATATAGGAACGATGTTCGTGCGGCGGAGATGGATACCGGGTTGAAGATTAGTTGTCTTGTGGTGGATGCGTTTCTTTGGTTTACAGCTGATATTGCCGAAGAAATGAATATTTCTTGGGTGCCGTTTTGGCCCGGTGGAACTTGTTCTTTTGCGGCACATCTTTATACCGATCTCATCAGAGAAAAATGTGCTCAACTCATAGGTTCTACTCGACAGGACGAAGTTGTCGACTTGGTCCCAGGACTCAAATCGATTCAACTACGTGATTTACCTGACGGGGTCGTATTGGGAGACCTCGAATCACCATGGGCAATCTTACCACTTAAAATGGGAAGAACCTTAACCCGAGCAACCGCGGTTCCCGCCAGTTCTTTCCAAGAACTAGAGGAAGAACTAGACCCCGATGTTACAAAAAACATTTCGTCAAAGTTGAACAATTTCCTCACCATTGGTCCCTTAAATCTCACATTCGAAGAAACAAAACCATCAAAATCTGATGAATACTCTTGCATTTCATGGTTAGATACTCAAAAGATAAGATCGGTTGCTTACATTTGCTTTGGTAGGGTTTGCACGCCTCCGCCTCACGAGCTAGTCGAGCTGGCTGAAGCACTCGAAGAGACCAAAACCGTGTTTCTTTGGTCGATAAACAAAGATGCCACTAAGCATTTGCCTAACGGTTTCTTGGAAAGAATACACGCCAACGGGACAGGGAAAGTTGTCCCGTGGGCCCCACAGGAGGAGGTCTTGAACCATATTGCGGTTCGGGTGTTTGTAGTGCATGGTGGTTGGAATTCGGTGTTGGAGAGTGTAGGAGCCGGTGTCCCGATGATATGTAGGCCGTTTTTCGGGGATCATCATATAAATTCTTGGATGGTTGAGAGGTTGGGAACCGGTTTGAGAATTAAAGGCGGGAGTTTCACAAAACATGCCACTTGTCACGCTTTGGAGCAGGTTTTCTCGGATTCGAAACTCCGGGATGAGAGAGTTAAAGTATTGAAAGATCTTGCTCATAAGACTGTTGCACCGAATGGTAGCTCTAATAAGAATTTCAAGACTTTGGTAGAGGTGGTCACGGGTACTGCCACTTGA

MDTTKSSKNGNKKEKHVAVFALPFASHPALLLAVTRRLASAAPNVVFSFFNTEPSNRLLFSELNCDNILPNNISNGTPEGFVFSGNHDEGMNLFLAVANEEYRNDVRAAEMDTGLKISCLVVDAFLWFTADIAEEMNISWVPFWPGGTCSFAAHLYTDLIREKCAQLIGSTRQDEVVDLVPGLKSIQLRDLPDGVVLGDLESPWAILPLKMGRTLTRATAVPASSFQELEEELDPDVTKNISSKLNNFLTIGPLNLTFEETKPSKSDEYSCISWLDTQKIRSVAYICFGRVCTPPPHELVELAEALEETKTVFLWSINKDATKHLPNGFLERIHANGTGKVVPWAPQEEVLNHIAVRVFVVHGGWNSVLESVGAGVPMICRPFFGDHHINSWMVERLGTGLRIKGGSFTKHATCHALEQVFSDSKLRDERVKVLKDLAHKTVAPNGSSNKNFKTLVEVVTGTAT

SrUGT91D4

ATGGCTACCACCGAAAACCGTAAACAGCTTCATGTTGCGATAATCCCATGGCTTGCTTTCGGTCACATTCTTCCTTTCCTTGAACTTTCCAAATATATAGCACAAATTGGTCACAAAGTCTCTTTTCTTTCGACCACTAGAAACATCCAACGTCTACCAACTCTCCCTTTTCACCTCTCACACCTCATTAATCTTGTTAAACTCACACTTCCACGTGTTCACGAACTGCCGGAGGACGCAGAGGCGACCACGGACGTCCACACTCATGATCTTCGTCACCTAATTAAAGCTTTTGACGGACTTCAGCCGGAGGTCACCGGGTTTCTTGAGCAAGAGTCTCCAGACTGGATTATTTATGATTTTACTCACCACTGGTTGCCATCCGTCGCGGCGGACCTTGGGATCTCGCGAGCCTTTTTTATCACTGCCTCACCATGGTTCATGGCGCACGTGGGACCGTTACCCGATGACATGAAAAATAGTTTCGAGAGTCGAACGACAGTTGAGGATTTCATGACACCGCCCAAGTGGATTCCGTTTCCGAGCAAAGTATGCTACCGGAAGCATGAGTTTGTGCGCAAGATGTCTGCTGCTAATAATCCTGTGATTTCAGGTCTAAAGCACAGAATGATGACGGTTTTGAAGGAATCTGATTGTCTGTTTATAAGAAGTTGTTATGAGGTCGAACCCCAATGGCTAACCCTTTTGGGGAAGCTATACCGTATACCGGTAGTTCCGGTTGGATTAATGCCACCTGAAACATACGGAGACGAGAAAGATGATACATGGGTGTCAGTCAAGAAGTGGCTCGATGGTCAGCAAGTTAACCATGTGGTGTACGTCGCATTAGGAAGTGAAGTTACGGTGACCAAAAGCGAGCTGGCTGAGTTAGCGTTAGGTCTCGAGCTCTCTGGGTTGCCATTTTTGTGGGTTCTCAGAAAACCCGCAGGTTCCACCGAGTCAGACTCGGTGGAGTTGCCTAACGGGTTCTTGGAACGAACTAGTAACCGTGGGATGGTTTGGACGAGTTGGGCACCTCAGTTACGAATACTGAGTCATGAGTCGGTGTGTGGTTTCTTAACTCACTGTGGTTGGAGTTCAGTGGTGGAAGGGTTAATGTTCGGCCACCCTTTAATCATGCTACCGATTTTCGGGGACCAATGTCTGAATGCTCGACTATTAGCGGACAAACAGGTGGGGATCGAGATACCAAGAAATGAGGAAGATGGCTACTTCACCAAGGAGTCGGTTGCCACGTCACTGAGGTCGGTTGTTGTAGACGATGAAGGAGAGATCTACAAGGCGAATGCGAGAGAGATGAGTAAAATATTCAGCGACACTAAGCTGCAAAAGGAATATATAAGTCGTTTCACAGACTATATGGAAAAGAACGTACGTGTGGTTGCCAACAACCATGAGAGTTAA

MATTENRKQLHVAIIPWLAFGHILPFLELSKYIAQIGHKVSFLSTTRNIQRLPTLPFHLSHLINLVKLTLPRVHELPEDAEATTDVHTHDLRHLIKAFDGLQPEVTGFLEQESPDWIIYDFTHHWLPSVAADLGISRAFFITASPWFMAHVGPLPDDMKNSFESRTTVEDFMTPPKWIPFPSKVCYRKHEFVRKMSAANNPVISGLKHRMMTVLKESDCLFIRSCYEVEPQWLTLLGKLYRIPVVPVGLMPPETYGDEKDDTWVSVKKWLDGQQVNHVVYVALGSEVTVTKSELAELALGLELSGLPFLWVLRKPAGSTESDSVELPNGFLERTSNRGMVWTSWAPQLRILSHESVCGFLTHCGWSSVVEGLMFGHPLIMLPIFGDQCLNARLLADKQVGIEIPRNEEDGYFTKESVATSLRSVVVDDEGEIYKANAREMSKIFSDTKLQKEYISRFTDYMEKNVRVVANNHES

SrUGT85C3-1

ATGGATGCAGTGGTCGAAACAGAAAAGAAGAAACCACACGTCATCTGTATGCCGTTTCCAGATCTAAGCCACATAAAAGCCACGCTCAAACTAGCCGAGCTTCTCTACCACAAAGGACTCCAGATAACCTTCATCAACACCGATTTCGTCCACAACCGGTTTCTTGAATCAGGAGGACCACACTGTTTAGACGGTGCACCTGGTTTCCGGTTTGAAACCATTCCAGATGGTGTTACTCGCAGATCGGAAGCTAGCATCCACAACACCAGAGAACTACTCAAGTCCGTCGAAACCATCTTCTTGGATCGTTTCATTGACCTTGTAACCAAACTTCCGGATCCTCCGACGTGTATTATCTGTGATGGTTTCTTGTCGGTTTTCACAATTGACGCTGCAAAAAAGCTTGGAATCCCGGTTATGATGTATTGGTCACTTGCTGCCTGTGGCTTCATGGGGTTTTATCAAATGCATTCTCTCATTGAGAAAGGATTTGCACCGCTTAAAGATGAAAGTTATTTGACAAATGGGTATTTAGACACCGTCGTTGATTGGGTTCCGGGAATGGAAAGCATCCGTCTTAAGGAGTTCCCATTGGTTTGGAACACTGACTTGAATGATAAACTTCTAACATTCTGCAAGGAAGCTCCCCAAAGGTGTCACTCGGTTTCAAATCATATTTTCCACACGTTTGATGAGTTAGAGCCTAGTGTTATCAAAGCTTTGTCATCTATATATTCTCGCGTTTACGCCATCGGCCCACTACAACTACTTCTTGATCAGATACCTGAAGAAAAAAAGCAAACTGGAATTTCAGGTTTGAATGGATACAGTTTTGTGAAAGAAGAACCCGAGTGTTTTAAGTGGCTTCAATCTAAGGAACCATATTCTGTCATTTATGTAAATTTTGGGAGTTCAACATTAATGTCTTTAGAAGACTTGATAGAATTTGGTTGGGGACTTGTTAATAGCAACCACTATTTCCTTTGGATCATCCGATCTAACTTGATAAATGGAGAATCTGCAGTTTTGCCTCTTGAATTAGACGAACATATAGAAAAGAGAGGCTTTATTGCAAGCTGGTGTTCACAAGAAAAGGTCTTGAACCACCCTTCCATTGGAGGGTTCTTGACTCATTGTGGGTGGGGATCCACAATCGAGAGCTTGTCAGCTGGGGTGCCAATGATATGTTGGCCTTTTTTTTGGGACCAACCGACCAACTGTAGGTATATATGCAATGAATGGGAAGTTGGACTTGAGATGGGGAATAAAGTGAAACGAGATGGAGTCAAGAGACTTGTACAAGAGTTAATGGGAGAAGGTGGTCACAAAATGAGAAACAAGGCCAAAGAATGGAAGGAAAAGGCTCGTATTGCCACATGCCCTAACGGTTCATCTTCTTTGAATGTGGATAAAATTGTCGAGGAGATAACGGTGCTATCAAGAGACTAG

MDAVVETEKKKPHVICMPFPDLSHIKATLKLAELLYHKGLQITFINTDFVHNRFLESGGPHCLDGAPGFRFETIPDGVTRRSEASIHNTRELLKSVETIFLDRFIDLVTKLPDPPTCIICDGFLSVFTIDAAKKLGIPVMMYWSLAACGFMGFYQMHSLIEKGFAPLKDESYLTNGYLDTVVDWVPGMESIRLKEFPLVWNTDLNDKLLTFCKEAPQRCHSVSNHIFHTFDELEPSVIKALSSIYSRVYAIGPLQLLLDQIPEEKKQTGISGLNGYSFVKEEPECFKWLQSKEPYSVIYVNFGSSTLMSLEDLIEFGWGLVNSNHYFLWIIRSNLINGESAVLPLELDEHIEKRGFIASWCSQEKVLNHPSIGGFLTHCGWGSTIESLSAGVPMICWPFFWDQPTNCRYICNEWEVGLEMGNKVKRDGVKRLVQELMGEGGHKMRNKAKEWKEKARIATCPNGSSSLNVDKIVEEITVLSRDSrUGT85C4

ATGGATGCGGAGAAGAAACCGCACGTCATATGCATACCTTGTCCAGGACAAAGCCACATAAAAGCCATGCTCAAACTAGCCGAGCTACTCCACCACAAGGGACTCCAAATAACCTTCGTCATCACCAATTTTGTCCACAACCAATTTCTTGAATCAGCGGGCCCACACTGCCTAGACGGGGCACCCGGGTTCCGGTTTGAAACCATTCCCGATGGTGTTTCTCGAAGTTTGGAAACCAGCATTGACACCACAAGAGAATTACTTTTGCGATCCATTGAAACCAACTTCTTGGCTCCTTTTGTTGACCTTGTAACCAAACTACCGGATTCTCCGACTTGTATTATCTCAGATGGGTACATGTCGGCTTTCACAATCGAGGCTGCACATAAGCTTGGAATCTCAGTCATGATGTATTGGACAGTTGCTGCTTGTGGCTTCATGGGGTGTTACCAAATCCAATCCCTCATCGATAGAGGATTTGCACCGCTTAAAGACAAAAGTTATTTGACAAATGGGTATTTAGACACCGTCGTTGATTGGGTTCCAGGAATGAATGGCATTCGTCTTAAGGATTTTCCAATGACTTGGACCACTGACATAAATGACAAAGTTTTAAAGTTTAGTACAGAAGCTCCCCGAAGGTCTCTCACGGTTTCACATCATATTTTCCACACGTTCGACGAGTTGGAGCCTAGTATTATCAAAGCTTTGTCATCGATGTATGCTAACGTGTACACCATCGGCCCACTACAACTACTTCTCGATCAAATACCTGAAGAACAAAAGCAAAATGGAATTTCGGGTATCAATAGATACAGTCTAGTGAAAGAAGAACCAGAATGTTTCAAGTGGCTTGAATCTAAGGAACCAAATTCTGTCATTTATGTAAATTTTGGGAGTACAACACTAATGTCTTCAGAAGACCTTATAGAATTTGCTTGGGGACTTGCTAATAGCAACCACTATTTCCTTTGGATAATCCGATCTTACTTGGTGAATGGGGAATCTATAGTTCTACCTCTTGAATTTGAGGAACATATAAAAAACAGAGGTTTTATCGCAAGCTGGTGTTCACAAGAAAAGGTCTTGAACCACCCTTCGGTCGGAGGGTTTTTGACTCATTGTGGGTGGGGATCCACCATTGAGAGCTTGTCGGCAGGGGTGCCGATGATATGTTGGCCATATTTGTGGGACCAGCTGACCAACTGTAGGTATATATGCAATGAATGGGAGGTTGGACTAGAGATGGGGAATAAAGTGAACCGCGATGAAGTCAAGATGCTTGTACAAGACTTGATGGGAGAAAGAGGTCAAAAAATGAGAAACAAAGCCATAGAATGGAAGGAAAAGGCTCGTATTGCAACAAGTCCCCACGGTTCATCTTCTTTGAACATTGACAAAATTGTGAAGGAAATCACCATGCTATCAAGAGACTAG

MDAEKKPHVICIPCPGQSHIKAMLKLAELLHHKGLQITFVITNFVHNQFLESAGPHCLDGAPGFRFETIPDGVSRSLETSIDTTRELLLRSIETNFLAPFVDLVTKLPDSPTCIISDGYMSAFTIEAAHKLGISVMMYWTVAACGFMGCYQIQSLIDRGFAPLKDKSYLTNGYLDTVVDWVPGMNGIRLKDFPMTWTTDINDKVLKFSTEAPRRSLTVSHHIFHTFDELEPSIIKALSSMYANVYTIGPLQLLLDQIPEEQKQNGISGINRYSLVKEEPECFKWLESKEPNSVIYVNFGSTTLMSSEDLIEFAWGLANSNHYFLWIIRSYLVNGESIVLPLEFEEHIKNRGFIASWCSQEKVLNHPSVGGFLTHCGWGSTIESLSAGVPMICWPYLWDQLTNCRYICNEWEVGLEMGNKVNRDEVKMLVQDLMGERGQKMRNKAIEWKEKARIATSPHGSSSLNIDKIVKEITMLSRD

SrUGT76H2

ATGGGAGATGAGAGAAACCAAACCAAACATTTGGTTCTTGTAGCAAGTCCATTTCAAGGTCATATGACTCCTATGCTTCAACTAGGTAGTGTTCTTCATTCCAAAGGCTTCTCAATCACCATCGCTCACACCAAATTAAACTCACCCGATCCTTCAAACCATCCCGAATTCGTCTTTCTTCCGCTTTTCGACAACTTTTTAGCCATTGATGCATCCGCCAACTTCACCAAGTTTCTTGAAATTCTTAACAATAACTGCAAAAAGCAACTACAAGATCGCTTGACTCAACAGATTCAACAACGGAAAGGAGTAAACGATGATAAGAACGAGAAGATTACAATTATTCATGATAATATTATGTATTTTGCGGAAGAAGTTGCAAGAAATTTGAATCTTTCGAGTATTGTTTTAAGAAGTTGTAGTGCGTCGTATATGCCAGCTTTTCTTGCTCTTGATAAACTTAATGCAGAAGGACATCTTCCTGTGCAAGAATCTATGCTAGAGAAGCTTGTACCAGAACTCTATCCTCTCAGATACAAAGATTTACCTTTCAACAACACATCAACAGAAGTATTAAAAGAAATTCTTGCACTTTCGGATTGTATTAGAACACCATCAGCCATAATTTGGAACACAATGGATTTTCTTGAGAATTCATCACTTACAATACTTAAGAAGTATTATGAGGCCCCAATCTTCACCATCGGCCCGTTGAATGAATTGGCTCAATGCCCGTCTACAAGCTTTCTCAAAGAAGACACCGATTGCATATCCTGGCTTGATAAGCAAGCTCCAAGATCTGTTATATATGTAAGTTTAGGGAGTTTAGCAACCATGGATGAACATGAGCTAGCCGAGACTGCATGGGGCTTAGCCGATAGTAACCAACCGTTCCTATGGGTGGTTAGACCTGGTTCAGTCAAAGGGTCGGATTGGATTGAGTTTTTGCCAGATGGGTTCGAAGAAAAAGTAAGAGGAAGAGGTCTCGTGTTGAAATGGGCCCCACAAAAGCAAGTTTTGGCTCATTTTGCCGTTGGAGGGTTTTGGAGCCATTGTGGTTGGAACTCGAGTTTGGAGAGTATTTCACAAGGGGTCCCGATGATATGTCGGCCATTTTTGGGGGACCAATATGTAAATGCAAGGTATTTAAGTTTCGTGTGGCGGGTGGGTTTGGAACTCGAATATTTGGAGAGACATGTGATTGTAAGTGCAATTAGAAGACTATTGGTGGATGATGAAGGAGAAGAGACGAGGAAACGTGCGAATAATATGAAAGAAAAGGCTAAACATTGTTTGTGCAAAGGCGGATCTTCAGTCAGTTCCTTGAATAACTTGGTAGAATTCATCCTTGGAGCTCGAGTTTTACAAAGTGTTACAACTTAG

MGDERNQTKHLVLVASPFQGHMTPMLQLGSVLHSKGFSITIAHTKLNSPDPSNHPEFVFLPLFDNFLAIDASANFTKFLEILNNNCKKQLQDRLTQQIQQRKGVNDDKNEKITIIHDNIMYFAEEVARNLNLSSIVLRSCSASYMPAFLALDKLNAEGHLPVQESMLEKLVPELYPLRYKDLPFNNTSTEVLKEILALSDCIRTPSAIIWNTMDFLENSSLTILKKYYEAPIFTIGPLNELAQCPSTSFLKEDTDCISWLDKQAPRSVIYVSLGSLATMDEHELAETAWGLADSNQPFLWVVRPGSVKGSDWIEFLPDGFEEKVRGRGLVLKWAPQKQVLAHFAVGGFWSHCGWNSSLESISQGVPMICRPFLGDQYVNARYLSFVWRVGLELEYLERHVIVSAIRRLLVDDEGEETRKRANNMKEKAKHCLCKGGSSVSSLNNLVEFILGARVLQSVTT

SrUGT88B2

ATGGGCCACCTGGTTTCCATGGTGGAACTCGCAAAACAAATCCAAATTCACAACCCTTCACTCTCTGTTATCGTCTTCATAACCCCGACACCTTTTGAAACTGAAAACTACATCAACATCGTCTCCACCGCCAACCCATCAATCAAGTTCCACCGTCTCCCCGATGATGTTACTCTTCCACCAGACTTCTCTTCAGATTTCATAGACCGTGCTTTTGGGATCCCGGAGCTTTACAACCCAATTGTCCACAACGCCCTCGTAAACATCCGAAACGAATCAACCATCAAAGCTGTGATCCTTGATTTCGTTTCAAATGCGGCTTTCCGAGTCTGTAAGTCTCTAAACCTTCCGACTTACTACTTTTATACAAGTGGTGCTTCTGGCCTTTGTGAGTTCTTATACCTCACTACCATTCACAACACCACTTCTAAGAACATCGGAGATCTCGACGTTTACTTTGATATTCCTGGAGTACCTCCAATTCATGCATCTGATATGCCCCCGGTTATGTTTGATAGAGAAACCAACATGTACAAAAACTTCATACACACTGCAAGTAACATGGCAAAATCTTCGGGTTTGATTGCAAATAGTTTTGCGGGGTTTGAAGAAAGAGCCACCGACGCTCTTCGAAATGGTAAATGCGTCACCGACGGTCCAACTCCACCACTTTATCTAATCGGGCCTTTGATTGCAAGTGACATTCAAGTGGATCCTAGTGAAAATGAGTGCCTAAAATGGTTGAACACACAGCCATCTACAAGTGTAGTGTTTTTATGTTTTGGAAGTATGGGTGTGTTCAAGAAACAGCAATTGAAAGAAATAGCTATTGGATTGATGAAGAGCGAGCAAAGATTTTTGTGGGTGGTGCGCGATCCACCACCAGATGATGAAAACGATTCGGGTTCAGTGGCTAATAATTACGGGCTCGATGCTATTCTTACTGAAGAGTTTGTGGCACGAACTGCGGGTAGGGGTTTGGTGGTCAAAAACTGGGCACCGCAGCCGGCTATACTGAGTCATGATTCAGTGGGTGGGTTTGTGAGTCATTGTGGGTGGAACTCGACGCTTGAAGCGGTGGTTGCTGGGGTTCCAATGGTGGCATGGCCATTGTATGCAGAGCAAAAGATGAACAGGGTGTTTTTGGTTGAAGAAATGAAGGTGGCAGTGGCGGTGAAAATGTCGGTGGATGGGTTTGTGACGGCGGATGCGGTGGAGGAGAAAGTGAGGGATTTAATGGAGGGTAAGGAAGGGAGAGTGGTGAGAGAACGGGTTTTAGAGATGAGTCGATTGGCAAAGGCGGCTGTGGAGGACGGCGGCTCTTCGCAACTTGATTTCTTTAAATTAACCAACTCCTGGACAAACATGTAA

MGHLVSMVELAKQIQIHNPSLSVIVFITPTPFETENYINIVSTANPSIKFHRLPDDVTLPPDFSSDFIDRAFGIPELYNPIVHNALVNIRNESTIKAVILDFVSNAAFRVCKSLNLPTYYFYTSGASGLCEFLYLTTIHNTTSKNIGDLDVYFDIPGVPPIHASDMPPVMFDRETNMYKNFIHTASNMAKSSGLIANSFAGFEERATDALRNGKCVTDGPTPPLYLIGPLIASDIQVDPSENECLKWLNTQPSTSVVFLCFGSMGVFKKQQLKEIAIGLMKSEQRFLWVVRDPPPDDENDSGSVANNYGLDAILTEEFVARTAGRGLVVKNWAPQPAILSHDSVGGFVSHCGWNSTLEAVVAGVPMVAWPLYAEQKMNRVFLVEEMKVAVAVKMSVDGFVTADAVEEKVRDLMEGKEGRVVRERVLEMSRLAKAAVEDGGSSQLDFFKLTNSWTNM

SrUGT91D2

ATGGCTACCAGTGACTCCATAGTTGACGACCGTAAGCAGCTTCATGTTGCGACGTTCCCATGGCTTGCTTTCGGTCACATCCTCCCTTACCTTCAGCTTTCGAAATTGATAGCTGAAAAGGGTCACAAAGTCTCGTTTCTTTCTACCACCAGAAACATTCAACGTCTCTCTTCTCATATCTCGCCACTCATAAATGTTGT

TCAACTCACACTTCCACGTGTCCAAGAGCTGCCGGAGGATGCAGAGGCGACCACTGACGTCCACCCTGAAGATATTCCATATCTCAAGAAGGCTTCTGATGGTCTTCAACCGGAGGTCACCCGGTTTCTAGAACAACACTCTCCGGACTGGATTATTTATGATTATACTCACTACTGGTTGCCATCCATCGCGGCTAGCCTCGGTATCTCACGAGCCCACTTCTCCGTCACCACTCCATGGGCCATTGCTTATATGGGACCCTCAGCTGACGCCATGATAAATGGTTCAGATGGTCGAACCACGGTTGAGGATCTCACGACACCGCCCAAGTGGTTTCCCTTTCCGACCAAAGTATGCTGGCGGAAGCATGATCTTGCCCGACTGGTGCCTTACAAAGCTCCGGGGATATCTGATGGATACCGTATGGGGCTGGTTCTTAAGGGATCTGATTGTTTGCTTTCCAAATGTTACCATGAGTTTGGAACTCAATGGCTACCTCTTTTGGAGACACTACACCAAGTACCGGTGGTTCCGGTGGGATTACTGCCACCGGAAATACCCGGAGACGAGAAAGATGAAACATGGGTGTCAATCAAGAAATGGCTCGATGGTAAACAAAAAGGCAGTGTGGTGTACGTTGCATTAGGAAGCGAGGTTTTGGTGAGCCAAACCGAGGTTGTTGAGTTAGCATTGGGTCTCGAGCTTTCTGGGTTGCCATTTGTTTGGGCTTATAGAAAACCAAAAGGTCCCGCGAAGTCAGACTCGGTGGAGTTGCCAGACGGGTTCGTGGAACGAACTCGTGACCGTGGGTTGGTCTGGACGAGTTGGGCACCTCAGTTACGAATACTGAGCCATGAGTCGGTTTGTGGTTTCTTGACTCATTGTGGTTCTGGATCAATTGTGGAAGGGCTAATGTTTGGTCACCCTCTAATCATGCTACCGATTTTTGGGGACCAACCTCTGAATGCTCGATTACTGGAGGACAAACAGGTGGGAATCGAGATACCAAGAAATGAGGAAGATGGTTGCTTGACCAAGGAGTCGGTTGCTAGATCACTGAGGTCCGTTGTTGTGGAAAAAGAAGGGGAGATCTACAAGGCGAACGCGAGGGAGCTGAGTAAAATCTATAACGACACTAAGGTTGAAAAAGAATATGTAAGCCAATTCGTAGACTATTTGGAAAAGAATGCGCGTGCGGTTGCCATCGATCATGAGAGTTAA

MATSDSIVDDRKQLHVATFPWLAFGHILPYLQLSKLIAEKGHKVSFLSTTRNIQRLSSHISPLINVVQLTLPRVQELPEDAEATTDVHPEDIPYLKKASDGLQPEVTRFLEQHSPDWIIYDYTHYWLPSIAASLGISRAHFSVTTPWAIAYMGPSADAMINGSDGRTTVEDLTTPPKWFPFPTKVCWRKHDLARLVPYKAPGISDGYRMGLVLKGSDCLLSKCYHEFGTQWLPLLETLHQVPVVPVGLLPPEIPGDEKDETWVSIKKWLDGKQKGSVVYVALGSEVLVSQTEVVELALGLELSGLPFVWAYRKPKGPAKSDSVELPDGFVERTRDRGLVWTSWAPQLRILSHESVCGFLTHCGSGSIVEGLMFGHPLIMLPIFGDQPLNARLLEDKQVGIEIPRNEEDGCLTKESVARSLRSVVVEKEGEIYKANARELSKIYNDTKVEKEYVSQFVDYLEKNARAVAIDHES

SrUGT83C1

ATGAAAACTATTCATGTTCTAGCAATACCCTACCCAGCACAAGGCCATGTGATACCACTTATGGAGGTAGCACGGTGCTTGACCAACTACTGTCTCAAAGTCACGTTTGTGAACTCAGAGTTTACAAACAAACAGATTATGAGTTCTGGTTCATCGATAGATGTTCCGAGTGATTTATTGCAAATGGTTTCAATCCCAGATGGGATTGAAACATTTGAAGATAGGAACAACCTTGGGAAGTTGTCTGAAACAATGTTCCAATTCATGCCCTCCAAACTAGAAGAGCTAATAAATGATATCAACAAAAATGAAGACGAGAGAATTGTGTGCATCATTGCGGACTATTGCATGGGATGGGTCAAAAGTGTTGCACAGAAGATGGGTATTAGACTAGCAGTCTTTTGTCCCACCTCAGCAGCCGTTTTGGCCGTGACCATGAGTATTCGAAAGCTGATGGACGATGAAATTCTAAACTGCAAAGGTGAGGCAACTTCAAAGAATCAAATGGTTCAATTGTCAGCATCCATGCCACTCATGGACCCTGCACACTTTGTATGGGTGTGTATGGGGGACCCAATAGTAAACCAAATTTTCTTTGATTACCTATTTTATAAAGGTATGGAAGCAGCAGAAGCAGCGGATCACATAATTTGCAATTCAAACATGGACTTGGAGCCTGGGGCGTTGACTCTTTTCCCAAAGATGTTGCCAATTGGTCCACTTTTGTCAACCAACCGATCCAAAAGACAAGTAGGCCACTTTTGGAGTGAAGACTCCACTTGCCTAACATGGCTTGATCAACAACCGATCTGCTCGGTCATCTATGTGGCATTTGGGAGCTTCACAATATTTGACCAGACTCAGTTTGAAGAGCTCGCACTAGGGCTCGAGTCAACCAAAAAGCCATTCTTATGGGTTGTTCGACCTAGTACAAGTGGAAGCATTGATTATGCATTCCCAAGTGGTTACATGGACCGAATAGCCAATTATGGGAAAATTGTGAGTTGGGCACCTCAACAGGAGGTGCTAAAGCATCCGTCGGTTGCTTGCTTTATGAGCCATTGTGGTTGGAACTCTACAGCAGAAGGTGTGAGCAATGGTCTTCCATTCTTGTGTTGGCCATACTTTGCTGATCAGTTTTTTAATGAAACTTACATATGTGATATTTGGAAAACCGGTGTCGGTTTAAACAAAGATGATACAGGCATTGTTCCGCGAGGAGAAATCAAGAGCAAAGTAGAAGAGTTGCTTAGCAACAAGATACTAAAAGAAAATGCCTTGAATTTGCATAAAAAACTGAGAGACTGCTTAAGAGCAGACAATTCCTCACAAAAAAATTTGAAAAAATTCGTTGAATGGATAAAAGAAGAAAATGATTATGCTTGTGCAAATGAAACCAACAAATGA

MKTIHVLAIPYPAQGHVIPLMEVARCLTNYCLKVTFVNSEFTNKQIMSSGSSIDVPSDLLQMVSIPDGIETFEDRNNLGKLSETMFQFMPSKLEELINDINKNEDERIVCIIADYCMGWVKSVAQKMGIRLAVFCPTSAAVLAVTMSIRKLMDDEILNCKGEATSKNQMVQLSASMPLMDPAHFVWVCMGDPIVNQIFFDYLFYKGMEAAEAADHIICNSNMDLEPGALTLFPKMLPIGPLLSTNRSKRQVGHFWSEDSTCLTWLDQQPICSVIYVAFGSFTIFDQTQFEELALGLESTKKPFLWVVRPSTSGSIDYAFPSGYMDRIANYGKIVSWAPQQEVLKHPSVACFMSHCGWNSTAEGVSNGLPFLCWPYFADQFFNETYICDIWKTGVGLNKDDTGIVPRGEIKSKVEELLSNKILKENALNLHKKLRDCLRADNSSQKNLKKFVEWIKEENDYACANETNK

SrUGT75E1-2

ATGGCTCAATCTCAACCTCAACCACACTTCCTCGTTGTAACCTTTCCAGCCCAAGGTCACATTAACCCGGCTCTACAGTTCGCTAAACAACTCGCCCGGTTCGATGTTAAACTCACCTTCACCACCACCGTCTCCGCCTACCGTCGCATGACCAAATTTGACCAGATTTACAATAATTTCAACTTTGTTGTTTATTCAGACGGCTTCGATGACGGTTTCACTTCTAAATCCGGTGACCCTGATCTCTTCATGACTCAGTTTAGGACCCGGGGAATCCAATCCTTGAAAGAAACCATAACTTCTAGTGCTGAAAATGGCACACCGGTCACATGTTTGGTGTACACCTTGCTCCTACCTTGGGCTGCAGAACTGGCGCGTGAACTTAACGTGCAACCAGCCCTTCTTTGGATCCAACCAGCATCGGTGTTTCGTGTGTACTATTATTATTTCAATGGGTATGATAAACTCATTGGTGAAGATTGTACCGACTCTTCATGGTCCATCGAGTTACCGGGGATACCATCGCTCAAAAGTTGTGATTTACCCTCGTTTTGTCTCCCTTCGAGCCCTTATGATATGTTGTTATCTTTGTTTAAGGAACTGCTTCATACGTTGAGTTCGTTTGAAAAGCCGAAGATACTTGCGAATACGTTTGATGCGTTGGAAGAAGAGGCCTTGAAAGAGATTGATGGCAAGATAAACATGATTGGTGTTGGACCGTTGATTCCATCGGCTTTCTTGGATGGAAACGAGCAATCGGATAGATCTTTTGGAGGAGATATGTTTGACAAATCACATGATTGTTTGGAATGGATGAACACAAATCATGAAGGATCCATTGTTTACGTATCTTTTGGTAGTCTTATAGAATTGTCAAAGAAAGAAAAGGAGTCAATAGCTTGTGGTTTGTTGGAGAGCAAAAGGCCTTTTTTGTGGGTGATGAGAGATAAAGATGGAGAAGCAAAAGAAAAGGAAGATGAAATAAGTTGCATAGAGGAATTGAAACAATTAGGTTTGATAGTTCCTTGGTGCAGCCAACTAGAGGTGTTGTCACACCCGTCTTTAGGTTGTTTTGTGACACATTGTGGTTGGAATTCGACACTCGAGAGTATCGCGTGTGGGGTTCCGGTGGTTGCGTTTCCTAGATGGTCCGATCAAACAACAAATGCAAAGCTTATTGAAGATGTGTGGGGAATTGGGACGAGGGTGACCGCGAATGATCAAGACGGAGTTGTTGAAGCGGAGGAGATAAGAAGGTGTATAGAAATGGTGATGGGAGGTCATGAAAGAGGAGAAACAATGAGAATGAATGCTAAGAAGTGGAAGGATTTTGCTAGAGAGGCTATGAAAGAAAGTGGATCTTCGTATATCAATCTCAAGGATTTTGTTACACAAATTGGAAGTTCTACTTAA

MAQSQPQPHFLVVTFPAQGHINPALQFAKQLARFDVKLTFTTTVSAYRRMTKFDQIYNNFNFVVYSDGFDDGFTSKSGDPDLFMTQFRTRGIQSLKETITSSAENGTPVTCLVYTLLLPWAAELARELNVQPALLWIQPASVFRVYYYYFNGYDKLIGEDCTDSSWSIELPGIPSLKSCDLPSFCLPSSPYDMLLSLFKELLHTLSSFEKPKILANTFDALEEEALKEIDGKINMIGVGPLIPSAFLDGNEQSDRSFGGDMFDKSHDCLEWMNTNHEGSIVYVSFGSLIELSKKEKESIACGLLESKRPFLWVMRDKDGEAKEKEDEISCIEELKQLGLIVPWCSQLEVLSHPSLGCFVTHCGWNSTLESIACGVPVVAFPRWSDQTTNAKLIEDVWGIGTRVTANDQDGVVEAEEIRRCIEMVMGGHERGETMRMNAKKWKDFAREAMKESGSSYINLKDFVTQIGSST

SrUGT88B1-1

ATGGAGTCCTCTAAGGTGATCCTGTATCCTTCTCCCGGAATCGGCCATCTTGTTTCCATGGTGGAGCTTGGAAAACTCATCCACACCCACCACCCTTCACTCTCCGTTATCATCCTCGTACTTCCGGCTACATATGAAACCGGGTCCACCACTACATACATCAACACCGTCTCCACCACCACCCCCTTCATCACCTTCCACCACCTCCCCGTTATCCCTCTTCCACCAGACTCATCTTCTGAATTCATAGACCTTGCCTTCGATATCCCTCAACTTTACAACCCGGTCGTCTACAACACCCTCGTAGCCATCTCCGAAACCTCAACCATCAAAGCTGTCATCCTTGATTTCTTTGTAAACGCAGCTTTTCAGATCTCTAAAAGTCTCGATCTTCCCACTTACTACTTCTTTACCAGTGGCGCCTCTGGTCTCTGTGCGTTCTTACATCTTCCAACCATCTACAAAACATATTCCGGAAACTTTAAAGATCTAGATACTTTCATTAATATTCCTGGGGTACCTCCCATTCATTCTTCCGATATGCCCACAGTTATGTTTGATAAGGAAAGTAATTCCTACAAAAACTTCGTAAAAACCTCAAATAACATGGCAAAATCTTCCGGAGTCATTGCAAACAGCTTCTTGCAGTTGGAGGAAAGAGCTGCTCAAACTCTCCGAGATGGTAAATCCATCACGGACGGTCCCTCTCCACCTATTTATCTAATCGGGCCTTTAATCGCTAGCGGCAATCAAGTTGATCATAACGAAAACGAGTGTTTAAAATGGCTGAACACACAACCTAGTAAAAGTGTAGTGTTTTTGTGCTTTGGGAGCCAGGGTGTGTTTAAGAAAGAACAATTGAAGGAAATAGCGGTTGGGTTAGAGAGAAGTGGGCAAAGATTTTTATGGGTGGTGCGAAAGCCGCCATCAGATGGTGGTAAAGAGTTCGGTCTTGATGATGTTCTTCCTGAAGGGTTTGTAGGCAGGACTAAAGAAAAGGGTCTGGTGGTGAAGAACTGGGCGCCTCAACCAGCGATTCTTGGTCATGAATCGGTGGGAGGATTTGTGAGTCATTGCGGGTGGAACTCGTCGCTTGAAGCGGTTGTTTTTGGTGTGCCGATGGTGGCATGGCCGTTGTACGCAGAGCAGAAGATGAACAGAGTGTATTTGGTTGAGGAAATAAAGGTGGCACTTTGGTTGAGAATGTCGGTAGATGGGTTTGTGAGTGCAGAGGCGGTAGAGGAGACGGTGAGACAGTTAATGGATGGGAGAAGAGTGAGAGAACGGATTTTGGAGATGAGTACAAAAGCGAAGGCTGCGGTGGAGGACGGCGGTTCCTCTCGAGTTGATTTCTTCAAATTAACTGAGTCATGGACCCACAAGTGA

MESSKVILYPSPGIGHLVSMVELGKLIHTHHPSLSVIILVLPATYETGSTTTYINTVSTTTPFITFHHLPVIPLPPDSSSEFIDLAFDIPQLYNPVVYNTLVAISETSTIKAVILDFFVNAAFQISKSLDLPTYYFFTSGASGLCAFLHLPTIYKTYSGNFKDLDTFINIPGVPPIHSSDMPTVMFDKESNSYKNFVKTSNNMAKSSGVIANSFLQLEERAAQTLRDGKSITDGPSPPIYLIGPLIASGNQVDHNENECLKWLNTQPSKSVVFLCFGSQGVFKKEQLKEIAVGLERSGQRFLWVVRKPPSDGGKEFGLDDVLPEGFVGRTKEKGLVVKNWAPQPAILGHESVGGFVSHCGWNSSLEAVVFGVPMVAWPLYAEQKMNRVYLVEEIKVALWLRMSVDGFVSAEAVEETVRQLMDGRRVRERILEMSTKAKAAVEDGGSSRVDFFKLTESWTHK

SrUGT73E1-2

ATGGTGGCACCACCAACCAACCTTCATTTTGTTTTGTTTCCTCTTATGGCTCAAGGCCATCTGGTACCCATGGTCGACATCGCTCGAATCTTAGCCCAACGTGGTGCAACGGTCACCATAATCACCACACCCTACGATGCCAACCGGGTCAGACCGGTTATCTCCCGAGCCATCGCGACCAATCTCAAGATCCAGCTACTCGAACTCCAACTGCGGTCAACCGAAGCCGGTTTACCCGAAGGGTGCGAAAGCTTCGACCAACTTCCGTCATTCGAGTACTGGAAAAATATTTCAACCGCTATCGATTTGTTACAACAACCCGCTGAAGATTTGCTCCGAGAACTTTCACCACCACCCGATTGCATCATATCGGACTTTTGGTTCCCGTGGACCACCGATGTGGCTCGACGGTTAAACATCCCCCGGCTCGTGTTCAACGGAAAGGGCTGCTTTTATCTCTTGTGCATGCATGTTGCGATCACTTCCAACATTTTGGGAGAGAATGAACCGGTCAGTAGTAATACCGAGCGCGTTGTGCTGCCCGGTTTACCTGACCGGATCGAAGTCACTAAACTTCAGATCCTCGGTTCGTCGAGACCAGCCAACGTAGACGAAATGGGCTCGTGGCTTCGAGCCGTAGAAGCCGAGAAAGCTTCATTCGGGATAGTGGTTAATACTTTCGAAGAGCTTGAACCGGAGTACGTTGAAGAATACAAAACGGTTAAAGATAAGAAGATGTGGTGTATCGGCCCGGTTTCGTTATGCAACAAAACCGGGCCGGATTTAGCCGAGCGAGGAAACAAGGCTGCAATAACCGAACACAACTGCTTAAAATGGCTCGATGAGAGAAAACTGGGGTCCGTGTTATACGTTTGTTTAGGTAGCCTTGCACGCATTTCTACCGCACAAGCAATCGAGCTCGGGTTAGGACTCGAGTCCATAAACCGACCCTTTATATGGTGCGTAAGAAACGAAACCGATGAGCTCAAAACATGGTTTTTGGATGGGTTTGAAGAAAGGGTTAGAGATCGCGGGTTGATCGTTCATGGTTGGGCGCCACAGGTTTTGATACTGTCGCACCCAACCATTGGCGGTTTCTTAACCCATTGCGGTTGGAACTCGACTATTGAATCGATTACCGCGGGTGTTCCAATGATCACGTGGCCGTTTTTTGCGGACCAGTTTTTGAATGAAGCTTTTATAGTTGAAGTTTTGAAGATTGGAGTTAGGATTGGTGTTGAGAGAGCTTGTTCGTTTGGGGAAGAAGATAAGGTTGGAGTGTTGGTGAAGAAGGAGGATGTGAAAAAGGCTGTTGAATGCTTGATGGATGAAGATGAAGATGGTGATCAGAGAAGAAAGAGGGTGATTGAGCTTGCAAAAATGGCGAAGATTGCAATGGCGGAAGGTGGATCTTCTTATGAAAATGTATCGTCGTTGATTCGAGATGTGACTGAAACAGTTAGAACACCACATTAG

MVAPPTNLHFVLFPLMAQGHLVPMVDIARILAQRGATVTIITTPYDANRVRPVISRAIATNLKIQLLELQLRSTEAGLPEGCESFDQLPSFEYWKNISTAIDLLQQPAEDLLRELSPPPDCIISDFWFPWTTDVARRLNIPRLVFNGKGCFYLLCMHVAITSNILGENEPVSSNTERVVLPGLPDRIEVTKLQILGSSRPANVDEMGSWLRAVEAEKASFGIVVNTFEELEPEYVEEYKTVKDKKMWCIGPVSLCNKTGPDLAERGNKAAITEHNCLKWLDERKLGSVLYVCLGSLARISTAQAIELGLGLESINRPFIWCVRNETDELKTWFLDGFEERVRDRGLIVHGWAPQVLILSHPTIGGFLTHCGWNSTIESITAGVPMITWPFFADQFLNEAFIVEVLKIGVRIGVERACSFGEEDKVGVLVKKEDVKKAVECLMDEDEDGDQRRKRVIELAKMAKIAMAEGGSSYENVSSLIRDVTETVRTPH

SrUGT72F1

ATGGAAAAACCACCACATATCGCCATTGTACCCAGCCCAGGTATGGGTCACCTAATCCCAATGGTTGAATTTGCAAAAACACTTGCAACCAAACACAATCTCTCTGCAACTTTCATCATCACAAACGATGGCCCTTTATCAAATTTACAACATGAATTTCTCGACTCACTACAAAATCCCATGAATTACATCCTCCTCCC

ACCTGTTAATTTTGATGATTTGCCTCCAGATACCAAAATCGAGACCCGAATAAGCCTCATGGTGACCCGATCCATTACTCCTCTTCGTGAAACCATGAACTCCATGATTGCAGACAAAAAGATTGTTGCTTTGTTTGCTGATCTGTTTACAACTGATGCATTCGATGTCGCTATTGAATTCAGTGTCCTGCATTATCTGTTCTTTCCGGCATCCGCCATGACTTTGTCTCTGTTTCTTCATTTGCCAAAACTTGATCAAATGATTTCTGGACCATATAAGGACATACCCGACCCGATTCAGTTCCCAGGTTGCATAACGGTTCATGGTAAGGACCTGCTTGACCCGGTTCAAGATAGGGATAATGATGCATACAAATGGTTACTACACAATGTAAAGAGG

TATACTATGGCTAAGGGTATAGTGGTAAATAGCTTCAAGGAGTTGGAGAGTGGAGCTATTGAAGCTTTACAACATGAGGCACCGGGTAACCCACCGGTTTACCCGGTTGGACCATTAATACGGCTAGGATCAGTTGAGTCTACTAAGGATGTAAACGAGTTGAGCTGTTTAAGATGGCTCGACGATCAGCCACGTGGGTCTGTTTTGTATATTAGTTTTGGGAGTGGTGGGACCCTTTCTTCGGAACAAATCAACGAGTTAGCCATGGGTTTGGAGTTGAGCGAGCAAAGGTTCATATGGGTGGTTAGAACCCCGAATGATAAAATTGCCGATGCTACATACTTCAATACCAATTGTCAAAATGACACTTTTGACTTCTTACCAAAAACATTTTTGGAAAGAACAAAAGGTTATGGGCTGGTGGTGCACAATTGGGCACCACAAGCCCAAATCTTGAGTCATAGCTCAACAGGTGGGTTTATGACTCACTGTGGTTGGAACTCGATTCTTGAAACAATAGTTCACGGGGTACCAATGATCGGTTGGCCACTTTATGCAGAACAAAAGATGAACGCTATAATGTTGACCGAGGGTCTGAAAATTGCGTTGAGGGCTAATACGAATGAAAATGGCATGGTCGATCGTTTAGAGATTGTTCGGGTCGTCAAGGGTTTATTGGAAGGAGGGAAAGAGATTCGAATTCGAACTCAAGAGCTTAAAGAAGCGGCTGCGAGTGTTCTAAGCAAAGATGGATGTTCAACAAAAACACTAGATCAACTG

MEKPPHIAIVPSPGMGHLIPMVEFAKTLATKHNLSATFIITNDGPLSNLQHEFLDSLQNPMNYILLPPVNFDDLPPDTKIETRISLMVTRSITPLRETMNSMIADKKIVALFADLFTTDAFDVAIEFSVLHYLFFPASAMTLSLFLHLPKLDQMISGPYKDIPDPIQFPGCITVHGKDLLDPVQDRDNDAYKWLLHNVKRYTMAKGIVVNSFKELESGAIEALQHEAPGNPPVYPVGPLIRLGSVESTKDVNELSCLRWLDDQPRGSVLYISFGSGGTLSSEQINELAMGLELSEQRFIWVVRTPNDKIADATYFNTNCQNDTFDFLPKTFLERTKGYGLVVHNWAPQAQILSHSSTGGFMTHCGWNSILETIVHGVPMIGWPLYAEQKMNAIMLTEGLKIALRANTNENGMVDRLEIVRVVKGLLEGGKEIRIRTQELKEAAASVLSKDGCSTKTLDQL

SrUGT73C1

ATGGCTTTAGATGAGTCACATGAACCCAACCAACTTCACTTTCTTGTTATACCCTTAGGATCTCCAGGCCACTATATCCCAACCATTGATTTAGCCAAATTACTAGCTCAACATGGTGTTAGAGTCACAATAATCACCACACCGGTCAACGCAATCAGATTCGGGTCAATCCTTGATCAAGCAATCAAATCGGGTCTTCCCATTAATTTTCTTGAATTTCAGTTACCATATCTGAAGTTTGGTATTCCAGAAGGTTGTGAATGCATGGATGATCTTCCTAATATTGGATTAGTCAATGATTTTTTTCTTGCCCATAGTTCACTTCAACAAGAAGTTGAACAATATATTGAAAAGCTTGATTCTAAACCAAGTTGCATACTTTCAAGCACATATATTCTTTGGACAGATGAAACATCAAAAAAATTTAAGATTCCAAAGATTGTGTTTGATGGAATGAATTGCTTCACTCAAATGTGTAACCGCGTTTTATACCTCTCAAAGGTATATGAAAGTGTCAACGAGTCGGATTATTTTGTTTTGCCTGGGTTGCCTGATCATATTGAACTAACAAGATCGCAACTAGCCTTCATATTCAATTCAGGTTCCAAAGACGTAAAGGATTATAGGGAAAAGCTTCGGGTATCGGAGTCCGAAGCATTTGGGATAGTTATAAACAGTTTTCAGGAGTTGGAACAAGAATATGTTGATGCATATCAAAAAGTCAAAGAAGATAAAGCTTGGTGCATAGGGCCATTATCTCTATGCCACAAGGATGCATCCGAGAAGGTCCAAAGAGGTAACAAGTCCTCAATTGACAAAAATGAATGCATCAAGTGGCTAGATTCTCAAGAAAACGAATCGGTGATATACACATGTTTAGGTAGCATTAGTCGCCTTGAGCCTACACAACTCATAGAGCTCGCTTTAGGTCTAGAGTCATCAAAAAAACCATTCATTTGGGTGGTTCGAGCTGGTCATAAGACTGAGAAGATAGAAAAGTGGATAGATGAAGAGGGGTTTGAACAAAGAACCAAAGATAGAGGTCTACTGATCCGCGGGTGGGCTCCACAACTGCTAGTGTTGTCACACCCTGCAATTGGTGCGTTTTTGACTCATTGCGGTTGGAACTCGGCTCTAGAAGGGATATCTGCTGGTGTCCCTATGGTAACATGGCCTCAGTTTCAAGAACAATTTTACAATGAGAAGTTACTTGTACAAGTATTAAAAATTGGCGTTAGTGTTGGCGCGCAAAAAGTTGTGCATTGGGGTGAAGAAGAAAAGTCGGGAGTGGTAGTAAAGAGTGAGGAATTTATAAAGGCTATAGAGCTGGTGATGGAAGATGGGAAAGAAAGTGAAGATAGAAGAAAGAGAGCTAAAGAACTTGGTAAGATGGCTAATGAAGCAATAGAAGATGGAGGATCTTCTCACCGGAATATGACACGATTAATCCAAGATATTAGGAACCTATCATGTGCAAACAATTCAAGCTAA

MALDESHEPNQLHFLVIPLGSPGHYIPTIDLAKLLAQHGVRVTIITTPVNAIRFGSILDQAIKSGLPINFLEFQLPYLKFGIPEGCECMDDLPNIGLVNDFFLAHSSLQQEVEQYIEKLDSKPSCILSSTYILWTDETSKKFKIPKIVFDGMNCFTQMCNRVLYLSKVYESVNESDYFVLPGLPDHIELTRSQLAFIFNSGSKDVKDYREKLRVSESEAFGIVINSFQELEQEYVDAYQKVKEDKAWCIGPLSLCHKDASEKVQRGNKSSIDKNECIKWLDSQENESVIYTCLGSISRLEPTQLIELALGLESSKKPFIWVVRAGHKTEKIEKWIDEEGFEQRTKDRGLLIRGWAPQLLVLSHPAIGAFLTHCGWNSALEGISAGVPMVTWPQFQEQFYNEKLLVQVLKIGVSVGAQKVVHWGEEEKSGVVVKSEEFIKAIELVMEDGKESEDRRKRAKELGKMANEAIEDGGSSHRNMTRLIQDIRNLSCANNSS

SrUGT73C2

ATGGCTTTAGAAGAATCACAAGAACCCAACCAACTTCACTTTCTTGTAATACCCTTAGGATCTCCAGGTCACTATATCCCAACCATTGATTTATCCAAATTACTAGCTCAACATGGAGTTAGAGTCACCATAGTCACCACCCCGGTTAACGCCGTCAGATTCGGGTCAATCCTTGATCAAGCAATCAGATCGGGTCTTCCCATTAGTTTTCTTGAATTTCGGTTACCATATATGAAGTTTAACATACCAGAAGGTTGTGAATGCTTAGATGATGTTCCTAATATTGGGTCAGCCCATGATCTTTTTCTTGCACATAGTTCACTGCAACAAGAAGTTGAAGAATATATTGAAAAGCTTGATCATAAACCAAGTTGCATACTTTCAGGCACATATCTTTTATGGACAAGTGAAACTGCAAAAAAGTTTCAGATTCCAAGGATTGTGTTTGATGGAATGAATTGCTTCACTCAGATGTGTAACCATGTTTTATACATCTCAAAGGTGTATGAAAGTGTTAGTGAGTCAGAATCTTTTGTGTTACCCTGTTTGCCTGATCGTATTGAACTAACAAGATCCCAACTATCTTTTGTATTCAATTCGGGCTCCAAAGACGTGAAGGATTTAAGTGAGAAGCTTCGGGTATCGGAGTCCGAAGCGTTTGGTATAGTTATAAATAGTTTTCATGAGTTGGAGCAAGAATATGTTGAGGCATATCAAAAAGTCAAAGAAGATAAAGCTTGGTGCATAGGGCCATTATCTCTATGCCACAAGGATGTATCCGAGAAAGTCCAAAGAGGTAACAAGTCCTCAATTGACAAAGATGAATGCATCAAGTGGCTCGATTCTCAAGAAAATGAATCGGTAATCTATGTGTGTTTAGGTAGCATCAGTCGCCTTGAGCCTTCACAGCTCATAGAGCTTGCTTTAGGTCTTGAGTCATCAAAAAGACCGTTCATTTGGGTGGTTCGAGCCGGCCACAAGACTGAGAAGATAGAAAAGTGGATAGAAGAAGAGGGGTTTGAAGAGAGAACCAAAGATAGAGGTCTATTGATCCGCGGGTGGGCTCCACAAGTGCTAATACTGTCACACCCTGCAGTTGGTGCGTTTTTGACTCACTGTGGTTGGAACTCAGCTCTCGAAGGGATATCTGCGGGTGTCCCTATGGTGACGTGGCCTCAGTTTCAAGAACAATTTTACAATGAGAAGTTGCTCGTACAAGTGTTGAGAATTGGCGTTAGTGTTGGCGCGCAAAAAGTTGTGCATTGGGGTGAAGAAGAAAAGTCAGGAGTGGTAGTAAAGAGTGAGGAATTTAGTAAGGCTATAGAGATGATGATGGAAGATGGGAAAGAAAGTGAAGATAGAAGAAAGAGAGCCAAAGATCTTGGTAAGATGGCAAATGAAGCAGTTGAAGAAGGAGGATCTTCTCACCGGAATATGACACGATTAATCCAAGATATTAGGAACCTATCTAGTACAAGGAATTCAAGCTAA

MALEESQEPNQLHFLVIPLGSPGHYIPTIDLSKLLAQHGVRVTIVTTPVNAVRFGSILDQAIRSGLPISFLEFRLPYMKFNIPEGCECLDDVPNIGSAHDLFLAHSSLQQEVEEYIEKLDHKPSCILSGTYLLWTSETAKKFQIPRIVFDGMNCFTQMCNHVLYISKVYESVSESESFVLPCLPDRIELTRSQLSFVFNSGSKDVKDLSEKLRVSESEAFGIVINSFHELEQEYVEAYQKVKEDKAWCIGPLSLCHKDVSEKVQRGNKSSIDKDECIKWLDSQENESVIYVCLGSISRLEPSQLIELALGLESSKRPFIWVVRAGHKTEKIEKWIEEEGFEERTKDRGLLIRGWAPQVLILSHPAVGAFLTHCGWNSALEGISAGVPMVTWPQFQEQFYNEKLLVQVLRIGVSVGAQKVVHWGEEEKSGVVVKSEEFSKAIEMMMEDGKESEDRRKRAKDLGKMANEAVEEGGSSHRNMTRLIQDIRNLSSTRNSS

SrUGT73C3

ATGGCTTTAGAAGAATCACAAGAACTAAACCACCTTCACTTTCTTGTTATACCCATTGGATCTCCAGGGCACTACATCCCAACAATTGATATGTCCAAGTTACTAGCTCAACACGGCATTAGAGTCACCATAGTCACCACCCCGGTCAATACCCTAAGATTCGGGTCAATCCTTGATCAAGCAATCCAATCAGGTCTTTCGATCCATTTTCTTGAATTCCCGTTTCCATATAAGGAGTTTGGTTTACCCGAAGGATGTGAAAGCATAGATGATCTTCCTTATATGGGGCTCACAAGTGCACTTATGCAAGCCTATGCTTCATTACAAAAAGAAGTTGAACAATATCTTGAAAATCTTAATCCTAAGCCTAACTGCATACTTTCAGACACATTTCTCCTATGGCCAGGTGAAATAGCAAAAAGGTTTGAGATCCCAAGAATTCTATTTGATGGGATGAATTGCTTTACTCAGATGTGTAACCATGTATTATACCTCTCAAAGGTGTATGAAACAGTGGGTGAGTCGGATTCATTCGTTTTGCCTGATTTGCCTGATTTGCCTGACCGTATTGAGCTAACAAGATCTCAACTTCCTTTTGCATTCAACCCGACACTTAAAGGTATGAGTGATTTCAATGAAAAGCTTCGGGTATCTGAATCCGAAGCGTATGGGGTAGTTGTAAATAGTTTTGAGGAGTTGGAACAAAGATATGTTGATGAATATCAGAAAGTTAAAGAAGGTAAAGTTTGGTGCATAGGGCCATTATCACTATGCCACAATGATGAATCAGATAAGGTCCA

AAGAGGTAACAAGTCATCAATCAACAAACACGAGTGTCTCAATTGGCTCGATTCTCAAGAAAACGGTTCAGTGATCTATGCTTGTTTAGGAAGCATTAGTCGGGTTGAGCCTGCACAGCTCACAGAGCTTGCTTTAGCTCTAGAAGCATCGAAAAGACCGTTCATATGGGTGGTTCGAGCAGGTCATAAGACTGAGATGATAGAAAAATGGATAGATGAAGAGGGGTTTGAAGAAAGGACCAAAGATAAAGGTCTATTGATCCGTGGGTGGGCACCACAACTGCTAGTGTTGTCACACCCTGCGATTGGAGGGTTTTTAACACATTGTGGATGGAACTCGACTCTAGAAGGGATTTGTGCTGGTGTCCCTTTGGTAACATGGCCTCAGTTTCAAGAACAATTTTACAATGAGAAGTTAGTTGTGCAAGTGTTGAGAATTGGAGTTAGTGTTGGTGCTCAAAAAAGCGTGTTTTGGGGTGATGGAGATAAGTCTGGAGTGGTAAAGAGTGAGGCTTTTGTAAAGGCTATAGAGATTGTAATGGAAGACGGGGAAGAAGGTGATGAGAGAAGAAAGAGAGCGAAAGAACTTGGTAAGATGGCGAATAAAGCAGTAGAAGAGGGAGGATCTTCGGACCGAAATATTACTCGACTAATTGAAGATTTCAAGAACCAAGCAAGATCTAGGAATTAA

MALEESQELNHLHFLVIPIGSPGHYIPTIDMSKLLAQHGIRVTIVTTPVNTLRFGSILDQAIQSGLSIHFLEFPFPYKEFGLPEGCESIDDLPYMGLTSALMQAYASLQKEVEQYLENLNPKPNCILSDTFLLWPGEIAKRFEIPRILFDGMNCFTQMCNHVLYLSKVYETVGESDSFVLPDLPDLPDRIELTRSQLPFAFNPTLKGMSDFNEKLRVSESEAYGVVVNSFEELEQRYVDEYQKVKEGKVWCIGPLSLCHNDESDKVQRGNKSSINKHECLNWLDSQENGSVIYACLGSISRVEPAQLTELALALEASKRPFIWVVRAGHKTEMIEKWIDEEGFEERTKDKGLLIRGWAPQLLVLSHPAIGGFLTHCGWNSTLEGICAGVPLVTWPQFQEQFYNEKLVVQVLRIGVSVGAQKSVFWGDGDKSGVVKSEAFVKAIEIVMEDGEEGDERRKRAKELGKMANKAVEEGGSSDRNITRLIEDFKNQARSRN

SrUGT73C4

ATGGCTTTAGAAGAATCACAAGAACCCAACCAACTTCACTTTCTTGTAATACCCTTAGGATCTCCAGGTCACTATATCCCAACCATTGATTTATCTAAATTACTAGCTCAACATGGAGTTAGAGTCACCATAGTCACCACCCCGGTTAACGCCGTCAGATTCGGGTCAATCCTTGATCAAGCAATTCAATCGGGTCTTCCCATTAGTTTTCTTGAATTTCGGTTACCATATATGAAGTTTAACATACCAGAAGGTTGTGAATGCTTGGATGATGTTCCTAATATTGGGTCAGCCCATGATCTTTTTCTTGCACATAGTTCACTGCAACAAGAAGTTGAAGAATATATTGAAAAGCTTGATCATAAACCAAGTTGCATACTTTCAGGCACATATCTTTTATGGACAAGTGAAACTGCAAAAAAGTTTCAGATTCCAAGGATTGTGTTTGATGGAATGAATTGCTTCACTCAGATGTGTAACCATGTTTTATACATCTCAAAGGTGTATGAAAGTGTTAGTGAGTCAGAATCTTTTGTGTTACCCGGTTTGCCTGATCGTATTGAACTAACAAGACCCCAACTATCTTTTGTATTCAATTCGGGCTCCAAAGACGTGAAGGATTTCAGTGAGAAGCTTCGGGTATCGGAGTCCGAAGCGTTTGGTATAGTTATAAATAGTTTTCATGAGTTGGAGCAAGAATATGTTGAGGCATATCAAAAAGTCAAAGAAGATAAAGCTTGGTGCATAGGGCCATTATCTCTATGCCACAAGGATGTATCCGAGAAAGTCCAAAGAGGTAACAAGTCCTCAATTGACAAAGATGAATGCATCAAGTGGCTCGATTCTCAAGAAAATCAATCGGTAATCTATGTGTGTTTAGGTAGCATCAGTCGCCTTGAGCCTTCACAGCTCATAGAGCTTGCTTTAGGTCTTGAGTCATCAAAAAGACCGTTCATTTGGGTGGTTCGAGCCGGCCACAAGACTGAGAAGATAGAAAAGTGGATAGATGAAGAGGGGTTTGAAGAGAGAACCAAAGATAGAGGTCTATTGATCCGCGGGTGGGCTCCACAAGTGCTAATACTGTCACACCCTGCAGTTGGTGCGTTTTTGACTCACTGTGGTTGGAACTCAGCTCTCGAAGGGATATCTGCAGGTGTCCCTATGGTGACGTGGCCTCAGTTTCAAGAACAATTTTACAATGAGAAGTTGCTCGTACAAGTGTTGAGAATTGGCGTTAGTGTTGGCGCGCAAAAAGTTGTGCATTGGGGTGAAGAAGAAAAGTCAGGAGTGGTAGTAAAGAGTGAGGAGTTTACTAAGGCTATAGAGATGATGATGGAAGATGGGAAAGTGTTAAAATATAAGTGGTTCTTTGTCATATTAATGGGCTATTTATTTCTTCTCCATGCAGGCTTGGGCTCTCTACTAAGCCCAGCTTTCTCATCCAAAATAAGGAGCTGA

MALEESQEPNQLHFLVIPLGSPGHYIPTIDLSKLLAQHGVRVTIVTTPVNAVRFGSILDQAIQSGLPISFLEFRLPYMKFNIPEGCECLDDVPNIGSAHDLFLAHSSLQQEVEEYIEKLDHKPSCILSGTYLLWTSETAKKFQIPRIVFDGMNCFTQMCNHVLYISKVYESVSESESFVLPGLPDRIELTRPQLSFVFNSGSKDVKDFSEKLRVSESEAFGIVINSFHELEQEYVEAYQKVKEDKAWCIGPLSLCHKDVSEKVQRGNKSSIDKDECIKWLDSQENQSVIYVCLGSISRLEPSQLIELALGLESSKRPFIWVVRAGHKTEKIEKWIDEEGFEERTKDRGLLIRGWAPQVLILSHPAVGAFLTHCGWNSALEGISAGVPMVTWPQFQEQFYNEKLLVQVLRIGVSVGAQKVVHWGEEEKSGVVVKSEEFTKAIEMMMEDGKVLKYKWFFVILMGYLFLLHAGLGSLLSPAFSSKIRS

SrUGT73E1-1

ATGGTGGCACCACCAACCAACCTTCATTTTGTTTTGTTTCCTCTTATGGCTCAAGGCCATCTGGTACCCATGGTCGACATCGCTCGAATCTTAGCCCAACGTGGTGCAACGGTCACCATAATCACCACACCCTACCATGCCAACCGGGTCAGACCGGTTATCTCCCGAGCCATCGCGACCAATCTCAAGATCCAGCTACTCGAACTCCAACTGCGGTCAACCGAAGCCGGTTTACCCGAAGGGTGCGAAAGCTTCGACCAACTTCCGTCATTCGAGTACTGGAAAAATATTTCAACCGCTATCGATTTGTTACAACAACCCGCTGAAGATTTGCTCCGAGAACTTTCACCACCACCCGATTGCATCATATCGGACTTTTTGTTCCCGTGGACCACCGATGTGGCTCGACGGTTAAACATCCCCCGGCTCGTGTTCAATGGACCGGGCTGCTTTTATCTCTTGTGCATCCATGTTGCGATCACTTCCAACATTTTGGGAGAGAATGAACCGGTCAGTAGTAATACCGAGCGCGTTGTGCTGCCCGGTTTACCTGACCGGATCGAAGTCACTAAACTTCAGATCGTCGGTTCGTCGAGACCAGCCAACGTAGACGAAATGGGCTCGTGGCTTCGAGCCGTAGAAGCTGAGAAAGCTTCATTCGGGATAGTGGTTAATACTTTCGAAGAGCTTGAACCGGAGTACGTTGAAGAATACAAAACGGTTAAAGATAAGAAGATGTGGTGTATCGGCCCGGTTTCGTTATGCAACAAAACCGGGCCGGATTTAGCCGAGCGAGGAAACAAAGCTGCAATAACCGAACACAACTGCTTAAAATGGCTCGATGAGAGAAAACTGGGGTCCGTGTTATACGTTTGTTTAGGTAGCCTTGCACGCATTTCTGCCGCACAAGCAATCGAGCTCGGGTTAGGACTCGAGTCCATAAACCGTCCCTTTATATGGTGCGTAAGAAACGAAACCGATGAGCTCAAAACATGGTTTTTGGATGGGTTTGAAGAAAGGGTTAGAGATCGCGGGTTGATCGTTCATGGTTGGGCGCCACAGGTTTTGATACTGTCGCACCCAACCATTGGCGGTTTCTTAACCCATTGCGGTTGGAACTCGACTATTGAATCGATTACCGCGGGTGTTCCAATGATCACGTGGCCATTTTTTGCGGACCAGTTTTTGAATGAAGCTTTTATAGTTGAAGTTTTGAAGATTGGAGTTAGGATTGGTGTTGAGAGGGCTTGTTTGTTTGGGGAAGAAGATAAGGTTGGAGTGTTGGTGAAGAAGGAGGATGTGAAGAAGGCTGTTGAATGCTTGATGGATGAAGATGAAGATGGTGATCAGAGAAGAAAGAGGGTGATTGAGCTTGCAAAAATGGCGAAGATTGCAATGGCGGAAGGTGGATCTTCTTATGAAAATGTATCGTCGTTGATTCGAGATGTGACTGAAACAGTTAGAGCACCACATTAG

MVAPPTNLHFVLFPLMAQGHLVPMVDIARILAQRGATVTIITTPYHANRVRPVISRAIATNLKIQLLELQLRSTEAGLPEGCESFDQLPSFEYWKNISTAIDLLQQPAEDLLRELSPPPDCIISDFLFPWTTDVARRLNIPRLVFNGPGCFYLLCIHVAITSNILGENEPVSSNTERVVLPGLPDRIEVTKLQIVGSSRPANVDEMGSWLRAVEAEKASFGIVVNTFEELEPEYVEEYKTVKDKKMWCIGPVSLCNKTGPDLAERGNKAAITEHNCLKWLDERKLGSVLYVCLGSLARISAAQAIELGLGLESINRPFIWCVRNETDELKTWFLDGFEERVRDRGLIVHGWAPQVLILSHPTIGGFLTHCGWNSTIESITAGVPMITWPFFADQFLNEAFIVEVLKIGVRIGVERACLFGEEDKVGVLVKKEDVKKAVECLMDEDEDGDQRRKRVIELAKMAKIAMAEGGSSYENVSSLIRDVTETVRAPH

SrUGT73F1

ATGGCTTCTCATCCCACAAATCTTCACTTTGTCTTGTTTCCTTTGATGGCTCAAGGTCATATGATTCCTATGGTAGACATGGCCCGTATACTAGCGGAACATGGTTCTATGGTTACTATAATCTCAAGCCCAGTTAACGCTAACCGCTTTAAGTCGATTATTGATCGTGCAGTTGAAGCTAAGCTCAAGATCCAGATTCTTGAACTCGGACTCCCACTAGCTGAGGTTGGTTTGCCGGAAGGAAGTGAGAATTTTGACTTGCTGCCATCAGTTGCCCACGTAGATAAATTGTACATAGCAATGCGTATGATAGAAGAACCAGCAGAAAACTTGCTCCGGGGTCTATGTCCGTCTCCAAGTTGCATCATCTCAGATGGTTGCTTTCCCTGGACTAATGACATCGCAAAGAGGTTGAATATTCCACGAGTTGTTTTCTATGGACCTGGATGTTTCTCATTCTTATGCATACACATAGCGACAAATACAAATATACTAGAAGAAATTGCATCTGACTCTGAATACTTTGTGATGCCTGGCTTGCCTGACCAGATTAAAGTCACCAAACCGCAGGTTGCAACTTGGGGGAGAGGAGGCTCAAAAGAATCCAACGCCGTTTTTGAAAAAATGCAAGAAGCCGAGAAAGATACAATAGGGATTGTGGTGAATAGCTTTGAGGAGTTGGAGCCCAAGTATGTTGAGGAATTTACAAAAGCAAAGGATAAAAAGGTGGGGTGCATTGGCCCGGTTTCACTGTGCAACAAAAGTTTCAAAGATAGAGTTGAGAGAGGAAACAAGACCGCAGTAAACGAGCATGATTGTTTGAAATGGCTCGACTCAAGAGAGTCAGGGTCAGTGGTTTATGTTTGCTTGGGTAGCCTATCATATGATTCCACTGAACAAGCCATTGAGCTTGGGTTGGGACTCGAGTCATCAAACATACCTTTTCTTTGGTTCGTAAGGAAAACAAGAGACGATTTTGAGACATGGCTCTCCAAAGAAGGATATGAAGAAAGGATAAAAGGTAGAGGCCTACTGATCCGTGGTTGGGCTCCACAAATCTTGATTTTGTCACATAAAGCAATCGGCGGTTTCGTAACACACTGTGGATGGAACTCGACTCTCGAAGGGATTTCGGCTGGGATGCCGATGGTTACATGGCCACATTTCGCAGAGCAATTTCTTAACGAGCGATTTATCATAGATGTGTTGAAGATTGGTGTGAGAATTGGGATGGAGGTTCCTATTATGTTCAAAAACCTAGACGAGTCAAAAGAGACACTGAAGAGGGATAATGTTAGGGAAGCTGTAGAACGCCTGATGAAAAACGATGAGGAAGGAGAAGCAAGAAGAAAGAGAGCTAGGGAGTTAAGGGAAATGGCGGAGAAAGCAATGGAAGAAGGGGGCTCATCTCACCATAATATGACATTGATGATTCAAGCAATTACCAAAGAGTTAGCCAAGAACACTAAACCGATTCAAGATATTGTGTAG

MASHPTNLHFVLFPLMAQGHMIPMVDMARILAEHGSMVTIISSPVNANRFKSIIDRAVEAKLKIQILELGLPLAEVGLPEGSENFDLLPSVAHVDKLYIAMRMIEEPAENLLRGLCPSPSCIISDGCFPWTNDIAKRLNIPRVVFYGPGCFSFLCIHIATNTNILEEIASDSEYFVMPGLPDQIKVTKPQVATWGRGGSKESNAVFEKMQEAEKDTIGIVVNSFEELEPKYVEEFTKAKDKKVGCIGPVSLCNKSFKDRVERGNKTAVNEHDCLKWLDSRESGSVVYVCLGSLSYDSTEQAIELGLGLESSNIPFLWFVRKTRDDFETWLSKEGYEERIKGRGLLIRGWAPQILILSHKAIGGFVTHCGWNSTLEGISAGMPMVTWPHFAEQFLNERFIIDVLKIGVRIGMEVPIMFKNLDESKETLKRDNVREAVERLMKNDEEGEARRKRARELREMAEKAMEEGGSSHHNMTLMIQAITKELAKNTKPIQDIV

SrUGT73G1

ATGGCTTTGGAATCAGTCAACCAGCTTCACTTCCTCCTGATCCCCTTTCTAGCTCCTGGTCACACCATCCCCATGATCCACATGGCCAAATTGCTCGCACAACGACCAAATGTGATGGTCACCATCGTCACCACACCCGTAAACGCGATTCGATATGGTTCTACGCTTCAAGAACACATCGAATCCGGACTCCCGGTGTGTTTTCTTGAACTTCCATTTCCGGCGACCGAGAATGGACTTCCGGAGGGATGCGAAAGTGTAGATGCTCTACATTGTCTAGAATTACTTCCAAACTTTTCGGCTGCAGTTGACACGTTACAAGAACGACTCGAGCAACGGTTCGAATCGATTAACCCACGTCCGAACTGTATTATATCTGATAAATACATGGTCTGGAGTGATTATACAGCAGCAAAGTATGAGATTCCCAGGATCATATTCGATGGGATGAGTTGTTTCAAACAACTATCTACACATCATTTGTATGCATCTAAGGTGTTCGATGATATGCCTGAGTCAGAACCATTTGTTCTCCCTGGATTGCCTGACAAGATCGAGATCACCCGAGCTCAACTGCCAGCAGAGTTCAATCATAGCCGTGGTGTAATGCGTGAGCAAATTGAACGAGTGAGAGAAACCGAGTCGAGAGCTTACGGAATGGTGATCAATAGTTTTGAGGAGTTGGAGCAAGAGTATGTTAAGGAACTTAAGAAGTTTAAAGGCGGTAAGATTTGGTGTTTAGGACCGTTATCATTATCTAACAATCATGATTCGAGTACATCTATTGATGATCAACGTTACTTGAAATGGCTCGATTCTAAAGAACCAGGATCGGTTGTGTATGCCTGCTTCGGTAGCAGTAGTCAAGTCACGCCCCCACAACTCATCGAGCTTGGGTTAGCATTGGAAGCATCCAAATCCCCGTTCATATGGGTGATTCGAGCCGGTGACAAAGTTAAAGAGGTCGAGAAATGGTTAATAGAAAGTGAGTTTGAAACTCGAGTAAAAGACAGAGGTCTTGTAGTCAGAGACTGGGCACCACAAATCTTAATCTTGTCCCACCGTTCGGTTGGAGGGTTCTTGACACACTGCGGTTGGAATTCAGTGTTGGAAGGGGTTTCGGCTGGTGTCCCTATGATCACGTGGCCTTTGTTTGCAGAACAATTCTTGAACGAGAAGTCGATAGTTCAAGTTTTGGGTGTCGGTGTTAGTGTTGGTGCTCCCGGTGTGGTGCACTGGGGCCAAGAAGACGAATTTGGGGTCACCGTGAAAAGCGAGCAGGTGAAAACAGCTATAGAAACAGTAATGGATGTCGGATCCGAAGGAAATGAGAGAAGAAAGAAGGCGAAATCACTTGCAATGGTAGCAAAAAAAAGCCATTGA

MALESVNQLHFLLIPFLAPGHTIPMIHMAKLLAQRPNVMVTIVTTPVNAIRYGSTLQEHIESGLPVCFLELPFPATENGLPEGCESVDALHCLELLPNFSAAVDTLQERLEQRFESINPRPNCIISDKYMVWSDYTAAKYEIPRIIFDGMSCFKQLSTHHLYASKVFDDMPESEPFVLPGLPDKIEITRAQLPAEFNHSRGVMREQIERVRETESRAYGMVINSFEELEQEYVKELKKFKGGKIWCLGPLSLSNNHDSSTSIDDQRYLKWLDSKEPGSVVYACFGSSSQVTPPQLIELGLALEASKSPFIWVIRAGDKVKEVEKWLIESEFETRVKDRGLVVRDWAPQILILSHRSVGGFLTHCGWNSVLEGVSAGVPMITWPLFAEQFLNEKSIVQVLGVGVSVGAPGVVHWGQEDEFGVTVKSEQVKTAIETVMDVGSEGNERRKKAKSLAMVAKKSH

SrUGT73G2

ATGGCTTTGGAATCAGTCAACCAGCTTCACTTCCTCCTGATCCCCTTTCTAGCTCCTGGTCACACCATCCCCATGATCCACATGGCCAAATTGCTCGCACAACGACCAAATGTGATGGTCACCATCGTCACCACACCCGTAAACGCGATTCGATATGGTTCTACGCTTCAAGAACACATCGAATCCGGACTCCCGGTGTGTTTTCTTGAACTTCCATTTCCGGCGACCGAGAATGGACTTCCGGAGGGATGCGAAAGTGTAGATGCTCTACATTGTCTAGAATTACTTCCAAACTTTTCGGCTGCAGTTGACACGTTACAAGAACGACTCGAGCAACGGTTCGAATCGATTAACCCACGTCCGAACTGTATTATATCTGATAAATACATGGTCTGGAGTGATTATACAGCAGCAAAGTATGAGATTCCCAGGATCATATTCGATGGGATGAGTTGTTTCAAACAACTATCTACACATCATTTGTATGCATCTAAGGTGTTCGATGATATGCCTGAGTCAGAACCATTTGTTCTCCCTGGATTGCCTGACAAGATCGAGATCACCCGAGCTCAACTGCCAGCAGAGTTCAATCATAGCCGTGGTGTAATGCGTGAGCAAATTGAACGAGTGAGAGAAACCGAGTCGAGAGCTTACGGAATGGTGATCAATAGTTTTGAGGAGTTGGAGCAAGAGTATGTTAAGGAACTTAAGAAGTTTAAAGGCGGTAAGATTTGGTGTTTAGGACCGTTATCATTATCTAACAATCATGATTCGAGTACATCTATTGATGATCAACGTTACTTGAAATGGCTCGATTCTAAAGAACCAGGATCGGTTGTGTATGCCTGCTTCGGTAGCAGTAGTCAAGTCACGCCCCCACAACTCATCGAGCTTGGGTTAGCATTGGAAGCATCCAAATCCCCGTTCATATGGGTGATTCGAGCCGGTGACAAAGTTAAAGAGGTCGAGAAATGGTTAATAGAAAGTGAGTTTGAAACTCGAGTAAAAGACAGAGGTCTTGTAGTCAGAGACTGGGCACCACAAATCTTAATCTTGTCCCACCGTTCGGTTGGAGGGTTCTTGACACACTGCGGTTGGAATTCAGTGTTGGAAGGGGTTTCGGCTGGTGTCCCTATGATCACGTGGCCTTTGTTTGCAGAACAATTCTTGAACGAGAAGTCGATAGTTCAAGTTTTGGGTGTCGGTGTTAGTGTTGGTGCTCCCGGTGTGGTGCACTGGGGCCAAGAAGACGAATTTGGGGTCACCGTGAAAAGCGAGCAGGTGAAAACAGCTATAGAAACAGTAATGGATGTCGGATCCGAAGGAAATGAGAGAAGAAAGAAGGCGAAATCACTTGCAATGGTAGCAAAAAAAGCCATTGAAGAAGGGGGATCTTCTCACTATAACTTGATGTTACTAATACAAGATATATTGGAACATATAAATGCTAGAACCCATAAGCCAGAAACTACTTATTGTAGCTAA

MALESVNQLHFLLIPFLAPGHTIPMIHMAKLLAQRPNVMVTIVTTPVNAIRYGSTLQEHIESGLPVCFLELPFPATENGLPEGCESVDALHCLELLPNFSAAVDTLQERLEQRFESINPRPNCIISDKYMVWSDYTAAKYEIPRIIFDGMSCFKQLSTHHLYASKVFDDMPESEPFVLPGLPDKIEITRAQLPAEFNHSRGVMREQIERVRETESRAYGMVINSFEELEQEYVKELKKFKGGKIWCLGPLSLSNNHDSSTSIDDQRYLKWLDSKEPGSVVYACFGSSSQVTPPQLIELGLALEASKSPFIWVIRAGDKVKEVEKWLIESEFETRVKDRGLVVRDWAPQILILSHRSVGGFLTHCGWNSVLEGVSAGVPMITWPLFAEQFLNEKSIVQVLGVGVSVGAPGVVHWGQEDEFGVTVKSEQVKTAIETVMDVGSEGNERRKKAKSLAMVAKKAIEEGGSSHYNLMLLIQDILEHINARTHKPETTYCS

SrUGT73G3

ATGGCGTCGGAACCAGTCAACCAGCTTCACATCCTCATGATCCCCTTGCTAGCTCCGGGGCACACCATCCCCATGATCTCCATGGCTAAATTGCTCTCCCAGCGACAGAACGTGATGGTCACCATCGTCACCACACCACTAAACGCCGTCCGATACGGTCCCAAACTCCAACAACACATCAATTCCGACTTCCCGGTGCGATTCCTTGAACTCCCGTTTCCGGCGACCGAGAACGGACTACCGGAGGGAT

GCGAAAGTGCAGATTCTCTACCGGGTCTGCATCTACTTCCAAACTTTTCAGCTGCAGTTGACACGTTGCAACATAGACTCGAAGAACGATTTGATTCGCTAAACCCTCGACCCATCTGTATTATTTCTGATAAATTCATGCTCTGGAGTGATGATACAGCGACAAAGTATAAGATACCCAGAATCATATTTGATGGAATGAGTTGTTTCAAACAGTTAGCTACTCATCATCTGTATGCATCCAAGGTGTTCGATGAATTGCCTGAGTCAGACCCTTTTGTTCTCCCAGGGTTACCTGACCGAATCGAGTTAACTCGAGCTCAGCTTCCACCAGAGTTCAATGATAGTGGTTTTGCATCGAGTGAGCACCTTGAACGAGTGAGAGAAACCGAGTCCAGATCTTATGGAATGGTGATCAATACTTTTGAGGAATTAGAACAAGAATATGTTAAGGAATTCAAGAGGTTAAAAGGGGGTAAGGTTTGGTGTGTTGGACCATTATCACTATCTAACAATAATGATTCTAGTTACAATGTCTCTACCGATGACCAATCTTACTTAAAATGGCTCGATTCCAAAGACCCTGGTTCAGTCGTCTATGCCTGCTTCGGTAGCAGCAGTCAGATCATCCCTACACAACTCATCGAGCTCGGGTTAGCCCTAGAAGCATCCGGATCCCCATTCATATGGGTGATCAGATCCGGTGACAAAGCTAACGAGATTGAAAAATGGTTATCAGAAAGCAGGTTTGAAACCAGAATCAAAGATAGAGGACTCATAGTCAGAAACTGGGCACCACAATCCCTGATCTTATCACACCATTCGGTTGGTGGGTTCTTGACGCATTGCGGTTGGAATTCAATCTTGGAAGGTGTGTCTGTTGGGGTCCCTATGATCACATGGCCTCAGTTTGCAGAACAGTTTTTGAATGAGAAGTTGATTGTTCAAGTTTTGGGGATTGGTGTGAGTGTTGGTGCTTCGGTTATGGTTCATTGGGGTCAAGAAGACAAATTTGGGGTTACCGTGAAGAGTGAGCAGGTGAAAATGGCTATAGAAACAGTAATGGATGTGGGGCCCGAAGGAAATGCGAGAAGATTGAAAGCAAAGGCGATCGGGATGGTAGCAAAGAAAGCGGTTGAAGAAGGAGGATCTTCTCACCATAACTTGAGTTTACTACTACAAGATCTATTGGAAGTTTCAAATGTTAGAAGCCAAAAGCCATTGATTTGA

MASEPVNQLHILMIPLLAPGHTIPMISMAKLLSQRQNVMVTIVTTPLNAVRYGPKLQQHINSDFPVRFLELPFPATENGLPEGCESADSLPGLHLLPNFSAAVDTLQHRLEERFDSLNPRPICIISDKFMLWSDDTATKYKIPRIIFDGMSCFKQLATHHLYASKVFDELPESDPFVLPGLPDRIELTRAQLPPEFNDSGFASSEHLERVRETESRSYGMVINTFEELEQEYVKEFKRLKGGKVWCVGPLSLSNNNDSSYNVSTDDQSYLKWLDSKDPGSVVYACFGSSSQIIPTQLIELGLALEASGSPFIWVIRSGDKANEIEKWLSESRFETRIKDRGLIVRNWAPQSLILSHHSVGGFLTHCGWNSILEGVSVGVPMITWPQFAEQFLNEKLIVQVLGIGVSVGASVMVHWGQEDKFGVTVKSEQVKMAIETVMDVGPEGNARRLKAKAIGMVAKKAVEEGGSSHHNLSLLLQDLLEVSNVRSQKPLI

SrUGT73G4

ATGGTAGCCAACAAACTCCATTTTCTTTTGGTTCCCTACATAGGTCCTGGCCTTACAATTCCCATGATCGACATGGCCAAACTGCTTGCAAAACAACCGAATGTTACTGTCACCATCGTCACCACACCTCTCAACGCCATCCGCTACGGTGCCAGTCTCGCCGGAGCAATCACCGCCGGACTTCCCGTCCGATTTCTTGAACTCCCGTTTCCGGCAGCAGAGGCTGGCTTGCCTGAAGGGTGTGAAAGTTCAGATAAAATCCCTAGTAGGGATTTAATTCCAAACTTTTTAGCTGCGGTTGACATGTTACAGCAAAAGCTTGAAGAACGATTCGAAATGATAAACCCTCGTCCGAATTGTATCATATCTGATAAGTATATGTCATGGACGGGTGATTTTGCTGATAAGAATCGGTTACCAAGAATCATGTTTGATGGGACGAGTTGTTTTAATGAGTTGTGTTACAATAATTTGTTTGCATGTAAGGTGTTCGACGATTTGCCTGACTCAAAACCATTTGTTGTCCCGGGTTTGCCCGATCGGGTCGAGCTGACAAGAAACCAGCTTCCACCTGAGTTCAACCCGAGTTCGATTGACACGAGTGCGTTTCGCGAGCGGGCTAGAGATGCTGAATTGAGGGCTTATGGTGTGGTGGTCAATAGTTTTGAGGAACTAGAACAAGAATACGTTAACGAGTATAAGAAGTTAAGAGGGGGTAAAGTTTGGTGTATCGGACCATTGTCGCTATGTGATAGTGACGATTCGGTTAAATCTCAAAGAGGAAACGTAGCCTCGATTGGTGAAGAACAATGCCTAAAATGGCTCGATTCTCACGAACCAGATTCAGTAGTTTACGCGTGTTTTGGTAGTCTTGTGAGGATTAACACCCCACAACTTATTGAGCTTGGTTTAGGCCTTGAAGCGTCAAACCACCCGTTCATTTGGGTGATTAATACCGTTCATAGAGAGAAAGAGGTCGAGGAATGGTTAGCAGAAAGTGGGTTCGAGGAGCGGGTCAAACATAGAGGGTTAATAATCCGGGGGTGGGCCCCACAATTGGTAATCTTGTCACATCCTTCTGTTGGAGGGTTCTTAACACATTGTGGTTGGAACTCGACTCTAGAATCCGTGTCTGCTGGTGTTCCCATGATCACATGGCCACAGTTTGCAGAGCAGTTTATTAACGAGAAACTTGTGGTCCAAGTTCTGGGGATCGGTTTCAGTGTTGGAGCTGAATCTGTTGTTCATGTGGTCGAAGAAGATCGGTTTGGAGTGAAAGTTAAGAGTGAGAGTGTGAAGAATGCTATCGAGCGGGTGATGAATGATGAGATTGAAGGAAATGAGGGACGAAAGAGAGCTATAGAGGTCGCTATAATGGCGAATAACGCGATAAAAGAGGAAGGTTCTTCACACTTGAACTTGACCCTGTTGATTCAAGACATAATACACCTTGTAAATACTTCAAGTTAA

MVANKLHFLLVPYIGPGLTIPMIDMAKLLAKQPNVTVTIVTTPLNAIRYGASLAGAITAGLPVRFLELPFPAAEAGLPEGCESSDKIPSRDLIPNFLAAVDMLQQKLEERFEMINPRPNCIISDKYMSWTGDFADKNRLPRIMFDGTSCFNELCYNNLFACKVFDDLPDSKPFVVPGLPDRVELTRNQLPPEFNPSSIDTSAFRERARDAELRAYGVVVNSFEELEQEYVNEYKKLRGGKVWCIGPLSLCDSDDSVKSQRGNVASIGEEQCLKWLDSHEPDSVVYACFGSLVRINTPQLIELGLGLEASNHPFIWVINTVHREKEVEEWLAESGFEERVKHRGLIIRGWAPQLVILSHPSVGGFLTHCGWNSTLESVSAGVPMITWPQFAEQFINEKLVVQVLGIGFSVGAESVVHVVEEDRFGVKVKSESVKNAIERVMNDEIEGNEGRKRAIEVAIMANNAIKEEGSSHLNLTLLIQDIIHLVNTSS

SrUGT73H1

ATGGCTTCTGATCTTCACTTTCTTGTCATACCGTTAATGTGCCCCGGTCATCTCATTCCGATGATCGACATGGTCAAACTCATCGCACAACATTCCGTCACCGTCACCATCGTCATCACTCCGCGAAACGCCGACCGCTACGGTGCCGTCCTCCGGCGAGCCATAGATTCCGGCCTTCCGATCAAACTCCTCCAGATCCGGTTTCCAGCCTCCGACCACGGTCTACCCGACGGATGTGAAAGTGTTGACGATCTACCAACATTTAACCTTTCGAAAAACTTCTTCGATGCATTAGCGACACTTCAAAAACCACTTGAAGATGCGTTAGAGTTTAATGAGCTTGAATCTAAACCAAATTGCATCATTTCCGACAAACATTTAACTTGGACAGCTGATGTCGCTAAAAAGTTTGAAATTCCTTGGGTTATATTCGACGGGATGAGTTGTTTCACTCAATTAGCGACACACAATCTATGTGTTTCCAAGATTTATGAAACGGTAACGGATTTTGATTCTGACATGTTTATCTTGCCCGGGTTACCGGATAAGATATCCATGACCAAATCACAGCTTCCTGGATTGTTTAATCCGGGAAGCAGTGTGAAAGCAAAGGAATTGTTATCCGTGCGCGAAAAGGTTCGCGCAGCAGAGCTAGAAGCGTACGGGACGCTTATAAACAGTTTTGAAGAGTTGGAAACACGGTATATCAATGAGTTTAAGATGCTAAAACAAGGTAAGGTTTGGTGTATTGGTCCGTTTTCGAATTTGAACACGAACGATTTGGATCGAGCACAACGCGGAACCAAAGATTCAATCAACAATGAATGCGTTACATGGCTCGATTCACAACAAAACCGGAGTGTAATCTATGCATCTTTAGGAAGCCTTACTCGGCTAACACCACTACAGTTTATCGAGCTCGCATTAGGGTTAGAAGATTCGCAATTCCCATTTATTTTGGTGGTGAAAGGTGGAAGTAGGACAAAAGAGATAGAGAAATGGTTAGATGAAAGTGGGTACGAGTCGCGAGTGAAAGGAAGAGGGTTTTTGATCCGCGGTTGGGCTTCACAAGTCCTGATCTTATCGGACAGAGCCGTGGGTGCGTTCTTGACTCATTGCGGTTGGAACTCGACTATCGAAGGGGTTTGTGCCGGTGTACCGATGATCACATGGCCGCAGTTTGCCGAACAGTTTTTTAATGAAAGATTGGTTGTTCAAGTGTTGGGTATTGGTGTTGAAGTTGGGGCTCAAAGAGTGATACAATTAGGTGAAGAAGATGAATCCCAAATGCAAGTGAAAAGAGATGATGTTTGTAGATCTCTAAAGAAAATTATGGATGAAGGTGAAGAAGGTGAAGAAAGGAGAAATAAGGCAAATTATTTGAAACAAATGGCTGAAAAAGCTATAGAAGAAGGGGGTTCATCCCAAGTTAATTTGAACCGGTTCATTGAAGATATTAGGATAAGCACAAATAAGGGCCATGTAGGCTAA

MASDLHFLVIPLMCPGHLIPMIDMVKLIAQHSVTVTIVITPRNADRYGAVLRRAIDSGLPIKLLQIRFPASDHGLPDGCESVDDLPTFNLSKNFFDALATLQKPLEDALEFNELESKPNCIISDKHLTWTADVAKKFEIPWVIFDGMSCFTQLATHNLCVSKIYETVTDFDSDMFILPGLPDKISMTKSQLPGLFNPGSSVKAKELLSVREKVRAAELEAYGTLINSFEELETRYINEFKMLKQGKVWCIGPFSNLNTNDLDRAQRGTKDSINNECVTWLDSQQNRSVIYASLGSLTRLTPLQFIELALGLEDSQFPFILVVKGGSRTKEIEKWLDESGYESRVKGRGFLIRGWASQVLILSDRAVGAFLTHCGWNSTIEGVCAGVPMITWPQFAEQFFNERLVVQVLGIGVEVGAQRVIQLGEEDESQMQVKRDDVCRSLKKIMDEGEEGEERRNKANYLKQMAEKAIEEGGSSQVNLNRFIEDIRISTNKGHVG

SrUGT73I1

ATGGCTTCCCCCAAAGACCTTCACTTTGTCTTGTTTCCCTTAATGCAACAAGGTCACATGATACCCATGATCGATATCGCCAGAATACTAGCCCACCGTAACGCCACCGTCACAATCATCACAACTCCGGTCAACGCCACCCGGTTCCGTCCAGCCATCGCTCGTGCAACCGAATCCAAACTCAACATCCAAGTTATTGAACTCGAACTCCGGCTAGCCGATGTCGGGTTACCCGAAGGCTGCGAAAATTTCGACATGCTTCCATCTGCTGATCTCTTTGGCAAAATGTACGCAGCGATGGACTTGTTGGAACACCCGTCGGAAAATGTTCTCCGGCGACTAACTCCGGCACCAAGCTGCATCATCTCCGATAACCAGTTTCCGTGGACAACTGATCTGGCTCGCCGGTTCGGTATCCCGAGGATTGTTTTTCATGGACCTGGATGCTTCACTTACTTGTGTTTAGACATAGTGATGAATACCAACATCCTAAATGAAATCGGGTCTGATTCGGATTACTTTATCCTCCCGGGAATCCCTGACCGGATTGAGATCACAAAAGCGCAAGCCAGCGGTTGGGGAAAACGAGAGACGAAACAGATGGAGAAGTTTTTTGAACGAATGGAGGTCGCGGCAGATACCGCGTATGGGATTGTGGTAAATAGTTTTGACGAGTTGGAGTCGAACTATGTTAAACGACTCGAAGAAGCGAAAGGTAAAAAAATATGGTGCATTGGGCCGGTTTCATTGCACAATCGCGGTTTTCTTGATCTAGCAGAGAGAGGAAACAAGGCGGGAATCGACGAGCATGATTGCGTAAAATGGTTAGACACGAAAGATGCACGGTCTGTGATCTTTGTTTGTTTAGGAAGTATGTCGAGTATGTGTCACGAGCAAATACTCGAGCTCGGATTGGGATTGGAGTTATCCAACGTACAGTTTATTTGGTGTATAAGATCGATTACAGACGAGACGGGGAGATGGTTGTTGGGATATGAACAACGGGTGAAAGATAGAGGGTTAATTGTTCGTGGTTGGGCCCCACAGGTCTTGATACTATCCCATCAATCAGTTGGTGGGTTCATGACTCATTGTGGTTGGAACTCGACTCTTGAAGGGGTTTCAGCCGGGGTCCCGATGGTAACATATCCGCAATTTGCGGATCAGTTTTTGAATGAAAGATTTTTGGTAGATGTGTTGAAAACAGGAGTGAGTATTGGTATGAAGGTGGTTGTTGGTGTTGGGGAGCAAGATAAGCATGGAGTGTTGGTGAAGAAAGAAAACATTAAGATGGCTGTTGAAAGTGTAATGGACGATAACGAGGAAGGAAAAGCGAGGAGAGATCGAGCTAGAGAGCTCGGGGAAATGGCAAAGAGAGCAATGACCGAAGGGGGTTCTTCGTATGTCAACACGACGTTGATGATTCAAGATGTTATTGATGAATTAGCCAAGAACACAAAGCCGATTTAA

MASPKDLHFVLFPLMQQGHMIPMIDIARILAHRNATVTIITTPVNATRFRPAIARATESKLNIQVIELELRLADVGLPEGCENFDMLPSADLFGKMYAAMDLLEHPSENVLRRLTPAPSCIISDNQFPWTTDLARRFGIPRIVFHGPGCFTYLCLDIVMNTNILNEIGSDSDYFILPGIPDRIEITKAQASGWGKRETKQMEKFFERMEVAADTAYGIVVNSFDELESNYVKRLEEAKGKKIWCIGPVSLHNRGFLDLAERGNKAGIDEHDCVKWLDTKDARSVIFVCLGSMSSMCHEQILELGLGLELSNVQFIWCIRSITDETGRWLLGYEQRVKDRGLIVRGWAPQVLILSHQSVGGFMTHCGWNSTLEGVSAGVPMVTYPQFADQFLNERFLVDVLKTGVSIGMKVVVGVGEQDKHGVLVKKENIKMAVESVMDDNEEGKARRDRARELGEMAKRAMTEGGSSYVNTTLMIQDVIDELAKNTKPI

SrUGT74G1-1

ATGGCGGAACAACAAAAGATCAAGAAATCACCACACGTTCTACTCATCCCATTCCCTTTACAAGGCCATATAAACCCTTTCATCCAGTTTGGCAAACGATTAATCTCCAAAGGTGTCAAAACAACACTTGTTACCACCATCCACACCTTAAACTCAACCCTAAACCACAGTAACACCACCACCACCTCCATCGAAATCCAAGCAATTTCCGATGGTTGTGATGAAGGCGGTTTTATGAGTGCAGGAGAATCATATTTGGAAACATTCAAACAAGTTGGGTCTAAATCACTAGCTGACTTAATCAAGAAGCTTCAAAGTGAAGGAACCACAATTGATGCAATCATTTATGATTCTATGACTGAATGGGTTTTAGATGTTGCAATTGAGTTTGGAATCGATGGTGGTTCGTTTTTCACTCAAGCTTGTGTTGTAAACAGCTTATATTATCATGTTCATAAGGGTTTGATTTCTTTGCCATTGGGTGAAACTGTTTCGGTTCCTGGATTTCCAGTGCTTCAACGGTGGGAGACACCGTTAATTTTGCAGAATCATGAGCAAATACAGAGCCCTTGGTCTCAGATGTTGTTTGGTCAGTTTGCTAATATTGATCAAGCACGTTGGGTCTTCACAAATAGTTTTTACAAGCTCGAGGAAGAGGTAATAGAGTGGACGAGAAAGATATGGAACTTGAAGGTAATCGGGCCAACACTTCCATCCATGTACCTTGACAAACGACTTGATGATGATAAAGATAACGGATTTAATCTCTACAAAGCAAACCATCATGAGTGCATGAACTGGTTAGACGATAAGCCAAAGGAATCAGTTGTTTACGTAGCATTTGGTAGCCTGGTGAAACATGGACCCGAACAAGTGGAAGAAATCACACGGGCTTTAATAGATAGTGATGTCAACTTCTTGTGGGTTATCAAACATAAAGAAGAGGGAAAGCTCCCAGAAAATCTTTCGGAAGTAATAAAAACCGGAAAGGGTTTGATTGTAGCATGGTGCAAACAATTGGATGTGTTAGCACACGAATCAGTAGGATGCTTTGTTACACATTGTGGGTTCAACTCAACTCTTGAAGCAATAAGTCTTGGAGTCCCCGTTGTTGCAATGCCTCAATTTTCGGATCAAACTACAAATGCCAAGCTTCTAGATGAAATTTTGGGTGTTGGAGTTAGAGTTAAGGCTGATGAGAATGGGATAGTGAGAAGAGGAAATCTTGCGTCATGTATTAAGATGATTATGGAGGAGGAAAGAGGAGTAATAATCCGAAAGAATGCGGTAAAATGGAAGGATTTGGCTAAAGTAGCCGTTCATGAAGGTGGTAGCTCAGACAATGATATTGTCGAATTTGTAAGTGAGCTAATTAAGGCTTAA

MAEQQKIKKSPHVLLIPFPLQGHINPFIQFGKRLISKGVKTTLVTTIHTLNSTLNHSNTTTTSIEIQAISDGCDEGGFMSAGESYLETFKQVGSKSLADLIKKLQSEGTTIDAIIYDSMTEWVLDVAIEFGIDGGSFFTQACVVNSLYYHVHKGLISLPLGETVSVPGFPVLQRWETPLILQNHEQIQSPWSQMLFGQFANIDQARWVFTNSFYKLEEEVIEWTRKIWNLKVIGPTLPSMYLDKRLDDDKDNGFNLYKANHHECMNWLDDKPKESVVYVAFGSLVKHGPEQVEEITRALIDSDVNFLWVIKHKEEGKLPENLSEVIKTGKGLIVAWCKQLDVLAHESVGCFVTHCGFNSTLEAISLGVPVVAMPQFSDQTTNAKLLDEILGVGVRVKADENGIVRRGNLASCIKMIMEEERGVIIRKNAVKWKDLAKVAVHEGGSSDNDIVEFVSELIKA

SrUGT74G1-2

ATGGCGGAACAACAAAAGATCAAGAAATCACCACACGTTCTACTCATCCCATTCCCTTTACAAGGCCATATAAACCCTTTCATCCAGTTTGGCAAACGATTAATCTCCAAAGGTGTCAAAACAACACTTGTTACCACCATCCACACCTTAAACTCAACCCTAAACCACAGCAACACCACCACCACCTCCATCGAAATCCAAGCAATTTCCGATGGTTGTGATGAAGGCGGTTTTATGAGTGCAGGAGAATCATATTTGGAAACATTCAAACAAGTTGGGTCTAAATCACTAGCTGACTTAATCAAGAAGCTTCAAAGTGAAGGAACCACAATTGATGCAATCATTTATGATTCTATGACTGAATGGGTTTTAGATGTTGCAATTGAGTTTGGAATCGATGGTGGTTCGTTTTTCACTCAAGCTTGTGTTGTAAACAGCTTATATTATCATGTTCATAAGGGTTTGATTTCTTTGCCATTGGGTGAAACTGTTTCGGTTCCTGGATTTCCAGAGCTTCAACGGTGGGAGACACCGTTAATTTTGCAGAATCATGAGCAAATACAGAGCCCTTGGTCTCAGATGTTGTTTGGTCAGTTTGCTAATATTGATCAAGCACGTTGGGTCTTCACAAATAGTTTTTACAAGCTCGAGGAAGAGGTAATAGAGTGGACGAGAAAGATATGGAACTTGAAGGTAATCGGGCCAACACTTCCATCCATGTACCTTGACAAACGACTTGATGATGATAAAGATAACGGATTTAATCTCTACAAAGCAAACCATCATGAGTGTATGAACTGGTTAGACGATAAGCCAAAGGAATCAGTTGTTTACGTAGCATTTGGTAGCCTGGTGAAACATGGACCCGAACAAGTGGAAGAAATCACACGGGCTTTAATAGATAGTGATGTCAACTTCTTGTGGGTTATCAAACATAAAGAAGAGGGAAAGCTCCCAGAAAATCTTTCGGAAGTAATAAAAACCGGAAAGGGTTTGATTGTAGCATGGTGCAAACAATTGGATGTGTTAGCACACGAATCAGTAGGATGCTTTGTTACACATTGTGGGTTCAACTCAACTCTTGAAGCAATAAGTCTTGGAGTCCCCGTTGTTGCAATGCCTCAATTTTCGGATCAAACTACAAATGCCAAGCTTCTAGATGAAATTTTGGGTGTTGGAGTTAGAGTTAAGGCTGATGAGAATGGGATAGTGAGAAGAGGAAATCTTGCGTCATGTATTAAGATGATTATGGAGGAGGAAAGAGGAGTAATAATCCGAAAGAATGCGGTAAAATGGAAGGATTTGGCTAAAGTAGCCGTTCATGAAGGTGGTAGCTCAGACAATGATATTGTCGAATTTGTAAGTGAGCTAATTAAGGCTTAA

MAEQQKIKKSPHVLLIPFPLQGHINPFIQFGKRLISKGVKTTLVTTIHTLNSTLNHSNTTTTSIEIQAISDGCDEGGFMSAGESYLETFKQVGSKSLADLIKKLQSEGTTIDAIIYDSMTEWVLDVAIEFGIDGGSFFTQACVVNSLYYHVHKGLISLPLGETVSVPGFPELQRWETPLILQNHEQIQSPWSQMLFGQFANIDQARWVFTNSFYKLEEEVIEWTRKIWNLKVIGPTLPSMYLDKRLDDDKDNGFNLYKANHHECMNWLDDKPKESVVYVAFGSLVKHGPEQVEEITRALIDSDVNFLWVIKHKEEGKLPENLSEVIKTGKGLIVAWCKQLDVLAHESVGCFVTHCGFNSTLEAISLGVPVVAMPQFSDQTTNAKLLDEILGVGVRVKADENGIVRRGNLASCIKMIMEEERGVIIRKNAVKWKDLAKVAVHEGGSSDNDIVEFVSELIKA

SrUGT74G2

ATGGCGGAACAAAAGAAGATGAACAGATCACCACATGTTCTACTCGTCCCATTCCCTTCACTAGGCCATATAAACCCTTTCATCCAATTTGGCAAACGCTTGATCTCCAAAGATGTCAAAACAACACTCGTTACCACCATTTACATCAGAAACTCTAGCCTCCAAACCAACAACAACAGCTCCATTAAAACCGAAGCAATTTCCGATGGTTTCGATGAAGGCGGTTACCCGAGTCACGATGTTCAAATCCATGAATCCTACCTCAAAACCTTCAAACAAGTTGGGTCCAAATCACTAGCTGATTTGATCAACAAGTTTCAAAGTGAGGGAACCACCATTAGTGCAATCATCTATGATTCAGTCATTCCATGGGCTTTGGATGTTGCAATTGAGTTTGGAATTGATGGGGGTTCGTTTTTCACTCAAGCTTGTGTTGTAAACAGCATATATTATCATGTTCATAAGGGTTTGATTTCTTTGCCATTGGGTGCAACTGTTTCGGTTCCTGGATTTCCAGAGCTTCAACGGTGGGAGACACCGTCTGCTGTACAGAATCATGAGCAATTACACAGCCCTTGGTCTCAGATTCTTTTCGGTCATCAGTTTGCTAATATTCATCAAGTACGTTGGGTTTTCGCAAATACTTTTTACCAACTCGAGGAACAGGTAATAGAGTGGACGAGAAAGATGTGGAACTTGAAGGTGATCGGGCCAACGGTTCCATCTATGTACCTTGACAAACGCCTAAATGATGATAAGGATTATGGGTTAAATCTCTTTAATGCGAACCATAATGAATGCATGAACTGGCTAGACGATAAGCCAAAGGAATCTGTTGTTTACGTATCATTTGGTAGCATGGCGAAACATGGACCCGAACAAGCGGAAGAAATCACATTGGCTTTGATAGATAGTGATGTCAACTTCTTGTGGGTTCTCAAAGATAAAGTAGAGGCAAAACATCCAAATGATCTTTTGGATGTAATAAAAAGTGGAAAGGGCTTGGTTGTGTCATGGTGCAAACAATTGGATGTGTTAGCACACGAAGGAGTAGGATGCTTTGTTACACATTGTGGGTTCAACTCAACTCTTGAAGCAATAAGTCTTGGAGTCCCCGTTGTTGCAATGCCTCAATGGTCAGATCAAACTACAAATGCCAAGCTTCTAGATGAAATTTTGGGTGTTGGAGTTAGAGTTAAAGCTGATGAGAAAGGGATAGTAAGAAGAGAAAATCTAGTATCATGTATTAAGACAATTATGGAGGACAAGAAAGAAACGCGAAAGAACACGGTAAAATGGAGGGATTTGGCAAAAGGAGCCGTTGACAAAGGTGGTAGCTCGGACAAGGATATTGATGAGTTTGTATCTGAGCTAATCAAGGCATAA

MAEQKKMNRSPHVLLVPFPSLGHINPFIQFGKRLISKDVKTTLVTTIYIRNSSLQTNNNSSIKTEAISDGFDEGGYPSHDVQIHESYLKTFKQVGSKSLADLINKFQSEGTTISAIIYDSVIPWALDVAIEFGIDGGSFFTQACVVNSIYYHVHKGLISLPLGATVSVPGFPELQRWETPSAVQNHEQLHSPWSQILFGHQFANIHQVRWVFANTFYQLEEQVIEWTRKMWNLKVIGPTVPSMYLDKRLNDDKDYGLNLFNANHNECMNWLDDKPKESVVYVSFGSMAKHGPEQAEEITLALIDSDVNFLWVLKDKVEAKHPNDLLDVIKSGKGLVVSWCKQLDVLAHEGVGCFVTHCGFNSTLEAISLGVPVVAMPQWSDQTTNAKLLDEILGVGVRVKADEKGIVRRENLVSCIKTIMEDKKETRKNTVKWRDLAKGAVDKGGSSDKDIDEFVSELIKA

SrUGT74G3

ATGGCGGAACAACACAAGATCAACAAATCACCGCATGTTCTACTCTTCCCCTACCCCGCACAAGGCCATATAAACCCTCTCATCCAATTTGGCAAACGTTTGATCTCCAAAGGTGTCAAAACAACACTCGTTACTACCGTCTTCCTCTTAAACTCTAACCTAACCAACACAGAAAACACCACCTCCATTAAAATCGAAGCTATTTCAGATGGTTTCGATGAAGGTGGATACAATAGTGCAGATAGCTCTGAAATCTACCTCGAAACGTTCAAAGAAGTTGGATCCAAATCACTAGCAGATCTAATCAAGAAACTTCAAAGTGAGGGGAACACTATTGATGCAATTATCTATGATTCCTTCATTCCATGGGCTTTGGATGTTGCAATCGAGTTGGGTATTAATGGTGGTTCTTTTTTCACTCAAGCTTGTGGTGTAAACATCATATATTATCATGTTCATAAGGGTTTGATCTCTTTGCCATGTGGTTCAACTGTTTCGGTTCCAGGGTTGCCGGAACTTAAACACTGGGAGACACCGTCTTTTGTACATAATTACGGACCATATCCTGGTTGGTCTAAGACTGTGTTTAGTCAGTTTGATAATATTGATCAAGCACGTTGGGTCTTCACAAATAGTTTTTACGAACTTGAGGCACAGGTGATAGAGTGGATGAGAAAGAAGTGGAACTTGAAGGTGATTGGGCCAACACTTCCATCTATGTACCTTGACAAACGCCTTGAAGATGATAAAGATTACGGGTTCAATCTCTATAAAGCAAACCATAACGAATGCATGAATTGGTTAAACAATAAGCCAAAGGAATCCGTTGTTTACGTTTCGTTTGGAAGCAGTGCAAAACTTGAACCCGAACATATGGAAGAAATGGCATGGGGTTTGATTGATAGCAATATGAACTTCTTATGGGTTGTTAGGGCCGAAGAAGAAGAAAAACTCCCAAAAGAATTTGTGCACCAAAAATTATCTGGAAAAGGTATGGTTGTAGCATGGTGTAGACAATTGGATGTGTTAGCACACGAATCAGTAGGTTGCTTTGTTACGCATTGTGGGTTTAACTCAACTCTTGAAGCGATAAGTCTTGGAGTCCCGGTTGTAGCAATGCCGCAATGGACAGATCAAATTACAAATGCCAAGTTTATAGATGAAATTTGGGGTGTTGGAGTTAGAGTTAAGGCTGATGAGAATGGGATCGTGAGACGTGAAAATCTAGCATCGTGTATTAAGACGATTATGGAGGATGAAAGAGGTGTAATAGTCCAAAAGAAAACGATAAAATGGAGGGATTTGGCTAAATTAGCCGTTGATAAGGGTGGTAGCTCGGAAAAGGATATTGATGAATTTCTATCTGAGCTACTCAGGGAATAA

MAEQHKINKSPHVLLFPYPAQGHINPLIQFGKRLISKGVKTTLVTTVFLLNSNLTNTENTTSIKIEAISDGFDEGGYNSADSSEIYLETFKEVGSKSLADLIKKLQSEGNTIDAIIYDSFIPWALDVAIELGINGGSFFTQACGVNIIYYHVHKGLISLPCGSTVSVPGLPELKHWETPSFVHNYGPYPGWSKTVFSQFDNIDQARWVFTNSFYELEAQVIEWMRKKWNLKVIGPTLPSMYLDKRLEDDKDYGFNLYKANHNECMNWLNNKPKESVVYVSFGSSAKLEPEHMEEMAWGLIDSNMNFLWVVRAEEEEKLPKEFVHQKLSGKGMVVAWCRQLDVLAHESVGCFVTHCGFNSTLEAISLGVPVVAMPQWTDQITNAKFIDEIWGVGVRVKADENGIVRRENLASCIKTIMEDERGVIVQKKTIKWRDLAKLAVDKGGSSEKDIDEFLSELLRE

SrUGT74G4

ATGGCGGAACTGAAGAAATCACCACACGTTCTGCTCTTCCCCTTCCCTATACAAGGACATATAAATCCTCTCTTCCAGTTCGGGAAACGCTTACTATCAAAAGGCATCAAAACAACACTCGTTCCTACCATCTACATCAAATCCACCCTCATCCGTAACAACACCACCATTTCCGCTGGAATCGAACCCATTTCTGATGGTTTTGATGAAGGAGGTTTCGTTAGTGCAGATACCACCGAATCCTACCTTGAAACCTTCAAACAAGTTGGGTCCAAATCACTATCTGACTTGATCAAGAAGCTTCAAAGTCAGGGAACCACCATTGATGCAATCATCTACGATTCCTTCATCACATGGGCTTTGGATGTCGCCATGGAGTTTGGAATCAACGGTGCTTCGTTTTTCACTCAAGCTTGTGCGGTAAACAGCATATATTATCATGTTCATAAGGGTTTGATTTCTTTGCCACCGGGTCCAACTGTTTCGGTTCCCGGGTTGCCCCAGATTGAATGGTGGGAGACACCGTCTTTTGTGCACAATTATGGAACATACCCTGGTTTGTGTGATATGGTGTTCAATCAGTTCGCTAACATTCGTCAAGCACGTTGGGTCTTCACAAATACATTCTACCAACTCGAGCAAGAGGTAATAGAGTGGATGAGAAAGATGTGGAAGTTAAAAGTAATCGGGCCAACAATCCCATCAATGTACCTTGACAAACAACTTGAGGATGATAAAGATTATGGGTTCAATATCTTCAAACCAAACCTCAAGGAATGCATGAATTGGCTAAATGATAAGCCAAAAAAATCAGTTGTTTACGTATCTTTTGGGAGCTTGGCACAACTTGGACCCGAGCAAATGGAGGAAATTGCATGGGGTTTGAGTGATAGCAATGTGAACTTCTTGTGGGTTGTTAGGACAGAAGAAAAGGAAAAACTCCCAAAAGAATTTGTAGAGAAAAATCTATCTGGAAAGGAGGGTTTGGTTGTTTCATGGTGTAAACAATTGGACGTATTAGCACACGAATCAACCGGATGCTTTGTTACACATTGCGGGTTTAACTCAGTTCTTGAGGCAATAAGTCTGGGAGTGCCGGTGGTGGGAATGCCACAATGGACAGATCAAACTACAAATGCCAAACTTCTTGATGAAATCTGGGGTGTTGGAGTAAGAGTTAAAGCTGATGAGAAAGGGATAGTGAGAAGAGGAGATCTGGTATCATGTATTAAGAAAATCATGGAAGAAGAAGAAGGAGTAGTGATCCGAAAGAATGCGGCAAAATGGATGGATTTGGCTAAATCAGCTTTTGATGAAGGTGGGAGCTCTCGCAAGGATATTGATGAATTTGTGGGTGAACTAAAGCATGAATGTTGTTTACCAACGCACAAAGGGGATACATAA

MAELKKSPHVLLFPFPIQGHINPLFQFGKRLLSKGIKTTLVPTIYIKSTLIRNNTTISAGIEPISDGFDEGGFVSADTTESYLETFKQVGSKSLSDLIKKLQSQGTTIDAIIYDSFITWALDVAMEFGINGASFFTQACAVNSIYYHVHKGLISLPPGPTVSVPGLPQIEWWETPSFVHNYGTYPGLCDMVFNQFANIRQARWVFTNTFYQLEQEVIEWMRKMWKLKVIGPTIPSMYLDKQLEDDKDYGFNIFKPNLKECMNWLNDKPKKSVVYVSFGSLAQLGPEQMEEIAWGLSDSNVNFLWVVRTEEKEKLPKEFVEKNLSGKEGLVVSWCKQLDVLAHESTGCFVTHCGFNSVLEAISLGVPVVGMPQWTDQTTNAKLLDEIWGVGVRVKADEKGIVRRGDLVSCIKKIMEEEEGVVIRKNAAKWMDLAKSAFDEGGSSRKDIDEFVGELKHECCLPTHKGDT

SrUGT75E1-1

ATGGCTCAATCTGATCAACCTCAACCACACTTCCTCGTAGTAACCTTTCCAGCCCAAGGTCACATTAACCCGGCTCTACAGTTCGCAAAACAACTCGCCCGGTTCGATGTTAAACTCACCTTCACCACCACCGTCTCCGCCTACCGTGGCATGACCAAATTTGACCAGATTTACAATAATTTCAACTTTGTTGTTTATTCAGACGGCTTCGATGACGGTTTCACTTCTAAATCCGTTGACCCTGATCTCTTCATGACTCAGTTTAGGACCCGGGGAATCCAATCCTTGAAAGAAACCATAACTTCTAGTGCTGAAAATGGCACACCGGTCACATGTTTGGTGTACACCTTCCTCCTACCTTGGGCTGCAGAACTGGCGCGTGAACTTAACGTGCTACCAGCCCTTCTTTGGATCCAACCAGCATCGGTGTTTCGTGTGTACTATTATTATTTCAATGGGTATGATAAACTCATTGGTGAAGATTGTACCGAGTCTTCATGGTCCATCGAGTTACCGGGGCTACCATCGCTCAAAAGTTGTGATTTACCCTCGTTTTGTCTCCCTTCGAGCCCTTATGATACGGTGTTATCTTTGTTTAAGGAACTGCTTCATACGTTGAGTTCGTTTGAAAAGCCGAAGATACTTGCGAATACGTTTGATGCGTTGGAAGAAGAGGCCTTGAAAGAGATTGATGGCAAGATAAACATGGTTGGTGTTGGACCGTTGATTCCATCGGCTTTCTTGGATGGAAACGAGCAATCGGATAGATCTTTTGGAGGAGATATGTTTGACAAATCACATGATTGTTTGGAATGGATGAACACAAATCATGAAGGATCCATTGTTTACGTATCTTTTGGTAGTCTTATAGAATTGTCAAAGAAAGAAAAGGAGTCAATAGCTTGTGGTTTGTTGGAGAGCAAAAGGCCTTTTTTGTGGGTGATGAGAGATAAAGATGGAGAAGCAAAAGAAAAGGAAGATGAAATAAGTTGCATAGAGGAATTGAAACAATTAGGTTTGATAGTTCCTTGGTGCAGCCAACTAGAGGTGTTGTCACACCCGTCTTTAGGTTGTTTTGTGACACATTGTGGTTGGAATTCGACACTCGAGAGTATCGCGTGTGGGGTTCCGGTGGTGGCGTTTCCTAGATGGTCCGATCAAACAACAAATGCAAAGCTTATTGAAGATGTGTGGGGAATTGGGACGAGGGTGACCGCGAATGATCAAGACGGAGTTGTTGAAGCGGAGGAGATAAGAAGGTGTATAGAAATGGTGATGGGAGGTCATGAAAGAGGAGAAACAATGAGAATGAATGCTAAGAAGTGGAAGGATTTGGCTAGAGAGGCTATGAAAGAAAGTGGATCTTCGTATATCAATCTCAAGGATTTTGTTACACAAATTGGAAGTTCTACTTAA

MAQSDQPQPHFLVVTFPAQGHINPALQFAKQLARFDVKLTFTTTVSAYRGMTKFDQIYNNFNFVVYSDGFDDGFTSKSVDPDLFMTQFRTRGIQSLKETITSSAENGTPVTCLVYTFLLPWAAELARELNVLPALLWIQPASVFRVYYYYFNGYDKLIGEDCTESSWSIELPGLPSLKSCDLPSFCLPSSPYDTVLSLFKELLHTLSSFEKPKILANTFDALEEEALKEIDGKINMVGVGPLIPSAFLDGNEQSDRSFGGDMFDKSHDCLEWMNTNHEGSIVYVSFGSLIELSKKEKESIACGLLESKRPFLWVMRDKDGEAKEKEDEISCIEELKQLGLIVPWCSQLEVLSHPSLGCFVTHCGWNSTLESIACGVPVVAFPRWSDQTTNAKLIEDVWGIGTRVTANDQDGVVEAEEIRRCIEMVMGGHERGETMRMNAKKWKDLAREAMKESGSSYINLKDFVTQIGSST

SrUGT75F1

ATGTCAAGCAATCGGAAAATCCTGATAGTTGCTTATTCCGGGAAAGGCCACATCAATCCAGCACTCCGGTTCACCAACCGCCTCCTCAAAGCAGGCGTTGATGTCACCTTCTCCACGAGTCTCTCAGTCGTTCAACTCATCGATACAAAAACCGTCCCTCAAGGCCTAAAGTTTGCTCCATTTTCCGACGGTCACGATACCGGAAAACATCCAAACACACCCCTTCAACAATTTATCTCTGATTTCGAAACAAACGGTGCCCGTGCTGTCACAGAAATAATAGTTTCTGCGACAGACGCAGGTCAACCATTTGACTGTGTGATCTACACCACTGTAATACCGTGGGCAGCACGCGTAGCGCATGCCCACGGCGTTACACCAGTTCTCCTGTGGTGTCAGTCTGCCACCATCTTGGATATTTACTACTACTATCGTAACGATAACGAATATCGAACTTTGATATCCACCAACAACAACAACCCGACGTTTCCGATCAATTTGCCAGGACTGCCGCCACTAACCATAGCCGATTTGCCTTCGTTTTTATTATCTTCATGTCCTAAAGAACACAAGTTTCTTGTTCCGATTTTAGAAGATCATATCGATGTACTGAAAATTACTCCAAGAATACTTGTAAACTCTGTTGATGAACTTGAATTCGAATCCATACGAGCAATCGAGAACCTTAAGTTCATTCCAATTGGGCCTTTGATCCCCTCAGATTCCTATGATCCAAAAGACTCATCGAACTATTCTTTGGGAGTCGATTTCTTCGACAAAACTGATAATGATGAATATGATTACATCCAGTGGTTAAACACACAACCAAAATCATCAGTCGTGTATGTTTCATTCGGAACAATAGCTACATTCAAGATGGAGCAACTGGAGGAGATGGCGATTGGATTAATCGCGATTGGTAGACCGTTTTTATGGGTGATTAGAGATAGTGAGCAAGCAGAAAGAATGAGCGAAATCGATGGATTGCAGAAACAGGGGTTGATAGTGAAATGGTGTTCTCAAACGGAGGTATTCAGTCATCAGTCAGTCGGTTGCGTTGTGATGCACTGCGGGTGGAATTCTACGGTGGAGGTGCTGGCGGCCGGAATTCCCACGGTGGCGTTTGCGCAGTGGTCGGATCAGCCGGCGGACGCGAAGATGATTGAAGATGTGTGGAAGATAGGAATTAGGGTTAAAACAAGGGAAGCAGATGGTATGGTGGAAGGTGTGGAGATTAAGAGGTGTGTGGAAATGGTGATGGAGGATGAACTGATGAAGATTAATGCTGTGAAATGGAAGGAGGTGACCAGAAAAGCTCTCATCAATGGCGGATCTTCCGCCATTAATCTTCAAACTTTCTTGAATGACCTTGGAAACTAA

MSSNRKILIVAYSGKGHINPALRFTNRLLKAGVDVTFSTSLSVVQLIDTKTVPQGLKFAPFSDGHDTGKHPNTPLQQFISDFETNGARAVTEIIVSATDAGQPFDCVIYTTVIPWAARVAHAHGVTPVLLWCQSATILDIYYYYRNDNEYRTLISTNNNNPTFPINLPGLPPLTIADLPSFLLSSCPKEHKFLVPILEDHIDVLKITPRILVNSVDELEFESIRAIENLKFIPIGPLIPSDSYDPKDSSNYSLGVDFFDKTDNDEYDYIQWLNTQPKSSVVYVSFGTIATFKMEQLEEMAIGLIAIGRPFLWVIRDSEQAERMSEIDGLQKQGLIVKWCSQTEVFSHQSVGCVVMHCGWNSTVEVLAAGIPTVAFAQWSDQPADAKMIEDVWKIGIRVKTREADGMVEGVEIKRCVEMVMEDELMKINAVKWKEVTRKALINGGSSAINLQTFLNDLGN

SrUGT75F2

ATGACAACTCACCGGAAAAACAAAATCCTGATCGTCTCCTATCCTAACCAAGGCCACATTAACCCATCTCTCCGTTTCGCCAACCGACTTGCTAAATTCGGTGTCGATGTCACCTTCTCCACCAGCGTCTCCGTCATACGACGCATTGACATAGAAACCACCCACCACGGCCTCATCTTTGCCCCATTTTCCGATGGTCACGACGATGGTCAACAACCATCGACCACCTTGCAACAATTCTTCTCAGATTTTGCCACTAATGGTGCTTGTGCAATCGAGAAAATCATTACCACCGCTACGGCAGCAAGACAACCATTTGACCACTTGGTTTACACCCTTATATTACCATGGGCCGCAAAGGTGGCTAAAGCTCACAATCTCGAGTCAACTCTCCTATGGTGCCAGTCAGCTACTATCTTCGATATTTATTATTACTACTTTAACGGTTACAAAGATTTAATATCGTGTAACAACAACCACCACCCAGCGGTTCCGATCAACTTATCTGGACTTCCGTCATTAACCACCGCCGATTTGCCGTCTTTTCTTTTGTCATCTAGCCCAAAAGAACACGATTTTATTCTAGAGTTTATAAAAGACCATATTGATGTGCTCAAAACTACTCCAAGAATACTTGTTAACACTTTTGATGAACTTGAAATGGAACCCGTTAGAGCGGTTGAGAAACTGGTGATGCTTCCGGTGGGACCGTTAGTCCCACCAGAACTGTTAGACGGAAGTGGTAGTACGTCATATAATTCACGCGGATATGACTTGTTTGAAAAACCAGAGGAAGATTATATGAAATGGTTGAACACAAGGCCGAAATCGTCGGTTGTGTATGCTTCATTTGGAAGCATGGCGACTTTGTCGATGGATCAAGCGGAGGAGATGGGATCCGTGTTGGTTGAAAGTGATCGACCGTTTTTATGGGTGATTAGAGACGGTGATCAAGCGGTGAAACTGAGTAATATAGAGATGCTTAAAAAGCAAGGTATGATAGTGAGTTGGTGTTCTCAAGTGGAGGTGTTGAACCACCAATCAATTGGGTGTTTCTTGACGCATTGTGGGTGGAATTCGACGATGGAAGCATTGGCGGCCGGTGTTCCGACTGTTGTGTTTCCACAGTGGTCGGATCAGATGACCAACGGGAAGATGATTGAAGATGTGTGGAAAACAGGTGTTAGAGTGAAAAGGAGGGAGGAAGATGGAGTGGTGGAAGCGAAGGAGATTAAGAGGTGTTTGGAGATGGTGATGGAAGATGGAGAGATGAGGAGAAATGCTGAGAAATGGAGGGAGTTAGCAAGACAAGCTCATAACAATGGCGGATCATCCACCATGAACATCCAAGCTTTCTTGGATGATGATTGA

MTTHRKNKILIVSYPNQGHINPSLRFANRLAKFGVDVTFSTSVSVIRRIDIETTHHGLIFAPFSDGHDDGQQPSTTLQQFFSDFATNGACAIEKIITTATAARQPFDHLVYTLILPWAAKVAKAHNLESTLLWCQSATIFDIYYYYFNGYKDLISCNNNHHPAVPINLSGLPSLTTADLPSFLLSSSPKEHDFILEFIKDHIDVLKTTPRILVNTFDELEMEPVRAVEKLVMLPVGPLVPPELLDGSGSTSYNSRGYDLFEKPEEDYMKWLNTRPKSSVVYASFGSMATLSMDQAEEMGSVLVESDRPFLWVIRDGDQAVKLSNIEMLKKQGMIVSWCSQVEVLNHQSIGCFLTHCGWNSTMEALAAGVPTVVFPQWSDQMTNGKMIEDVWKTGVRVKRREEDGVVEAKEIKRCLEMVMEDGEMRRNAEKWRELARQAHNNGGSSTMNIQAFLDDD

SrUGT76G1-1

ATGGAAAATAAAACGGAGACCACCGTTCGCCGGCGCCGGAGAATAATATTATTCCCGGTACCATTTCAAGGCCACATTAACCCAATTCTTCAGCTAGCCAATGTGTTGTACTCTAAAGGATTCAGTATCACCATCTTTCACACCAACTTCAACAAACCCAAAACATCTAATTACCCTCACTTCACTTTCAGATTCATCCTCGACAACGACCCACAAGACGAACGCATTTCCAATCTACCGACTCATGGTCCGCTCGCTGGTATGCGGATTCCGATTATCAACGAACACGGAGCTGACGAATTACGACGCGAACTGGAACTGTTGATGTTAGCTTCTGAAGAAGATGAAGAGGTATCGTGTTTAATCACGGATGCTCTTTGGTACTTCGCGCAATCTGTTGCTGACAGTCTTAACCTCCGACGGCTTGTTTTGATGACAAGCAGCTTGTTTAATTTTCATGCACATGTTTCACTTCCTCAGTTTGATGAGCTTGGTTACCTCGATCCTGATGACAAAACCCGTTTGGAAGAACAAGCGAGTGGGTTTCCTATGCTAAAAGTGAAAGACATCAAGTCTGCGTATTCGAACTGGCAAATACTCAAAGAGATATTAGGGAAGATGATAAAACAAACAAAAGCATCTTCAGGAGTCATCTGGAACTCATTTAAGGAACTCGAAGAGTCTGAGCTCGAAACTGTTATCCGTGAGATCCCGGCTCCAAGTTTCTTGATACCACTCCCCAAGCATTTGACAGCCTCTTCCAGCAGCTTACTAGACCACGATCGAACCGTTTTTCAATGGTTAGACCAACAACCGCCAAGTTCGGTACTGTATGTTAGTTTTGGTAGTACTAGTGAAGTGGATGAGAAAGATTTCTTGGAAATAGCTCGTGGGTTGGTTGATAGCAAGCAGTCGTTTTTATGGGTGGTTCGACCTGGGTTTGTCAAGGGTTCGACGTGGGTCGAACCGTTGCCAGATGGGTTCTTGGGTGAAAGAGGACGTATTGTGAAATGGGTTCCACAGCAAGAAGTGCTAGCTCATGGAGCAATAGGCGCATTCTGGACTCATAGCGGATGGAACTCTACGTTGGAAAGCGTTTGTGAAGGTGTTCCTATGATTTTCTCGGATTTTGGGCTCGATCAACCGTTGAATGCTAGATACATGAGTGATGTTTTGAAGGTAGGGGTGTATTTGGAAAATGGGTGGGAAAGAGGAGAGATAGCAAATGCAATAAGAAGAGTTATGGTGGATGAAGAAGGAGAATACATTAGACAGAATGCAAGAGTTTTGAAACAAAAGGCAGATGTTTCTTTGATGAAGGGTGGTTCGTCTTACGAATCATTAGAGTCTCTAGTTTCTTACATTTCATCGTTGTAA

MENKTETTVRRRRRIILFPVPFQGHINPILQLANVLYSKGFSITIFHTNFNKPKTSNYPHFTFRFILDNDPQDERISNLPTHGPLAGMRIPIINEHGADELRRELELLMLASEEDEEVSCLITDALWYFAQSVADSLNLRRLVLMTSSLFNFHAHVSLPQFDELGYLDPDDKTRLEEQASGFPMLKVKDIKSAYSNWQILKEILGKMIKQTKASSGVIWNSFKELEESELETVIREIPAPSFLIPLPKHLTASSSSLLDHDRTVFQWLDQQPPSSVLYVSFGSTSEVDEKDFLEIARGLVDSKQSFLWVVRPGFVKGSTWVEPLPDGFLGERGRIVKWVPQQEVLAHGAIGAFWTHSGWNSTLESVCEGVPMIFSDFGLDQPLNARYMSDVLKVGVYLENGWERGEIANAIRRVMVDEEGEYIRQNARVLKQKADVSLMKGGSSYESLESLVSYISSL

SrUGT76G1-2

ATGGAAAATAAAACGGAGACCACCGTTCGCCGGCGCCGGAGAATAATATTATTCCCGGTACCATTTCAAGGTCACATAAACCCAATGCTTCAGCTAGCCAATGTGTTGTACTCCAAAGGATTCAGTATCACCATCTTTCACACCAACTTCAACAAACCCAAAACATCTAATTACCCTCACTTCACTTTCAGATTCATCCTCGACAACGACCCACAAGACGTACGCATTTCCAATCTACCGACTCATGGTCCGCTCGCTGTTATGCGGATTCTGATTATCAACGAACACGGAGCTGACGAATTACGACGCGAACTGGAACTGTTGATGTTAGCTTCTGAAGAAGATGGAGAGGTATCGTGTTTAATCGCCGATCAGATTTGGTACTTCACGCAATCTGTTGCTGACAGTCTTAACCTCCGACGGCTTGTTTTGGTGACAAGCAGCTTGTTTAATTTTCATGCACATGTTTCACTTCCTCAGTTTGATGAGCTTGGTTACCTCGATCCTGATGACAAAACCCGTTTGGAAGAACAAGCGAGTGGGTTTCCTATGCTGAAAGTGAAAGATATCAAGTGTAGTTTTTCGATGTGGAAAAAATACAAAGAGTATTTCGAGAACATTACGAAACAAACAAAAGCATCTTCAGGAGTCATCTGGAACTCATTTAAGGAACTCGAAGAGTCTGAGCTCGAAACTGTTATCCGTGAGATCCCGGCTCCAAGTTTCTTGATACCACTCCCCAAGCATTTGACAGCCTCTTCCAGCAGCTTACTAGACCACGATCGAACCGTTTTTCCATGGTTAGACCAACAACCGTCACGTTCGGTACTGTATGTTAGTTTTGGTAGTGGTACTGAAGTACTGGATGAGAAAGATTTCTTGGAAATAGCTCGTGGGTTGGTTGATAGCAAGCAGTCGTTTTTATGGGTGGTTCGACCTGGGTTTGTCAAGGGTTCGACGTGGGTCGAACCGTTGCCAGATGGGTTCTTGGGTGAAAGAGGACGTATTGTGAAATGGGTTCCGCAGCAAGAAGTGCTAGCTCATGGAGCAATAGGCGCATTCTGGACTCATAGCGGATGGAACTCTACGTTGGAAAGCGTTTGTGAAGGTGTTCCTATGATTTTCTCGGATTTTGGGCTCGATCAACCGTTGAATGCTAGATACATGAGTGATGTTTTGAAGGTAGGGGTGTATTTGGAAAATGGGTGGGAAAGAGGAGAGATAGCAAATGCAATAAGAAGAGTTATGGTGGATGAAGAAGGAGAATACATTAGACAGAATGCAAGAGTTTTGAAACAAAAGGCAGATGTTTCTTTGATGAAGGGTGGTTCATCTTACGAATCATTAGAGTCTCTAGTTTCTTACATTTCATCGTTGTAA

MENKTETTVRRRRRIILFPVPFQGHINPMLQLANVLYSKGFSITIFHTNFNKPKTSNYPHFTFRFILDNDPQDVRISNLPTHGPLAVMRILIINEHGADELRRELELLMLASEEDGEVSCLIADQIWYFTQSVADSLNLRRLVLVTSSLFNFHAHVSLPQFDELGYLDPDDKTRLEEQASGFPMLKVKDIKCSFSMWKKYKEYFENITKQTKASSGVIWNSFKELEESELETVIREIPAPSFLIPLPKHLTASSSSLLDHDRTVFPWLDQQPSRSVLYVSFGSGTEVLDEKDFLEIARGLVDSKQSFLWVVRPGFVKGSTWVEPLPDGFLGERGRIVKWVPQQEVLAHGAIGAFWTHSGWNSTLESVCEGVPMIFSDFGLDQPLNARYMSDVLKVGVYLENGWERGEIANAIRRVMVDEEGEYIRQNARVLKQKADVSLMKGGSSYESLESLVSYISSL

SrUGT76G2

ATGGAGAACCAACCACAAACAACCGTTCGTCGGCACCGGAGAATAATACTTTTTCCGATGCCATTTCAAGGTCATATAAACCCAATGATTCAGCTAGCCAATCTTCTCTATTCCAATGGATTCAGCATCGTCATCCTTCACACAAACTTCAACGCGCCCAAATTCTCTAATTACCCTAACTTCACTTTCATATCAGTTCTCGATAACGCCGAAAACGAACCGTTTTCCACATCATCGTCGTTCGAATTCGCTCAAAATTATACCTTTAAACAAGACGGTGCAGATGAATTACGTCATAAACTGGAATTATTGTTAGCTTCTGGAAACGATGAGCCGGTATCGTGTTTGATCACTGACGCGATTTGGCACTTCACGCAATCGGTGGCTGATAGTCTTCAGATTCCGAGAATTGTTTTGAGGACAACTAGCGTTTATTGTTCTATTGTTTATGCTTCCATTCCCCTTTTTGATGATCGTGGTTACTTCGCACTTGATGACAGTCATTTGGAAGAACAAGTAATGGAGTTTCCATTACTAAAAGTGAAAGACATAAAAAAGATTGGAATAAAGAGCATGAATGACCCATATGCGAAGATGATACGCGAAATGGTTAAACAAATAAAAGCATCATCGGGAATCATTTGGAACTCTTTCAAAGAACTCGAAGAAACCGAGCTCGATAGCATTCCACATGATTTCCCGATACCACGTTTCATTATACCATTCCACAAGTATTTTAACGCTTCATCGAGTAGTTTACTAGAACAAGATCGAACCATTTTTCCTTGGTTAGACCAACAAGCACCCAAGTCTGTACTCTATGTTAGTTTTGGTAGCCTTTGTCAAGTGGATGAGAAAGAGTTCTTGGAAATAGCTCACGGGTTGGTCTATAGCCAACAACCCTTTTTATGGGTGGTTCGACTAGGGTTCGTTAAAGATTCAACATGGCTCGAGTCATTGCCATTGCCAGATGGGTTCCCAGGTGAAAGGGGGCGAGTTGTTAAATGGGCTCCTCAACAAGAAGTTTTAGCTCATGAAGCAACGGGTGCGTTTTGGACTCATAGCGGATGGAACTCGACTTTGGAGAGTGTTTGTGAAGGTGTTGCAATGATTTGTTCGCCTTTTTGGGGCGATCAACCGATTGACGCGCGATACATGAGCGATGTCTCGAAGGTGGGGGTGTATTTAGAGAATGGGTTTCAAAGAGATGAGATATCAAGTGCCATAAGAAGAGTAATGGTGGATGAAGATGGAAAAGATATTAGAGAGAGG

MENQPQTTVRRHRRIILFPMPFQGHINPMIQLANLLYSNGFSIVILHTNFNAPKFSNYPNFTFISVLDNAENEPFSTSSSFEFAQNYTFKQDGADELRHKLELLLASGNDEPVSCLITDAIWHFTQSVADSLQIPRIVLRTTSVYCSIVYASIPLFDDRGYFALDDSHLEEQVMEFPLLKVKDIKKIGIKSMNDPYAKMIREMVKQIKASSGIIWNSFKELEETELDSIPHDFPIPRFIIPFHKYFNASSSSLLEQDRTIFPWLDQQAPKSVLYVSFGSLCQVDEKEFLEIAHGLVYSQQPFLWVVRLGFVKDSTWLESLPLPDGFPGERGRVVKWAPQQEVLAHEATGAFWTHSGWNSTLESVCEGVAMICSPFWGDQPIDARYMSDVSKVGVYLENGFQRDEISSAIRRVMVDEDGKDIRER

SrUGT76H1

ATGATTCCCACTCTCCAGCTAGCCACAACCCTTCATGCCAAAGGTTTCACCATAGCAATCGCACACTCCAAACTTAACCCACCCAACCCATCTAACCATCCTTCCGATTTCATCTTCCTCCCGCTTTCCGATGACATACCGGCCATTGACAACTCCGGCAGCTTCACTGACTTCACCAGAAAGCTCAACAACAACTGCAAACCATCATTCAAGGAACACTTGACTCGATTGATCAGTGAAGGAAACAAATCGATCGTTGTCGTGTATGATAATGTGTTGCACTTTGCCGGAATTGTTGCCGTTGATCTGAATCTCGCCGCCGTTATGCTCCGCAGTTCTAGTGCGGCGTACTTTCCGGCTTTTCTTGCCCGTCAACAGCTCCGTCAACGAGGCAGGTTTCTTGAAGAAGATTTTAAGATGGATGAGATGGTGCCAAATCATTATCCCATGAGGTACAAAGACCTACCATTTTCTAAATCACCAATTGAAGATTGGAGACAACTTTATTCAAACTTCAGTCAACAAGCACACCCTTCAGCCGTTATCTGGAACACCATCAAATTCCTCGAACACGAGTCGTTGACTCAAGTCCACAACTACTATCAGGTTCCTGTTTTCGCAGTTGGACCTTTGCACAAAATGACACCAACCTCGTATATTGGTTCTCACGAAGAAGACAACGGTTGCATCACATGGCTCGATAAACAACCTCCCAAATCCGTGGTCTACATAAGCTTTGGAAGCTTAGCCACAATGGAGGCAAAAATTCTAACCGAGATGGCATTTGGTCTAGCTAAAAGCAACCAACGGTTCCTATGGGCGGTTAGACCAGGTTTGATTAGTGATTCAGGGTGGGTTGAGTTTTTGCCAGAGGGGTTCGTAGAGGAAACGAGAGGACGAGGGTTAATCGTGAAATGGGCTCCTCAGAAGGAAGTGTTAGCCCATTTTGCGGTGGGTGGGTTTTGGAGTCATTGTGGATGGAATTCGTGTTTGGAGAGTATTTCATCAGGAGTTGTGATGATGTGTCAACCGTTTATTGCAGACCAAGGGGTGAATGCGCGTTATGTGAGTTACGTGTGGAAGATCGGGCTTGAGTTGGAGCGAGTTGAGAGAGGAGAGATTGAAAGGATGATTAAAAGAGTTATGGTGGATGATGAAGGAGAGGAAATGAGAGTTAGAGTGAATGATATGAAGAAAATGGTTAAAGAAGCTTTGGAGATTGGTGGTTCTTCACAAGAATCATTTGAAGGTTTGGTTGAATTCTTGTTGTCATGTTGA

MIPTLQLATTLHAKGFTIAIAHSKLNPPNPSNHPSDFIFLPLSDDIPAIDNSGSFTDFTRKLNNNCKPSFKEHLTRLISEGNKSIVVVYDNVLHFAGIVAVDLNLAAVMLRSSSAAYFPAFLARQQLRQRGRFLEEDFKMDEMVPNHYPMRYKDLPFSKSPIEDWRQLYSNFSQQAHPSAVIWNTIKFLEHESLTQVHNYYQVPVFAVGPLHKMTPTSYIGSHEEDNGCITWLDKQPPKSVVYISFGSLATMEAKILTEMAFGLAKSNQRFLWAVRPGLISDSGWVEFLPEGFVEETRGRGLIVKWAPQKEVLAHFAVGGFWSHCGWNSCLESISSGVVMMCQPFIADQGVNARYVSYVWKIGLELERVERGEIERMIKRVMVDDEGEEMRVRVNDMKKMVKEALEIGGSSQESFEGLVEFLLSC

SrUGT76H3

ATGGCGGAGCACGGCGGCCGGAGACGGCGATTGGTGATGGTAGCAAGTCCACTTCAAGGTCACATGACCCCCATGCTTCAGCTTGCAACTTACCTTCACTCCCAAGGGTTTTCAATAACCGTCGCTCACTCCGATCTAAACCCGCCGGAATCTTCCAACCACCCTGATTTAACCTTCCTCTCACTTTCCGGTAACTTATCCGGCACCGGCAGCTTCACCAGCTTGATCCAGTTATTTCAAACTCTTAACGATATCTGCAAACCCCATCTCATTGAATATCTGGCTCAGATTATAAAATCAGAAGAAGATATTATTGATAAGGAGTTGATCGTAATCATCCATGATAACCTCACGTTTTTCGCCGGAGAAGTTGCCGGCGAGCTGGGTTTGCCGGCGATCGTCTTGCGTGGAAGTAGTGCAGTGTTTTCCCCTGCGTTTAACATCATCCCTCTGCTTCATCAAGATAATCGATTTCCTCCACAAGATTCTTTATTGCAGGAAATAATTCCAGAACTTCATCCATTCAAATACAAAGATCTACCTTTCATTAACCGCCCGATAAATCAAACCCTTCATTTGATCAAAATGATTAACCTCAAGAACCCCCCATCCGCTTTAATTTGGAACACCATCGAGTTTCTTGAACAATCGGCGTTAACCCAGATCCGCGATACCCACCAAGTTCCAGTTTTTACAATCGGTCCGTTGCACAAAATAGTCACAACACGTTCGACTAGCTTTCTTGAAGAGGACACAAGTTGCATCAATTGGTTAGATAAACAATTACCCAAATCAGTGGTTTATGTGAGTTTAGGAAGTTTAGCAAACTTGGATGAAAAGGTTGCATCTGAAATGGCATGCGGTCTAGCCATGAGTAACCATAAGTTCATATGGGTGGTTCGACCCGGTTCGGTCAGTGGGTTTGCATGGATCGAGTTTTTGCCGGATAGTTTGGTGGGTGAAATGAAGGCGAGAGGTTTGATTGTGAAGTGGGCACCTCAGAAGTCGGTTTTGGCGCATAACGCGATTGGTGGATTTTGGAGTCATTGCGGTTGGAACTCGACTATGGAAGGTGTTTCTGAAGGGGTCCCAATGTTGTGTCAACCGTTTGATGTTGATCAGTTGTTGAATGCGAGGTATGTGAGTGACGTTTGGAAGACGGGTTTTGAAATTGTTGTTGAGAGAGGTGAGATTGCGCGTGCGATTAAAAGAGTTTTGGTGGATTACGAAGGCGAAGAAATGAGGCAAAGAGCTATGGAGAATCAAGAAAAGGTTAAACTTGCGATCAGTTATGGTGGTTCTTCGTATAACTCGTTAAAAGACTTGGTGTCCTTCATTTTGTCTTTGTAA

MAEHGGRRRRLVMVASPLQGHMTPMLQLATYLHSQGFSITVAHSDLNPPESSNHPDLTFLSLSGNLSGTGSFTSLIQLFQTLNDICKPHLIEYLAQIIKSEEDIIDKELIVIIHDNLTFFAGEVAGELGLPAIVLRGSSAVFSPAFNIIPLLHQDNRFPPQDSLLQEIIPELHPFKYKDLPFINRPINQTLHLIKMINLKNPPSALIWNTIEFLEQSALTQIRDTHQVPVFTIGPLHKIVTTRSTSFLEEDTSCINWLDKQLPKSVVYVSLGSLANLDEKVASEMACGLAMSNHKFIWVVRPGSVSGFAWIEFLPDSLVGEMKARGLIVKWAPQKSVLAHNAIGGFWSHCGWNSTMEGVSEGVPMLCQPFDVDQLLNARYVSDVWKTGFEIVVERGEIARAIKRVLVDYEGEEMRQRAMENQEKVKLAISYGGSSYNSLKDLVSFILSL

SrUGT76H4

ATGGCGGAGCACGGCGGCCGGAGACAGCGGCTGGTGTTGGTACCATCTCCACTTCAAGGTCACGTGACCCCCATGCTTCAGCTTGCAACTTACCTCCATTCTCAAGGGATTTCAATAACCATCGCTCAGTACCCCAACTTCAACTCGCCGGATTCTTCCAACCATCCAGAACTAACCTTCCTCCCACTATCCTCCGGCAACTTATCCGTCGCCGACATCTCCGGCGGCTTTTTCAAGTTCATCCAAACTCTTAACCATAACTGCAAACCCCATTTCCGGGAATACCTTGTTCAGAACATGAGTTCTGATGATAAGGAATCAATCGTTATCATCCGTGATAATCTCATGTTTTTCGCCGGAGAAATCGCCGGCGAGCTGGGTCTGCCTTCGATCATTTTACGTGGCAGCAATGCTGTCATGTTGACTGCTAGCGACATCATCCCTCAACTTCATCAAGAAGGTCGTTTTCCGCCACCAGATTCTTTGTTGCAGGAAACAATTCCAGAACTGGTTCCATTCAGATACAAAGATCTACCATTTATTGGCTATCCAATACATCAAACCCTTGAATTTAGTATCACCATGATGACCCCCAAATCACCTGCTTCCGCCATTCTTATCAACACCCTCGAATTTCTTGAACAATCGGCATTAACCCAGATCCGTGATCATTACAAAGTTCCAGTTTTTACAATCGGACCATTGCACAAAATAGTCACAACTCGTTCCACTAGCATTCTTGAAGAAGATACAAGTTGCATCAATTGGTTAGATAAACAATCACCCAAATCAGTGGTTTATGTGAGTTTAGGAAGCTTAGCAAAGTTGGATGAAAAGGTTGCATCTGAAATGGCATGTGGTTTAGCCATGAGTAACCATAAGTTCCTATGGGTGGTTCGACCCGGTATGGTTCATGGGTTTGAATGGGTCGAGTTTTTGCCGGATAGTTTGGTGGGTGAAATGAAGGCGAGAGGTTTGATTGTGAAATGGGCACCCCAGACGACGGTTTTGGCGCATAACGCGGTTGGTGGATTTTGGAGTCATTGCGGTTGGAACTCGACCATAGAATGCTTAGCTGAAGGGGTCCCGATGATGTGTCAACCGTTTTTTGCCGATCAGTTGTTGAATGCTAGGTATGTGAGTGATGTTTGGAAGACGGGTTTTGAGATTGTTATCGAGAAAGGTGAGATTGCGTGCGCGATTAAACGAGTTTTGGTGGATGAAGAAGGCGAAGAAATGAGGCAGAGAGCTATGGAGATTAAAGAAAAGGTTAAAATTGCAATCAACGATGGTGGTTCTTCTTATGACTCGTTCAAGGACTTGGTGGCGTTTATTTCATCACTCTAA

MAEHGGRRQRLVLVPSPLQGHVTPMLQLATYLHSQGISITIAQYPNFNSPDSSNHPELTFLPLSSGNLSVADISGGFFKFIQTLNHNCKPHFREYLVQNMSSDDKESIVIIRDNLMFFAGEIAGELGLPSIILRGSNAVMLTASDIIPQLHQEGRFPPPDSLLQETIPELVPFRYKDLPFIGYPIHQTLEFSITMMTPKSPASAILINTLEFLEQSALTQIRDHYKVPVFTIGPLHKIVTTRSTSILEEDTSCINWLDKQSPKSVVYVSLGSLAKLDEKVASEMACGLAMSNHKFLWVVRPGMVHGFEWVEFLPDSLVGEMKARGLIVKWAPQTTVLAHNAVGGFWSHCGWNSTIECLAEGVPMMCQPFFADQLLNARYVSDVWKTGFEIVIEKGEIACAIKRVLVDEEGEEMRQRAMEIKEKVKIAINDGGSSYDSFKDLVAFISSL

SrUGT76I1-1

ATGCTTCAATTAGCAAACATCCTTCACACTCACGGTTTTAAAATTACGATCATACACGCTAAATTCAACTCTCCAAATCCTTCAAAATATCCTCACTATAACTTCAAGTCCATTGACGAAGGTTTTTCGAAGATTGCAGATCAGTTGGGTACTAACAAAGACGCTAGCTATTTTCTTAGGTACCTAAATGAAAGTTGTGTAGATCCGTTTCGGGATTGTTTGGGTGGGTTGTTGGCACAATCCGGTGAAGAGAAGGTTGCTTGTTTAATCACGGATGCGGGTTTCTACTTCACACAGGCGGTGGCGGATAAACTGAAGATACCTAGGATTGTGCTACGGACAAGTAGTCTTGGTTGTGTCCTTGCTTACGATGTTATACCTTTTTACTCTAAAAAGGATT

GCTTTCACCTTACAAAAGAAGATCCAGATTATGAAGCATCGGTGCCGGAGTATCCACTTTTGAAATTTAAAGACATCGCAAAGATCGCACCCAACCCACAAGGCATGGGTGATTTTGTCTCCAACAAGATTACTCAGATGAGAGCATCATCAGGGATCATATGGAACACTTACAAAGAACTCGAAGAATCCGATCTAGAAACTATCACTCAAACGATTCCGGTTCCACACTTCACTTTAGGCCCATTCCACAAGTACTTCCCCGCAACTTCTAGCAGCTTGATCGAACAAGACCGCACCATTCTGTCATGGCTAGACAAACAAGCTCCCAAATCGGTCATATATGTTAGTTTTGGAAGTGTTGCATATATAACAGAGTCAGAGTTTCAAGAAGTGGCTCATGGGCTAGCTAACACTGGTTTACCATTTTTGTGGGTGGTTCGACCAGGGATAGTTGCTGGTTCAAAATGGCTCGAGTCATTGCCCGAAAATTTTCTAGAGAAAATGGGCGATAGGGGACGGATAGTGAAATGGTCTCCTCAACAAGAAGTGCTGGCTCATCCATCAACGGGGTGTTTTTGGACTCATAATGGATGGAATTCAACGTTGGAGAGTATATGTGAAGGAGTTCCCATGATTTGTTCCCCGTGTTTTGTTGACCAACCAATAAATGCGCGATATGTGAGTGATGTTTGGAAGATTGGTGTTTTTTTGGAGGATGGGTTCGAAAGAGTGGGAATCGAGAACGCGATTAAACGAGTGATGATGGATAAAGAAGGAGAAGAAATCCGTGAGAGAATAACTTCTCTTAAGGATAAGGTAAACCCTTCACTTGACAAAGATGGCTCTTCTTGTCACTCACTAAACAAGTTGGTGGAATATATTACATCATTATAA

MLQLANILHTHGFKITIIHAKFNSPNPSKYPHYNFKSIDEGFSKIADQLGTNKDASYFLRYLNESCVDPFRDCLGGLLAQSGEEKVACLITDAGFYFTQAVADKLKIPRIVLRTSSLGCVLAYDVIPFYSKKDCFHLTKEDPDYEASVPEYPLLKFKDIAKIAPNPQGMGDFVSNKITQMRASSGIIWNTYKELEESDLETITQTIPVPHFTLGPFHKYFPATSSSLIEQDRTILSWLDKQAPKSVIYVSFGSVAYITESEFQEVAHGLANTGLPFLWVVRPGIVAGSKWLESLPENFLEKMGDRGRIVKWSPQQEVLAHPSTGCFWTHNGWNSTLESICEGVPMICSPCFVDQPINARYVSDVWKIGVFLEDGFERVGIENAIKRVMMDKEGEEIRERITSLKDKVNPSLDKDGSSCHSLNKLVEYITSL

SrUGT76I1-2

ATGCTTCAATTAGCAAACATCCTTCACACTCACGGTTTTAAAATTACGATCATACACGCTAAATTCAACTCTCCAAATCCTTCAAAATATCCTCACTATAACTTCAAGTCCATTGACGAAGGTTTTTCGAATATTGCAGATCAGTTGGGTTCTAACAAAGACGCTAGCTATTTTCTTAGGTACCTAAATGAAAGTTGTGTAGATCCGTTTAGGGATTGTTTGGGTGGGTTGTTGGCACAATTCGGTGAAGAGAAGGTTGCTTGTTTAATCACGGATGCGGGTTTCTACTTCACACAGGCGGTGGCGGATGAACTGAAGATACCTAGGATTGTGCTACGGACAAGTAGTCTTGGTTGTGTCCTTGCTTACGATGTTATACCTTTTTACTCTAAAAAGGATTGCTTTCACCTTACAAAAGAAGATCCAGATTATGAAGCATCGGTGCCGGAATATCCACTTTTGAAATTTAAAGACATCGCAAAGATCGCACCCAACCCACAAGGCATGGGTGATTTTGTCTCCAACAAGATTACTCAGATGAGAGCATCATCAGGGATCATATGGAACACTTACAAAGAACTCGAAGAATCCGATCTAGAAACTATCACTCAAACGATTCCGGTTCCACACTTCACTTTAGGCCCATTCCACAAGTACTTCCCCGCAACTTCTAGCAGCTTGATCGAACAAGACCGCACCATTCTGTCATGGCTAGACAAACAAGCTCCCAAATCGGTCATATATGTTAGTTTTGGAAGTGTTGCATATATAACTGAGTCAGAGTTTCAAGAAGTGGCTCATGGGCTAGCTAATACTGGTTTACCATTTTTGTGGGTGGTTCGACCAGGGATAGTTGCTGGTTCAGAATGGCTAGAGTCATTGCCCGAAAATTTTCTAGAGAAAATGGGCGATAGGGGACGGATAGTGAAATGGTCTCCTCAACAAGAAGTGCTGGCTCATCCATCAACTGGGTGTTTTTGGACTCATAATGGATGGAATTCAACGTTGGAGAGTATATGTGAAGGAGTTCCCATGATTTGTTCCCCGTGTTTTGTTGACCAACCAATAAATGCGCGATATGTGAGTGATGTTTGGAAGATTGGTGTTTTTTTGGAGGATGGGTTCGAAAGAGTGGGAACCGAGAACACGATTAAACGAGTGATGATGGATAAAGAAGGAGAAGAAATCCGTGAGAGAATAACTTCTCTTAAGGATAAGGTAAACCCTTCACTTGACAAAGGTGGCTCTTCTTGTCACTCACTAAACAAGTTGGTGGAATATATTACATCATTATAA

MLQLANILHTHGFKITIIHAKFNSPNPSKYPHYNFKSIDEGFSNIADQLGSNKDASYFLRYLNESCVDPFRDCLGGLLAQFGEEKVACLITDAGFYFTQAVADELKIPRIVLRTSSLGCVLAYDVIPFYSKKDCFHLTKEDPDYEASVPEYPLLKFKDIAKIAPNPQGMGDFVSNKITQMRASSGIIWNTYKELEESDLETITQTIPVPHFTLGPFHKYFPATSSSLIEQDRTILSWLDKQAPKSVIYVSFGSVAYITESEFQEVAHGLANTGLPFLWVVRPGIVAGSEWLESLPENFLEKMGDRGRIVKWSPQQEVLAHPSTGCFWTHNGWNSTLESICEGVPMICSPCFVDQPINARYVSDVWKIGVFLEDGFERVGTENTIKRVMMDKEGEEIRERITSLKDKVNPSLDKGGSSCHSLNKLVEYITSL

SrUGT76I2

ATGGTAACCAATGGAAACGACACCAGTCTCCGGTCAAGAAACCACCGGAGAATAATATTATTTCCGTTACCTTTTCAGGGTCATATCAACCCCATGCTTCAATTAGCAAACATCCTTCACACACACCATTTTAAAATCACAATCATACACGCTAAATTCAACTCTCTGAATCCGTCAAAATATCCTCACTTCAACTTCAACCCCATTGACGAGGGTTTTTCGGAGATTGCGGACGAGTTGGGTACTAACAAAGACGCTAGCTATTTTCTTAGGTACCTAAATGAAAGTTGTGTAGATCCGTTTAGGGATTGTTTGGGTGGGTTGTTGGCACAACCCGGTGAAGAGAAGGTTGCTTGTTTAATCACGGATGCGGGTTTCTACTTCACACAGGCGGTGGCGGATGAACTGAAGATACCTAGGATTGTGCTACGGACAAGCAGTATTGGTTGTGTCCTTGCTTACGATGCTATACCTTTTTACTCTAAAAAGGATTGCTTTCTCCTTACAAAAGAAGATCCAGATTATGAAGCATCGGTGCCGGAATATCCACTTTTGAAATTTAAAGACATCGCAAAGATCGCACCCAACCCACAAGGCATGGGTGATTTTGTCTCCAACAAGATTACTCTGATGAGAGCATCATCAGGGATCATATGGAACACTTACAAAGAACTCGAAGAATCCGATCTAGAAACTATCACTCAAACGATTCCGGTTCCACACTTCACTTTAGGCCCATTCCACAAGTACTTCCCCGCAACTTCTAGCAGCTTGATCGAACAAGACCGCACCATTCTGTCATGGCTAGATAAACAAGCTCCCAAATCGGTCGTATACGTTAGTTTTGGAAGTGTGGCATATATAACCGAGTCAGAGTTTCAAGAAGTGGCTCATGGGCTAGCTAATACTGGTTTACCATTTTTGTGGGTGGTTCGACCAGGGATAGTTGCTGGTACAAAATGGCTCGAGTCATTACCTGAAAATTTTCTAGAAAAAATGGGCGATAGGGGACGGATAGTGAAATGGTCTCCTCAACAAGAAGTGCTGGCTCATCCTGCAACGGGGTGTTTTTGGACTCATAACGGATGGAATTCAACGTTGGAGAGTATATGTGAAGGAGTTCCCATGATTTGTTCCCCGTGTTTTGTTGACCAACCAATAAATGCGCGATATGTGAGTGATGTTTGGAAGATTGGTGTTTTTTTGGAGGATGGGTTCGAAAGAGTGGGAATCGAGAACGCGATTAAACGAGTGATGATGGATAAAGAAGGAGAAGAAATCCGTGAGAGAATAACTTCTCTTAAGGATAAGGTAAATCCCTCACTTGACAAAGGTGGCTCTTCTTGTCACTCACTAAACAAGTTGGTGGAATATATTTCATCATTATAA

MVTNGNDTSLRSRNHRRIILFPLPFQGHINPMLQLANILHTHHFKITIIHAKFNSLNPSKYPHFNFNPIDEGFSEIADELGTNKDASYFLRYLNESCVDPFRDCLGGLLAQPGEEKVACLITDAGFYFTQAVADELKIPRIVLRTSSIGCVLAYDAIPFYSKKDCFLLTKEDPDYEASVPEYPLLKFKDIAKIAPNPQGMGDFVSNKITLMRASSGIIWNTYKELEESDLETITQTIPVPHFTLGPFHKYFPATSSSLIEQDRTILSWLDKQAPKSVVYVSFGSVAYITESEFQEVAHGLANTGLPFLWVVRPGIVAGTKWLESLPENFLEKMGDRGRIVKWSPQQEVLAHPATGCFWTHNGWNSTLESICEGVPMICSPCFVDQPINARYVSDVWKIGVFLEDGFERVGIENAIKRVMMDKEGEEIRERITSLKDKVNPSLDKGGSSCHSLNKLVEYISSL

SrUGT79A1

ATGACTTTAACCTGCAAACCTAAGGAACTACACTTAGTGATGCTCCCTTTTTTTGCATTTGGTCACATTAGTCCTTTTGTTCAGTTAACCAACAAGTTATCATCGTATCTCGGTGTCAAGATTTCTTTCTTGGCTGCTTCCGCTAGTGTCAGCCGTATTGAAACCATGCTCAACTCTACCACCAACACCAAAGTCATCCCTCTAACTCTACCGCGTGTCGATGGTCTCCCTGAAGGCGTTGAAAACACCTCCGACACCTCACCAGCCACCATCGAGCTTCTCAAAGTCGCATTAGACCTCATGCGACCCCAAATTAAAACCGTACTAGCTAATCTTAAACCTAATTTTGTCTTCTTTGACTTTGCGCAATGGTGGTTGCCCGAAATGGCGTCTGAACTCGGCATAAAAACAGTATTTTTTTCTGTTTTTATGCCGAGCAACTTGGCATTTGTTGCCACGTGGTTCACCCACGATAAGGTCCCAACTATAGAAGTAATGAAGAAGCCTTCATCCGTGTTCCCAGATCTACAACTACCCGAAGCCATAACTTTAAAGACTTTTGAAGCGCTAGATTTCATGTATATCTTCAAGAGCTTTCATGGCACACTAAGTGTATTCGATCGGTTGATGAAATGCTTCAATGGATGCAATGCTGTACTCATCAAGTCATGTAGAGAAATGGAAGCACCGTACATCGACTACCTTAGCAACCAAGTTAAAAAGCCAGTTCTTCTAATCGGTCCGGTGGTTCCTGAGCCACACTCCGGCGAGTTAGATGAAACATGGGCCAACTGGTTGAGCCAATTTCCGGCTAAATCGGTTATATATTGTTCTTTCGGGAGTGAAACGTTTCTAACCGACGATCAAATTAGGGAATTAGCTTTAGGGTTGGAACTCACCGGACTTCCATTCTTCCTGGTGTTGAATTTTCCGGCGAATGTTGATAAATCTGCAGAACTGAAAAGAACACTACCTGATGGGTTTCTTAAAAGAGTGAAGGATAAAGGGATTGTACACTCAGGGTGGGTGCAACAACGACACATTTTGGCGCACGTTAGTGTGGGGTGTTATGTGTTTCATGCTGGTTTTAGCTCAGTGGTTGAAGGTCTGATTAATGATTGTCAGCTGGTGATGCTGCCATTAAAAGGAGATCAACTTACGAATTCTAAAGTGATTGCTTCAGATTGGAAAGCTGGAGTTGAAGTAAACAGGAGAGATGAAGATGGGTATTTTGGGAAAGATGATGTTTTTGAAGCTGTGAAGAGTGTTATGATGGAGACTGAAAAAGAACCAGCAAAATCTATAAGAGAAAATCAAAAGAAATGGAAAGAGTTTTTGCAGAACGATGAGATACATAGCAAGTATATTGCAGATTTTGTTGAGAACTTGAAGGCTCTTTCATAA

MTLTCKPKELHLVMLPFFAFGHISPFVQLTNKLSSYLGVKISFLAASASVSRIETMLNSTTNTKVIPLTLPRVDGLPEGVENTSDTSPATIELLKVALDLMRPQIKTVLANLKPNFVFFDFAQWWLPEMASELGIKTVFFSVFMPSNLAFVATWFTHDKVPTIEVMKKPSSVFPDLQLPEAITLKTFEALDFMYIFKSFHGTLSVFDRLMKCFNGCNAVLIKSCREMEAPYIDYLSNQVKKPVLLIGPVVPEPHSGELDETWANWLSQFPAKSVIYCSFGSETFLTDDQIRELALGLELTGLPFFLVLNFPANVDKSAELKRTLPDGFLKRVKDKGIVHSGWVQQRHILAHVSVGCYVFHAGFSSVVEGLINDCQLVMLPLKGDQLTNSKVIASDWKAGVEVNRRDEDGYFGKDDVFEAVKSVMMETEKEPAKSIRENQKKWKEFLQNDEIHSKYIADFVENLKALS

SrUGT83B1

ATGGCAAAAACTCATGTTCTAGCGATACCTTATCCGGCACAAGGCCATGTAATTCCTTTAATGGAGCTTGCTCAAGAGTTAGTCAAACAAGGAGTTAGGGTTACATTTATCAACACAGAGTTCAATCACAAGCTTATTGTGACAAGCAATTGTTTAGATAAATATGGTTACGGGGATATGATGCAAATGGTCTCAATCCCCGATGGTTTAGAATCATGGGAGGACAGAAGTGACCTTTGTAAGTTGACGATGTCAATATTAAAAACCATGCCCCAAAAGCTTGAAGAATTAATAGAGTCGATTAACAAAGAAGACAAAACTAAAGTTTGTTGTGTTATCGCCGATGGTTGCATGGGATGGGCCATACGAGTTGCGAAGAATATGGGAATTAGACGAGCCGCCTTCTGGCCTGCCTCAGTTGCAACATTGGCCTCAATGTTGTCTTTTCAAAAACTGATTGATGATGGGATTATAAACAACAAAGGCAAACCTCAAAGTCATGAGATAATCCGACTGTCAGAAACCATGCCACCTATAAAACCTAAGAACCTTGCTTGGGCATGCTTTGAGGACTCAGCTACAGTAGAAGCTGTTTTTCAAGCTGCAATACAGTGTGAAGAAGCTTCTAGACTAACTGAATGGTTTATATGTAACTCGTGCCCAGAGCTCGAGGTTGCAGCATTTAGCCTGTACCCACAGTTGTTGCCCATAGGACCTCTCCTGGCAACAAACCGGCTAGGTGACCAGACTGGCCACTTTTGGGAAGAAGACACCACCTGCTTAGAATGGCTTGATCAACAAGCAACATGTTCAGTCTTTTATATTGCGTTTGGGAGCTTTACTATTTTCAACCAAACTCAGCTTGAAGAACTGGCACTTGGAATTGAACTTTGTAACATACCATTCTTGTGGGTGGTGCGGCCAGGTATGACCAAGGAGACTAAGACTAATTTCCCAATTGGATTTATGGACAGAGTGGGGTCTCGTGGAAGAATCGTGAGTTGGGCACCACAACAGAAGGTCTTGGCTCATCCTTCTATAGCATGTTTCATGAGTCATTGTGGTTGGAACTCTACCATAGAAGGTGTCACAAATGGACTCCCTTTTTTGTGTTGGCCATACTTTGGTGATCAATTTCATAACGAGACTTATGTTTGTGACTTTTGGAAGACTGGTTTGGGTCTAGAAAAGGATGAGGCAGGTATCATCACTCGTGGAGAAATTAAAAGTAAGGTGGAGCGGCTGCTTAGTGACAAAACTTTCAAAGTTAACGCGTTTGACATTAAAGAAAAGGTTAAAAGTTGCATCAGTTTAAGGGGCTGCTCACGCAAAAATCTTACTATTTTTATTGAATGGATAAAAAAGAATGATGTAGATGCCAAGGATTAA

MAKTHVLAIPYPAQGHVIPLMELAQELVKQGVRVTFINTEFNHKLIVTSNCLDKYGYGDMMQMVSIPDGLESWEDRSDLCKLTMSILKTMPQKLEELIESINKEDKTKVCCVIADGCMGWAIRVAKNMGIRRAAFWPASVATLASMLSFQKLIDDGIINNKGKPQSHEIIRLSETMPPIKPKNLAWACFEDSATVEAVFQAAIQCEEASRLTEWFICNSCPELEVAAFSLYPQLLPIGPLLATNRLGDQTGHFWEEDTTCLEWLDQQATCSVFYIAFGSFTIFNQTQLEELALGIELCNIPFLWVVRPGMTKETKTNFPIGFMDRVGSRGRIVSWAPQQKVLAHPSIACFMSHCGWNSTIEGVTNGLPFLCWPYFGDQFHNETYVCDFWKTGLGLEKDEAGIITRGEIKSKVERLLSDKTFKVNAFDIKEKVKSCISLRGCSRKNLTIFIEWIKKNDVDAKD

SrUGT83B2

ATGGTAAAACCTCACGTCCTACTCCTACCCGGTCCGGCACAAGGCCATGTAAATCCTACAATCGAACTCGGTCTACGGTTAGTCGCACAAGGTGTAAAAGTCACACTCATCAACTCGGAAACAAGTCACAAGTTCATAACGAGCAATTGGGTGGCGCCCGATGGATGTGAGGATCTAATGCAGATGGTTTCCATCCCTGACGGGTTAGAACCAGGGGAAGATAGAAATGAATTTGGTAAGTTAATGGCGT

CGATGGAAAGATGTATGCCTAGCAAGCTCGAACAAGTTATAAAGACGATTAACGAAAAAGACGACAACAAAATCACGTGTCTTATTGTTGATTTTTGGATTGTGTGGGCTCTACGAGTCGCGAAGAAGATGGGAATTGGAAGAGTGAGCTTCTTTCCTGCATCGACAGCTACACTAGCCACCATGTTGAGCCTTCAGAAGTTGGTTGATGGTGGATTCATAAGCGACAATGGCATACCGCAAAATAGCGAGATGATTCGGTTGACAGAAGCCATGCCACCTATAAAACCCGAAAACCTCTTGTGGGCGAGTTATCGTGACTCAACTAGCATAGAGGATCATTTTCGGTATCATATAAGAATTAATGAATTAGCAACATTAATCGAATGGTTTATATGTAACTCAGCTGTTGAGCTAGAACCTGTAGCATTTAGCCTGTTCCCGAAGCTGTTGCCGGTAGGCCCTCTTTTGGCAAGCAACCGACTTGGTGATCAAGTAGGTCATTTTTGGCCCGCAGACTCCACTTGCTTATCATGGCTCGATCAACAACCTATATGTTCAGTCATTTACATAGCATTTGGGAGTATCACTATTATTAACCAAACTCAGTTCGAAGAACTAGCACTTGGTCTTGAACTTACCAACCGACCGTTCTTGTGGGTTGTGCGGCCTGGTATGACCAAGGAGACAACAGTTGCTTACCCAGATGGTTATATCAACCGAGTGGGGTCTCGTGGAAAAATTGTGACATGGGCACCTCAACAGAAGGTCTTGGCTCATCCTTCTGTAGCTTGTTTCATGAGTCACTGTGGTTGGAACTCAACTTTAGAAGGCGTGGCAAATGGACTCCCTATTTTGTGTTGGCCATACTTTGGTGATCAGGTTTACAACGAGACATACATTTGTGACGTATGGAAAACTGGGTTGGGGTTGAAGGTAGATGAAGAAGTGAGTATTGTTACACGAGAAGAAATAAAATGTAAGGTAGAAAAATTGCTTGGTGACACAACATTCAAATCTAAGGCCTTGGATCTTAAAGAAAAGGTCGCAAGTGGTGTCTCAAAAGGTGGATGTTCACACCGTAACCTTACCAAATTTATCGAATGGATACATAAAAAAGATGCGGATACCAATGATCATCATGAATCTAAGTGA

MVKPHVLLLPGPAQGHVNPTIELGLRLVAQGVKVTLINSETSHKFITSNWVAPDGCEDLMQMVSIPDGLEPGEDRNEFGKLMASMERCMPSKLEQVIKTINEKDDNKITCLIVDFWIVWALRVAKKMGIGRVSFFPASTATLATMLSLQKLVDGGFISDNGIPQNSEMIRLTEAMPPIKPENLLWASYRDSTSIEDHFRYHIRINELATLIEWFICNSAVELEPVAFSLFPKLLPVGPLLASNRLGDQVGHFWPADSTCLSWLDQQPICSVIYIAFGSITIINQTQFEELALGLELTNRPFLWVVRPGMTKETTVAYPDGYINRVGSRGKIVTWAPQQKVLAHPSVACFMSHCGWNSTLEGVANGLPILCWPYFGDQVYNETYICDVWKTGLGLKVDEEVSIVTREEIKCKVEKLLGDTTFKSKALDLKEKVASGVSKGGCSHRNLTKFIEWIHKKDADTNDHHESK

SrUGT84C1

ATGGGTTCAACTTCCGAACCAAATCAACAACCAAACGTGTTGCTCGTTACAGTTGCAGCTCAAGGTCATGTTAACCCAATGATAAGGTTCGGTAACTTACTTGTCTCCAAAGGTCTTCATGTCACTCTCGCCACTCACGATTATGCTCTCAACCACTGTTCGTCCATCGTCGGCGGCATCCACCTCGAGTTCTTCTCCGATGGTCTACCTCATGACTATAACCGACAAACCGGCGATTTCAATTACTACATGAACTCTCTACGTAAACACGGACCCATAAATCTATCTGCTTTAATCCGATCACACCGTCGGAAGTTTTCATGCATTATCAACACCCCTTTCGTGCCGTGGGCTGCTGACGTGGCGGCGGAGTTCCAGCTCCCGTGTGCGATGGTTTGGATCCAACCGTGCGCTATCTATCAGATATATTATTGTTATTACAATCGTTTGAACGAGTTTCCTACTGAAAGTAACCCTAATCTGAACGTGAAGTTACCCGGGCTGCCGGAGTTTTGCCCCGAAGAGTTACCGTCGTTTGTTCTTCCGTCGAATACGTTTCCTACGTTTGATAGCATACTGAAGGAAGTGTTTCATAACATGCACAAGATCAAATGGGTGTTGGGAAATTCGTTTATGGAACTCGAAAAAGAAGTGATAACGTCGATGAATGACGCCGGTCGGCCGTTTTGGCCGGTGGGACCGCTTGTTCCGGTGAGTCTTTTCGGAAAGATAGATCAAATCGGCGGCGATCTGAGTGGTTTCGATAAGTTTAAATCTGACAATGAAACTAACTGTTTTGAATGGTTAGATAAACAGCAACCGGCATCGGTTGTTTACATATCATTCGGAAGTCTTATATTTTCATCGGAGAAACAGATCGAAAGCATAGCAAGTGGTTTAAAAAGCTCAAAACGGCCGTTTCTGTGGGTGATAAAGTCGCCGGAAAATCAAGAAACAAAACAAGATTCGATCTTGGAAGAGATTAAGGAACAGGGTTTAATCGTGAAATGGAGTCCTCAAACGGCTGTATTATCACACCCATCGGTTGGG

TGTTTCTTGAGCCACTGTGGGTGGAACTCGTTGATTGAAAGTGTTACCGCCGGTGTGCCGGTGATTGCTTGCCCGCAGTGGACTGATCAGCCGACTAATGCTAAACTTGTGACTGATGTTTGGAATGTTGGGGTGAAATTGAATAAGAATTCGGAAGGGTTTTTTGTTGGGGAAGAAGTAGCGAGATGTGTGGATGAGATTATGAGCGGGTTGAGATCGGAGGAGTTTAGGAAGAATGCGGCGGAGTTAAAACGGGCGGCACGTGAGGCGGTGGCAGATGGTGGCTCGTCGGACGAGAATATAAATTTGTTTGTAAAAGAATTATTGTCTTCTTGCTCATGA

MGSTSEPNQQPNVLLVTVAAQGHVNPMIRFGNLLVSKGLHVTLATHDYALNHCSSIVGGIHLEFFSDGLPHDYNRQTGDFNYYMNSLRKHGPINLSALIRSHRRKFSCIINTPFVPWAADVAAEFQLPCAMVWIQPCAIYQIYYCYYNRLNEFPTESNPNLNVKLPGLPEFCPEELPSFVLPSNTFPTFDSILKEVFHNMHKIKWVLGNSFMELEKEVITSMNDAGRPFWPVGPLVPVSLFGKIDQIGGDLSGFDKFKSDNETNCFEWLDKQQPASVVYISFGSLIFSSEKQIESIASGLKSSKRPFLWVIKSPENQETKQDSILEEIKEQGLIVKWSPQTAVLSHPSVGCFLSHCGWNSLIESVTAGVPVIACPQWTDQPTNAKLVTDVWNVGVKLNKNSEGFFVGEEVARCVDEIMSGLRSEEFRKNAAELKRAAREAVADGGSSDENINLFVKELLSSCS

SrUGT84C2

ATGAGTTCTTTCGGTGACCAAACCAATCAACATACAAACATTCTTCTAGTTACTCTTTCTGCTCAAGGTCACATAAACCCCATCCTAAGATTCGGCAAAAATCTTGTCACTAAAGGGCTCAACGTCACTCTAGCCACCACCCATTCCGCCCTAAACACCATCTCCTCCGTACCAACCACCATCGGTGGGGTCCACCTTGAGTTCTTCTCCGATGGTCTACCAATCGACTACAACCGAAAGGCTGATATCGATTACTACATGGATACACTAACCGAATTAGGACCCGATAACCTCTTGAAATTAATCCGATCACATCAACGTAAATTTGCATGTATCATCCATACACCATTCGTGCCGTGGGTTGCTGACGTGGCAGCCGAGGTTGGTGTTCCGAACGCCATGCTTTGGATCCAACCATGCGCTTTGTACCAAATATATCATCGTTATTACAATCGGCTCGATGAGTTCCCGACTGGAAGTGACCCGAATATGAGCGTTAAGCTGCCCGGACTGCCCGTTTTTGGTTTGGATGAGTTACCATCGTTTGTTCTTCCGTCTAATACCCTTCGTTGCTTTGATACAATACTAAACAAAGCGTTTACTAACATGCATAAAGTGAAGTGGGTTCTCGGTAATTCGTTCATGGAGCTCGAGAAAGATGTAATTCTGTCGCTGAATAATTCCGGCCATGTGTTTTTGCCCGTCGGACCAACAGTTCCGGCGACGATTTTGGGTCAGGAGAAAGATGCGGATTTTGATCTATTTGAATCTAACGATGACAGTAATTGTATGGATTGGTTAAACAAACAAAAACCCTCATCGGTTGTTTATATTTCATTCGGGACTCTAATCTCTTCATCGGGGAAAGAAATCGAGAACATTGCAGCTGGTCTGAAACGCACAAAACGACCGTTTTTATGGGTGATAAGGTCACCGGAAAATCAAGAACCGACGGAGTTAGAGTTCTTGGAGGAGATCAAGGATCAAGGGTTGATTGTGAGCTGGAGCCCGCAAACCGTGGTTTTGTCACACCCGTCGGTTGGGTGTTTCGTGAGTCACTGCGGGTGGAACTCGTTGCTGGAAAGTATCGCCGCCGGTGTACCGGTGGTTGCTTGTCCGAAGTGGACCGATCAGCCGACAAACGCGAAACTCGTGACGGATGTTTGGGGGGTGGGTGTGAAGGTGAAGAAGAATTCTGAAGGTGGTGTTTGTGGGGAGGATGTGGGAAGATCCGTGGAAGAAGTTATGAGTGGTCCGAGATCGGAAGAGTTTAGGAAGAATGCGTTGGAGTTGAAGGCGGCGGCGCGTGAGGCGTTGACGGATGGTGGTTCATCGGACAAGAATATTCAGATATTTGTAAATGAAATCATCTTCTTGTAA

MSSFGDQTNQHTNILLVTLSAQGHINPILRFGKNLVTKGLNVTLATTHSALNTISSVPTTIGGVHLEFFSDGLPIDYNRKADIDYYMDTLTELGPDNLLKLIRSHQRKFACIIHTPFVPWVADVAAEVGVPNAMLWIQPCALYQIYHRYYNRLDEFPTGSDPNMSVKLPGLPVFGLDELPSFVLPSNTLRCFDTILNKAFTNMHKVKWVLGNSFMELEKDVILSLNNSGHVFLPVGPTVPATILGQEKDADFDLFESNDDSNCMDWLNKQKPSSVVYISFGTLISSSGKEIENIAAGLKRTKRPFLWVIRSPENQEPTELEFLEEIKDQGLIVSWSPQTVVLSHPSVGCFVSHCGWNSLLESIAAGVPVVACPKWTDQPTNAKLVTDVWGVGVKVKKNSEGGVCGEDVGRSVEEVMSGPRSEEFRKNALELKAAAREALTDGGSSDKNIQIFVNEIIFL

SrUGT84C3

ATGAGTTCTTTCGGTGACCAAACCAATCTACATACAAACATTCTTCTAGTTACTCTTTCTGCTCAAGGTCACATAAACCCCATCCTAAGATTCGGCAAAAATCTTGTCACTAAAGGGCTCAACGTCACTCTAGCCACCACCCATTCCGCCCTAAACACCATCTCCTCCGTACCAACCACCATCGGTGGGGTCCACCTTGAGTTCTTCTCCGATGGTCTACCAATCGACTACAACCGAAAGGCTGATATCGATTACTACATGGATACACTAACCGAATTAGGACCCGATAACCTCTTGAAATTAATCCAATCACATCAACGTAAATTTGCATGTATCATCCATACACCATTCGTGCCGTGGGTTGCTGACGTGGCAGCCGAGGTTGGTGTTCCAAACGCCATGCTTTGGATCCAACCATGCGCTTTGTACCAAATATATCATCGTTATTACAATCGGCTCGATGAGTTCCCGACTGGAAGTGACCCGAATATGAGCGTTAAGCTGCCCGGACTGCCCGTTTTTGGTTTGGATGAGTTACCATCGTTTGTTCTTCCGTCTAATACCCTTCGTTGCTTTGATACAATACTAAACAAAGCGTTTACTAACATGCATAAAGTGAAGTGGGTTCTCGGTAATTCGTTCATGGAGCTCGAGAAAGATGTAATTCTGTCGCTGAATAATTCCGGCCATGTGTTTTTGCCGGTCGGACCAACAGTTCCGGCGACGATTTTGGGTCAGGAGAAAGATGCGGATTTTGATCTATTTGAATCTAACGATGACAGTAATTGTATGGATTGGTT

AAACAAACAAAAACCCTCATCGGTTGTTTATATTTCATTCGGGACTCTAATCTCTTCATCGGGGAAAGAAATCGAGAACATTGCAGCTGGTCTGAAACGCACAAAACGACCGTTTTTATGGGTGATAAGGTCACCGGAAAATCAAGAACCGACGGAGTTAGAGTTCTTGGAGGAGATCAAGGATCAAGGGTTGATTGTGAGCTGGAGCCCGCAAACCGTGGTTTTGTCGCACCCGTCGGTTGGGTGTTTCGTGAGTCACTGCGGGTGGAACTCGTTGCTGGAAAGTATCGCCGCCGGTGTACCGGTGGTTGCTTGTCCGAAGTGGACCGATCAGCCGACAAACGCGAAGCTCGTGACGGATGTTTGGGGGGTGGGTGTGAAGGTGAAGAAGAATTCTGAAGGTGGTGTTTGTGGGGAGGATGTGGGAAGATCCGTGGAAGAAGTTATGAGTGGTCCGAGATCGGAAGAGTTTAGGAAGAATGCGTTGGAGTTGAAGGCGGCGGCGCGTGAGGCGTTGACGGATGGTGGTTCATCGGACAAGAATATTCAGATATTTGTAAATGAAATCATCTCTTCTTGTTAG

MSSFGDQTNLHTNILLVTLSAQGHINPILRFGKNLVTKGLNVTLATTHSALNTISSVPTTIGGVHLEFFSDGLPIDYNRKADIDYYMDTLTELGPDNLLKLIQSHQRKFACIIHTPFVPWVADVAAEVGVPNAMLWIQPCALYQIYHRYYNRLDEFPTGSDPNMSVKLPGLPVFGLDELPSFVLPSNTLRCFDTILNKAFTNMHKVKWVLGNSFMELEKDVILSLNNSGHVFLPVGPTVPATILGQEKDADFDLFESNDDSNCMDWLNKQKPSSVVYISFGTLISSSGKEIENIAAGLKRTKRPFLWVIRSPENQEPTELEFLEEIKDQGLIVSWSPQTVVLSHPSVGCFVSHCGWNSLLESIAAGVPVVACPKWTDQPTNAKLVTDVWGVGVKVKKNSEGGVCGEDVGRSVEEVMSGPRSEEFRKNALELKAAAREALTDGGSSDKNIQIFVNEIISSC

SrUGT84C4

ATGAGTTCTTTCGGTGACCAAACTAATCAACATACAAACATTCTTCTAGTTACTCTTTCTGCTCAAGGTCACATAAACCCCATCCTAAGATTCGGCAAAAATCTTGTCACTAAAGGGCTCAACGTCACTCTAGCCACCACCCACTCCGCCCTAAACACCATCTCCTCTGTACCAACCACCATCGGTGGGGTCCACCTTGAGTTCTTCTCCGATGGTCTACCAATCGACTACAACCGAAAGGCTGATATCGATTACTACATGGATACACTAACCGAATTAGGACCCGATAACCTCTTGAATTTAATCCGATCACATCAACGTAAATTTGCATGTATCATCCATACACCATTCGTGCCGTGGGTTGCTGACGTGGCAGCCGAGGTTGGTGTTCCGAACGCCATGCTTTGGATCCAACCATGCGCTTTGTACCAAATATATCATCGTTATTACAATCGGCTCGATGAGTTCCCGACTGGAAGTGACCCGAATATGAGCGTTAAGCTGCCCGGACTGCCCGTTTTTGGTTTGGATGAGTTACCATCGTTTGTTCTTCCGTCTAATACCCTTCGTTGCTTTGATACAATACTAAACAAAGCGTTT

ACTAACATGCATAAAGTGAAGTGGGTTCTCGGTAATTCATTCATGGAGCTCGAGAAAGATGTAATTCTGTCGCTGAATAATTCCGGCCATGTGTTTTTGCCGGTCGGACCAACAGTTCCGGCGACGATTTTGGGTCAGGAGAAAGATGCGGATTTTGATCTATTTGAATCTAACGATGACAGTAATTGTATGGATTGGTTAAACAAACAAAAACCCTCATCGGTTGTTTATATTTCATTCGGGACTCTAATCTCTTCATCGGGGAAAGAAATCGAGAACATTGCAGCTGGTCTGAAACGCACAAAACGACCGTTTTTATGGGTGATAAGGTCACCGGAAAATCAAGAACCGACGGAGTTAGAGTTCTTGGAGGAGATCAAGGATCAAGGGTTGATTGTGAGCTGGAGCCCGCAAACCGTGGTTTTGTCGCACCCGTCGGTTGGGTGTTTCGTGAGTCACTGCGGGTGGAACTCGTTGCTGGAAAGTATCGCCGCCGGTGTACCGGTGGTTGCTTGTCCGAAGTGGACCGATCAGCCGACAAACGCGAAGCTCGTGACGGATGTTTGGGGGGTGGGTGTGAAGGTGAAGAAGAATTCTGAAGGTGGTGTTTGTGGGGAGGATGTGGGAAGATCCGTGGAAGAAGTTATGAGTGGTCCGAGATCGGATGAGTTCAGGAAGAATGCGTTGGTGTTGAAGACGGCGGCGCGTGAGGCGTTGACGGATGGTGGTTCATCGGACAAGAATATTCAGATATTTGTAAATGAAATCATCTCTTCTTGTTTGTTACATTCGGAACAAAAGTAA

MSSFGDQTNQHTNILLVTLSAQGHINPILRFGKNLVTKGLNVTLATTHSALNTISSVPTTIGGVHLEFFSDGLPIDYNRKADIDYYMDTLTELGPDNLLNLIRSHQRKFACIIHTPFVPWVADVAAEVGVPNAMLWIQPCALYQIYHRYYNRLDEFPTGSDPNMSVKLPGLPVFGLDELPSFVLPSNTLRCFDTILNKAFTNMHKVKWVLGNSFMELEKDVILSLNNSGHVFLPVGPTVPATILGQEKDADFDLFESNDDSNCMDWLNKQKPSSVVYISFGTLISSSGKEIENIAAGLKRTKRPFLWVIRSPENQEPTELEFLEEIKDQGLIVSWSPQTVVLSHPSVGCFVSHCGWNSLLESIAAGVPVVACPKWTDQPTNAKLVTDVWGVGVKVKKNSEGGVCGEDVGRSVEEVMSGPRSDEFRKNALVLKTAAREALTDGGSSDKNIQIFVNEIISSCLLHSEQK

SrUGT85A8-1

ATGGCTTCAATAGCAGAAATGCAAAAGCCACATGCCATCTGCATCCCCTACCCAGCCCAAGGCCACATCAACCCCATGATGCAATTTGCTAAGCTCCTTCACTTCAAAGGCTTTCACATCTCTTTTGTCAATAACCACTACAACCATAAGCGGTTGCAGCGGTCCCGCGGTCTGTCCGCCCTCGAAGGTCTACCTGATTTTCATTTCTACTCGATTCCCGATGGCCTTCCACCTTCAAATGCTGAGGCCACCCAGTCGATCCCCGGGCTATGTGAGTCGATTCCTAAGCACAGTTTGGAACCATTTTGTGAATTGATCGCTACGCTAAATGGTTCGGACGTGCCACCTGTAAGCTGTATAATCTCTGATGGGGTCATGAGCTTTACGCTTCAAGCTGCCGAGAGGTTCGGGTTGCCGGAAGTTTTGTTCTGGACCCCAAGTGCTTGTGGGTTTTTGGCTTACACTCACTATCGAGATCTTGTGGATAAGGAGTATATTCCCCTCAAAGACACGAACGACTTGACAAATGGGTATTTAGAAACAAGCTTGGATTGGATTCCTGGGATGAAAAACATCCGATTAAAAGATTTCCCATCCTTTATTCGAACCACAGACATAAATGATATTATGCTCAATTATTTCTTGATTGAAACCGAAGCGATCCCAAAAGGCGTAGCGATCATTCTTAACACATTTGACGCGTTAGAAAAAGATAGTATTACGCCTGTACTTGCTCTAAATCCACAAATATACACCATTGGTCCATTACACATGATGCAACAATATGTCGATCATGATGAGAGACTCAAACACATTGGGTCCAACCTTTGGAAGGAAGATGTGAGCTGCATCAATTGGCTTGACACCAAAAAGCCTAATTCGGTTGTTTATGTGAACTTTGGAAGTATTACGGTTATGACGAAAGAACAACTGATCGAGTTTGGGTGGGGACTGGCTAATAGCAAGAAGGATTTCTTGTGGATAACGAGGCCTGATATTGTTGGAGGCAATGAAGCCATGATACCACCAGAGTTCATAGAGGAGACCAAAGAAAGGGGCATGGTTACTAGCTGGTGCTCTCAGGAAGAGGTTTTAAAACATCCATCAATCGGGGTATTCTTGACTCATAGTGGATGGAACTCGACCATTGAGAGTATTAGCAACGGTGTTCCCATGATTTGTTGGCCTTTTTTTGCAGAGCAACAAACAAATTGTCGGTATTGTTGTGTTGAATGGGAAATTGGATTGGAAATTGATACAGATGTGAAGAGAGAGGAGGTAGAGGCTCAAGTGAGGGAGATGATGGATGGGTCGAAAGGGAAGATGATGAAAAACAAGGCTTTGGAATGGAAGAAGAAGGCTGAAGAAGCGGTATCCATTGGTGGATCTTCTTATCTCAACTTTGAAAAATTAGTTACTGATATTCTTTTAAGAAAGTGA

MASIAEMQKPHAICIPYPAQGHINPMMQFAKLLHFKGFHISFVNNHYNHKRLQRSRGLSALEGLPDFHFYSIPDGLPPSNAEATQSIPGLCESIPKHSLEPFCELIATLNGSDVPPVSCIISDGVMSFTLQAAERFGLPEVLFWTPSACGFLAYTHYRDLVDKEYIPLKDTNDLTNGYLETSLDWIPGMKNIRLKDFPSFIRTTDINDIMLNYFLIETEAIPKGVAIILNTFDALEKDSITPVLALNPQIYTIGPLHMMQQYVDHDERLKHIGSNLWKEDVSCINWLDTKKPNSVVYVNFGSITVMTKEQLIEFGWGLANSKKDFLWITRPDIVGGNEAMIPPEFIEETKERGMVTSWCSQEEVLKHPSIGVFLTHSGWNSTIESISNGVPMICWPFFAEQQTNCRYCCVEWEIGLEIDTDVKREEVEAQVREMMDGSKGKMMKNKALEWKKKAEEAVSIGGSSYLNFEKLVTDILLRK

SrUGT85B1-1

ATGGGTTCGGTTCAAGAGAAAAAGGCGCCACATGTTGTGTGCATACCGGCACCACTTCAAGGTCACATTAACCCGATGCTAAAACTAGCCAAAATCCTACACTCCAAAGGCTTTCTTATCACCTTTGTCAACACCGAGTTTAACCACCAACGGCTCGTTAGGTCACAGGGGGTTGAAGCCCTACACGGGCTCCCAACCTTCCGGTTCGAGACCATCCCAGATGGTCTACCGCCACCTGAAAACAAAGATGCCACCCAAGATATCCCGACTCTAGCCAAGTCGGTTGATGAAAACTTTTTGGGTCCGTTTAAAAGTCTTGTAACCAAAGCGGGTGCTTTGTATGCACCCGTGACTTGTATCGTGTCTGACATGCTTATGTGCTTCACTCTTGATGCCGGTGCTGAATTGGATATCCCGGTAATACTCCATTGGACCAGTGGTACTGGTTCTTTGATATGTTACAATGAATATCCTAATCTATTGGAAAGCAAATTGATGCCCCTCAAAGATGCAAGTTATTTAGTGAATGGTTACTTAGATACGATTGTAGATTCTATCCCCATTTTGCATGGCATACGTTTAAGAGATTTCCCTCCCTTCATTAGAAAGATCTTTCCTGGTGATGATTTCATGGTTCAATTTTTGACTTCACAAGTAAACAAAGCAAAAAACGGATCTTCAGCTATCATTTTCAACACTTTTGATGAACTAGATCGTGATGTTTTAGACACACTCACTTCAATGTATCCTACATGTTATGGAATTGGTCCGTTACATCTACTAGAGAAACATGTTACCGATAAATCTCTTGATTTTGTGAAATCAAACCTTTGGAAAGAAGAACCCGAATGTTTAAAATGGTTAGATACACAAGCTCCATCATCAGTCATTTATGTGAATTTTGGTAGCATTACAGTAATGACACCTCAACAACTAGTCGAGTTTTGTTGGGGACTCGCAAAGAGCAACTATCCGTTCTTATGGATAATACGACCTGACCTTGTGATTGGTGATTCCGCGATGCTTCCACCCGAGTTTATAAAGGAAACAAGTGATAGAGGGATGTTGGTTGGATGGTGTCCTCAAGAAGAAGTTTTGAATCACCCGTCAATTGGAGGGTTTTTAACGCACAGTGGATGGAATTCAACGCTTGAAAGTATTTCGAGTGGTGTGCCGATGATTTGTTGGCCGTTTTTTGCGGATCAACAAACGAATTGCTGGTGGAGTTGCAACAAATGGGGTGTTTCCATGGAGATTGATAATAATGTGAAGAGTGATGAAGTTTCAAAGCTTGTGATTGAATTAATGGATGGAGAAAAAGGAAAGGAAATTAAGAAGAATGCCATTGACTTGAAGAATAAAGCTGAGGATGCATGTACCTCTCCTCTGGGTTCATCAGTGGCTAATTTGGAGAAAGTGGTTCAACTGATTCGTACATTTTCAAAATAA

MGSVQEKKAPHVVCIPAPLQGHINPMLKLAKILHSKGFLITFVNTEFNHQRLVRSQGVEALHGLPTFRFETIPDGLPPPENKDATQDIPTLAKSVDENFLGPFKSLVTKAGALYAPVTCIVSDMLMCFTLDAGAELDIPVILHWTSGTGSLICYNEYPNLLESKLMPLKDASYLVNGYLDTIVDSIPILHGIRLRDFPPFIRKIFPGDDFMVQFLTSQVNKAKNGSSAIIFNTFDELDRDVLDTLTSMYPTCYGIGPLHLLEKHVTDKSLDFVKSNLWKEEPECLKWLDTQAPSSVIYVNFGSITVMTPQQLVEFCWGLAKSNYPFLWIIRPDLVIGDSAMLPPEFIKETSDRGMLVGWCPQEEVLNHPSIGGFLTHSGWNSTLESISSGVPMICWPFFADQQTNCWWSCNKWGVSMEIDNNVKSDEVSKLVIELMDGEKGKEIKKNAIDLKNKAEDACTSPLGSSVANLEKVVQLIRTFSK

SrUGT85B1-3

ATGGGTTCGGTTCAAGAGAAAAAGGCGCCACATGTTGTGTGCATACCGGCACCACTTCAAGGTCACATTAACCCGATGCTAAAACTAGCCAAAATCCTACACTCCAAAGGCTTTCTTATCACCTTTGTCAACACCGAGTTTAACCACCAACGGCTCGTTAGGTCACAGGGGGTTGAAGCCCTACACGGGCTCCCAACCTTCCGTTTCGAGACCATCCCAGATGGTCTACCGCCACCTGAAAACAAAGATGCCACCCAAGATATCCCGACTCTAGCCAAGTCGGTTGATGAAAACTTTTTGGGTCCGTTTAAAAGTCTTGTAACCAATGTGGGTGCTTTGTATGCACCCGTGACTTGTATCGTGTCTGACATGCTTATGTGCTTCACTCTTGATGCCGGTGCTGAATTGGATATCCCGGTGATACTCCATTGGACCAGTGGTACTGGTTCTTTGATATGTTACAATGAATATCCTAATCTATTGGAAAGCAAATTGATGCCCCTCAAAGATGCAAGTTATTTAGTGAATGGTTACTTAGATACGATTGTAGATTCTATCCCCATTTTGCATGGCATACGTTTAAGAGATTTCCCTCCCTTCATTAGAAAGATCTTTCCTGGTGATGAGTTCATGGTTCAATTTTTGACTTCACAAGTAAACAAAGCAAAAAACGGATCTTCTGCTATCATTTTCAACACTTTTGATGAACTAGATCGTGATGTTTTAGACACACTCGCTTCAATGTATCCTCCATGTTATGGAATTGGTCCGTTACATCTACTAGAGAAACATGTTACCGATAAATCTCTTGATTTCGTGAAATCAAACCTTTGGAAAGAAGAACCCGAATGTTTAAAATGGTTAGATACACAAGCTCCATCATCAGTCATTTATGTGAATTTTGGTAGCATTACAGTAATGACACCTCAACAACTAGTCGAGTTTTGTTGGGGACTCGCAAAGAGCAACTATCCGTTCTTATGGATAATACGACCTGACCTTGTGATTGGTGATTCCGCGATGCTTCCACCCGAGTTTGTGAAGGAAACAAGTGATAGAGGGATGCTGGTTGGATGGTGTCCTCAAGAAGAAGTTTTGAATCACCCGTCAATTGGAGGGTTTTTAACGCACAGTGGATGGAATTCAACGCTTGAAAGTATTTCGAGTGGTGTGCCGATGATTTGTTGGCCGTTTTTTGCGGATCAACAAACGAATTGCTGGTGGAGTTGCAACAAATGGGGTGTTTCCATGGAGATTGATAATAATGTAAAGAGTGATGAAGTTTCAAAGCTTGTGATTGAATTAATGGATGGAGAAAAAGGAAAGGAAATTAAGAAGAATGCCATTGACTTGAAGAATAAAGCTGAGGATGCATGTACCTCTCCTCTTGGTTCATCAGTGGTTAATTTGGAGAAAGTGGTTCAACTGATTCATACATTTTCAAAATAA

MGSVQEKKAPHVVCIPAPLQGHINPMLKLAKILHSKGFLITFVNTEFNHQRLVRSQGVEALHGLPTFRFETIPDGLPPPENKDATQDIPTLAKSVDENFLGPFKSLVTNVGALYAPVTCIVSDMLMCFTLDAGAELDIPVILHWTSGTGSLICYNEYPNLLESKLMPLKDASYLVNGYLDTIVDSIPILHGIRLRDFPPFIRKIFPGDEFMVQFLTSQVNKAKNGSSAIIFNTFDELDRDVLDTLASMYPPCYGIGPLHLLEKHVTDKSLDFVKSNLWKEEPECLKWLDTQAPSSVIYVNFGSITVMTPQQLVEFCWGLAKSNYPFLWIIRPDLVIGDSAMLPPEFVKETSDRGMLVGWCPQEEVLNHPSIGGFLTHSGWNSTLESISSGVPMICWPFFADQQTNCWWSCNKWGVSMEIDNNVKSDEVSKLVIELMDGEKGKEIKKNAIDLKNKAEDACTSPLGSSVVNLEKVVQLIHTFSK

SrUGT85C1-1

ATGGATCAAATGGCAAAAATTGACGAGAAGAAACCTCATGTGGTGTTCATACCGTTTCCCGCACAAAGTCATATCAAGTGCATGCTCAAACTAGCCAGAATCCTACACCAAAAGGGCCTCTATATAACCTTCATCAACACCGACACGAACCATGAGCGACTCGTAGCCTCTGGTGGGACCCAATGGCTCGAGAATGCTCCTGGTTTTTGGTTCAAAACGGTTCCTGATGGGTTCGGTTCTGCTAAAGACGACGGTGTCAAGCCTACTGACGCTTTACGAGAACTCATGGATTACCTTAAAACCAATTTCTTTGATTTGTTTCTTGATCTTGTACTCAAGCTTGAAGTCCCGGCTACATGCATCATTTGTGATGGTTGCATGACTTTCGCGAACACAATTCGCGCGGCTGAAAAACTTAATATTCCGGTTATTCTTTTCTGGACCATGGCTGCTTGTGGATTCATGGCGTTTTACCAGGCTAAAGTTTTAAAGGAGAAAGAAATTGTCCCAGTTAAAGATGAAACTTATTTGACCAATGGATATCTTGACATGGAAATAGACTGGATTCCTGGAATGAAAAGAATCCGTTTGAGGGATCTACCCGAGTTCATACTGGCCACAAAACAAAATTATTTTGCTTTTGAGTTTTTATTTGAAACCGCTCAATTGGCCGATAAGGTCTCGCATATGATCATCCATACCTTTGAGGAACTTGAGGCTAGTCTTGTGAGTGAGATTAAATCCATATTTCCTAATGTTTACACCATTGGGCCTCTCCAGTTGCTTTTGAACAAAATTACACAAAAAGAAACTAACAACGATAGCTATAGCTTATGGAAGGAAGAACCCGAGTGTGTCGAGTGGCTAAACTCAAAGGAACCGAATTCTGTGGTGTATGTCAACTTTGGAAGTTTGGCGGTGATGTCTTTACAAGATTTGGTAGAATTTGGGTGGGGACTTGTTAATAGCAACCATTATTTTCTTTGGATTATACGCGCTAATTTGATTGATGGGAAGCCGGCGGTTATGCCTCAAGAACTCAAGGAGGCGATGAACGAGAAAGGGTTTGTAGGAAGCTGGTGTTCACAGGAAGAGGTGTTGAACCACCCTGCGGTTGGTGGGTTCTTGACACACTGTGGTTGGGGTTCGATAATTGAAAGCTTGTCAGCTGGAGTGCCAATGCTGGGTTGGCCGTCAATAGGTGACCAACGCGCTAATTGTAGACAAATGTGTAAGGAATGGGAGGTTGGTATGGAGATTGGGAAGAATGTGAAAAGGGATGAAGTTGAGAAGCTTGTGAGGATGTTAATGGAGGGATTGGAGGGTGAACGAATGAGGAAGAAAGCTTTGGAGTGGAAGAAAAGTGCAACACTGGCGACATGTTGTAATGGGTCATCTAGTTTGGATGTAGAGAAACTTGCTAATGAAATCAAGAAGTTATCAAGAAACTAG

MDQMAKIDEKKPHVVFIPFPAQSHIKCMLKLARILHQKGLYITFINTDTNHERLVASGGTQWLENAPGFWFKTVPDGFGSAKDDGVKPTDALRELMDYLKTNFFDLFLDLVLKLEVPATCIICDGCMTFANTIRAAEKLNIPVILFWTMAACGFMAFYQAKVLKEKEIVPLKDETYLTNGYLDMEIDWIPGMKRIRLRDLPEFILATKQNYFAFEFLFETAQLADKVSHMIIHTFEELEASLVSEIKSIFPNVYTIGPLQLLLNKITQKETNNDSYSLWKEEPECVEWLNSKEPNSVVYVNFGSLAVMSLQDLVEFGWGLVNSNHYFLWIIRANLIDGKPAVMPQELKEAMNEKGFVGSWCSQEEVLNHPAVGGFLTHCGWGSIIESLSAGVPMLGWPSIGDQRANCRQMCKEWEVGMEIGKNVKRDEVEKLVRMLMEGLEGERMRKKALEWKKSATLATCCNGSSSLDVEKLANEIKKLSRN

SrUGT85C1-2

ATGGATCAAATGGCAAAAATTGATGAGAAGAAACCTCATGTGGTGTTCATACCGTTTCCCGCACAAAGTCATATCAAGTGCATGCTCAAACTAGCCAGAATCCTACACCAAAAGGGCCTCTATATAACCTTCATCAACACCGACACGAACCATGAGCGACTCGTAGCCTCTGGTGGGACCCAATGGCTCGAGAATGCTCCTGGTTTTTGGTTCAAAACGGTTCCTGATGGGTTCGGTTCTGCTAAAGACGACGGTGTCAAGCCTACTGACGCTTTACGAGAACTCATGGATTACCTTAAAACCAATTTCTTTGATTTGTTTCTTGATCTTGTACTCAAGCTTGAAGTCCCGGCTACATGCATCATTTGTGATGGTTGCATGACTTTCGCGAACACAATTCGCGCGGCTGAAAAACTTAATATTCCGGTTATTCTTTTCTGGACCATGGCTGCTTGTGGATTCATGGCGTTTTACCAGGCTAAAGTTTTAAAGGAGAAAGAAATTGTCCCAGTTAAAGACTGGATTCCTGGAATGAAAAGAATCCGTTTGAGGGATCTACCCGAGTTCATACTAGCCACAAAACAAAATTATTTTGCTTTTGAGTTTTTATTTGAAACCGCTCAATTGGCCGATAAGGTCTCGCATATGATCATCCATACCTTTGAGGAACTTGAGGCTAGTCTTGTGAGTGAGATTAAATCCATATTTCCTAATGTTTACACCATTGGGCCTCTCCAGTTGCTTTTGAACAAAATTACACAAAAAGAAACTAACAACGATAGCTATAGCTTATGGAAGGAAGAACCCGAGTGTGTCGAGTGGCTAAACTCAAAGGAACCGAATTCTGTGGTGTATGTCAACTTTGGAAGTTTGGCGGTGATGTCTTTACAAGATTTGGTAGAATTTGGGTGGGGACTTGTTAATAGCAACCATTATTTTCTTTGGATTATACGCGCTAATTTGATTGATGGGAAGCCGGCGGTTATGCCTCAAGAACTCAAGGAGGCGATGAACGAGAAAGGGTTTGTAGGAAGCTGGTGTTCACAGGAAGAGGTGTTGAACCACCCTGCGGTTGGTGGGTTCTTGACACACTGTGGTTGGGGTTCGATAATTGAAAGCTTGTCAGCTGGAGTGCCAATGCTGGGTTGGCCGTCAATAGGTGACCAACGCGCTAATTGTAGACAAATGTGTAAGGAATGGGAGGTTGGTATGGAGATTGGGAAGAATGTGAAAAGGGATGAAGTTGAGAAGCTTGTGAGGATGTTAATGGAGGGATTGGAGGGTGAACGAATGAGGAAGAAAGCTTTGGAGTGGAAGAAAAGTGCAACACTGGCGACATGTTGTAATGGGTCATCTAGTTTGGATGTAGAGAAACTTGCTAATGAAATCAAGAAGTTATCAAGAAACTAG

MDQMAKIDEKKPHVVFIPFPAQSHIKCMLKLARILHQKGLYITFINTDTNHERLVASGGTQWLENAPGFWFKTVPDGFGSAKDDGVKPTDALRELMDYLKTNFFDLFLDLVLKLEVPATCIICDGCMTFANTIRAAEKLNIPVILFWTMAACGFMAFYQAKVLKEKEIVPVKDWIPGMKRIRLRDLPEFILATKQNYFAFEFLFETAQLADKVSHMIIHTFEELEASLVSEIKSIFPNVYTIGPLQLLLNKITQKETNNDSYSLWKEEPECVEWLNSKEPNSVVYVNFGSLAVMSLQDLVEFGWGLVNSNHYFLWIIRANLIDGKPAVMPQELKEAMNEKGFVGSWCSQEEVLNHPAVGGFLTHCGWGSIIESLSAGVPMLGWPSIGDQRANCRQMCKEWEVGMEIGKNVKRDEVEKLVRMLMEGLEGERMRKKALEWKKSATLATCCNGSSSLDVEKLANEIKKLSRN

SrUGT85C1-3

ATGGATCAAATGGCAAAAATTGACGAGAAGAAACCTCATGTGGTGTTCATACCGTTTCCCGCACAAAGTCATATCAAGTGCATGCTCAAACTAGCCAGAATCCTACACCAAAAGGGCCTCTATATAACCTTCATCAACACCGACACGAACCATGAGCGACTCGTAGCCTCTGGTGGGACCCAATGGCTCGAGAATGCTCCTGGTTTTTGGTTCAAAACGGTTCCCGATGGGTTCGGTTCTGCTAAAGACGACGGTGTCAAGCCTACTGACGCTTTACGAGAACTCATGGATTACCTTAAAACCAATTTCTTTGATTTGTTTCTTGATCTTGTACTCAAGCTTGAAGTCCCGGCTACATGCATCATTTGTGATGGTTGCATGACTTTCGCGAACACAATTCGCGCGGCTGAAAAACTTAATATTCCGGTTATTCTTTTCTGGACCATGGCTGCTTGTGGATTCATGGCGTTTTACCAGGCTAAAGTTTTAAAGGAGAAAGAAATTGTCCCACTTAAAGATGAAACTTATTTGACCAATGGATATCTTGACATGGAAATAGACTGGATTCCTGGAATGAAAAGAATCCGTTTGAGGGATCTACCCGAGTTCATACTAGCCACAAAACAAAATGATTTTGCTTTTGAAGTTTTCTTTGAAACCGCTCAATTGGCCGATAAGGTCTCGCATATGATCATCCATACCTTTGAGGAACTTGAGGCTAGTCTTGTGAGTGAGATTAAATCCATATTTCCTAATGTTTACACCATTGGGCCTCTCCAGTTGCTTTTGAACAAAATTACACAAAAAGAAACTAACAACGATAGCTATAGCTTATGGAAGGAAGAACCCGAGTGTGTCGAGTGGCTAAACTCAAAGGAACCGAATTCTGTGGTGTATGTCAACTTTGGAAGTTTGGCGGTGATGTCTTTACAAGATTTGGTAGAATTTGGGTGGGGACTTGTTAATAGCAACCATTATTTTCTTTGGATTATACGCGCTAATTTGATTGATGGGAAGCCGGCGGTTATGCCTCAAGAACTCAAGGAGGCGATGAACGAGAAAGGGTTTGTAGGAAGCTGGTGTTCACAGGAAGAGGTGTTGAACCACCCTGCGGTTGGTGGGTTCTTGACACACTGTGGTTGGGGTTCGATAATTGAAAGCTTGTCAGCTGGAGTGCCAATGCTGGGTTGGCCGTCAATAGGTGACCAACGCGCTAATTGTAGACAAATGTGTAAGGAATGGGAGGTTGGTATGGAGATTGGGAAGAATGTGAAAAGGGATGAAGTTGAGAAGCTTGTGAGGATGTTAATGGAGGGATTGGAGGGTGAACGAATGAGGAAGAAAGCTTTGGAGTGGAAGAAAAGTGCAACACTGGCGACATGTTGTAATGGGTCATCTAGTTTGGATGTAGAGAAACTTGCTAATGAAATCAAGAAGTTATCAAGAAACTAG

MDQMAKIDEKKPHVVFIPFPAQSHIKCMLKLARILHQKGLYITFINTDTNHERLVASGGTQWLENAPGFWFKTVPDGFGSAKDDGVKPTDALRELMDYLKTNFFDLFLDLVLKLEVPATCIICDGCMTFANTIRAAEKLNIPVILFWTMAACGFMAFYQAKVLKEKEIVPLKDETYLTNGYLDMEIDWIPGMKRIRLRDLPEFILATKQNDFAFEVFFETAQLADKVSHMIIHTFEELEASLVSEIKSIFPNVYTIGPLQLLLNKITQKETNNDSYSLWKEEPECVEWLNSKEPNSVVYVNFGSLAVMSLQDLVEFGWGLVNSNHYFLWIIRANLIDGKPAVMPQELKEAMNEKGFVGSWCSQEEVLNHPAVGGFLTHCGWGSIIESLSAGVPMLGWPSIGDQRANCRQMCKEWEVGMEIGKNVKRDEVEKLVRMLMEGLEGERMRKKALEWKKSATLATCCNGSSSLDVEKLANEIKKLSRN

SrUGT85C2-1

ATGGATGCAATGGCTACAACTGAGAAGAAACCACACGTCATCTTCATACCATTTCCAGCACAAAGCCACATTAAAGCCATGCTCAAACTAGCACAACTTCTCCACCACAAAGGACTCCAGATAACCTTCGTCAACACCGACTTCATCCACAACCAGTTTCTTGAATCATCGGGCCCACATTGTCTAGACGGTGCACCGGGTTTCCGGTTCGAAACCATTCCGGATGGTGTTTCTCACAGTCCGGAAGCGAGCATCCCAATCAGAGAATCACTCTTGAGATCCATTGAAACCAACTTCTTGGATCGTTTCATTGATCTTGTAACCAAACTTCCGGATCCTCCGACTTGTATTATCTCAGATGGGTTCTTGTCGGTTTTCACAATTGACGCTGCAAAAAAGCTTGGAATTCCGGTCATGATGTATTGGACACTTGCTGCCTGTGGGTTCATGGGTTTTTACCATATTCATTCTCTCATTGAGAAAGGATTTGCACCACTTAAAGATGCAAGTTACTTGACAAATGGGTATTTGGACACCGTCATTGATTGGGTTCCGGGAATGGAAGGCATCCGTCTCAAGGATTTCCCGCTGGACTGGAGCACTGACCTCAATGACAAAGTTTTGATGTTCACTACGGAAGCTCCTCAAAGGTCACACAAGGTTTCACATCATATTTTCCACACGTTCGATGAGTTGGAGCCTAGTATTATAAAAACTTTGTCATTGAGGTATAATCACATTTACACCATCGGCCCACTGCAATTACTTCTTGATCAAATACCCGAAGAGAAAAAGCAAACTGGAATTACGAGTCTCCATGGATACAGTTTAGTAAAAGAAGAACCAGAGTGTTTCCAGTGGCTTCAGTCTAAAGAACCAAATTCCGTCGTTTATGTAAATTTTGGAAGTACTACAGTAATGTCTTTAGAAGACATGACGGAATTTGGTTGGGGACTTGCTAATAGCAACCATTATTTCCTTTGGATCATCCGATCAAACTTGGTGATAGGGGAAAATGCAGTTTTGCCCCCTGAACTTGAGGAACATATAAAGAAAAGAGGCTTTATTGCTAGCTGGTGTTCACAAGAAAAGGTCTTGAAGCACCCTTCGGTTGGAGGGTTCTTGACTCATTGTGGGTGGGGATCGACCATCGAGAGCTTGTCTGCTGGGGTGCCAATGATATGCTGGCCTTATTCGTGGGACCAGCTGACCAACTGTAGGTATATATGCAAAGAATGGGAGGTTGGGCTCGAGATGGGAACCAAAGTGAAACGAGATGAAGTCAAGAGGCTTGTACAAGAGTTGATGGGAGAAGGAGGTCACAAAATGAGGAACAAGGCTAAAGATTGGAAAGAAAAGGCTCGCATTGCAATAGCTCCTAACGGTTCATCTTCTTTGAACATAGACAAAATGGTCAAGGAAATCACCGTGCTAGCAAGAAACTAG

MDAMATTEKKPHVIFIPFPAQSHIKAMLKLAQLLHHKGLQITFVNTDFIHNQFLESSGPHCLDGAPGFRFETIPDGVSHSPEASIPIRESLLRSIETNFLDRFIDLVTKLPDPPTCIISDGFLSVFTIDAAKKLGIPVMMYWTLAACGFMGFYHIHSLIEKGFAPLKDASYLTNGYLDTVIDWVPGMEGIRLKDFPLDWSTDLNDKVLMFTTEAPQRSHKVSHHIFHTFDELEPSIIKTLSLRYNHIYTIGPLQLLLDQIPEEKKQTGITSLHGYSLVKEEPECFQWLQSKEPNSVVYVNFGSTTVMSLEDMTEFGWGLANSNHYFLWIIRSNLVIGENAVLPPELEEHIKKRGFIASWCSQEKVLKHPSVGGFLTHCGWGSTIESLSAGVPMICWPYSWDQLTNCRYICKEWEVGLEMGTKVKRDEVKRLVQELMGEGGHKMRNKAKDWKEKARIAIAPNGSSSLNIDKMVKEITVLARN

SrUGT85C2-2

ATGGATGCAATGGCTACAACTGAGAAGAAACCACACGTCATCTTCATACCATTTCCAGCACAAAGCCACATTAAAGCCATGCTCAAACTAGCACAACTTCTCCACCACAAAGGACTCCAGATAACCTTCGTCAACACCGACTTCATCCACAACCAGTTTCTTGAATCATCGGGCCCACATTGTCTAGACGGTTCACCGGGTTTCCGGTTCGAAACCATTCCGGATGGTGTTTCTCACAGTCCGGAAGCGAGCATCCCAATCAGAGAATCACTCTTGAGATCCATTGAAACCAACTTCTTGGATCGTTTCATTGATCTTGTAACCAAACTTCCGGATCCTCCGACTTGTATTATCTCAGATGGGTTCTTGTCGGTTTTCACAATTGACGCTGCAAAAAAGCTTGGAATTCCGGTCATGATGTATTGGACACTTGCTGCCTGTGGGTTCATGGGTTTTTACCATATTCATTCTCTCATTGAGAAAGGATTTGCACCACTTAAAGATGCAAGTTACTTGACAAATGGGTATTTGGACACCGTCATTGATTGGGTTCCGGGAATGGAAGGCATCCGTCTCAAGGATTTCCCGCTGGACTGGAGCACTGACCTCAATGACAAAGTTTTGATGTTCACTACGGAAGCTCCTCAAAGGTCACACAAGGTTTCACATCATATTTTCCACACGTTCGATGAGTTGGAGCCTAGTATTATAAAAACTTTGTCATTGAGGTATAATCACATTTACACCATCGGCCCACTGCAATTACTTCTTGATCAAATACCCGAAGAGAAAAAGCAAACTGGAATTACGAGTCTCCATGGATACAGTTTAGTAAAAGAAGAACCAGAGTGTTTCCAGTGGCTTCAGTCTAAAGAACCAAATTCCGTCGTTTATGTAAATTTTGGAAGTACTACAGTAATGTCTTTAGAAGACATGACGGAATTTGGTTGGGGACTTGCTAATAGCAACCATTATTTCCTTTGGATCATCCGATCAAACTTGGTGATAGGGGAAAATGCAGTTTTGCCCCCTGAACTTGAGGAACATATAAAGAAAAGAGGCTTTATTGCTAGCTGGTGTTCACAAGAAAAGGTCTTGAAGCACCCTTCGGTTGGAGGGTTCTTGACTCATTGTGGGTGGGGATCGACCATCGAGAGCTTGTCTGCTGGGGTGCCAATGATATGCTGGCCTTATTCGTGGGACCAGCTGACCAACTGTAGGTATATATGCAAAGAATGGGAGGTTGGGCTCGAGATGGGAACCAAAGTGAAACGAGATGAAGTCAAGAGGCTTGTACAAGAGTTGATGGGAGAAGGAGGTCACAAAATGAGGAACAAGGCTAAAGATTGGAAAGAAAAGGCTCGCATTGCAATAGCTCCTAACGGTTCATCTTCTTTGAACATAGACAAAATGGTCAAGGAAATCACCGTGCTAGCAAGAAACTAG

MDAMATTEKKPHVIFIPFPAQSHIKAMLKLAQLLHHKGLQITFVNTDFIHNQFLESSGPHCLDGSPGFRFETIPDGVSHSPEASIPIRESLLRSIETNFLDRFIDLVTKLPDPPTCIISDGFLSVFTIDAAKKLGIPVMMYWTLAACGFMGFYHIHSLIEKGFAPLKDASYLTNGYLDTVIDWVPGMEGIRLKDFPLDWSTDLNDKVLMFTTEAPQRSHKVSHHIFHTFDELEPSIIKTLSLRYNHIYTIGPLQLLLDQIPEEKKQTGITSLHGYSLVKEEPECFQWLQSKEPNSVVYVNFGSTTVMSLEDMTEFGWGLANSNHYFLWIIRSNLVIGENAVLPPELEEHIKKRGFIASWCSQEKVLKHPSVGGFLTHCGWGSTIESLSAGVPMICWPYSWDQLTNCRYICKEWEVGLEMGTKVKRDEVKRLVQELMGEGGHKMRNKAKDWKEKARIAIAPNGSSSLNIDKMVKEITVLARN

SrUGT85C3-2

ATGGATGCAGTGGTCGAAACAGAAAAGAAGAAACCACACGTCATCTGTATGCCGTTTCCAGATCTAAGCCACATAAAAGCCATGCTCAAACTAGCCGAGCTTCTCTACCACAAAGGACTACAGATAACCTTCATCAACACCGATTTCGTCCACAACCGGTTTCTTGAATCAGGAGGACCACACTGTTTAGACGGTCCACCTGGTTTCCGGTTTGAAACCATTCCAGATGGTGTTACTCGCAGATCGGAAGCTAGCATCCACAACACCAGAGAACTACTCAAGTCCGTCGAAACCATCTTCTTGGATCGTTTCATTGACCTTGTAACCAAACTTCCGGATCCTCCGACTTGTATTATCTGTGATGGTTTCTTGTCGGTTTTCACAATTGACGCTGCAAAAAAGCTTGGAATCCCGATTATGATGTATTGGACACTTGCTGCCTGTGGCTTCATGGGGTTTTATCAAATGCATTCTCTCATTGAGAAAGGATTTGCACCGCTTAAAGATGAAAGTTATTTGACAAATGGGTATTTAGACACCGTCGTTGATTGGGTTCCGGGAATGGAAAGCATCCGTCTTAAGGAGTTCCCATTGGTTTGGAACACTGACTTGAATGATAAACTTCTAACATTCTGCAAGGAAGCTCCCCAAAGGTGTCACTCGGTTTCAAATCATATTTTCCACACGTTTGATGAGTTAGAGCCTAGTGTTATCAAAGCTTTGTCATCTATATATTCTCGCGTTTACGCCATCGGCCCACTACAACTACTTCTTGATCAGATACCTGAAGAAAAAAAGCAAACTGGAATTTCAGGTTTGAATGGATACAGTTTTGTGAAAGAAGAACCCGAGTGTTTTAAGTGGCTTCAATCTAAGGAACCATATTCTGTCATTTATGTAAATTTTGGGAGTTCAACATTAATGTCTTTAGAAGACTTGATAGAATTTGGTTGGGGACTTGTTAATAGCAACCACTATTTCCTTTGGATCATCCGATCTAACTTGATAAATGGAGAATCTGCAGTTTTGCCTCTTGAATTAGACGAACATATAGAAAAGAGAGGCTTTATTGCAAGCTGGTGTTCACAAGAAAAGGTCTTGAACCACCCTTCGGTTGGAGGGTTCTTGACTCATTGTGGGTGGGGATCGACCATCGAGAGCTTGTCTGCTGGGGTGCCAATGATATGCTGGCCTTATTCGTGGGACCAGCTGACCAACTGTAGGTATATATGCAAAGAATGGGAGGTTGGGCTCGAGATGGGAACCAAAGTGAAACGAGATGAAGTCAAGAGGCTTGTACAAGAGTTGATGGGAGAAGGAGGTCACAAAATGAGGAACAAGGCTAAAGATTGGAAAGAAAAGGCTCGCATTGCAATAGCTCCTAACGGTTCATCTTCTTTGAACATAGACAAAATGGTCAAGGAAATCACCGTGCTAGCAAGAAACTAG

MDAVVETEKKKPHVICMPFPDLSHIKAMLKLAELLYHKGLQITFINTDFVHNRFLESGGPHCLDGPPGFRFETIPDGVTRRSEASIHNTRELLKSVETIFLDRFIDLVTKLPDPPTCIICDGFLSVFTIDAAKKLGIPIMMYWTLAACGFMGFYQMHSLIEKGFAPLKDESYLTNGYLDTVVDWVPGMESIRLKEFPLVWNTDLNDKLLTFCKEAPQRCHSVSNHIFHTFDELEPSVIKALSSIYSRVYAIGPLQLLLDQIPEEKKQTGISGLNGYSFVKEEPECFKWLQSKEPYSVIYVNFGSSTLMSLEDLIEFGWGLVNSNHYFLWIIRSNLINGESAVLPLELDEHIEKRGFIASWCSQEKVLNHPSVGGFLTHCGWGSTIESLSAGVPMICWPYSWDQLTNCRYICKEWEVGLEMGTKVKRDEVKRLVQELMGEGGHKMRNKAKDWKEKARIAIAPNGSSSLNIDKMVKEITVLARN

SrUGT85D1

ATGGAGAACATGAAGATGAAACCTCATGCAGTTTGCATACCATTTCCAGCACAAGGTCACATAAATCCCATGCTAAAACTTGCCAAAATCCTTCATTCCAAAGGCTTCCATATAACCTTCGTCAACACCGAATTCAACCACCGACGACTCCTTAAATCGCAAGGTTCAGAAATGCTAAACGCGTACACATCATTTGAGTTTGAGACCATTCCCGATGGGCTTCCGCCATCTGGAAACCAAGATGTGACCCAAGACATCCCGTCACTATGCATTTCCACCTCTGAAACATGTCTTAAACCTTTTAAAACCCTCCTCTCAAAACTTAATAATAACGCGAATGTACCACCGGTGAGTTGTATAGTTTCTGATGGTATAATGAGCTTCACACTTGACGCGTCGGATGAATTGGGTATCCCTAATGTTCTGTTTTGGACAACAAGCGCTTGTGGCTTTTTGGCTTATGCACACTATATCACTCTCAAGCAAAATGGAATCATTCCTGCAAAAGATTCGGACCATTTATCCAATGGGTACTTGGATACCATAGTTGATTGTATACCTAGCATGAAAGGCATACGCTTGAAGGACATCCCAACGTTCATTAGAACCACAAATCCAAACGAACCCATGATCAATTTTTGCATCCGAGAACCAGCAAGAGCCAAAAAAGCTTCAGCAATAATACTCAACACTTTCGAAGAGCTAGAACAAGAAGTCTTAAACGAGTTATCTTCCATGTATCCTCCTATTTTCACAATCGGACCATTACACAACATTGCAAAAATCGTTGTGGGAAATGATCTGAAATTGTTGGGTTCGAATCTGTGGAAAGAAGACACCGAATGCATTGAATGGCTTGACTCGAAAGAAGCTAATTCGGTTGTTTATGTGAACTTTGGAAGCATAACCGTGATGACACCACAACAACTAGTTGAGTTCTCTTGGGGTCTTGCAAATAGTAACCAGACCTTTTTATGGGTCATCCGACCCGACTTGGTTACTGGAGACTCACGGATTTTGCAACCGGAGTTTCTTGAGGCGACTCGTGAGAGGGGTTTGTTAACAAGTTGGTGCCCGCAAGAAAAGGTTTTAAATCATCCGTCAATTGGAGGGTTTTTGACGCATTGCGGGTGGAACTCGACCATTGAGAGTATATCGAGTGGGGTTCCAATGATTTGTTGGCCGTTTTTCGCGGAACAACAGACTAATTGTTGGTATAGTTGTAATCAATGGGGCATTGGGATGGAGATTGATAGTGATGTGGACAGAAAACAGGTTGAAAAGCTTGTGAGGACATTAATGGTGGAAGAAAAAGGTGAAGAGATGAGAAAGATGGCGATGGTTTGGAAGAAAAAGGCCGAATCCTCATCGTCTTTGTTGAACATAGACAACTTGATCAACCAAGTGCTTCTTTAA

MENMKMKPHAVCIPFPAQGHINPMLKLAKILHSKGFHITFVNTEFNHRRLLKSQGSEMLNAYTSFEFETIPDGLPPSGNQDVTQDIPSLCISTSETCLKPFKTLLSKLNNNANVPPVSCIVSDGIMSFTLDASDELGIPNVLFWTTSACGFLAYAHYITLKQNGIIPAKDSDHLSNGYLDTIVDCIPSMKGIRLKDIPTFIRTTNPNEPMINFCIREPARAKKASAIILNTFEELEQEVLNELSSMYPPIFTIGPLHNIAKIVVGNDLKLLGSNLWKEDTECIEWLDSKEANSVVYVNFGSITVMTPQQLVEFSWGLANSNQTFLWVIRPDLVTGDSRILQPEFLEATRERGLLTSWCPQEKVLNHPSIGGFLTHCGWNSTIESISSGVPMICWPFFAEQQTNCWYSCNQWGIGMEIDSDVDRKQVEKLVRTLMVEEKGEEMRKMAMVWKKKAESSSSLLNIDNLINQVLL

SrUGT85E1

ATGCAGGTCTCTACAATGCCGATGGATCATGAGAAGAAACCCCATGCATTGTTCGTACCATATCCGGCTCAAAGTCACGTCAAATGTATGTTGAAACTAGCATGGCTACTGCACCACAAAGGCATTCAAATAACCTTCGTTAACATCCAGTCCATCCACAACCGGCTCGATCCTGTTGGTGCCTCCGGTTTTCAGTTTAAAATAGTTCCTGATGATTTCCCTAGTTCCGATAGTGTAACGCCGAACCCAGAAATCTTGTTTCGGTATCTCGCCACCGACTTCTTGGGCTCCTTACTTGATGTTGCAGTGGGACTCGAAACTCCCGTCACTTGTATCGTGTCCGATGCTTTGTTTAGACTCATGAAAATCAATCACGTGGCTGAGAGGCTCAAAGTGGCCAACGTAACGCTTTGGCCGGTTGCTGCTTGTGGGTTTCTTAATATATACCGAGCTAAAGTTATGGAGGCAAAAGGAATTCTCCCACTTAAAGATGAAAGTTGCTTGACAAATGGGTACCTTGATATGGAGATAGAGTGGATACCAGGAATGGAAAAATTCCGTCTGCGCGATCTACCAAGGAATTTCTTAGCCATAAAACGTGATGGTCATCAACAAGAGATTATCGAGGAAGAAGATAGGATGGATGACAATTCCTCACACATGATTATCCATACTTTTTATGAACTAGAGGACACTCTTGTTACTAAGCTTCGAACCATTTTTCCTCGAATCTACAACGTCGGGCCTCTGCAGTTGCATCTGAATCAGATGGAGAATTCGAGTTTCAATGGCTATAGTTTACTTAAGGAAGAACCCGAGTGTGTAGAGTGGCTCCAGTCGAAGGAACCCAACTCTGTGTTGTATATCAGCTTTGGAAGCGAGACAGTTATCTCTTCTCAAGAGTTACTGGAATTCGGGTGGGGACTTGTGAATAGTAACCATCATTTTCTTTGGATCATACGTGTCGATTTGGTTGATGGCAAGCCAACGGTTTTGCCTCAAGAACTTGAGGATGCAATTAAAAAAAGAGGGTTTATCGCGAGCTGGTGTTCACAAGAAGAGGTTCTAAACCACCCTTCGATTGGTGGGTTCTTAACACATGGTGGGTGGGGTTCGGTGATTGAGAGCTTGTCGGCTGGGGTGCCGATGATTTGTTTTCCAAGCTCCCATGATCAACCGATTAACTGTAAATACGTGTGTGAGAAATGGGAGGTTGGCATAGAGGTCAAGAGTCCACTAAAGAGGGGTGAAGTTGAAGCACTTGTAAGTACTTTAATGGCAGGGATTGAGGGTAAGAGAATGAGAAAGAAGGCAACGGAGTGGAAGCGACTGGCCGAAATCGCGACACGTCCCGGTGGCTCATCATGTTCAAGCATTCAGAAACTTGTTAACGAAATTACCTTGATGTCAAGAAAGTAG

MQVSTMPMDHEKKPHALFVPYPAQSHVKCMLKLAWLLHHKGIQITFVNIQSIHNRLDPVGASGFQFKIVPDDFPSSDSVTPNPEILFRYLATDFLGSLLDVAVGLETPVTCIVSDALFRLMKINHVAERLKVANVTLWPVAACGFLNIYRAKVMEAKGILPLKDESCLTNGYLDMEIEWIPGMEKFRLRDLPRNFLAIKRDGHQQEIIEEEDRMDDNSSHMIIHTFYELEDTLVTKLRTIFPRIYNVGPLQLHLNQMENSSFNGYSLLKEEPECVEWLQSKEPNSVLYISFGSETVISSQELLEFGWGLVNSNHHFLWIIRVDLVDGKPTVLPQELEDAIKKRGFIASWCSQEEVLNHPSIGGFLTHGGWGSVIESLSAGVPMICFPSSHDQPINCKYVCEKWEVGIEVKSPLKRGEVEALVSTLMAGIEGKRMRKKATEWKRLAEIATRPGGSSCSSIQKLVNEITLMSRK

SrUGT86B1

ATGGCTGATCGGAGAACTGAAAATCCTCATGCCATATTAATACCATTACCTCTTCAAGGTCACCTCATCCCTTTCGTTCATCTTGCCATCAAGTTAGCATCTAAAGGATTCACCATCACCTTCATCAACACCGAATCAGTCCACCATAGCATCACCAACTCCTCACCGGAATATTCCACCGGCAGCGATCTCTTTGCAGATGCACATAAATCCGGTCTCGATATAAGGTACGCCACCGTGAGTGATGGTCTTCCGGTAGGGTTTGATCGATCCCTGAACCATGACCAGTTCATGGAGTGTCTTTTTCATGTGTTTTCGGCTCACGTTGATGAAATTGTGGGAAATTTGGTGAAACATGATCCGTCGATCAGCTGTTTGGTAGCTGATAGCTTTTATGTTTGGCCGTTGATGATCTCAAAGAAGTATAAGTTGGTTAATATATCATTCTGGACTGAACCTGCTTTGGTTTTGAATTTGTATTATCATTTGGATTTGCTAAAGAAAAATGGTCACTTTGATCCACTTGATAAGTATGATGATGTCATAGATTACATACCCGGGGTCGTATCAATCAAACCAACAGACATGATGTCATATCTTCAAGCCACTAACACGAACACCATAGTCCATAGAATCATACGTAAGGCGTTGTTTGAAGTAACAAGGAGTGCTGATATCATCATATGTAACACAATACAAGAACTTGAACCTAATACAATTTCAGCAATGAATCAAATACAACCTTTTTATGCAATCGGTCCAATTTTCCCTAATGACTTCACTCGAGAACTTGTGTCGACAAGCTTATGGTGCGAGCAAGATTGCACCCGTTGGCTCGATAGTAAACCTCCCGGTTCAGTTTTGTATGTATCATTTGGTAGTTATGCTCACATTAGCAAACATGATCTTGAAGAGATAGCTTATGGTCTCCTACGAAGTGGTGTCAATTTTGTTTGGGCCCTTCGGCCCGATATAGTGAGTTCTAGTGACCCAAGTGCTTTGCCTATTGGATTTGAAGCCCAAGTTAAAGATCAAGGGTTGATTGTTCCTTGGTGTCATCAAAAGACGGTCCTTTCACATCTGTCGGTAGGTGGGTTTCTGACACATTGCGGGTGGAACTCGATTTTGGAAAGCGTTTGGTGTGGTGTACCTTTGATTTGTTTTCCACTTTTAACGGATCAATTCACTAATAGGAAATTAGTAGTTGATGATTGGAAAATTGGGATAAATTTATGTGAAAGGAGGAATTTGGTTGACAGAGAAGAAGTGGAAAAGAAAGTGAAAGAATTGATGCAAGAGAAGTCATATGAATTTAGGGTTGAAGTCAAGAAAGTTAAAAGAACACTAGAAGATGCATTGGGTACGGGTGGATCATCTCAGATAAATCTGGACCAATTCATTAGTCAAGTTATAGTAAAAGCTAAACAAATAAAGTGA

MADRRTENPHAILIPLPLQGHLIPFVHLAIKLASKGFTITFINTESVHHSITNSSPEYSTGSDLFADAHKSGLDIRYATVSDGLPVGFDRSLNHDQFMECLFHVFSAHVDEIVGNLVKHDPSISCLVADSFYVWPLMISKKYKLVNISFWTEPALVLNLYYHLDLLKKNGHFDPLDKYDDVIDYIPGVVSIKPTDMMSYLQATNTNTIVHRIIRKALFEVTRSADIIICNTIQELEPNTISAMNQIQPFYAIGPIFPNDFTRELVSTSLWCEQDCTRWLDSKPPGSVLYVSFGSYAHISKHDLEEIAYGLLRSGVNFVWALRPDIVSSSDPSALPIGFEAQVKDQGLIVPWCHQKTVLSHLSVGGFLTHCGWNSILESVWCGVPLICFPLLTDQFTNRKLVVDDWKIGINLCERRNLVDREEVEKKVKELMQEKSYEFRVEVKKVKRTLEDALGTGGSSQINLDQFISQVIVKAKQIK

SrUGT88B1-2

ATGGAGTCCTCTAAGGTGATCCTGTATCCTTCTCCCGGAATCGGCCATCTTGTTTCCATGGTGGAGCTTGGAAAACTCATCCACACCCACCACCCTTCACTCTCCGTTATCATCCTCGTACTTCCGGCTACATATGAAACCGGGTCCACCACTACATACATCAACACCGTCTCCACCACCACCCCCTCCGTCACCTTCCACCACCTCCCCGTTATCCCTCTTCCACCAGACTCATCTTCTGAATTCATAGACCTTGCCTTCGATATCCCTCAACTTTACAACCCGGTCGTCTACAACACCCTCGTAGCCATCTCCGAAACCTCAACCATCAAAGCTGTCATCCTTGATTTCTTTGCAAACGCAGCTTTTCAGATCTCTAAAAGTCTCGATCTTCCCACTTACTACTTCTATACCAGTGGTGCCTCTGGTCTCTGTGCGTTCTTACATTTTCCAACCATCTACAAAACATATTCCGGAAACTTTAAAGATCTAGATACTTTCATTAATATTCCCGGGGTACCTCCCATTCATTCTTCCGATATGCCCACGGTTATGTCTGATAAGGAAAGTAATTCCTACAAAAACTTCGTAAAAACCTCAAATAACATGGCAAAATCTTCCGGAGTCATTGCAAACAGCTTCTTGCAGTTGGAGGAAAGAGCTGCTCAAACTCTCCGAGATGGTAAATCCATCACGGACGGTCCCTCTCCACCTATTTATCTAATCGGGCCTTTAATCGCTAGCGGCAATCAAGTTGATCATAACGAAAACGAGTGTCTAAAATGGCTGAACACACAACC

TAGTAAAAGTGTAGTGTTTTTGTGCTTTGGGAGCCAGGGTGTGTTTAAGAAAGAACAATTGAAGGAAATAGCGGTTGGGTTAGAGAGAAGTGGGCAAAGATTTTTATGGGTGGTGCGAAAGCCGCCATCAGATGACGGTTCAGGTGGTAAAGAGTTCGGTCTTGATGATGTTCTTCCTGAAGGGTTTGTAGCCAGGACTAAAGAAAAGGGTCTGGTGGTGAAGAACTGGGCGCCTCAACCAGCGATTCTTGGTCATGAATCGGTGGGAGGATTTGTGAGTCATTGCGGGTGGAACTCGTCGCTTGAAGCGGTTGTTTTTGGTGTGCCGATGGTGGCATGGCCGTTGTACGCAGAGCAGAAGATGAACAGAGTGTATTTGGTTGAGGAAATAAAGGTGGCACTTTGGTTGAGAATGTCGGTAGATGGGTTTGTGAGTGCAGAGGCGGTGGAGGAGACGGTGAGACAGTTAATGGATGGGAGAAGAGTGAGAGAACGGATTTTGGAGATGAGTACAAAAGCGAAGGCTGCGGTGGAGGACGGCGGTTCCTCTCGAGTTGATTTCTTCAAATTAACTGAGTCATGGACCCACAAGTGA

MESSKVILYPSPGIGHLVSMVELGKLIHTHHPSLSVIILVLPATYETGSTTTYINTVSTTTPSVTFHHLPVIPLPPDSSSEFIDLAFDIPQLYNPVVYNTLVAISETSTIKAVILDFFANAAFQISKSLDLPTYYFYTSGASGLCAFLHFPTIYKTYSGNFKDLDTFINIPGVPPIHSSDMPTVMSDKESNSYKNFVKTSNNMAKSSGVIANSFLQLEERAAQTLRDGKSITDGPSPPIYLIGPLIASGNQVDHNENECLKWLNTQPSKSVVFLCFGSQGVFKKEQLKEIAVGLERSGQRFLWVVRKPPSDDGSGGKEFGLDDVLPEGFVARTKEKGLVVKNWAPQPAILGHESVGGFVSHCGWNSSLEAVVFGVPMVAWPLYAEQKMNRVYLVEEIKVALWLRMSVDGFVSAEAVEETVRQLMDGRRVRERILEMSTKAKAAVEDGGSSRVDFFKLTESWTHK

SrUGT88C1

ATGGCTACCATAGTTTTGTATCCATCTCCAGCTATGGGTCACCTCATATCAATGGTGGAACTCGGAAAACTCATCACCAAACACCACCCATCTTTCTCCATCATCGTCCTCACTCTCATCCCCTCTTTCAACACCGGCACCACTGCCGCCTACGTCCGTCGCATCTCCGCCACTTTCCCCGCCATCACCTTCCACCACCTCCCCGACATCCCTCTTGACCCCCTACTTTTTCCGTCAATGGAAGCCATCATCGTCGAGCTTATTCGCCATAGCAATCCAAACGTCAAAAACGCACTTGAATCGATTTCTGCATCGTCGTCTAACATCTCCGCGTTTGTAATCGACACTTTTTGCACTCCGTCGATGCCGGTGGCGGATCAATTTAACCTACCGGTTTACTACTTCTTCACTTCCGGTGCTTGCTGTCTCGCACAGTTGCTTTACTTTCCGACGCTTGATAAAACCACGACTGAAAGTTTTAAAGATATGAACAAACTCATCCATTCGCCGGGGTTGCCACCGATACCGTCGTCGGAGATGGTGAGTACGATTCTGGATCGAACATCAATCGATTATTCCAATTTTCTCGAAAAATGCAAGCAATTTCCCAAATCAGCTGGAATCATCATCAACACATTTGAATCGCTGGAGCCAAAAGCGATTAAAGCAATAACTGACGGAGATTGTGTACCGGATCAGCCTACGCCGCCGATCTACTGTGTCGGACCGTTGGTGGCCACCGGTGGCGATGGTTCGCATGAGTGTTTGAGTTGGCTCGATTCGCAACCGAGTCGGAGTGTGGTGTATTTGTGTTTCGGGAGTTTGGGTGTGTTTTCGATTGAGCAGTTGAAGGAGATTGCAAACGGGTTGGAGATGAGTGGTCACCGGTTTCTTTGGGTGGTGAGAAGTCCACCGTCGGAGAAAAAAGAAGACCGGTTTCTGCCACCGCCGGAGCCGGAGTTGGATGTATTATTGCCGGAAGGTTTCTTGGATAGAACAAAAGATAGAGGGCTCGTTGTGAAGAAATGGGCCCCACAAGTGGTGGTTCTCGGCCACGAGTCGATAGGCGGGTTCGTGACGCACTGCGGATGGAACTCGGTTTTGGAAGCGGTTAGTTTCGGGGTTCCGATGGTGGCGTGGCCCCTATACGCAGAACAAAGGTTTAATAGGGTGGTGTTGAGAGATGAAATGAAGCTGGCATTATCAATGGACGAGTCGGACAATGGTATGGTGACGGCAGCAGAGGTGGAGAGGCGGGTTCGACAGTTAATGGAGACGGATGAAGGTGAAAATGTTAGGGCGATGGCCGCCGTGAGAAAAGTGGAGGCGGCGGCAGCGATTAGCGACGGTGGATCTTCTCGGATGGCTTTGGCAAAGTTAGTTGCATTGTGGTAG

MATIVLYPSPAMGHLISMVELGKLITKHHPSFSIIVLTLIPSFNTGTTAAYVRRISATFPAITFHHLPDIPLDPLLFPSMEAIIVELIRHSNPNVKNALESISASSSNISAFVIDTFCTPSMPVADQFNLPVYYFFTSGACCLAQLLYFPTLDKTTTESFKDMNKLIHSPGLPPIPSSEMVSTILDRTSIDYSNFLEKCKQFPKSAGIIINTFESLEPKAIKAITDGDCVPDQPTPPIYCVGPLVATGGDGSHECLSWLDSQPSRSVVYLCFGSLGVFSIEQLKEIANGLEMSGHRFLWVVRSPPSEKKEDRFLPPPEPELDVLLPEGFLDRTKDRGLVVKKWAPQVVVLGHESIGGFVTHCGWNSVLEAVSFGVPMVAWPLYAEQRFNRVVLRDEMKLALSMDESDNGMVTAAEVERRVRQLMETDEGENVRAMAAVRKVEAAAAISDGGSSRMALAKLVALW

SrUGT89A1

ATGATTTTCATACCTAATATTTTTATATTTTTGTCAACCCTGAAATCCGTTGTTATTTCCACTCCTCTTTTTCTCTCCCTCTTGCAAACCATGGCGACCTCAAAAAAAAGCTCACATATACTCGTGTTTCCATTTCCAGCACAAGGTCACATGCTTCCACTTCTAGACCTGACCCACCATCTAGCCACCCATGGTCTAACCATAACCATCTTGGTCACACCCAAAAACCTCCCAATCTTGAACCCACTTCTCTCATCTTCTCCCAACATCGAACCACTGGTCTTACCGTTCCCACCACACCCATCACTTCCATCCAGTGTAGAAAACGTCAAAGACATCGGGAACCATGGAAACGTATCCATCATCAATTCATTGGCTAAACTCAAAGACCCGATCATCAAATGGTTCAACAATCACCCGAACCCTCCTGTTGCCATCGTTCATGACTTCTTCCTCGGATGGACCCATCAGTTAGCTGATAAGTTACGGGTCCCCCGATTCTGTTTCTACTCATCCGGTGCGTTCTTAACCGCGGTTCTTGACTACTGCTGCAAGAATATATCATTCGTTCGATCACAGGACCTAACCGTCTTTCACGATCTGCCCAATTCGCCTAGTTTTCCTAGGGAGCATCTTCCATCTCTGATTCGGGTCTACAAAGAATCCGACCCGGAATGGGAATTGGTCATGGATGGTCATAGAGCACACGCATCGTGTTCGGGTTGGATTATGAATACCTTTGATGACTTGGAGTCTAGGTACATGGATTATTTGAGTAAGTTATTGGGACACGGGAGGGTTTTTGGAGTCGGGCCGGTTAGTTTACTTTCCGGATCAGATCCCATGACCCGTGGGCAATCAGAATCGGGTTCGGACTCAGATGTGGTCAGATGGCTCGATGACAGACCGGATGGGTCGGTTGTGTACGTGTGCTTTGGTAGCCAGAAGTTTCTTACAAGTGACCAAATGGAGGCTTTGGCTATTGGGCTTGAAGATAGTGGGGTCCATTATGTTTGGGTTGTGAAACCTGAACAAGGTGATTCGGTTCAAGTTGGGTCGGGTAGAGGGTTTGTGATAAAGGGTTGGGCTCCTCAAGTGACGATACTGAGTCACCGAGCCGTGGGTGGGTTTCTGAGTCACTGTGGGTGGAATTCAGCTTTGGAAGCGATTTTGGCTGGAGTAATGATACTAGCTTGGCCCATGGAGGCTGACCAGTATATTAATGCTAGGTTGTTAGTTGAGGACCATGGTGCGGCTGTGCGGGTTTGTGAAGGACCGAATACCGTACCTGACTCGGCCAAGTTGGCTCGAACAATTGCTGAGTCAATGAGTGAGGATAAAAGTGAGAAGTTGAAGGCAAAAGAATTGAAAAACAAAGCAATTGAAGCAGTAAAGGAGGGTGGGAGCTCATCAATGGAATTGGACAGGCTTGTTTTAGAGTTGGTTAACTTGGGTAAAAAATGA

MIFIPNIFIFLSTLKSVVISTPLFLSLLQTMATSKKSSHILVFPFPAQGHMLPLLDLTHHLATHGLTITILVTPKNLPILNPLLSSSPNIEPLVLPFPPHPSLPSSVENVKDIGNHGNVSIINSLAKLKDPIIKWFNNHPNPPVAIVHDFFLGWTHQLADKLRVPRFCFYSSGAFLTAVLDYCCKNISFVRSQDLTVFHDLPNSPSFPREHLPSLIRVYKESDPEWELVMDGHRAHASCSGWIMNTFDDLESRYMDYLSKLLGHGRVFGVGPVSLLSGSDPMTRGQSESGSDSDVVRWLDDRPDGSVVYVCFGSQKFLTSDQMEALAIGLEDSGVHYVWVVKPEQGDSVQVGSGRGFVIKGWAPQVTILSHRAVGGFLSHCGWNSALEAILAGVMILAWPMEADQYINARLLVEDHGAAVRVCEGPNTVPDSAKLARTIAESMSEDKSEKLKAKELKNKAIEAVKEGGSSSMELDRLVLELVNLGKK

SrUGT89A2-1

ATGCCGATCTCAGAGAAAAACTCACATGTACTCGTATTCCCATTTCCTGCACAAGGTCACATGCTCCCACTTCTGGACCTAACCCACCAACTAGCCACCCACGGTCTAACCATAACCATCTTGGTCACACCCAAAAACCTCCCCATCTTGAACCCACTTCTCTCATCTTCACCCAACATTCAACCCCTGGTCCTCCCCTTTCCACCGCACCCATCTCTCCCATCCGGTGTAGAACACGTCAGTCAAACCGGAATCCATGGAAACGTCCCCATCATTAACGCACTCGCTAAACTACACGAACCAATCATCCAATGGTTCAATTCCCACCCGAACCCACCTGTCGCCATCCTCCACGACTTCTTCCTCGGTTCGATCAATCACTTAGCCGATAAGTTACAGGTCCCGATGTTCTCTTTCTTCTCCTCCGGTGCGTTCTTCACCGCAGCGGTCAACTACTGCAGCAAGAACATATCACCGGTTCCATCTGATGACCCAATCGTCTTGCAAGATCTACCCAATTCGCCTACTTTCGTATGGGACCAAATTCCAAACCCGTTTCGGATCCACACCGAAGCCAACCCACCAAGGAAATTCGTCAAAGATGGGATTATCTCGAATGAATTAAGTTCAGGTTGGGTTGTTAATACCTTCGATGCGTTAGAATCTCCGTACATCGAACATTTAACAAACTTATTAGGCGATGGGAAGGTTTTTGGAGTCGGGCCGATTAGTTATCTTTCCGGGTCGGATCCCATGACACGTGGGCAATCGCAATCGGAATCGGGTTCGGATTTGGATGTGACTCAATGGCTCGACGGGAAGCCTGATGGGTCGGTTGTGTACGTGTGTTTTGGTAGTCAAAAGTTTCTTTCAAGTGGTCAAATGAACGCGTTGACTATTGGACTTGAAGATAGTGGGATTCATTATGTTTGGGTTGTGAAAACCGAAGAAGGTGTTTCGGTCCCATCCGGGTCGGATAGAGGATTTGTGATATCGGGTTGGGCTCCACAACTCGCGATATTGAATCACCGAGCCGTGGGTGGGTTTCTGAGTCACTGCGGTTGGAACTCGGTTTTGGAATCGATTTTAGCCGGAGTGATGATACTCGCTTGGCCAATGGAGGCTGACCAATATGTTAACGCTAGGTTGTTAGTTGATTCTCTTGGTGCGGCTGTGAAGGTTTGTGAAGGTACGGATACGGTACCCGACTCGACTGAGTTGACTCGCGTTATTGCTGAGTCAATGAGTGGTGATAACAATGAGAAGTTGAAGGCAAAAGAGTTGAAAAAGAAAGCAATTGAAGCAGTAAATGAAGGTGGGAGCTCATCAAAAGAGTTGGCAAGACTTGTTAAGGAGTTGGCTAACTTGCGCCAAAAATAA

MPISEKNSHVLVFPFPAQGHMLPLLDLTHQLATHGLTITILVTPKNLPILNPLLSSSPNIQPLVLPFPPHPSLPSGVEHVSQTGIHGNVPIINALAKLHEPIIQWFNSHPNPPVAILHDFFLGSINHLADKLQVPMFSFFSSGAFFTAAVNYCSKNISPVPSDDPIVLQDLPNSPTFVWDQIPNPFRIHTEANPPRKFVKDGIISNELSSGWVVNTFDALESPYIEHLTNLLGDGKVFGVGPISYLSGSDPMTRGQSQSESGSDLDVTQWLDGKPDGSVVYVCFGSQKFLSSGQMNALTIGLEDSGIHYVWVVKTEEGVSVPSGSDRGFVISGWAPQLAILNHRAVGGFLSHCGWNSVLESILAGVMILAWPMEADQYVNARLLVDSLGAAVKVCEGTDTVPDSTELTRVIAESMSGDNNEKLKAKELKKKAIEAVNEGGSSSKELARLVKELANLRQK

SrUGT89A2-2

ATGCCGATCTCAGAGAAAAACTCACATGTACTCGTATTCCCATTTCCTGCACAAGGTCACATGCTCCCACTTCTCGACCTAACCCACCAACTAGCCACCCACGGTGTAACCATAACCATCTTGGTCACACCCAAAAACCTCCCCATCTTGAACCCACTTCTCTCATCTTCACCCAACATTCAACCCCTGGTCCTCCCCTTTCCACCGCACCCATCTCTCCCATCCGGTGTAGAACACGTCAGTCAAACCGGAATCCATGGAAACGTCCCCATCATTAACGCACTCGCTAAACTACACGAACCAATCATCCAATGGTTCAATTCCCACCCGAACCCACCTGTCGCCATCCTCCACGACTTCTTCCTCGGTTCGATCAATCACTTAGCCGATAAGTTACAGGTCCCGATGTTCTCTTTCTTCTCCTCCGGTGCGTTCTTCACCGCAGCGGTCAACTACTGCAGCAAGAACATATCACCGGTTCCATCTGATGACCCAATCGTCTTGCAAGATCTACCCAATTCGCCTACTTTCGTATGGGACCAAATTCCAAACCCGTTTCGGATCCACACCGAAGCCAACCCACCAAGGAAATTCGTCAAAGATGGGATTATCGCGAATGAATTAAGTTCAGGTTGGGTTGTTAATACCTTCGATGCGTTAGAATCTCCGTACATCGAACATTTAACAAACTTATTAGGCGATGGGAAGGTTTTTGGAGTCGGGCCGATTAGTTATCTTTCCGGGTCGGATCCCATGACACGTGGGCAATCGCAATCGGAATCGGGTTCGGATTTGGATGTGACTCAATGGCTCGACGGGAAGCCTGATGGGTCGGTTGTGTACGTGTGTTTTGGTAGTCAAAAGTTTCTTTCAAGTGGTCAAATGAACGCATTGACTATTGGACTTGAAGATAGTGGGATTCATTATGTTTGGGTTGTGAAAACCGAAGAAGGTGTTTCGGTCCCATCCGGGTCGGATAGAGGATTTGTGATATCGGGTTGGGCTCCACAACTCGCAATATTGAATCACCGAGCCGTGGGTGGGTTTCTGAGTCACTGCGGTTGGAACTCGGTTTTGGAATCGATTTTAGCCGGAGTGATGATACTCGCTTGGCCAATGGAGGCTGACCAATATGTTAACACTAGGTTGTTAGTTGATTCTCTTGGTGCGGCTGTGAAGGTTTGTGAAGGTACGGATACGGTACCCGACTCGACTGAGTTGACTCGCGTTATTGCTGAGTCAATGAGTGGTGATAACAATGAGAAGTTGAAGGCAAAAGAGTTGAAAAAGAAAGCAATTGAAGCAGTAAATGAAGGTGGGAGCTCATCAAAAGAGTTGGCAAGACTTGTTAAGGAGTTGGCTAACTTGCGCCAAAAATAA

MPISEKNSHVLVFPFPAQGHMLPLLDLTHQLATHGVTITILVTPKNLPILNPLLSSSPNIQPLVLPFPPHPSLPSGVEHVSQTGIHGNVPIINALAKLHEPIIQWFNSHPNPPVAILHDFFLGSINHLADKLQVPMFSFFSSGAFFTAAVNYCSKNISPVPSDDPIVLQDLPNSPTFVWDQIPNPFRIHTEANPPRKFVKDGIIANELSSGWVVNTFDALESPYIEHLTNLLGDGKVFGVGPISYLSGSDPMTRGQSQSESGSDLDVTQWLDGKPDGSVVYVCFGSQKFLSSGQMNALTIGLEDSGIHYVWVVKTEEGVSVPSGSDRGFVISGWAPQLAILNHRAVGGFLSHCGWNSVLESILAGVMILAWPMEADQYVNTRLLVDSLGAAVKVCEGTDTVPDSTELTRVIAESMSGDNNEKLKAKELKKKAIEAVNEGGSSSKELARLVKELANLRQK

SrUGT89B2

ATGCCGATTTCCGACATTAACGCCGGATCTCACATCCTGGTTTTCCCGTATCCGGCGCAGGGTCACATGCTCACACTCCTCGATCTTACCCACCAATTAGCCATCAGAAACCTCACCATCACCATACTCGTCACGCCCAAAAACCTACCTACCATTTCTCCGCTTCTCGCCGCCCATCCGACCACCGTCTCCGCCCTCCTCCTCCCTCTTCCACCGCACCCCGCCATCCCCTCCGGCATCGAGAACGTCAAAGACCTTCCTAACGACGCCTTCAAAGCCATGATGGTCGCACTCGGAGACCTCTATAACCCCCTTCGAGATTGGTTTCGTAATCAACCGAATCCGCCGGTAGCCATCATCTCCGACTTCTTCTTGGGCTGGACCCACCACCTCGCCGTCG

AGCTCGGTATCCGCCGGTACACGTTTTCGCCGTCTGGTGCGTTAGCTCTTTCTGTCATATTTTCTCTCTGGCGATACCAACCCAAACGGATTGATGTAGAAAATGAAAAGGAGGCGATCAAGTTCCCGAAAATTCCGAACTCGCCGGAATATCCTTGGTGGCAGCTGTCTCCGATTTATCGGAGCTATGTCGAAGGAGATCCAGATTCGGAATTTATCAAAGATGGGTTTTTGGCTGATATAGCGAGTTGGGGGATTGTGATCAACTCGTTCACTGAGTTGGAACAAGTTTACGTTGACCATTTAAAACACGAACTCGGGCATGATCAGGTGTTTGCGGTCGGACCGTTACTCCCTCCGGGTGACAAGACATCCGGTCGAGGTGGGTCAAGCTCGAATGACGTGCTGTCATGGCTCGACACGTGTGCTGATCGGACGGTGGTGTACGTTTGTTTCGGAAGTCAAATGGTGTTGACGAATGATCAAATGGAAGTCGTCGCACTCGGGTTGGAAAAAAGCCGGGTCAAGTTCGTGTGGTCCGTTAAAGAACCAACCGTCGGACACGAAGCCGCGAATTACGGTCGGGTTCCACCGGGGTTTGAAGATCGGGTGTCGGGTAGGGGTCTTGTGATTCGAGGTTGGGTTCCCCAAGTGGCTATACTGAGTCATGACTCGGTAGGTGTGTTCTTGACTCACTGTGGATGGAACTCGGTCATGGAAGCGGTGGCTGCGGAGGTTTTGATGCTGACGTGGCCGATGAGTGCTGACCAGTTCTCAAATGCCACGTTGTTGCATGAGTTGAAAGTGGGAATCAAAGTATGTGAGGGATCGAATATTGTTCCTAACTCCGACGAGTTAGCCGAGTTGTTTAGTAAATCACTGAGTGACGAGACACGTTTGGAAAGAAAACGAGTTAAGGAGTTTGCGAAGTCAGCAAAGGAAGCAGTTGGTCCTAAAGGGAGTTCAGTCGGTGAGTTGGAGAGATTAGTTGCCAATTTGTCACTATAA

MPISDINAGSHILVFPYPAQGHMLTLLDLTHQLAIRNLTITILVTPKNLPTISPLLAAHPTTVSALLLPLPPHPAIPSGIENVKDLPNDAFKAMMVALGDLYNPLRDWFRNQPNPPVAIISDFFLGWTHHLAVELGIRRYTFSPSGALALSVIFSLWRYQPKRIDVENEKEAIKFPKIPNSPEYPWWQLSPIYRSYVEGDPDSEFIKDGFLADIASWGIVINSFTELEQVYVDHLKHELGHDQVFAVGPLLPPGDKTSGRGGSSSNDVLSWLDTCADRTVVYVCFGSQMVLTNDQMEVVALGLEKSRVKFVWSVKEPTVGHEAANYGRVPPGFEDRVSGRGLVIRGWVPQVAILSHDSVGVFLTHCGWNSVMEAVAAEVLMLTWPMSADQFSNATLLHELKVGIKVCEGSNIVPNSDELAELFSKSLSDETRLERKRVKEFAKSAKEAVGPKGSSVGELERLVANLSL

SrUGT90B1

ATGGCTTCATCACTTCCCGTTCCTACGGTTGGTTCGACACACCGACCTCACATAGTCTTGTTTCCGTTCATGTCTAAAGGTCACACCATCCCACTACTCCAACTAGCCCGGTTGCTCGTGAACCGTGGTTCCATGGTCACTGTTTTCACCACCAAAGCTAACCACCCATTCGTGGCTCAATTTCTTGAGCCCTACCCAATTGGTTCCATCTCGATCATAGACCTTAAGTTCCCTAACCATGTCGAGGGTCTACCGGAAGGCGTCGAGAGCACCGATAAGCTACCTTCGATCAAGCTTTTTAGGCAATTTGCGGTCGCAACAAAACTCATGCAGCCTCAATTTGAACAATCACTCGAGAAACTATCGAATGTTACGTGCATCCTTTCCGATGGTTTTTTGAGTTGGACGCTTGCATCGGCAAACAAGTTTGGGATCCCTCGGATGACGTTTTACGGGATGAACGCTTATTCAAGTGCCGTGTCTAAAGATGTCAGTTTAAGCCTGTGGGGACCCGAGTCGGATGATGAACTAATCACCGCCCCCGGGTTCCCGTGGATCAAAGTCACTAGAAATGACTTTGACGAGCCGTTTAATAAGCGTGATTCAACGAGTCCCCTTTTTGAATTCGTAATGGAGGCGGTTATAGCCTCGGCTAACAGTTATGGTCTGATCTTAAACAGCTTCTACGAGCTAGAGCCATTGTTCGTAGACTACTTAAACCGCGAGTCCAAACCCAAAGCTTGGTGTCTCGGACCACTTTGTCTAGTTGACACACTAGACAGTGCAGATAACAAACAAACAAACGTACCCAGTAATATTATTAGTACCTCACCGGATCAGAAACCGAAAGTCCCGGAATGGATAGAGTGGCTAGACCAAAAGCTAGCCAAAGGAAGCTCGGTTTTATACGTCGCGTTCGGTTCACAAGCCGAGATCTCAATCCAACAATTGGAAGCCATATCCAAAGGTTTGGAACAATCAGAAGTGAACTTCTTGTGGGTGGTGAGAAAGTGTGATCAAACAAATGTTCTCGACGAGCTTGAACAGCGAGTAGGCGAAAGAGGGATGATAGTTAACCAATGGGTAGACCAAAGGGATATTTTGAACCACGAGAGCGTGAACGGGTTCGTGAGTCACTGTGGTTGGAACTCAGCTCTAGAGAGCATATGTTCGGGGGTACCGATTCTAGCTTGGCCCATGATGGCTGAACAGCATCTAAATGCGCGAATGGTGATCGAGGAGATTAAGGTCGGGTTACGAGTCGAAACGTGTGATGGTTCGCTTAGAGGGTTTGTGAAACCAGATGGGTTGAAGAAGATGGTTAAGGAGTTGATGGAAGGTGAGAAGGGGAAAGAGGTTAGGAAGACGGTTAAGGCGGTTGGTGCAGCCGCGAAGCGAGCCATGGCGCATGGTGGTTCGTCTTGGCGGTCATTGAACGAGCTCATAAACGAGTTGCAGACTGTTAGAGTCTGTGGTAGTTAA

MASSLPVPTVGSTHRPHIVLFPFMSKGHTIPLLQLARLLVNRGSMVTVFTTKANHPFVAQFLEPYPIGSISIIDLKFPNHVEGLPEGVESTDKLPSIKLFRQFAVATKLMQPQFEQSLEKLSNVTCILSDGFLSWTLASANKFGIPRMTFYGMNAYSSAVSKDVSLSLWGPESDDELITAPGFPWIKVTRNDFDEPFNKRDSTSPLFEFVMEAVIASANSYGLILNSFYELEPLFVDYLNRESKPKAWCLGPLCLVDTLDSADNKQTNVPSNIISTSPDQKPKVPEWIEWLDQKLAKGSSVLYVAFGSQAEISIQQLEAISKGLEQSEVNFLWVVRKCDQTNVLDELEQRVGERGMIVNQWVDQRDILNHESVNGFVSHCGWNSALESICSGVPILAWPMMAEQHLNARMVIEEIKVGLRVETCDGSLRGFVKPDGLKKMVKELMEGEKGKEVRKTVKAVGAAAKRAMAHGGSSWRSLNELINELQTVRVCGS

SrUGT90B2

ATGGCTTCATCACTTCCGGTTCCGGCGGTTGGTTCGACCCACCGACCTCACATAGTCTTGTTTCCGTTCATGTCTAAAGGTCACACCATCCCACTACTCCAACTAGCCCGGTTGCTCGTGAACCGCGGGACCATGGTCACTGTTTTCACCACCAAAGCTAACCACCCATTTGTGGCTCAATTTCTTGAAACCTACCCAAACGGTTCCATCTCGATCATAGACCTTATGTTCCCTAACGATGTCGAGGGTGTGCCCAAAGGCGTCGAGAGCACCGATAAGCTACCTTCGATCAAGCTTTTTAGACAATTTGCGGTTGCAACCAAACTCATGCAGCCTCAATTTGAACAATCACTCGACAAACTATGGAATGTTACGTGCATCGTTTCCGATGGTTTTTTGAGTTGGACGCTTGCATCGGCAAACAAGTTTGGGATCCCACGGATGACGTTTTACGGGATGAACGCTTATTCGAGTGTTGTGTCTAAAGAAGTCGGTTTAAGCCTCTGGGGACCCGAGTCGGATGATGAACTAATAACCGTCCCCGGGTTCCCGTGGATCAAAGTCACTAGAAATGACTTTGACGAGCCGTTTAATCAGCGCGATTCAACGAGTCCTCTTTTTGAATTCGTAATGGAGGCGGTAATAGCCTCGGCTAACAGTTATGGTCTGATCTTAAACAGCTTCTACGAGCTAGAGCCATTGTTCGTAGACTACTTAAACCGCGAGTCCAAACCCAAAGCTTGGTGTGTCGGACCACTTTGTCTGGTTGACACACCAGACAGTGCAGATAACAAACAAACAAACGTACCCAGTAATATTATTAGTACCTCACCGGATGAGAAACCGAAAGTCCCGGAATGGATAGAGTGGCTAGACCAAAAGCTAGCTAAAGGAAGCTCGGTTTTATACGTCGCGTTCGGTTCACAAGCCGAGATCTCAATCCAACAATTGGAAGCCATATCCAAAGGTTTGGAACAATCAGAAGTGAACTTCTTGTGGGTGGTGAGAAAGTGTGATCAAACCAATGTTCTCGACGAGCTTGAACAGCGAGTAGGCGAAAGAGGGATGATAGTTAACCAATGGGTGAACCAAAGGGAGATTTTGAACCACAAGAGCGTGAACGGGTTCGTGAGTCACTGTGGTTGGAACTCGGTTCTAGAGAGCATATGTTCCGGGGTACCGATCCTAGCTTGGCCCATGATGGCTGAACAGCATCTGAATGCGCGAATGGTGGTTGAGGAGATTAAGGTCGGGTTACGAGTCGAAACGTGTGATGGTTCGGTTAGAGGGTATGTGAAACCAGATGGGTTGAAGAAGATGGTTAAGGAGTTGATGGAAGGTGAGAAGGGGAAAGAGGTTAGGAAGACGGTTAAGGCAGTTGGTGCAGCCGCGAAGCGAGCCATGGCGGATGGTGGTTCGTCTTGGCGGACGTTGAACGAGCTCATAAACGAGTTGCAGACTGTTAGAAACTGTGGTAGTTAA

MASSLPVPAVGSTHRPHIVLFPFMSKGHTIPLLQLARLLVNRGTMVTVFTTKANHPFVAQFLETYPNGSISIIDLMFPNDVEGVPKGVESTDKLPSIKLFRQFAVATKLMQPQFEQSLDKLWNVTCIVSDGFLSWTLASANKFGIPRMTFYGMNAYSSVVSKEVGLSLWGPESDDELITVPGFPWIKVTRNDFDEPFNQRDSTSPLFEFVMEAVIASANSYGLILNSFYELEPLFVDYLNRESKPKAWCVGPLCLVDTPDSADNKQTNVPSNIISTSPDEKPKVPEWIEWLDQKLAKGSSVLYVAFGSQAEISIQQLEAISKGLEQSEVNFLWVVRKCDQTNVLDELEQRVGERGMIVNQWVNQREILNHKSVNGFVSHCGWNSVLESICSGVPILAWPMMAEQHLNARMVVEEIKVGLRVETCDGSVRGYVKPDGLKKMVKELMEGEKGKEVRKTVKAVGAAAKRAMADGGSSWRTLNELINELQTVRNCGS

SrUGT91C1

ATGGATCAAGAACCACTTCACATCGCGATGTTTCCATGGTTAGCCATGGGTCATCTCATACCCTTTTTTCACTTATCAAAAATCTTAGCTCACAAAGGCCATCAAATCTCATACATTTCCACTTCAAGAAATCTAGAACGAATTCCCAAAATACCCCCCAAACTATCCCATAATATCAAACTTATCTCACTCCCATTTCCAAAAGTGGAAAATTTACCAGAACTTGCAGAGTCTTCCATGGATATCCCTTACCAAAAATCCCAATTTCTAAAAATAGCATTTGACTTACTTGAACCATCTATTTTGACTTTTCTTCAAACCTCAAAACCAAGAGTTGACTTTATCATCTTTGACTATGCTTCCCATTGGCTACCTTCACTTGCTTCAAAATTTAATATCTCAACTGCTTACTTTAGCCTTTTTACTGCTGCAACACAAGGTTTTCTTGGTCCTCCATCTTTATTGTCAAATACCGCGCTTATGAGGTCAAATCCTGAGGATTTTTGTCGGGTGCCGGAGTGGGTCCCGTTTGATTCAGATGTCAGGTATCGTATCCACGAGTTAACAAAGTATACTGAGGGTGCGGTTGGGAACGAGTCAGGCGTATTGGATACCGTAAGGTTTCTAGTGTCGATCGACGGTTGTGATTTGGTCTTGTTCAGAACAAGTTATGAGTTTGAACCCGAGTGGTTCGATCTTGTTTGTAAGCTTTACCAAAAACCCGTTATCCCAATTGGCGTTTTGCCTCCATCAATAGAAGATAATGAGTTTGATGATTATGAAAATTGGGATTCTATTAAAAACTGGCTAGATAAACAACCGGTTGACTCGGTTGTTTTTGTTGCACTTGGAAGTGAGGCGGTTTTGAGTCAAGTTGAACTCAGTGAGTTGGCTTTAGGGTTAGAGCGTTCGGGTTTGCCTTTCTTTTGGGTAATACGAAAGTCGATCCTCGAGTCAAATCAGCTTCCTGACGGTTTTCTTGAGAGGATCAAGGGTCGGGGAGTGGTTAACATCGGGTGGGTCCCACAAGTGAGGATACTGAGTCACTCATCAATAGGTGGATTCTTGACTCACTGTGGTTGGAACTCAGCTATTGAAGGACTCTCTTTTGGGCGAGTTTTGATATTTTTTCCGGTTATGAACGACCAAGGTTTGAATGTGAGATTATTAAGTGGAAAGAAACTTGGAGTCGAGATTCCT

CGAAATGAGATAGACGGGTCTTTTACGAGTGACTCGGTTGCTGAGTCAATTCAGTTGGCAATAGTGAGTGAAGAAGGAAAGGAATTGAGGGCAAATGCAAGAGAGATGAAAGAAGTATTTGGAGATAAGAGTAAGAATGATCACTATATTGATGATTGTGTAAATTACTTGGTGAAAATGAGGAAACCATAG

MDQEPLHIAMFPWLAMGHLIPFFHLSKILAHKGHQISYISTSRNLERIPKIPPKLSHNIKLISLPFPKVENLPELAESSMDIPYQKSQFLKIAFDLLEPSILTFLQTSKPRVDFIIFDYASHWLPSLASKFNISTAYFSLFTAATQGFLGPPSLLSNTALMRSNPEDFCRVPEWVPFDSDVRYRIHELTKYTEGAVGNESGVLDTVRFLVSIDGCDLVLFRTSYEFEPEWFDLVCKLYQKPVIPIGVLPPSIEDNEFDDYENWDSIKNWLDKQPVDSVVFVALGSEAVLSQVELSELALGLERSGLPFFWVIRKSILESNQLPDGFLERIKGRGVVNIGWVPQVRILSHSSIGGFLTHCGWNSAIEGLSFGRVLIFFPVMNDQGLNVRLLSGKKLGVEIPRNEIDGSFTSDSVAESIQLAIVSEEGKELRANAREMKEVFGDKSKNDHYIDDCVNYLVKMRKP

SrUGT91D1-2

ATGTTCCCATGGCTTGCTTTCGGTCACATTCTCCCATTCTTCGAACTCTCCAAATTCATAACCAAAAACGGTCACAAAGTTTCTTTTCTTTCCCCCACCGGAAACATACAACGTCTCCCTTCTTCTAATCTTTCGCCACTCATGAATCTTGTTAAACTCACACTTCCACGTGTTCAAGAGCTGCCCCAAAATGCATCCGCCACCACGGACCTCCATGCTGATGATGTCCAATATCTCAAACAAGCTTTTGATGGTCTTCAACCGGAGGTCACTCAATTTCTTGAACAAGAGTCGCCGGACTGGATTATTTATGATTTTGCTCCCTATTGGTTGCCGGCGGTAGCCACTAGCCTTGGAATTTCACGAGGTTTTTTCTCAATCTATAACGCATGGACCGTCTCTTTTATCGGATCGTCTTCCGATAACATGATAAACGGTACAGACAATCGGAAAACACCCGACGATTTCTTGACACCGCCGAAGTGGGTTCCGTTTCCAAGCAAAGTATGCTACCGGAAGCATGAGGCCAATTCGATATTTGCTGATAATTTTTCTATTAATTCTTCTGGGGTTTCAGATATATATCGTGCTGGAATGGTTATAAAGGGATCAGATTGTATGTTTATAAGACATTGCCATGAGTTCGAACCCCAATGGCTAACCCTTTTAGAAAAGCTGCATCACCTACCAGTGGTTCCCGTGGGATTATTGCCACCCGAACCACCCACCAGCACCGGAGACCCATGGGTGACAATCAAGAAGTGGCTCGATGGTCAACCAATAGGGCATGTGGTGTACGTGGCATTTGGAAGTGAAGGTACGATGAGCCAAAGCGAGCTGGCTGAGTTAGCTCTGGGCCTCGAGCTCTCCGGGTTGCCATTCTTTTGGGTTCTTAGAAAACCGGTTGGCTCTGGTAACTCGGTGGAGTTGCCAGAGGGGTTCTTGGAACGAACTCGTGACCGTGGGTTGGTGTGGACGAGTTGGGTACCTCAGTTACAAATACTGAGCCATGAGTCAGTGTGTGGTTTCTTGACTCATTCTGGTTGGAGTTCATTTGTGGAAGCGATGATGTTCGGTCACCCTTTGATAATGCTACCGCTTTCGGTGGATCAAGGTCTAAATGCTCGAGTAATGGCGGATAATCAGGTGGGAATTGAGATACCAAGAAATGACGAAGATGGTTCATTCACCAAGGAGTCGGTGGCCAGATCATTGAGGTTGGTTTTAGTCGATGATGAAGGGAAGATCTACAAGGCGAAGGCGATGGAGTTGAGTCAACGATTCGGGGATAGTAAGCCGGAAAATAAGTATATAAACCCTTTTATAGACTATTTGGAACAAAAAGGTCGTGTGGTTGCTATTGAGCATGAGCTTTAA

MFPWLAFGHILPFFELSKFITKNGHKVSFLSPTGNIQRLPSSNLSPLMNLVKLTLPRVQELPQNASATTDLHADDVQYLKQAFDGLQPEVTRFLEQESPDWIIYDFAPYWLPAVATSLGISRGFFSIYNAWTVSFIGSSSDNMINGTDNRKTPDDFLTPPKWVPFPSKVCYRKHEANSIFADNFSINSSGVSDIYRAGMVIKGSDCMFIRHCHEFEPQWLTLLEKLHHLPVVPVGLLPPEPPTSTGDPWVTIKKWLDGQPIGHVVYVAFGSEGTMSQSELAELALGLELSGLPFFWVLRKPVGSGNSVELPEGFLERTRDRGLVWTSWVPQLQILSHESVCGFLTHSGWSSFVEAMMFGHPLIMLPLSVDQGLNARVMADNQVGIEIPRNDEDGSFTKESVARSLRLVLVDDEGKIYKAKAMELSQRFGDSKPENKYINPFIDYLEQKGRVVAIEHEL

SrUGT91D1-4

ATGGACGACCATAAGCAGCTTCATGTTGCGATGTTTCCCATGGCTTGCTTTTCGGTCCACATTCTCCCATTCTTCGAACTCTCCAAATTCATAACCAAAAACGGTCACAAAGTTTCTTTTCTTTCCCCCACCGGAAACATACAACGTCTCCCTTCTTCTAATCTTTCGCCACTCATGAATCTTGTTAAACTCACACTTCCACGTGTTCAAGAGCTGCCCCAAAATGCATCCGCCACCACGGACCTCCATGCTGATGATGTCCAATATCTCAAACAAGCTTTTGATGGTCTTCAACCGGAGGTCACTCGATTTCTTGAACAAGAGTCGCCGGACTGGATTATTTATGATTTTACTCCCTATTGGTTGCCGGCGGTAGCCACTAGCCTTGGAATTTCACGAGGTTTTTTCTCAATTTTTAACGCATGGACCGTCTCTTTTTTCGGATCTTCTCCCGATGACATCATAAACGGTACAGACGATCGGAAAACAGCCGACGATTTCCTGACACCGCCGAAGTGGTTTCCGTTCCCAAGTAAAGTATGCTACCGGAAGCATGAGGCCAATTTGATATTTGCTGATAATATTTCTGTTAATTCTTCTGGTGTATCAGATTTATATCGTTTAGGAATGGTTATAAAGGGATCAGATTGTATGTTTATAAGACATTGCCATGAGTTCGAACCCCAATGGCTAACCCTTTTAGAAAAGCTGCATCAGCTACCGGTGGTTCCCGTGGGATTATTGCCACCCGAACCACCCACCAGCACCGGAGACCCATGGGTGACAATCAAGAAGTGGCTCGATGGTCAACCAATAGGGCATGTGGTGTACGTGGCATTTGGAAGTGAAGGTACGATGAGCCAAAGCGAGCTGGCTGAGTTAGCTCTGGGTCTCGAGCTCTCTGGGTTGCCATTCTTTTGGGTTCTTAGAAAACCGGTTGGCTCTGGTAACTCGGTGGAGTTGCCAGAGGGGTTCTTGGAACGAACTCGTGACCGTGGGTTGGTGTGGACGAGTTGGGTACCTCAGTTACAAATACTGAGCCATGAGTCGGTGTGTGGTTTCTTGACTCATTCTGGTTGGAGTTCATTTGTGGAAGCGATGATGTTCGGTCACCCTTTGATAATGCTACCGCTTTCGGTGGATCAAGGTCTAAATGCTCGAGTAATGGCGGATAATCAGGTGGGAATTGAGATACCAAGAAATGACGAAGATGGTTCATTCACCAAGGAGTCGGTGGCCAGATCATTGAGGTTGGTTTTAGTCGATGATGAAGGGAAGATCTACAAGGCGAAGGCGATGGAGTTGAGTCAACGATTCGGGGATAGTAAGCCGGAAAATAAGTATATAAACCCTTTTATAGACTATTTGGAACAAAAAGGTCGTGTGGTTGCTATTGAGCATGAGCTTTGA

MDDHKQLHVAMFPMACFSVHILPFFELSKFITKNGHKVSFLSPTGNIQRLPSSNLSPLMNLVKLTLPRVQELPQNASATTDLHADDVQYLKQAFDGLQPEVTRFLEQESPDWIIYDFTPYWLPAVATSLGISRGFFSIFNAWTVSFFGSSPDDIINGTDDRKTADDFLTPPKWFPFPSKVCYRKHEANLIFADNISVNSSGVSDLYRLGMVIKGSDCMFIRHCHEFEPQWLTLLEKLHQLPVVPVGLLPPEPPTSTGDPWVTIKKWLDGQPIGHVVYVAFGSEGTMSQSELAELALGLELSGLPFFWVLRKPVGSGNSVELPEGFLERTRDRGLVWTSWVPQLQILSHESVCGFLTHSGWSSFVEAMMFGHPLIMLPLSVDQGLNARVMADNQVGIEIPRNDEDGSFTKESVARSLRLVLVDDEGKIYKAKAMELSQRFGDSKPENKYINPFIDYLEQKGRVVAIEHEL

SrUGT92B1

ATGGCGGATAACAACATCATCATGTTCCCTTTCATGGCACAAGGCCATATCATCCCTTTTCTATCTTTATCCCTCCAATTGGAACAAAAGGGCTACCAGATCACCTTCATCAACACCCCTCTCAATATCGCCAATCTCCGCCGTGCTCTTCCGCCGTCCTCCGCCATCCGCCTCTTTGAAATCCCCTTCAACCCCTCCGACTTCGGTCTCCCTGCTAACGCCGAGAACACCGATTCACTCTCTCCGAACAAAATTTACGAACTCCTCGTCGCCTCCACCTCACTCAAACCCGCATTCAGAAACATAATCAACGACCTCGTTTCCGACGGAAGCTCCGCCGCTGTGTGTGGTGGCGGACTTTTCTTCGGGTGGTCGGCGGCCGTGTCGCATGAATTCGGAATCTTTCATGTGATCTTCAGTGGATCCGGTGGGTTTGGATTAGCGTGTTACTTCTCTTCTTGGATCCATTTACCTCACAAAAATTCCGATTGCGGTGAGTTCACGTTGCCGGATTTTCCAGAAGCCGGTAAATTACAGGTGACCCAGTTGACCCCAAGTCTTATAGCAGCGACCGGCGAAGACCCTTGGTCGGAATTCCAAAAAAAGAATCTTCCGGCATGGGGCGATGCAGATGGGTTCTTGTTCAACACAATTGAAAAGCTAGATAAAGTTGGTCTATCGTATTTCAGAAAAAAATTCCGGCGACCGGTGTGGGCAATTGGGCCGATAAACTTATCATTAACTGGCGGAGCTCCTACCAAGAAAAACTATTCCGGCAAAGAAGAATTGGTTCAATTTCTGAACTCAAAACCAGCATCCTCTGTTTTGTACATTTCATTCGGATCACAAAACACAATAACGGCTTCCCAGATGATGCAATTGGCAAAGGCATTAGATAAAAGCAACAAAAGCTTCATCTGGGTTGTCCGGCCGCCGTTAGGATTCGACATAAACGCCGAGTTCCGAGCAGATGAATGGTTGCCGGAAAACTTCACAACACGAATTAAAGACGAAAACAGAGGTCTAATCATCGAAAAATGGGCACCACAGCTTGAAATACTGTCACATGAATCTGTAGGTGGGTTTATGACTCACTGTGGGTGGAATTCGGTGCTTGAATCGGTCAGCCGTGGTGTGCCGATGCTCGGGTGGCCGATGGCGGCGGAGCAGTTTTATAACGCGAAGATGATGGTGGAGATTGCAGAGGTGTGCGTGGAGGTGGCGCGTGGGACTAATTTTGATGTCCGTTTTGAGGATTTGAGGGAGAAGATAGAGGAAGTGATGAGTGAAGATGGAAGAGGGAAAGAGATGAGGAAGAAAGCGTTTGAGATTAAGATGATGATTGAAGAAGCAACGAGAGATGAAGAAGGTTTTAAAGGTTCTTCTGTGGAAGCCATGGAAGAGTTCCTTCAAGCTCCATTGAAGAAGATGAACAATAACACCACCAATGCTTGA

MADNNIIMFPFMAQGHIIPFLSLSLQLEQKGYQITFINTPLNIANLRRALPPSSAIRLFEIPFNPSDFGLPANAENTDSLSPNKIYELLVASTSLKPAFRNIINDLVSDGSSAAVCGGGLFFGWSAAVSHEFGIFHVIFSGSGGFGLACYFSSWIHLPHKNSDCGEFTLPDFPEAGKLQVTQLTPSLIAATGEDPWSEFQKKNLPAWGDADGFLFNTIEKLDKVGLSYFRKKFRRPVWAIGPINLSLTGGAPTKKNYSGKEELVQFLNSKPASSVLYISFGSQNTITASQMMQLAKALDKSNKSFIWVVRPPLGFDINAEFRADEWLPENFTTRIKDENRGLIIEKWAPQLEILSHESVGGFMTHCGWNSVLESVSRGVPMLGWPMAAEQFYNAKMMVEIAEVCVEVARGTNFDVRFEDLREKIEEVMSEDGRGKEMRKKAFEIKMMIEEATRDEEGFKGSSVEAMEEFLQAPLKKMNNNTTNA

SrUGT94A1-1

ATGAATCAAGAATCACTTCCACCTCATGTTCTAATATTTCCATTGCCATTACAAGGTCCAGTGAATTCCATGCTCAAGTTAGCCGAGCTTCTATGTCTATCTGGTCTCCACATCACCTTTCTTGTTACTGATCATATCCACAATCGACTAATCAAATATTCCAATATTGAATCGCGCTTTAATGGTTACCCTGGTTTTCGTCTTGAAACCATATCTGATGGTCTTCCAGAATACCATCCTCGATCTGGACCTGTGCTTATGGAAATGTTTGACTCTTTAAAAGTCAAAACCAAGATTCTTCTTAAAGATCTTTTGACATCTGGTAAACTGAATTCAGATTCACGGAGACCTGTAACTTGTATAATTGGAGATGGAATAATGGGGTTTACATGTGATGTTGCTAATGATGTTGGATTACCGATTATATATGTTCGTACAATCAGTGCTTGTTGCTTGTGGGTTTTCTTTTGTCTTCCGAAGCTCATTGAATCGGGTGAAATCCCATTTGCAGGTAACGACTTGGATACACCAATAAAGAGTATACCGGGAATGGAAGGGTTTTTTAGGCGTCGTGATCTTCCAATGTTTTGTCGTTCTGGTAGTTTGTCAGACCCCAACTTGAACCTCTATCTACCTGAAATTACCGAAAACTCAAGAGCCCATGGGCTTATACTCAACACATTTGACGATTTAGAGGGCCCAATACTAGCCCAAATACGCGCATTTTGTCCAAACCTATACACGATCGGCCCACTTCATTCTCATCTTAAATACAAGCTATTAGATCAATCATCGTCATCACCACCTTCTTCAAATAGTCTATGGAAAGAAGATATGAGTTGCATCGCATGGTTGGACTCACAACCACCAAAATCGGTTATTTATGTTAGTTTCGGGAGTCTTGCAGTAATGACTAAGGATCAATACATGGAGTTTTGGCATGGGTTGGTGAATAGTGGGTCCCGTTTCTTGTGGGTCATACGGCCTGACTCAGTTACCAGTGATCCAACTGACGTTTCGTCAGAACTTTTGAAAGGTACAAAGGAGAGAGGGTACATAGTCGAATGGGCCCCACAAGAGGAGGTATTGGCCCATCGGGCCGTGGGTGGGTTTTTAACGCACAGTGGATGGAATTCGACTTTGGAGAGTGTGATTGAAGGTGTGCCGATGATCTGTTGGCCGTATTTTTTAGACCAACAAGTGAATAGTAGGTATGTGGGGGAAGTGTGGAAGTTGGGGTTGGATATGAAAGATACTTGTGATAGAGTGATTGTAGAGAAAACTGTGAGAGATTTGATGGAAGATAGGAAAGATGAGTTTAGAAAATCGGCGGATGAGATGGCGAATTATGCAAAAGAATGCTTGATGAAAGGTGGATCATCTTATTGTAATTTAGAGCGTTTGGTGAAGGATATCAAAGCTATGTAA

MNQESLPPHVLIFPLPLQGPVNSMLKLAELLCLSGLHITFLVTDHIHNRLIKYSNIESRFNGYPGFRLETISDGLPEYHPRSGPVLMEMFDSLKVKTKILLKDLLTSGKLNSDSRRPVTCIIGDGIMGFTCDVANDVGLPIIYVRTISACCLWVFFCLPKLIESGEIPFAGNDLDTPIKSIPGMEGFFRRRDLPMFCRSGSLSDPNLNLYLPEITENSRAHGLILNTFDDLEGPILAQIRAFCPNLYTIGPLHSHLKYKLLDQSSSSPPSSNSLWKEDMSCIAWLDSQPPKSVIYVSFGSLAVMTKDQYMEFWHGLVNSGSRFLWVIRPDSVTSDPTDVSSELLKGTKERGYIVEWAPQEEVLAHRAVGGFLTHSGWNSTLESVIEGVPMICWPYFLDQQVNSRYVGEVWKLGLDMKDTCDRVIVEKTVRDLMEDRKDEFRKSADEMANYAKECLMKGGSSYCNLERLVKDIKAM

SrUGT94A1-2

ATGAATCAAGAATCACTTCCACCTCATGTTCTAATATTTCCATTGCCATTACAAGGTCCAGTGAATTCCATGCTCAAGTTAGCCGAGCTTCTATGTCTATCTAGTCTCCACATCACCTTTCTTGTTACTGATCATATCCACAATCGACTAATCAAATATTCCAATATTGAATCGCGCTTTAATGGTTACCCTGGTTTTCGTCTTGAAACCATATCTGATGGTCTTCCAGAAAACCATCCTCGATCTGGACCTGTGCTTATGGAAATGTTTGACTCTTTAAAAGTCAAAACCAAGATTCTTCTTAAAGATCTTTTGACATCTGGTAAACTGAATTCAGATTCACGGAGACCTGTAACTTGTATAATTGGAGATGGAATAATGGGGTTTACATGTGATGTTGCTAATGATGTTGGATTACCGATTATATATGTTCGTACAATCAGTGCTTGTTGCTTGTGGGTTTTCTTTTGTCTTCCGAAGCTCATTGAATCGGGTGAAATCCCATTTGCAGGTAACGACTTGGATACACCAATAAAGAGTATACCGGGAATGGAAGGGTTTTTTAGGCGTCGTGATCTTCCAATGTTTTGTCGTTCTGGTAGTTTGTCAGACCCCAACTTGAACCTCTATCTACCTGAAATTACCGAAAACTCAAGAGCCCATGGGCTTATACTCAACACATTTGACGATTTAGAGGGCCCAATACTAGCCCAAATAAGCGCATTTTGTCCAAACCTATACACGATCGGCCCACTTCATTCTCATCTTAAATACAAGCTATTAGATCAATCATCGTCATCACCACCTTCTTCAAATAGTCTATGGAAAGAAGATATGAGTTGCATCGCATGGTTGGACTCACAACCACCAAAATCGGTTATTTATGTTAGTTTCGGGAGTCTTGCGGTGATGACAAAGGATCAATACATGGAGTTTTGGCATGGGTTGGTGAATAGTGGGTCCCGTTTCTTGTGGGTCATACGGCCTGACTCAGTTACCAGTGATCCAACTGACGTTTCGTCAGAACTTTTGAAAGGTACAAAGGAGAGAGGGTACATAGTCGAATGGGCCCCACAAGAGGAGGTATTGGCCCATTGGGCCGTGGGTGGGTTTTTAACGCACAGTGGATGGAATTCGACGTTGGAGAGTGTGATTGAAGGTGTGCCGATGATCTGTTGGCCGTATTTTTTAGACCAACAAGTGAATAGTAGGTATGTGGGGGAAGTGTGGAAGTTGGGGTTGGATATGAAAGATACTTGTGATAGAGTGATTGTAGAGAAAACTGTGAGAGATTTGATGGAAGATAGGAAAGATGAGTTTAGAAAATCGGCGGATGAGATGGCGAATTATGCAAAAGAATGCTTGATGAAAGGTGGATCATCTTATTGTAATTTAGAGCGTTTGGTTAAGGATATCAAAGCTATGTAA

MNQESLPPHVLIFPLPLQGPVNSMLKLAELLCLSSLHITFLVTDHIHNRLIKYSNIESRFNGYPGFRLETISDGLPENHPRSGPVLMEMFDSLKVKTKILLKDLLTSGKLNSDSRRPVTCIIGDGIMGFTCDVANDVGLPIIYVRTISACCLWVFFCLPKLIESGEIPFAGNDLDTPIKSIPGMEGFFRRRDLPMFCRSGSLSDPNLNLYLPEITENSRAHGLILNTFDDLEGPILAQISAFCPNLYTIGPLHSHLKYKLLDQSSSSPPSSNSLWKEDMSCIAWLDSQPPKSVIYVSFGSLAVMTKDQYMEFWHGLVNSGSRFLWVIRPDSVTSDPTDVSSELLKGTKERGYIVEWAPQEEVLAHWAVGGFLTHSGWNSTLESVIEGVPMICWPYFLDQQVNSRYVGEVWKLGLDMKDTCDRVIVEKTVRDLMEDRKDEFRKSADEMANYAKECLMKGGSSYCNLERLVKDIKAM

SrUGT94B1

ATGGATCTACAACCACCACATGTTCTTATCTTTCCGTTTCCGGCTCAAGGCCACGTCAACGCCATGTTAAAACTCACCGAGCTCCTTCTGCCAGTCGGTCTCCACATCACCTTCTTAATATCCTCCAAAGACTACCGCAGTCTCTGCAGTTATGCCACCATCCACTCGCGCCTTAACTCCAAACCAGGTTTCCGGTTCCATGTTATCGAGGGTCTCTATGAAGGAAATATGGACACCGGTGAAAAGATCAGTCTGATGGTTGATAGTTTGGCTCAAGTAGCAACACCGTTGCTCAGGAAATTGATTCTTGATTTTCCGGTGACTTGTGTTATTGACGATGGAATTAATGGGTTTTCACTTGATGCGGTTGAGGGTACCGGAATTCCGGTCATTTTTTTCCGGACGATTAGTGCTTGTGCTTTCTGGGCTTACTTTAGCATCCCGGAACTCGTAAGCTCCGGTGAACTTCCTTTTACAGGCACAAACATGGATGAACGAATAGTGAGTGTGAAAGGTATGGAAGGATTCCTTCGCCGGCGTGATCTTCCAAGCTTTTGCCGTTCCGACATAAGCAACTACACTTTCCAGCAGGTCAGCAGTAAAACACGTCAGACGGTAAATGCTCATGCACTCATTCTTAACACCTTTGATGACCTTGAAGGGCCTATTGTCTCACAGATTCAAAACCATTGCACCAATATCTACACAATCGGGCCACTTCATGCACATCTAAAATCTCGATCCTTGTCTGTATCCACATCCTCAAATAGTTTTTTCAAGGAAGACAAGACTTGCATAGGCTGGCTTGATCAACAACCACCAAAATCTGTGTTGTACGTGAGTTTTGGAAGTTTAGCCACGTTAACAAGGGACCAACTTCTCGAGTTTTGGTATGGTTTAGTCAATAGCAATAAACTATTTTTGTGGGTCATTCGCGAGGATCTAGTTATGAGCAAAGATGATGAAAATAAAATACCATTTGAGGTAGAGAAAGGTACCAAAGAAAGAGGATATCTGGTTGGGTGGGCTCCACAAGAAGACGTGCTAGCCCACCCAGCCGTAGGTGCCTTTCTAACACATAATGGGTGGAACTCGACTTTGGAGAGCATTGTCGCAGGTGTGCCAATGGTGAGTTGGGCATTTTTTGCTGATCAACAAATAAATAGTAGATTTATCGATGCAGTTTGGAAACTAGGGCTAGACATGAAGGACACATGTGATCGAACCACGGTAGAGAAGATTGTAAATGAAGTAATGGAGATTTGGAAGGAGGAGTTTATAGAATCAGCCAATCATATGGCCAAATTAGCTATGAAGTCTGTGAACAAAGGTGGATCTTCATATAGTAATTTGGATCGTCTGATCGAAGACATTAAAAAAATGGGTCTTAACAACATAAATTAG

MDLQPPHVLIFPFPAQGHVNAMLKLTELLLPVGLHITFLISSKDYRSLCYATIHSRLNSKPGFRFHVIEGLYEGNMDTGEKISLMVDSLAQVATPLLRKLILDFPVTCVIDDGINGFSLDAVEGTGIPVIFFRTISACAFWAYFSIPELVSSGELPFTGTNMDERIVSVKGMEGFLRRRDLPSFCRSDISNYTFQQVSSKTRQTVNAHALILNTFDDLEGPIVSQIQNHCTNIYTIGPLHAHLKSRSLSVSTSSNSFFKEDKTCIGWLDQQPPKSVLYVSFGSLATLTRDQLLEFWYGLVNSNKLFLWVIREDLVMSKDDENKIPFEVEKGTKERGYLVGWAPQEDVLAHPAVGAFLTHNGWNSTLESIVAGVPMVSWAFFADQQINSRFIDAVWKLGLDMKDTCDRTTVEKIVNEVMEIWKEEFIESANHMAKLAMKSVNKGGSSYSNLDRLIEDIKKMGLNNIN

SrUGT94C1

ATGGCTCAAACTCACCAACAACCAAACAACCCTAAAACCAAGCTCGAACCACATGTGCTTATATTTCCCATACCATTTCAAGGTCCAGTCAATTGTGCACTCAAGTTAGCCGAGCTCCTATGTCTTTCCGGCATCCACGTCACCTTCCTCAACACCGAACACATCCACCGACCCCTCATCCACCACACCAACGTCCTCTCCCGGTTCAACCGCTACCCGAACTTCCGGTTTGAGACTCTCCCTGATGGTCTCCAGCATGAAAAACCGGTTTCTGGAGACGGGTTTATGGAGGTTATGGAGGCTGTAGATGCGGTGAGTAAACCTCTTTTCCGGGAGATGATGGTTTCCGGTAAGATGAGTCGGAGATCGGAGCGGCCGGTGACGGTGATGATACCGGATGCATGTTTTAGTTTTGCGGTGGATATAGCGACGGAGGCATCGATTCCGGTGATATGTTTCGAGACTGTAAGTCCATGTTGTTTGTGGACTTCATATTTGAATCTTCCAACGCTTATTGAAGCAGGAGATGTCCCCTTTAAAGGAGATGATCTAGACCAATTCATAACAAGTGTGCCAGGAACAGAGCATATCATCCGACGCCGCGATCTTGCTAGCTTTTGTCGAACCGACGACTTGTCGATCCCCGTAATAGATCTCATCGTCAAAGAAGCTCACACGGTTCCTCGAGCTCAAGGTCTCATACTCAACACGTTTGAAGAGTTGGATGCTCTCATACTCTCTCACATGCGGAAGCTTTGTCCAAATATTTACACCATAGGCCCGCTTCATACGTTCCACAAAGCTCAACTCATGGCTAACACAACAAAACCATCGCAAGAAACCACATTTTCCAATAGTGTTTGGAAGGAAGATAGATCTTGCATGGCGTGGCTCGATGAGCACGAACCACAAACCGTCATTTATGTTAGCATAGGGAGTCTTGCGACCATGACGGTTGAACAACTTATTGAGATATGGTATGGTGTGGTTAATAGTGGGAAACCGTTTTTGTGGGTGAGACGACCTGGTTCGATCACCGGTGGGTATGATGAGTCTTTGGTTCCCCCAGAGCTACTAGCGCGTACGAGGGAGATAGGGTGTATAGTAGAATGGGCTCCACAAGAAGATGTACTCGCCCATCCAGCCATTGGCGGGTTTTTGACTCATAGTGGATGGAACTCGACTATGGAGAGTATAGCCGCGGGTGTCCCCATGGTTTGTTGGCCATATTTTGTGGACCAACAAGTGAATAGTCGATTTGTGGGCGAGGTATGGAAAATTGGAGTAGACATGAAAGATACATGTGATCGGTTGATTGTTGAAAAGGCAGTGAGGGATATTATGGACTTGAAACCGAATGAGTTTACCCAATCTGCCAATGCTTGGGAAAATTCGGCTAAAGAATCGATCACGAAGACCGGTTCATCGTCTATAAGTTTGAGTCGGTTGATTGATGATATTTTAGCAATGAGCTCGTCCCAATCCTGA

MAQTHQQPNNPKTKLEPHVLIFPIPFQGPVNCALKLAELLCLSGIHVTFLNTEHIHRPLIHHTNVLSRFNRYPNFRFETLPDGLQHEKPVSGDGFMEVMEAVDAVSKPLFREMMVSGKMSRRSERPVTVMIPDACFSFAVDIATEASIPVICFETVSPCCLWTSYLNLPTLIEAGDVPFKGDDLDQFITSVPGTEHIIRRRDLASFCRTDDLSIPVIDLIVKEAHTVPRAQGLILNTFEELDALILSHMRKLCPNIYTIGPLHTFHKAQLMANTTKPSQETTFSNSVWKEDRSCMAWLDEHEPQTVIYVSIGSLATMTVEQLIEIWYGVVNSGKPFLWVRRPGSITGGYDESLVPPELLARTREIGCIVEWAPQEDVLAHPAIGGFLTHSGWNSTMESIAAGVPMVCWPYFVDQQVNSRFVGEVWKIGVDMKDTCDRLIVEKAVRDIMDLKPNEFTQSANAWENSAKESITKTGSSSISLSRLIDDILAMSSSQS

SrUGT96A1

ATGGATCAAAACCAACAAACATGTGTCGTGATGGTTCCCTTTATAGCACACGGCCATCTCAACCAGCTCATCCACCTCTCCCACCTAATCTCCACCTACGACCTACCGGTCCACTTTGTGTGCGCCTCAGGTCACACCCGCCAAGCCAAAATACGTGTCCATGGTTGGGACCCTCACTCGGACCCTAACATCCGGTTCCATGAGTTCCCAATACCATCTTTCCCTAACGATGCACCCAACCCTAATGGCCCAACACGCTTCCCTAACCACCTCATGCCGGCGTTCAAAGCCATCATGCACATCCAAGAACCCTTTGTTAAACTACTTTCCGATCTCTCGGTTACAATGAAAAGGGTTGTGATCATTCATGATTACTTGATGAGCTCCATTGTTCAAGACTTCGTTTCGTTCCCTAACGTTGAAGCTTACATGTTTCAAAGTTGCTCTGCTTTTACTGCCTTTTGGTTCCATTGGGAGGAAACACAAGCGTTAAAGTTGGACGATGAAGCCGAGTCGTTATGGGCAAAAATCCCACCGTTAGAAGGGTGTTTGAGTAGTGAGTTTGTTGAGTTACTTGACTCGGAAAACACGTCATTCAAGAAGATCAGCTCGGGAACACTTTATGACACGAGCAAAGTGTTTGAAGAGAAGTATTTAGAGTTACTTAAACATGAAGAGGTCACCTCTGGGACTGTCAAGAATTGGGCTATTGGCCCGTTTAACCCGGTTAGCATAACCGGTCATAAGAACTCGAGTACCGAATCGATCAAGCTATTCGATTGGTTAAACAAACAAGAACCAAATTCTGTTATTTATGTGTCGTTTGGTACCACGGTCACATTTACAAACGAAGAAATAAAAGAAATCGCAATCGGGTTGGAAGAAAGCGGTCAGAAGTTTATATGGGTGGTGAGGGAAGCGGATAAAGCTAACATTTTCGACGGAAAAGATGACCAGAGAGTTGCATTACCGGAGGGTTATGAAGAGAGGGTGGAAACCAGTGGGGTAGGGGTGGTGGTGAGGGGTTGGGCACCGCAGCTTGAGATACTGAACCACCAGTCTACAGGTGGGTTCATGAGTCACTGTGGTTGGAACTCATGCATGGAAGGGATCACAATGGGGGTTCCGATGGCTGCATGGCCCATGCATTCCGATCAACCGAGGAACGCCGTGTTGATTACCGAGGTGCTTGGAACCGCGATATACGCGAGAGAATGGGAGCGGCGAGACGAAAAGCTGGCGGCCGCGGTGGTTTCAGCGGCGGTTAGACGGTTGATGGCGTCCGAGGAGGGGTGTGTGGTGAGAAAGAAGGCGGAGGAACTTGGTGGTGCGGTCCGGCGATCGGTGGAAGAAGGTGGCGTTATGCGCAAAGAGTTGGATGAGTTTGTTGGTCATATCACCAGACAGTGA

MDQNQQTCVVMVPFIAHGHLNQLIHLSHLISTYDLPVHFVCASGHTRQAKIRVHGWDPHSDPNIRFHEFPIPSFPNDAPNPNGPTRFPNHLMPAFKAIMHIQEPFVKLLSDLSVTMKRVVIIHDYLMSSIVQDFVSFPNVEAYMFQSCSAFTAFWFHWEETQALKLDDEAESLWAKIPPLEGCLSSEFVELLDSENTSFKKISSGTLYDTSKVFEEKYLELLKHEEVTSGTVKNWAIGPFNPVSITGHKNSSTESIKLFDWLNKQEPNSVIYVSFGTTVTFTNEEIKEIAIGLEESGQKFIWVVREADKANIFDGKDDQRVALPEGYEERVETSGVGVVVRGWAPQLEILNHQSTGGFMSHCGWNSCMEGITMGVPMAAWPMHSDQPRNAVLITEVLGTAIYAREWERRDEKLAAAVVSAAVRRLMASEEGCVVRKKAEELGGAVRRSVEEGGVMRKELDEFVGHITRQ

SrUGT96B1

ATGACCGGCGACGACACCACTTTTCCGGCGACCGACGTGGTGGTAGTGACGGTGCCATTCGTGGCCCACGGCCACCTTACCCAACTTCTCCACCTCTCCCACCTCATCTCCGCCTACAACATCCCCGTAAACTTCATCACCACCACCGATTGTCTCCGTCAACTCCGCTCCCGCCACCGCACCTCCGCCGCCGGCATCGAATTTCACGACTTTCCGGTCCCGCCGTTCACCACCCCTCCACCGGACCCATCCAACCCGTTCCCTACCCACCTCCAACCGTCGTTCGACGCCACCCTCCATCTCCGGCAACCCACCGGCGACCTCATACGTTCACTGTCGCAAAAAGTCAAACGACGAGTTGTTGTAATTCACGACGTTCTCATGTCATACGTCGTTCAAGACGTGAAATCAATACCAAACGCTGAAACTTATGTTTTCCAGCCGCCACCGGCAATCTACAGAGCCGTCGGCCAGTGGGAGAGAACTGGCGGACCGTTACCGGTCGAACCGGAATTGATGAACCGGCTTCCTTCTCGAGACGGGACTTCAAGTCCAGAGTTTTTGGAGTTCTTGAAATATCAGAAATCTCACATGAATTTTCACGTCGGAGAATTATACGATTCGTCAAGAATAATCGAAGGCAAATTTATTGAATATCTAGAAAAACAAAATGTTAACGGGAAAATAAAGATATGGGCGGTCGGACCGGTTAACCCGGTCCAAATAGCATCTGATGTGACGGATCCAACAAACCGTCACAAATGCTTGAAATGGCTCGACAAGCAACCAGCAAACTCGGTCGTTTATGTGTCTTTCGGGACGACAACCACCTTTAGCGATGATCAAATTACCGAATTAGCCCTCGGGTTAGAGAGAAGTGGACAGAGGTTTGTTTGGGTGGTTCGAGTCGCTGACACGGTTGGTCCGTGTGGGTTCGAGGATAGGAAGGTTAAGTTGCCGGACGGGTTCGAGGAGAGGGTGAAAGAAAGGGGTTTGGTTGTGCGGTCGTGGGCCCCGCAGTCCGAGATTTTGGGACATTTTGCGACGGGCGGGTTTATGAGTCACTGTGGATGGAACTCAAGCATGGAGAGTATATCATTTGGGGTGCCAATTGTCGCGTGGCCGATGCACTCGGATCAACCGACGAACGCGTTTTTGGTGACGGATGTTCTTAAGATTGGGGTCGCAGTGAAGAGTTGGAATCGGAGAGATGAGTTGGTGACGGCGGATGTGGTGGCGGAGGTTGTTAGGATGCTGATGGGTTCGGATGAAGGGGAGGAGATGCGGCGGAGGGCGGTGAAATTGGGT

MTGDDTTFPATDVVVVTVPFVAHGHLTQLLHLSHLISAYNIPVNFITTTDCLRQLRSRHRTSAAGIEFHDFPVPPFTTPPPDPSNPFPTHLQPSFDATLHLRQPTGDLIRSLSQKVKRRVVVIHDVLMSYVVQDVKSIPNAETYVFQPPPAIYRAVGQWERTGGPLPVEPELMNRLPSRDGTSSPEFLEFLKYQKSHMNFHVGELYDSSRIIEGKFIEYLEKQNVNGKIKIWAVGPVNPVQIASDVTDPTNRHKCLKWLDKQPANSVVYVSFGTTTTFSDDQITELALGLERSGQRFVWVVRVADTVGPCGFEDRKVKLPDGFEERVKERGLVVRSWAPQSEILGHFATGGFMSHCGWNSSMESISFGVPIVAWPMHSDQPTNAFLVTDVLKIGVAVKSWNRRDELVTADVVAEVVRMLMGSDEGEEMRRRAVKLG
