# Supplementary material for: Characterizing glycosyltransferases by a combination of sequencing platforms applied to the leaf tissues of Stevia rebaudiana
Source: BMC Genomics. 2020 Nov 13;21:794. doi: 10.1186/s12864-020-07195-5 (PMC7664074; doi:10.1186/s12864-020-07195-5)
Supplement: Supplementary file 7 — Additional file 7.The nucleotide sequences of the five SrUGTs, which cloned and differed with transcriptome in this study. [file 12864_2020_7195_MOESM7_ESM.docx]

Additional file 7

The nucleotide sequences of the five *SrUGTs,* which cloned and differed with transcriptome in this study

SrUGT73G2

ATGGCTTTGGAATCAGTCAACCAGCTTCACTTCCTCCTGATCCCCTTTCTAGCTCCTGGTCACACCATCCCCATGATCCACATGGCCAAATTGCTCGCACAACGACCAAATGTGATGGTCACCATCGTCACCACACCCGTAAACGCGATTCGATATGGTTCTACGCTTCAAGAACACATCGAATCCGGACTCCCGGTGTGTTTTCTTGAACTTCCATTTCCGGCGACCGAGAATGGACTTCCGGAGGGATGCGAAAGTGTAGATGCTCTACATTGTCTAGAATTACTTCCAAACTTTTCGGCTGCAGTTGACACGTTACAAGAACGACTCGAGCAACGGTTCGAATCGATTAACCCACGTCCGAACTGTATTATATCTGATAAATACATGGTCTGGAGTGATTATACAGCAGCAAAGTATGAGATTCCCAGGATCATATTCGATGGGATGAGTTGTTTCAAACAACTATCTACACATCATTTGTATGCATCTAAGGTGTTCGATGATATGCCTGAGTCAGAACCATTTGTTCTCCCTGGATTGCCTGACAAGATCGAGATCACCCGAGCTCAACTGCCAGCAGAGTTCAATCATAGCCGTGGTGTAATGCGTGAGCAAATTGAACGAGTGAGAGAAACCGAGTCGAGAGCTTACGGAATGGTGATCAATAGTTTTGAGGAGTTGGAGCAAGAGTATGTTAAGGAACTTAAGAAGTTTAAAGGCGGTAAGATTTGGTGTTTAGGACCGTTATCATTATCTAACAATCATGATTCGAGTACATCTATTGATGATCAACGTTACTTGAAATGGCTCGATTCTAAAGAACCAGGATCGGTTGTGTATGCCTGCTTCGGTAGCAGTAGTCAAGTCACGCCCCCACAACTCATCGAGCTTGGGTTAGCATTGGAAGCATCCAAATCCCCGTTCATATGGGTGATTCGAGCCGGTGACAAAGTTAAAGAGGTCGAGAAATGGTTAATAGAAAGTGAGTTTGAAACTCGAGTAAAAGACAGAGGTCTTGTAGTCAGAGACTGGGCACCACAAATCTTAATCTTGTCCCACCGTTCGGTTGGAGGGTTCTTGACACACTGCGGTTGGAATTCAGTGTTGGAAGGGGTTTCGGCTGGTGTCCCTATGATCACGTGGCCTTTGTTTGCAGAACAATTCTTGAACGAGAAGTCGATAGTTCAAGTTTTGGGTGTCGGTGTTAGTGTTGGTGCTCCCGGTGTGGTGCACTGGGGCCAAGAAGACGAATTTGGGGTCACCGCGAAAAGCGAGCAGGTGAAAACAGCTATAGAAACAGTAATGGATGTCGGATCCGAAGGAAATGAGAGAAGAAAGAAGGCGAAATCACTTGCAATGGTAGCAAAAAAAAGCCATTGAAGAAGGGGGATCTTCTCACTATAACTTGATGTTACTAATACAAGATATATTGGAACATATAAATGCTAGAACCCATAAGCCAGAAACTACTTATTGTAGCTAA

SrUGT85C3-1

ATGGATGCAGTGGTCGAAACAGAAAAGAAGAAACCACACGTCATCTGTATGCCGTTTCCAGATCTAAGCCACATAAAAGCCATGCTCAAACTAGCCGAGCTTCTCTACCACAAAGGACTACAGATAACCTTCATCAACACCGATTTCGTCCACAACCGGTTTCTTGAATCAGGAGGACCACACTGTTTAGACGGTCCACCTGGTTTCCGGTTTGAAACCATTCCAGATGGTGTTACTCGCAGATCGGAAGCTAGCATCCACAACACCAGAGAACTACTCAAGTCCGTCGAAACCATCTTCTTGGATCGTTTCATTGACCTTGTAACCAAACTTCCGGATCCTCCGACTTGTATTATCTGTGATGGTTTCTTGTCGGTTTTCACAATTGACGCTGCAAAAAAGCTTGGAATCCCGATTATGATGTATTGGACACTTGCTGCCTGTGGCTTCATGGGGTTTTATCAAATGCATTCTCTCATTGAGAAAGGATTTGCACCGCTTAAAGATGAAAGTTATTTGACAAATGGGTATTTAGACACCGTCGTTGATTGGGTTCCGGGAATGGAAAGCATCCGTCTTAAGGAGTTCCCATTGGTTTGGAACACTGACTTGAATGATAAACTTCTAACATTCTGCAAGGAAGCTCCCCAAAGGTGTCACTCGGTTTCAAATCATATTTTCCACACGTTTGATGAGTTAGAGCCTAGTGTTATCAAAGCTTTGTCATCTATATATTCTCGCGTTTACGCCATCGGCCCACTACAACTACTTCTTGATCAGATACCTGAAGAAAAAAAGCATACTGGAATTTCAGGTTTGAATGGATACAGTTTTGTGAAAGAAGAACCCGAGTGTTTTAAGTGGCTTCAATCTAAGGAACCATATTCTGTCATTTATGTAAATTTTGGGAGTTCAACATTAATGTCTTTAGAAGACTTGATAGAATTTGGTTGGGGACTTGTTAATAGCAACCACTATTTCCTTTGGATCATCCGATCTAACTTGATAAATGGAGAATCTGCAGTTTTGCCTCTTGAATTAGACGAACATATAGAAAAGAGAGGCTTTATTGCAAGCTGGTGTTCACAAGAAAAGGTCTTGAACCACCCTTCCATTGGAGGGTTCTTGACTCATTGTGGGTGGGGATCCACAATCGAGAGCTTGTCAGCTGGGGTGCCAATGATATGTTGGCCTTTTTTTTGGGACCAACCGACCAACTGTAGGTATATATGCAATGAATGGGAAGTTGGACTTGAGATGGGGAATAAAGTGAAACGAGATGAAGTCAAGAGACTTGTACAAGAGTTAATGGGAGAAGGTGGTCACAAAATGAGAAACAAGGCCAAAGAATGGAAGGAAAAGGCTCGTATTGCCACATGCCCTAACGGTTCATCTTCTTTGAATGTGGATAAAATTGTCGAGGAGATAACGGTGCTATCAAGAGACTAG

SrUGT91D1-1

ATGTTCCCATGGCTTGCTTTCGGTCACATTCTCCCATTCTTCGAACTCTCCAAATTCATAACCAAAAACGGTCACAAAGTTTCTTTTCTTTCCCCCACCGGAAACATACAACGTCTCCCTTCTTCTAATCTTTCGCCACTCATGAATCTTGTTAAACTCACACTTCCACGTGTTCAAGAGCTGCCCCAAAATGCATCCGCCACCACGGACCTCCATGCTGATGATGTCCAATATCTCAAACAAGCTTTTGATGGTCTTCAACCGGAGGTCACTCAATTTCTTGAACAAGAGTCGCCGGACTGGATTATTTATGATTTTGCTCCCTATTGGTTGCCGGCGGTAGCCACTAGCCTTGGAATTTCACGAGGTTTTTTCTCAATCTATAACGCATGGACCGTCTCTTTTATCGGATCGTCTTCCGATAACATGATAAACGGTACAGACAATCGGAAAACACCCGACGATTTCTTGACACCGCCGAAGTGGGTTCCGTTTCCAAGCAAAGTATGCTACCGGAAGCATGAGGCCAATTCGATATTTGCTGATAATTTTTCTATTAATTCTTCTGGGGTTTCAGATATATATCGTGCTGGAATGGTTATAAAGGGATCAGATTGTATGTTTATAAGACATTGCCATGAGTTCGAACCCCAATGGCTAACCCTTTTAGAAAAGCTGCATCACCTACCAGTGGTTCCCGTGGGATTATTGCCACCCGAACCACCCACCAGCACCGGAGACCCATGGGTGACAATCAAGAAGTGGCTCGATGGTCAACCAATAGGGCATGTGGTGTACGTGGCATTTGGAAGTGAAGGTACGATGAGCCAAAGCGAGCTGGCTGAGTTAGCTCTGGGCCTCGAGCTCTCCGGGTTGCCATTCTCTTGGGTTCTTAGAAAACCGGTTGGCTCTGGTAACTCGGTGGAGTTGCCAGAGGGGTTCTTGGAACGAACTCGTGACCGTGGGTTGGTGTGGACGAGTTGGGTACCTCAGTTACAAATACTGAGCCATGAGTCAGTGTGTGGTTTCTTGACTCATTCTGGTTGGAGTTCATTTGTGGAAGCGATGATGTTCGGTCACCCTTTGATAATGCTACCGCTTTCGGTGGATCAAGGTCTAAATGCTCGAGTAATGGCGGATAATCAGGTGGGAATTGAGATACCAAGAAATGACGAAGATGGTTCATTCACCAAGGAGTCGGTGGCCAGATCATTGAGGTTGGTTTTAGTCGATGATGAAGGGAAGATCTACAAGGCGAAGGCGATGGAGTTGAGTCAACGATTCGGGGATAGTAAGCCGGAAAATAAGTATATAAACCCTTTTATAGACTATTTGGAACAAAAAGGTCGTGTGGTTGCTATTGAGCATGAGCTTTGA

SrUGT95A2

ATGGATACCGAAAATCAGACCAAGAAAAGAAAGCTTGAATCCACAACAATGGAAGCTGCCGGTGAGATCTTTGTTCTACCATTCTTCGGTCAAGGTCATCTCAACCCATCGATGGAACTCTGCCGGAACATCTCCTCTCATAACTTCAATGTCACACTCATCATCCCCTCTCACCTTTCTTCATCGATCCCTGCAACTTTTCCCGGCGATTCGCCCTTCATTCATGTTGCAGAGATTCCGTTCGCCGCTTCTCCGCCGGAAACAGAGGCTCCGAATTGGGGGAACCGTTTTGAACAGCAGAATAAACAGATGGGTGAGGGAATCAAGTCGTTTTTGTCGAATAGATCCGGAATCCGACCCACGTGCGTTGTGATTGATGTTATGATGAGCTCGATCAAGGAGATTTTTGCAGATCACCGGATTCCGGTGGTGTCGTTTTTCACTTCCGGCGTGACGAATTACGCCATAGAGCACGGAAAGTGGAAGGCGAAGATCGGAGACTTGAAACCCGGTGAGACCCGAGAGTTACCCGGGTTACCCAAAGAAATGGCTGCTACTTATTTGGATCTTTTCAAAGGTCCGAGAGGAAGAGCTCAGAGACCCAACCTGCCGACTGGAGATCACGCAAACCGGGTCGGACCGCCACATGGATCAAGGAGCCACCGCGGTCCACCCGGTCCGGGTGATAAGCCACGTTGGGTTGACGAAGTTGATGGAGCGGTCGCGTTGCTTATCAACACATGCGACAATCTCGAGCGTTTGTTCCTCGATTACCTCACGGAACAAACCAAGGTTCCGGTGTGGGGCGTCGGACCGCTCCTACCGGAAAAATTCTGGCAATCCGCCGGTTCAGTCCTCCACGATCGGGATATGAGATCGAATCGGAAAGCTAATTACTCAGAAGACGAAGTTTTCCAATGGCTAGAGTCCAAACCAATAGGTTCGGTGCTCTACATCGCATTCGGAAGTGAAGTTGGACCCTCGATTGATGAATACAAAGAACTAGCGAAAGCGTTGGAAGAATCGAACCACGCTTTCATATGGGTGATCCAACCCGGTTCGGGTAAAAACCCGATCCCAAAATCTTTTTTAGGACCGGTTCAAACCGATAGCGAAGAAGAACAAGAAGGTTACTATCCAGATGGTTTAGACAAAATAGTTGGGAATAGGGGTATGATCATCACCGGATGGGCCCCACAATTGTTGATTCTGAGCCACCCATCAACCGGTGGGTTCTTGTCACACTGCGGTTGGAACTCGACTGTTGAGGCGATTGGTCGAGGGGTCCCGATCTTGGGTTGGCCAATTAGGGGTGATCAGTTTGAAAATGCGAAGCTGGTGGCTAACCATCTCAAGATTGGGTTCGTGATTGCAAGTGGGGTCGGTGAAGACGGTCGGCCGAAAAGGTTCAACAAGGATGATATAGAAACAGGGGTTGATAAACTGATGAGTGATGAAGAGGTTCATGAAAATGCCAAGAAATTAAGTAAGGAGTTTGAAAGTGGGTTTCCAGTGAGTTCGGTTAATGCTTTGGGTGCATTTGTGGAGTTTATTAGCAAAAAAGCAACTTGA

**SrUGT85B4**

ATGGGTTCGGTTCAAGAGAAAAAGGCGCCACATGTTGTGTGCATACCGGCACCACTTCAAGGTCACATTAACCCGATGCTAAAACTAGCCAAAATCCTACACTCCAAAGGCTTTCTTATCACCTTTGTCAACACCGAGTTTAACCACCAACGGCTCGTTAGGTCACAGGGGGTTGAAGCCCTACACGGGCTCCCAACCTTCCGGTTCGAGACCATCCCAGATGGTCTACCGCCACCTGAAAACAAAGATGCCACCCAAGATATCCCGACTCTAGCCAAGTCGGTTGATGAAAACTTTTTGGGTCCGTTTAAAAGTCTTGTAACCAATGTGGGTGCTTTGTATGCACCCGTGACTTGTATCGTGTCTGACATGCTTATGTGCTTCACTCTTGATGCCGGTGCTGAATTGGATATCCCGGTGATACTCCATTGGACCAGTGGTACTGGTTCTTTGATATGTTACAATGAATATCCTAATCTATTGGAAAGCAAATTGATGCCCCTCAAAGATGCAAGTTATTTAGTGAATGGTTACTTAGATACGATTGTAGATTCTATCCCCATTTTGCATGGCATACGTTTAAGAGATTTCCCTCCCTTCATTAGAAAGATCTTTCCTGGTGATGAGTTCATGGTTCAATTTTTGACTTCACAAGTAAACAAAGCAAAAAACGGATCTTCTGCTATCATTTTCAACACTTTTGATGAACTAGATCGTGATGTTTTAGACACACTCGCTTCAATGTATCCTCCATGTTATGGAATTGGTCCGTTACATCTACTAGAGAAACATGTTACCGATAAATCTCTTGATTTCGTGAAATCAAACCTTTGGAAAGAAGAACCCGAATGTTTAAAATGGTTAGATACACAAGCTCCATCATCAGTCATTTATGTGAATTTTGGTAGCATTACAGTAATGACACCTCAACAACTAGTCGAGTTTTGTTGGGGACTCGCAAAGAGCAACTATCCGTTCTTATGGATAATACGACCTGACCTTGTGATTGGTGATTCCGCGATGCTTCCACCCGAGTTTGTGAAGGAAACAAGTGATAGAGGGATGCTGGTTGGATGGTGTCCTCAAGAAGAAGTTTTGAATCACCCGTCAATTGGAGGGTTTTTAACGCACAGTGGATGGAATTCAACGCTTGAAAGTATTTCGAGTGGTGTGCCGATGATTTGTTGGCCGTTTTTTGCGGATCAACAAACGAATTGCTGGTGGAGTTGCAACAAATGGGGTGTTTCCATGGAGATTGATAATAATGTAAAGAGTGATGAAGTTTCAAAGCTTGTGATTGAATTAATGGATGGAGAAAAAGGAAAGGAAATTAAAAAGAATGCCATTGACTTGAAGAATAAAGCTGAGGATGCATGTACCTCTCCTCTTGGTTCATCAGTGGTTAATTTGGAGAAAGTGGTTCAACTGATTCGTACATTTTCAAAATAA
